# Discovery and Biosynthetic Characterization of Darumycins Expand the Chemical Space of Sesterterpene Antibiotics

**DOI:** 10.1021/jacsau.6c00499

**Published:** 2026-08-19

**Authors:** Dmytro Bratiichuk, Dominik Kolling, Irma Redzic, Marc Stierhof, Franziska Fries, Josef Zapp, Rolf Müller, Maksym Myronovskyi, Jesko Koehnke, Andriy Luzhetskyy

**Affiliations:** † Department of Pharmaceutical Biotechnology, PharmaScienceHub (PSH), 9379 Saarland University, Bld. C2.3 66123, Saarbrücken, Germany; ‡ Institute of Food Chemistry, Leibniz University Hannover 30167, Hannover, Germany; § Helmholtz Institute for Pharmaceutical Research Saarland (HIPS), Helmholtz Centre for Infection Research (HZI), Saarland University 66123, Saarbrücken, Germany; ∥ Fraunhofer Institute for Translational Medicine and Pharmacology (ITMP) Immunology, Infection und Pandemic Research (IIP) 80799, München, Germany; ⊥ German Center for Infection Research (DZIF), Partner Site Hannover-Braunschweig 38124, Braunschweig, Germany

**Keywords:** antibiotics, terpenes, bioactivity, biosynthesis, In vitro characterization

## Abstract

The global rise of antibiotic resistance poses a public health challenge, emphasizing the urgent need for novel antibiotics featuring unique chemical scaffolds and mechanisms of action. Here, we report the discovery and characterization of darumycins, guanidine-containing pentacyclic sesterterpene antibiotics that exhibit potent activity against high-priority Gram-positive pathogens and mycobacteria. The darumycin biosynthetic gene cluster was identified through genome mining in the Actinobacterium *Micromonospora rubida* and heterologously expressed in *Streptomyces* chassis strains. Gene cluster engineering facilitated the discovery of novel darumycin derivatives, thereby expanding the chemical diversity within the sesterterpene class of natural products and revealing nuanced variations in their bioactivity. Targeted gene deletions, along with LC–MS and NMR analyses, enabled us to propose a darumycin biosynthetic pathway, further complemented by in vitro biochemical characterization of two O-methyltransferase tailoring enzymes, DarM and DarG. The high-resolution crystal structure of DarM in complex with SAH provided valuable insights into the enzymatic mechanism and revealed a distinct architecture compared to other methyltransferases acting on terpene scaffolds.

## Graphical abstract

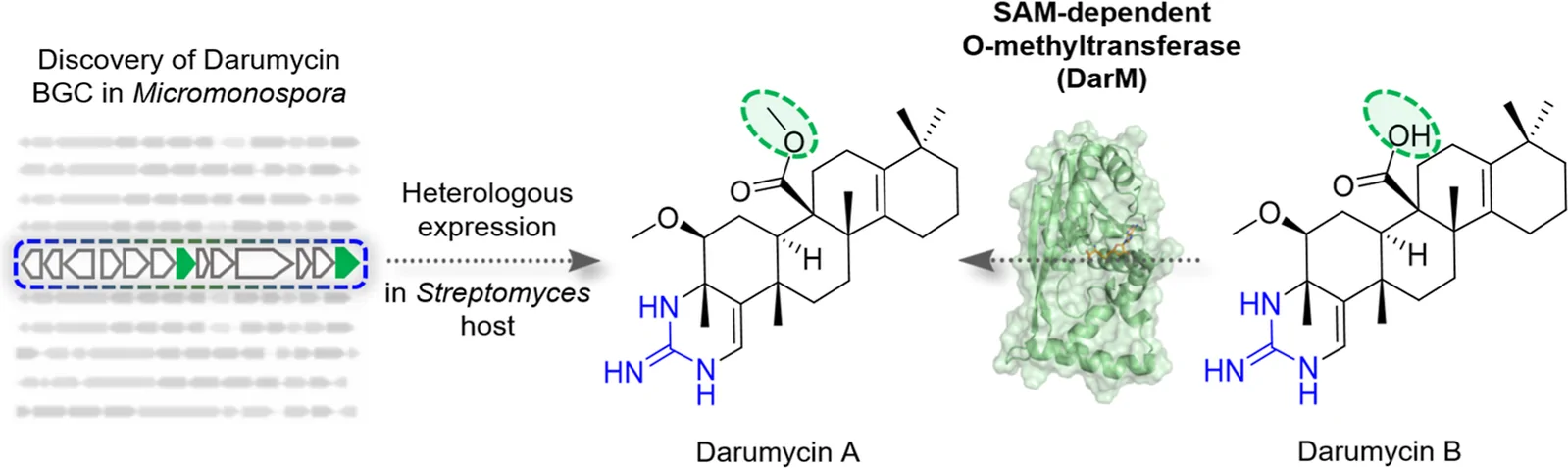

## Introduction

Actinomycetes are renowned for their unique biology and ecological roles and represent one of the most prolific sources of bioactive secondary metabolites, supporting many cornerstone discoveries in natural product chemistry.^1–5^ Among the most prominent examples is streptomycin, an aminoglycoside antibiotic isolated from *Streptomyces* spp. and historically used for the treatment of tuberculosis.^6^ A defining structural feature of streptomycin is the presence of two guanidine moieties, which play a critical role in ribosomal binding and antibacterial activity.^7,8^

Guanidine is an exceptional functional group in biological systems. Owing to its resonance-stabilized cationic character, it is among the strongest naturally occurring bases and exhibits distinctive physicochemical properties that are frequently linked to potent biological activity.^9–11^ Consequently, guanidine motifs are distributed across a wide range of chemically diverse natural product classes.^12,13^ The majority of guanidine-containing natural products comprises peptide-derived metabolites produced by nonribosomal peptide synthetases (NRPSs) or ribosomally synthesized and post-translationally modified peptides (RiPPs), in which guanidine is intrinsically present as part of the amino acid arginine.^12,14–16^ Beyond these canonical contexts, guanidine functionalities can also be enzymatically installed or modified, for example through amidinotransfer reactions, as exemplified by the biosynthesis of the cyanobacterial neurotoxin saxitoxin.^17–19^

In contrast, terpenes, despite constituting one of the largest and most structurally diverse classes of natural products,^20^ remain underrepresented as guanidine-containing metabolites. Only a handful of such compounds have been reported, including the cyanobacterial natural products cybastacines A and B,^21^ and scytoscalarol^22^ (Figure S1). These molecules belong to the sesterterpene family, which is defined by a carbon skeleton derived from five isoprene units. Sesterterpenes represent one of the rarest terpene subclasses, with only ∼1600 structurally and functionally diverse members identified to date.^23,24^ They are predominantly isolated from plants,^25^ fungi,^26,27^ and marine sponges,^28–30^ with comparatively few examples reported from bacteria, primarily within Cyanobacteria^21,22^ and Actinobacteria.^31–34^

The unusual combination of a sesterterpene scaffold with a guanidine functional group, together with the reported bioactivities of cybastacines^23^ and scytoscalarol,^35,36^ highlights this compound class as a largely unexplored chemical space. However, the lack of genomic information for the original producer strains has long hindered elucidation of their biosynthetic origins. This situation changed with the recent discovery of gromomycin, a structurally related nonsqualene cyclic guanidine-containing terpene.^37^ Identification of the gromomycin biosynthetic gene cluster (BGC) in *Streptomyces* sp. Je 1–332, combined with targeted gene deletions and isotope-labeling experiments, enabled the proposal of a biosynthetic framework for cyclic guanidine-containing terpenes. Notably, gromomycin displays potent antibacterial activity against high-priority Gram-positive pathogens and mycobacteria, establishing this scaffold as mechanistically and chemically distinct from conventional antibiotics.^37^ Key biosynthetic enzymes were identified within the gromomycin BGC, including the polyprenyl diphosphate synthase GroD, the prenyltransferase GroH, and the terpene cyclase GroF.

Guided by this biosynthetic blueprint, we sought to explore the broader chemical space of guanidine-containing terpenes through a genome-mining approach, while retaining antibacterial potency. Here, we report the discovery of the darumycins, a new family of guanidine-containing sesterterpene antibiotics identified in *M. rubida* and produced heterologously in optimized *Streptomyces* chassis strains. Structural elucidation revealed that the darumycins share a sesterterpene core architecture with gromomycin but feature a distinct pattern of biosynthetic tailoring, suggesting a potential basis for modulation of antibacterial activity.

## Results and Discussion

### Genome Mining, Heterologous Expression, and Identification of Darumycin

Gromomycins constitute a recently identified class of bacterial guanidine-containing triterpenes featuring a pentacyclic scaffold assembled through a nonsqualene biosynthetic pathway.^37^ Their biosynthesis proceeds via a linear hexaprenylguanidine precursor and culminates in an unusual cyclization reaction. These distinctive structural and biosynthetic features, together with their potent activity against drug-resistant pathogens, motivated a systematic search for related natural products.

To identify additional gromomycin-like biosynthetic gene clusters (*gro*BGCs), we performed a genome-mining analysis using the three hallmark biosynthetic genes of the gromomycin pathway, *groD*, *groH*, and *groF*, encoding a polyprenyl diphosphate synthase, a guanidine transferase, and a terpene cyclase, respectively, as search queries. Homology searches using tBLASTn against publicly available genome databases revealed numerous candidate BGCs predominantly in *Streptomyces* species, as well as a smaller number of related clusters in *Kitasatospora*, *Lentzea*, *Dactylosporangium*, and *Micromonospora*. While most *Streptomyces*-derived clusters closely resembled the canonical gromomycin BGC, several clusters from less common genera displayed distinct architectural features, including additional genes predicted to encode tailoring enzymes, suggesting the potential for structurally diversified analogs (Figure 1A). A comprehensive analysis of the distribution and diversity of *gro*BGCs has since been reported separately.^38^

**Figure 1:**
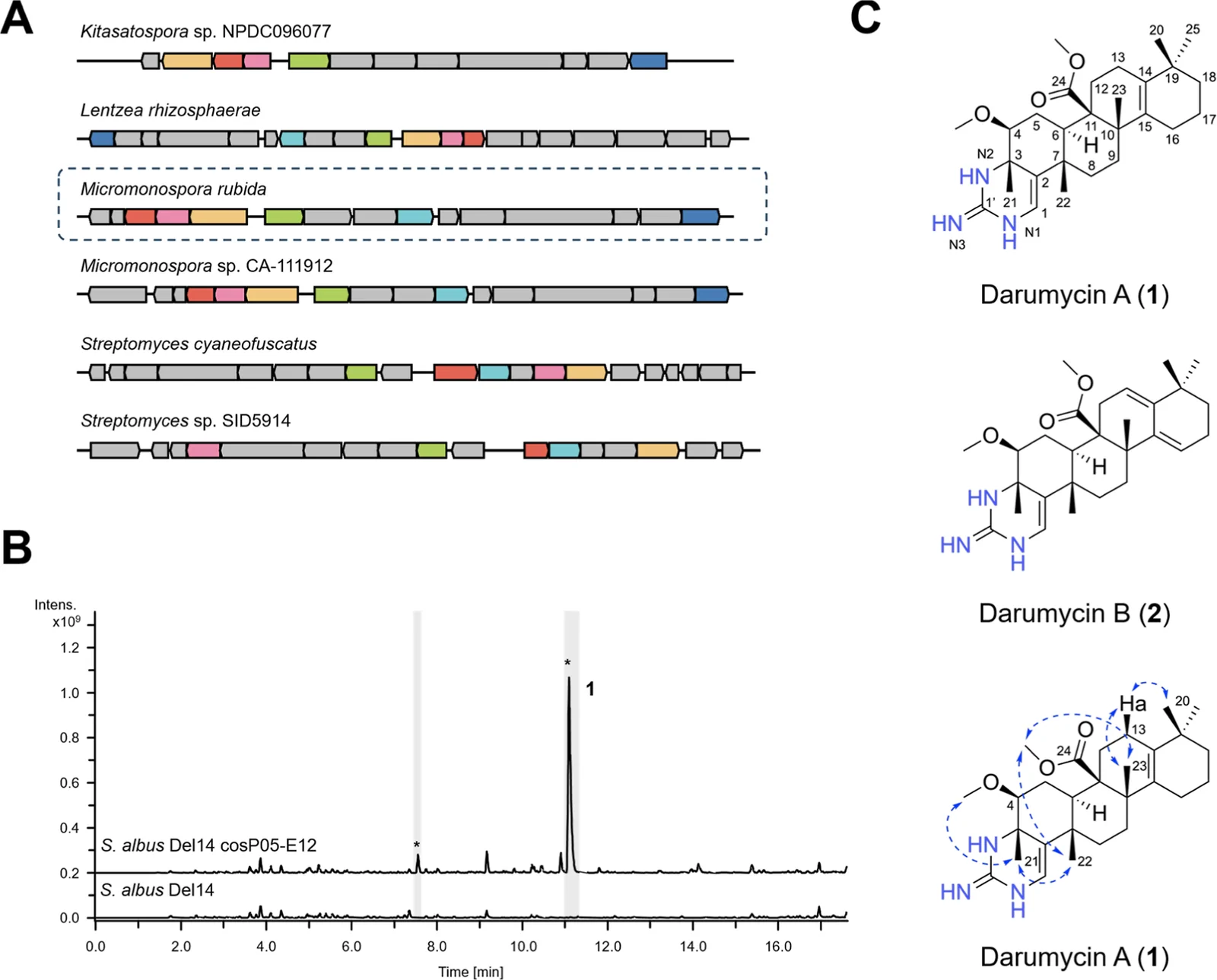
Genome mining-guided discovery of darumycins. (**A**) Gromomycin-like BGCs identified in non-*Streptomyces* strains. Homologues of the biosynthetic key genes *groD*, *groF*, and *groH* are highlighted in green, red, and pink. The gene encoding for the highly conserved cytochrome P450 oxygenase is colored in beige. Additional potential biosynthetic genes not present in the gromomycin BGC are colored in blue and light-blue. (**B**) LC–MS chromatograms showing darumycin production in the heterologous host strain *S. albus* Del14 harboring the cosmid P05-E12. The novel peaks corresponding to darumycin A (**1**) and metabolite *m*/*z* 486.33 [M + H]^+^ (later degrades to darumycin B (**2**) during purification) are highlighted by asterisks. (**C**) Chemical structure of the novel guanidine-containing sesterterpenes darumycin A (**1**) and darumycin B (**2**) and NOESY correlations for assignment of the relative stereochemistry for darumycin A (**1**).

Among these candidates, we selected *M. rubida* JCM 32386 (Japan Collection of Microorganisms, RIKEN BRC)^39^ for experimental characterization. A cosmid library of *M. rubida* genomic DNA was constructed using the φC31-based integrative vector cos15AAmInt, and PCR screening identified cosmid P05-E12 containing a 39.2 kb genomic fragment (RefSeq GCF_009908295.1) encompassing a putative *gro*BGC. Introduction of cosmid P05-E12 into the heterologous host strains *Streptomyces albus* Del14 (now *Streptomyces albidoflavus* Del14),^40^ and *Streptomyces lividans* DelYA9 ^41^ resulted in the appearance of two new metabolites with molecular ions at *m*/*z* 470.34 and 486.33 [M + H]^+^ (calculated molecular formulas C_28_H_44_N_3_O_3_ and C_28_H_44_N_3_O_4_), which were absent from control strains lacking the cosmid (Figures 1B, S2 and S3A). Notably, both metabolites were also detected in extracts of the native *M. rubida* strain (Figure S3B,C). Compared to the previously reported triterpene gromomycins A and E (*m*/*z* 478.38 and 492.39 [M + H]^+^),^37^ the observed mass differences suggested related but distinct chemical structures, likely arising from differences in chain length and/or postcyclization tailoring.

For structural characterization, *S. albus* Del14 harboring cosmid P05-E12 was cultivated at a 10 L scale in DNPM medium. Extraction and chromatographic purification yielded two new compounds. Detailed analysis of 1D and 2D NMR data for the metabolite with *m*/*z* 470.34 [M + H]^+^ revealed a pentacyclic guanidine-containing terpene scaffold closely related to that of gromomycin (Table S1, Figures S4–S11). Key similarities were established by HMBC and ^15^N-HMBC correlations confirming the cyclic guanidine motif. In contrast, ^1^H–^1^H COSY and HMBC data established a carbon framework composed of five fused rings with a conserved methylation pattern. In contrast to gromomycins, the absence of an additional prenyl unit unambiguously classified the compound as a sesterterpene rather than a triterpene (Figure 1C). Further structural differences were observed at the periphery of the scaffold, including an O-methyl ester at C-24, an O-methyl group at C-4, and a double bond between C-14 and C-15. Relative stereochemistry, determined by NOESY analysis, matched that of gromomycins (Figure 1C). This compound was designated darumycin A **(1)**, and the corresponding gene cluster was termed the darumycin BGC.

The purification of the second metabolite with *m*/*z* 486.33 [M + H]^+^ resulted in the isolation of the compound with 468.32 [M + H]^+^ (calculated molecular formula C_28_H_42_N_3_O_3_). This 18 Da mass difference is consistent with dehydration of the 486.33 [M + H]^+^ metabolite. Since the corresponding *m*/*z* 486.33 [M + H]^+^ mass was also detected in the native producer, we suggest that it represents a genuine biosynthetic metabolite rather than an artifact arising from heterologous expression. The *m*/*z* 468.32 [M + H]^+^ compound, formed during purification, shares the same pentacyclic core scaffold as darumycin A but differs in the position of unsaturation within the C/D ring system, as revealed by NMR analysis (Table S2, Figures S12–S18). This compound was named darumycin B (**2**).

### Bioinformatic Analysis of the Darumycin BGC and Targeted Gene Deletions

The structural similarity between gromomycin and the newly identified darumycins, together with conservation of hallmark biosynthetic genes, suggests a shared biosynthetic logic for these guanidine-containing terpenes. In contrast to gromomycin, however, darumycin A (**1**) and B (**2**) are sesterterpenes and feature two distinct O-methylations (at C-4 and C-24), indicating additional late-stage tailoring chemistry. To delineate the genetic basis of these modifications and to assign functions across the pathway, we performed bioinformatic analysis of the 39.2 kb *M. rubida* genomic fragment captured on cosmid P05-E12 and conducted targeted gene deletions in the heterologous production system.

NCBI BLAST analysis identified 31 open reading frames (ORFs; *dar1*–*dar31*) within the cloned fragment (Figure S19). Based on cluster context and similarity to the gromomycin pathway, we delineated a minimal darumycin BGC comprising 15 genes (*dar3*–*dar17*). Within this region, genes *darA*–*darG*, *darI*, and *darM* (corresponding to genes 5–11, 13, and 17 in the cloned fragment) were assigned as putative biosynthetic/structural genes, whereas *dar3*, *dar4*, *darH*, and *darJ*–*darL* (genes 3, 4, 12, and 14 – 16) were tentatively assigned regulatory or auxiliary roles based on homology and conservation across gromomycin-like clusters (Table S3).

Homologues of the core gromomycin biosynthetic triad (*groD*, *groH*, *groF*) are conserved and clustered within the left region of the darumycin BGC. Specifically, *darD* encodes a polyprenyl diphosphate synthase (GroD homologue), *darB* encodes a guanidine transferase/prenyltransferase (GroH homologue), and *darA* encodes the terpene cyclase (GroF homologue). The gene *darC*, located downstream of *darD*, encodes a cytochrome P450 oxygenase, consistent with oxidative tailoring steps described in related systems. The central region contains three conserved Rieske-type iron–sulfur proteins (DarE, DarF, DarI), closely related to GroA–C, which have been implicated in the formation and/or functionalization of the cyclic guanidine terpene scaffold.

A key distinguishing feature of the darumycin BGC relative to the gromomycin BGC is the presence of two genes annotated as SAM-dependent methyltransferases: *darG* (positioned between *darF* and *darI*) and *darM* (near the right boundary of the minimal cluster) (Figure 2A). Given the O-methyl groups observed in the darumycin structures, we hypothesized that DarG and DarM catalyze site-selective methylation during late-stage scaffold tailoring. Genes located downstream of *darM* and including *darH* are unlikely to encode direct biosynthetic enzymes for darumycin assembly; however, homologues of *dar3*, *darH*, *darJ*, and *darL* are conserved across multiple *gro*BGCs (*dar3*–*groG*, *darH*–*gro3*, *darJ*–*gro5*, *darL*–*gro4*), suggesting roles in pathway regulation or physiological integration.

**Figure 2:**
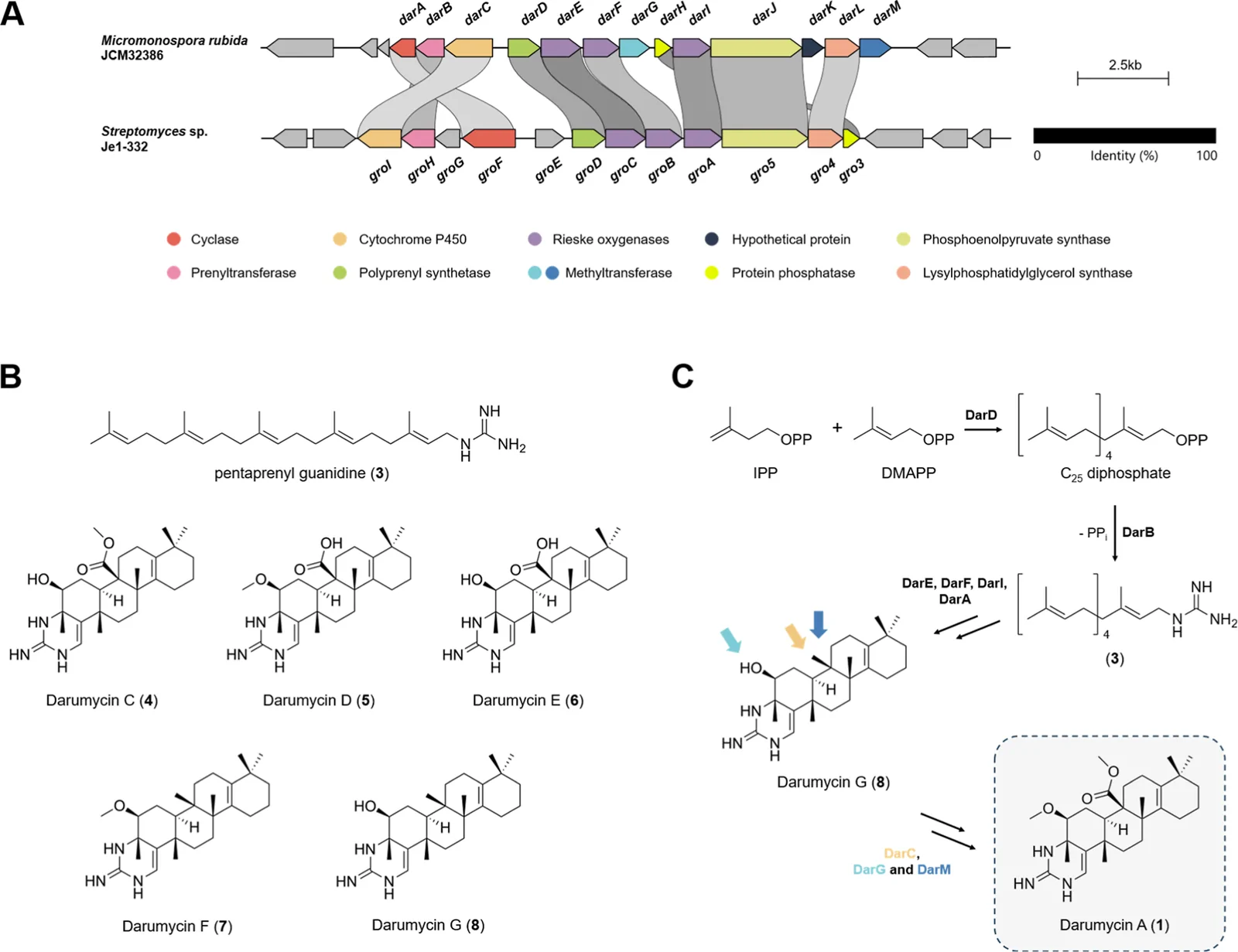
Targeted gene deletions within the darumycin BGC reveal biosynthetic intermediates and propose a biosynthetic logic. (**A**) Comparison between the *dar* BGC (top) from *M. rubida* producing darumycin and the *gro* BGC (bottom) producing gromomycin. Biosynthetic core genes are colored and the putative function of encoded enzymes is shown below. Other potential regulatory genes are colored in gray. The two genes encoding for SAM-dependent methyltransferases only present in the *dar* BGC are highlighted in blue and light-blue. (**B**) Structure of biosynthetic darumycin intermediates identified through gene knockout experiments. (**C**) Proposed biosynthesis of darumycin A (**1**). Both compounds, the linear pentaprenyl guanidine (**3**) and darumycin G (**8**), serve as key biosynthetic intermediates in darumycin biosynthesis, followed by distinct tailoring catalyzed by DarC, DarG and DarM.

To functionally assign genes and identify biosynthetic intermediates, we generated targeted deletions directly on cosmid P05-E12 by Red/ET recombination using an ampicillin resistance cassette (*bla*) as a temporary selection marker, followed by marker excision to minimize polar effects. In total, eight mutant cosmids harboring single or double deletions were constructed (Table S16) and introduced into *S. albus* Del14 and *S. lividans* DelYA9 (Table S15). Metabolites were analyzed by LC–MS following cultivation and extraction (Figure 2B).

Deletion of *darA* (terpene cyclase) resulted in the accumulation of a new metabolite with *m*/*z* 400.37 [M + H]^+^ (Figures S20A and S21). Isolation and NMR-based structure elucidation (Table S4, Figures S22–S26) revealed a linear C_25_ isoprenoid bearing a terminal guanidine group. The presence of five olefinic methines and six methyl groups was consistent with a five-isoprene-unit backbone, and key HMBC correlations supported guanidine substitution. Combined MS and NMR analysis established this compound as pentaprenylguanidine (**3**). This intermediate parallels early steps in gromomycin biosynthesis and supports a model in which DarD produces pentaprenyl diphosphate (chain-length determination), followed by guanidine installation by DarB to yield (**3**), which is then cyclized by DarA. To further support the proposed role of the conserved Rieske proteins (DarE, DarF, DarI) in pentacyclic darumycin scaffold formation, a Δ*darI* mutant was generated. LC–MS analysis of this mutant revealed the accumulation of a metabolite with *m*/*z* 400.37 [M + H]^+^ (Figure S20B), corresponding to the pentaprenylguanidine (**3**) previously observed upon deletion *darA* gene.

The structures of darumycin A (**1**) and B (**2**) indicated methylation at two positions: a methoxy group at C-4 and an O-methyl ester at C-24. Consistent with the presence of two methyltransferases in the BGC, deletion of either *darG* or *darM* yielded distinct monodemethylated products, each with *m*/*z* 456.32 [M + H]^+^ (calcd for C_27_H_42_N_3_O_3_) but different retention times. NMR analysis unambiguously assigned the structural differences: Δ*darG* accumulated darumycin C (**4**) lacking the C-4 O-methyl group (Table S5, Figures S27–S33), whereas Δ*darM* accumulated darumycin D (**5**) lacking methylation of the C-24 carboxyl group (Table S6, Figures S34–S40). The availability of the C-4 hydroxy group in (**4**) further enabled assignment of absolute configuration by Mosher ester analysis (Table S7, Figures S41–S46).

To confirm that DarG and DarM account for the two methylations, we constructed a Δ*darG*/Δ*darM* double mutant. This strain produced darumycin E (**6**) with *m*/*z* 442.31 [M + H]^+^ (calcd. for C_26_H_40_N_3_O_3_) (Figures S47 and S48). The −28 Da mass shift relative to (**1**) and NMR analysis (Table S8, Figures S49–S53) confirmed loss of both methyl groups.

We next interrogated the role of the gene *darC*, presumably encoding for cytochrome P450. Deletion of *darC* yielded a new metabolite with *m*/*z* 426.35 [M + H]^+^ (calcd. for C_27_H_44_N_3_O) (Figure S54), and NMR analysis demonstrated the absence of the C-24 carboxylate/ester functionality, establishing darumycin F (**7**) (Table S9, Figures S55–S60). Together with the Δ*darM* phenotype, these results support a model in which DarC installs the C-24 carboxyl group by sequential oxidation, followed by methyl ester formation catalyzed by DarM.

To obtain a minimally tailored cyclic scaffold, we generated a Δ*darC*/Δ*darG* double mutant, which produced darumycin G (**8**) with *m*/*z* 412.33 [M + H]^+^ (calcd. for C_26_H_42_N_3_O) (Figure S61). Comprehensive NMR analysis (Table S10, Figures S62–S67) confirmed the absence of both O-methylation and the C-24 oxidation state changes. Notably, the presence of a C-4 methoxy group in darumycin F (**7**), despite lacking the C-24 carboxylate, suggests that DarG can act on multiple scaffold oxidation states, consistent with its activity on both (**8**) and (**6**) (see below).

Beyond core biosynthetic and tailoring genes, we investigated *darK*, annotated as a hypothetical protein without a direct counterpart in the gromomycin BGC. Protein homology detection using the HHpred interactive server^42^ indicated remote similarity to COQ4-family proteins involved in ubiquinone biosynthesis. Inactivation of *darK* led to a marked reduction in darumycin production (Figure S68), suggesting an auxiliary role in pathway efficiency and/or regulation rather than direct scaffold construction.

Integrating bioinformatic analysis with intermediate profiling, we propose the following pathway for darumycin biosynthesis (Figure 2C): DarD catalyzes the formation of pentaprenyl diphosphate, establishing the C_25_ chain length. DarB then installs guanidine to yield pentaprenylguanidine (**3**), which is cyclized by DarA to generate the polycyclic guanidine terpene core. The Rieske proteins DarE/DarF/DarI, conserved across related pathways, are likely involved in the cyclization process and/or in installation of the C-4 hydroxyl group, although the precise roles of these proteins remain unresolved. The cyclic intermediate darumycin G (**8**) represents a minimally tailored scaffold that undergoes late-stage diversification: DarG catalyzes O-methylation at C-4, while DarC installs the C-24 carboxyl group via sequential oxidation, which is subsequently converted to the methyl ester by DarM. Oxidation of a methyl group to a carboxylate by cytochrome P450 enzymes has precedent in bacterial natural product biosynthesis, including XiaM in xiamycin biosynthesis^43,44^ and RosC in rosamicin biosynthesis.^45^

### In Vitro Characterization of Darumycin Methyltransferases and Tailoring Pattern

Bioinformatic analysis and targeted gene deletions identified two SAM-dependent methyltransferases, DarG and DarM, as tailoring enzymes responsible for O-methylation of the darumycin scaffold. While the genetic data clearly established site selectivity, DarG for the C-4 hydroxyl group and DarM for the C-24 carboxyl group, the temporal order of these methylation events remained unresolved. To further define enzyme specificity and substrate scope, both DarG and DarM were heterologously expressed in *E. coli*, purified to homogeneity, and characterized in vitro using isolated darumycin biosynthetic intermediates (Figure 3A, B).

**Figure 3:**
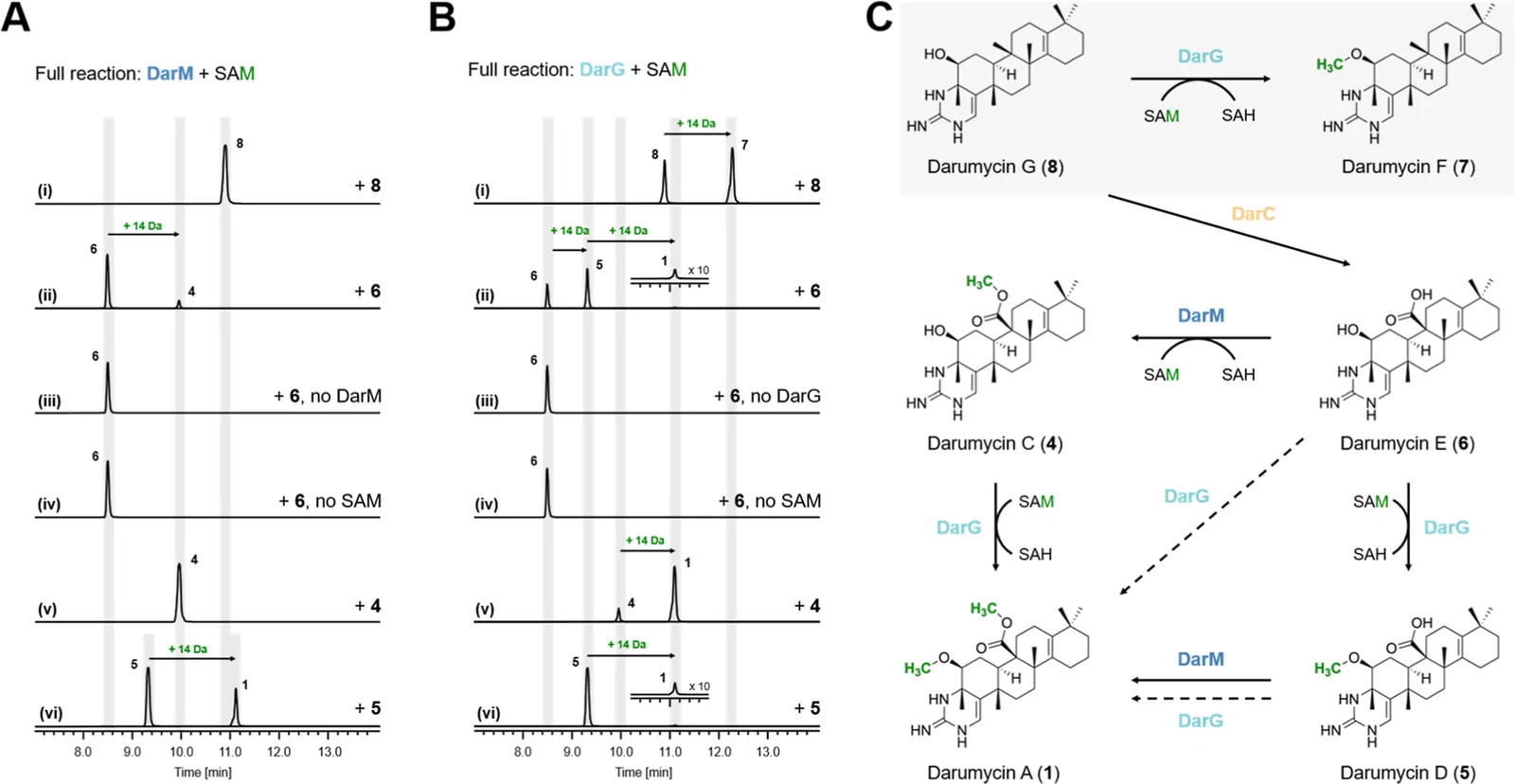
Biochemical characterization of the two SAM-dependent methyltransferases DarG and DarM involved in darumycin biosynthesis. (A) In vitro reaction of DarM and unmethylated darumycin biosynthetic intermediates. Extracted ion chromatograms (EICs) revealed SAM-dependent conversion of darumycin D (**5**) and darumycin E (**6**). (**B**) In vitro reaction of DarG and unmethylated darumycin precursors. EIC traces indicated the efficient SAM-dependent methylation of darumycin G (**8**), darumycin C (**4**) as well as darumycin E (**6**). Darumycin A (**1**) was detected in trace amounts in a one-pot reaction of DarG and darumycin E (**6**) (**C**) Tailoring steps in darumycin biosynthesis based on in vitro characterization of DarG and DarM. While DarM appears to be site-specific, acting on the C-24 carboxyl group, DarG exhibits a rather relaxed specificity.

We first assessed methyltransferase activity using the minimally tailored cyclic intermediate darumycin G (**8**) as the substrate. Incubation of **8** with DarG in the presence of S-adenosyl-l-methionine (SAM) resulted in the quantitative formation of darumycin F (**7**), corresponding to O-methylation at C-4, as determined by LC–MS analysis (Figure S69). No conversion was observed in the absence of SAM, in control reactions lacking enzyme, or upon incubation with DarM, indicating that DarG alone catalyzes C-4 O-methylation of the unoxidized scaffold.

We next examined methylation of the oxidized intermediate darumycin E (**6**), which bears a free carboxyl group at C-24. In accordance with the gene deletion phenotypes, DarG catalyzed site-selective methylation of the C-4 hydroxyl group to yield darumycin D (**5**), whereas DarM exclusively catalyzed methyl ester formation at C-24 to generate darumycin C (**4**) (Figures S70 and S71). These reactions were strictly SAM-dependent. Notably, closer inspection of the LC–MS data revealed the presence of a minor product in reactions containing DarG and darumycin E (**6**). Exact mass determination and retention time comparison confirmed this species as darumycin A (**1**), indicating that DarG is capable of catalyzing both O-methylation reactions on the same substrate, albeit with a strong preference for the C-4 hydroxyl group.

This unexpected activity demonstrates that darumycin A (**1**) can be formed in a single in vitro reaction catalyzed by DarG alone, although only in low yield. To further probe pathway connectivity, we evaluated the singly methylated intermediates darumycin C (**4**) and darumycin D (**5**) as substrates. Incubation of darumycin C (**4**) with DarG resulted in efficient conversion to darumycin A (**1**), whereas no conversion was observed with DarM. Conversely, darumycin D (**5**) was selectively converted to darumycin A (**1**) by DarM, with only trace activity observed for DarG (Figures S72 and S73).

Collectively, these *in vitro* data establish that both DarG and DarM are SAM-dependent O-methyltransferases with complementary but distinct substrate specificities. DarM displays strict site selectivity for carboxyl methylation, whereas DarG exhibits broader substrate tolerance and can catalyze methylation at both the hydroxyl and carboxyl positions, with a pronounced preference for C-4 O-methylation (Figure 3C). Despite this functional overlap, the enzymes do not operate obligatorily in a fixed sequence. Accordingly, while the biochemical data firmly establish the tailoring roles of DarG and DarM, they do not allow unambiguous assignment of the chronological order of methylation events in vivo.

### Crystal Structure of DarM Methyltransferase in Complex with SAH

The distinct substrate specificities observed for the two darumycin methyltransferases prompted us to investigate their structural basis. Sequence-based searches against the Protein Data Bank revealed several close structural homologues of DarG, whereas DarM showed fewer and more distantly related matches, suggesting that DarM may represent a less common subclass of SAM-dependent methyltransferases. In light of its strict site selectivity and unique functional role, we therefore focused our structural studies on DarM.

Crystals of DarM suitable for X-ray diffraction were obtained exclusively in the presence of S-adenosyl-l-homocysteine (SAH), and the structure of the binary DarM–SAH complex was determined to 1.5 Å resolution (Figures 4A and S74). DarM adopts the canonical class I methyltransferase fold, characterized by a central seven-stranded β-sheet flanked by α-helices.^46,47^ The parallel β-strands (β1−β6) form the core Rossmann fold, whereas two extended antiparallel β-strands (β7 and β8) connect to an appended α-helical bundle that likely contributes to substrate recognition and binding.^48^

**Figure 4:**
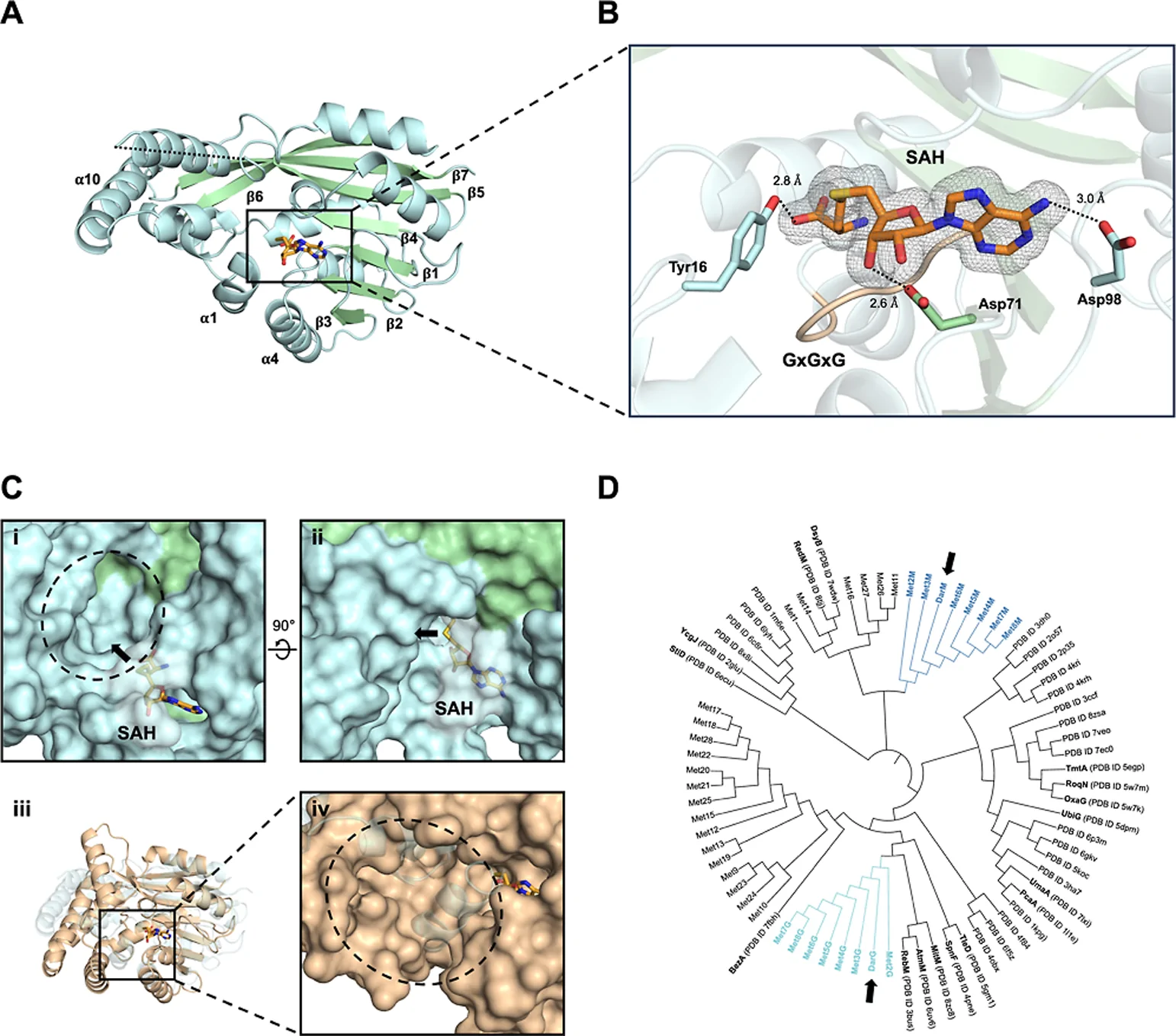
Structural investigation of DarM in complex with SAH and phylogenetic analysis of methylransferases embedded in *gro*BCGs (A) Crystal structure of DarM in complex with SAH. The cartoon representation of DarM (light blue) displays a characteristic Rossmann fold (β sheets colored in green), thereby classifying DarM as member of class I methyltransferases. (B) Detailed view on SAH binding mode within the active site of DarM. SAH is represented as sticks (orange) and the F_O_-F_C_ omit electron density map (gray mesh) is contoured at 3σ. The highly conserved GxGxG motif is shown in wheat and amino acid residues engaged in SAH binding are depicted as sticks and labels by three-letter-code. Hydrogen bond interactions are highlighted as dashed lines and the distance is shown in Å. (C) Surface representation of DarM substrate binding pocket and structural comparison with an AlphaFold3 model of DarG. The sulfur atom of SAH directs to a narrow hydrophobic cavity responsible for darumycin substrate binding (i, ii). Superposition of DarM structure in complex with SAH (light blue) and an AlphaFold3 model of DarG (wheat) revealed conservation of the Rossmann fold and SAM binding (iii). Closer inspection of DarG’s potential substrate pocket indicated a larger, more solvent-exposed cavity (iv). (D) Phylogenetic tree of methyltransferases embedded in *gro*BGC as well as homologues of DarG and DarM identified in a structure-based DALI search. Both enzymes, DarG and DarM, are highlighted by an arrow and DarM homologues are depicted in blue, whereas DarG homologues are colored in light blue. Enzymes involved in natural product biosynthesis are shown in bold letters and the PDB code for experimental structures is given in parentheses.

The SAH cofactor is bound in a conserved pocket typical of class I methyltransferases^49^ (Figure 4B). The carboxyl group of the homocysteine moiety forms a hydrogen bond with the hydroxyl group of Tyr16, while the α-amino group interacts with the backbone carbonyl of Gly47 at the N-terminus of the conserved GxGxG motif (Gly47-Thr48-Gly49-Arg50-Gly51). The ribose hydroxyl group engages in a hydrogen bond with the side chain of Asp71,^50^ and the adenine ring is stabilized by interactions with residues located in the loop connecting β3 and β4, including a hydrogen bond between the adenine amino group and Asp98, as well as a backbone interaction involving Gly99.

Based on its classification as a class I methyltransferase, DarM is expected to catalyze methyl transfer via a canonical S_N2_ mechanism, in which the activated methyl group of SAM is positioned for direct nucleophilic attack by the acceptor oxygen.^51,52^ Inspection of the DarM–SAH complex revealed a narrow tunnel extending from the SAH sulfur atom toward a hydrophobic cavity positioned at the C-terminal end of helix α9. A flexible and partially unresolved loop spanning residues Asp168–Asp180 was observed at the N-terminal edge of the α-helical bundle. This loop covers the entrance to a hydrophobic cavity and is likely to function as a dynamic gate controlling substrate access to the active site. Comparison of the crystal structure with an AlphaFold3-predicted model of DarM revealed conformational differences in this region, suggesting that cofactor binding may induce structural rearrangements that regulate substrate accommodation (Figure S75). In-depth analysis of the DarM electrostatic surface identified a hydrophobic pocket directly linked to the SAH-binding cavity, with SAH sulfur atom oriented toward the hydrophobic site via a narrow tunnel. Structural superposition with the homologous methyltransferase DnrK (PDB code 1tw2, R.M.SD = 2.8 Å) complexed with both SAH and substrate further defined the location of the putative DarM substrate-binding cavity (Figure S75). The identified pocket is predominantly lined by hydrophobic residues (Phe24, Phe25, Trp223, and Leu229), indicating a highly constrained substrate-binding pocket that enforces a single productive binding mode and is consistent with the strict site selectivity of DarM observed in vitro. To map residues engaged in substrate binding in more detail, we modeled darumycin D (**5**) into the DarM-SAH cocrystal structure. Docking results verified the importance of the previously identified hydrophobic amino acid side chains (notably Phe24 and Leu229) and further predicted a hydrogen bond between the darumycin D (**5**) carboxylate moiety and Gln227 together with a salt bridge between the guanidium group and Asp186. To further probe their role in substrate binding, we generated single-site mutants of these residues and assayed activity in vitro using darumycin D (**5**) as substrate (Figure S76). Conservative (Asp186Asn) and nonpolar (Asp186Ala) substitutions at the Asp186 position retained activity comparable to the wild-type, supporting a dominant role for hydrophobic interactions in substrate recognition. In contrast, Gln227Ala, which abolished the predicted hydrogen bond to the carboxylate moiety, caused a substantial activity loss (∼40%). To probe steric constraints, we substituted Ph24, Leu229 and Ala185 for bulkier amino acid residues, displacing the substrate and thereby resulting in less efficient catalytic conversion. Leu229Phe and Phe24Trp mutations reduced conversion to ∼60% and ∼50% compared to the DarM wild-type. Ala185Trp impaired efficient displacement of darumycin D (**5**) from the hydrophobic pocket, consistent with the increased steric hindrance (Figure S76).

In contrast to DarM, attempts to crystallize DarG were unsuccessful. An AlphaFold3-generated model of DarG nonetheless revealed a canonical class I methyltransferase fold closely resembling that of DarM. Structural superposition showed good agreement in the Rossmann core and the SAH-binding region, whereas pronounced differences were observed in the α-helical bundle implicated in substrate recognition (RMSD 5.852 Å). In particular, the putative substrate-binding pocket in DarG is substantially larger and more solvent-exposed than that of DarM, consistent with the broader substrate tolerance and relaxed site selectivity observed for DarG in vitro (Figure 4C).

To further contextualize these findings, we performed a structure-based DALI search using the DarM crystal structure and the DarG AlphaFold3 model (Table S11 and S12).^53,54^ This analysis identified related methyltransferases involved in natural product biosynthesis, including enzymes from rebeccamycin and mitomycin pathways (Figures S77 and S78).^55,56^ Phylogenetic analysis incorporating these homologues and methyltransferases from *gro*BGCs revealed clear separation between DarM-like and DarG-like enzymes, supporting functional divergence within this tailoring enzyme family (Figure 4D, Table S13).

Together, the structural and computational analyses provide a clear molecular rationale for the divergent substrate specificities of the two darumycin methyltransferases. DarM employs a narrowly defined and sterically constrained substrate-binding pocket that enforces strict site selectivity for carboxyl methylation, whereas DarG features a more spacious and adaptable binding architecture consistent with its broader substrate tolerance. This structural divergence mirrors the functional plasticity observed in vitro and highlights how subtle architectural variations within a conserved class I methyltransferase framework can enable diversification of terpene scaffolds through late-stage tailoring. These findings underscore the role of methyltransferase evolution as a key driver of chemical diversity in guanidine-containing sesterterpene biosynthesis.

### Bioactivity Profiling of Darumycins

Darumycins A (**1**) and B (**2**) display antibacterial activity profiles that closely mirror those of the gromomycins,^37^ exhibiting potent activity against high-priority Gram-positive pathogens and mycobacteria. Activity against Gram-negative bacteria is limited, consistent with restricted outer membrane penetration and efflux-mediated resistance mechanisms, as evidenced by reduced MIC values against efflux-deficient and outer membrane-permeabilized *E. coli* strains. As observed for gromomycins, darumycins also demonstrate pronounced cytotoxicity, underscoring a nonselective bioactivity profile (Table 1).

**Table 1:** Antimicrobial and Toxicity Spectrum of Darumycins. Minimum Inhibitory Concentrations (MICs) Were Determined Using the Broth Microdilution Method in Accordance with Eucast Guidelines^a^

organism	MIC [μg/mL]								
	(1)	(2)	(3)	(4)	(5)	(6)	(7)	(8)	REF^d^
S. aureus ATCC29213	2	2	8	4	16	32	1	1	CIP: 0.5
S. aureus Newman	2	2	4	8	16	32	2	1	CIP: 0.5
S. aureus Cowan 1	2	2	8	8	16	32	1	1	CIP: 1
S. aureus N315	2	2	8	4	16	32	1	1	CIP: 1, AMP: >64
S. aureus Mu50	4	4	4	4	32	32	1	2	VAN: 8, AMP: >64
S. aureus HG001 WT	2	2	8	4	16	32	2	2	DAP: 0.5
S. aureus HG001 Dap^R^	2	2	2	2	8	32	1	1	DAP: 32
S. pneumoniae DSM11865	2	2	4	4	8	16	1	4	CIP: 1
E. faecalis ATCC29212	2	2	4	4	16	64	1	2	CIP: 2
B. subtilis DSM10	2	2	8	4	16	32	1	1	CIP: 0.25
M. smegmatis mc^2^ 155	2	2	4	2	16	32	2	4	RIF: 32
M. tuberculosis H37Ra	4	4	8	8	32	64	2	8	RIF: 0.02
E. coli BW25113	64	64	>64	16	>64	>64	32	16	CIP: 0.03
E. coli K12 ΔtolC^b^	8	8	>64	8	16	16	4	4	CIP: 0.01
E. coli K12 ΔtolC^b^ + PMBN^c^	2	2	64	2	4	16	1	1	CIP: 0.01
E. coli WO153	2	2	>64	4	8	16	2	2	CIP: 0.01
S. enterica DSM5569	>64	>64	>64	16	>64	>64	16	8	CIP: 0.02
C. freundii DSM30039	64	64	>64	16	>64	>64	16	8	CIP: 0.02
K. pneumoniae DSM681	8	8	>64	8	32	64	4	4	CIP: 0.01
A. baumannii DSM30007	16	16	>64	16	>64	>64	4	4	CIP: 1
A. baumannii DSM30008	16	16	>64	16	>64	>64	4	4	CIP: 0.5
A. baumannii NCTC13301	16	16	>64	16	>64	>64	8	8	CIP: >64, COL: 1
P. aeruginosa PA14	>64	>64	>64	64	>64	>64	>64	64	CIP: 0.25
cell line	IC_50_[μg/mL]								
HepG2	8.95 ± 3.6	14.3 ± 3.9	4.65 ± 0.7	17.5 ± 2.9	>37	>37	5.5 ± 0.3	14.7 ± 3.7	DOX: 0.1 ± 0.02
CHO–K1	>37	32.7 ± 1.5	0.8 ± 0.05	21.3 ± 1.8	>37	>37	4.6 ± 0.05	23.4 ± 0.03	DOX: 0.01 ± 0.00
Species	MTC [μg/mL]								
Danio rerio	5	1	1	5	10	>25	1	1	-

a Minimum Inhibitory Concentrations (MICs) Were Determined Using the Broth Microdilution Method in Accordance with Eucast Guidelines. In vitro cytotoxicity was evaluated performing a MTT assay (half maximal inhibitory concentration IC_50_ ± standard deviation). In vivo toxicity was assessed by determination of the maximum tolerated concentration (MTC) in zebrafish (*Danio rerio*) embryos. CHO, Chinese hamster ovary.

b Keio collection mutant; efflux-deficient.

c 3 μg/mL polymyxin B nonapeptide (PMBN).

d Reference compounds: AMP, ampicillin; CIP, ciprofloxacin; COL, colistin; DAP, daptomycin; DOX, doxorubicin; RIF, rifampicin; VAN, vancomycin.

Notably, the linear precursor pentaprenylguanidine (**3**) shows only a modest reduction in activity relative to the cyclized metabolites, with MIC values only marginally higher than those of darumycins A (**1**) and B (**2**). This observation suggests that cyclization of the guanidine-containing terpene scaffold is not strictly required for antibacterial activity, although it does provide a measurable enhancement. In contrast, removal of methyl groups from the darumycin scaffold resulted in a pronounced decrease in antibacterial potency. This effect was most evident for darumycin E (**6**), which lacks both O-methylations. Comparison with the singly demethylated derivatives darumycin C (**4**) and darumycin D (**5**) suggests that the loss of activity primarily arises from the presence of a free carboxyl group, rather than from demethylation of the C-4 hydroxyl group alone.

Given the close structural similarity between darumycins and gromomycins, together with their highly comparable activity profiles, it is likely that the darumycin sesterterpenes exert their antibacterial effects through interactions with the bacterial cell envelope.^38^ This interpretation is supported by the strong correlation between antibacterial activity and cytotoxicity observed across the compound series (Table 1), consistent with nonspecific interactions with membrane lipids rather than engagement of a defined protein target.

The proposed membrane-targeting mode of action also provides a rationale for the reduced activity of darumycin D (**5**) and darumycin E (**6**), as the introduction of a negatively charged carboxyl group is expected to disrupt hydrophobic interactions between the terpene core and lipid acyl chains. Conversely, derivatives lacking the carboxyl functionality, including darumycin F (**7**) and darumycin G (**8**), exhibit slightly enhanced antibacterial activity relative to darumycins A (**1**) and B (**2**), consistent with increased hydrophobicity and improved membrane association.

Collectively, the results presented here establish the darumycins as a distinct family of guanidine-containing sesterterpene antibiotics that expand the known chemical space of bacterial terpenoids. Genome mining-guided by gromomycin biosynthetic logic enabled the identification of a divergent pathway in *M. rubida*, while heterologous expression, intermediate trapping, and targeted gene deletions revealed a modular biosynthetic framework in which scaffold assembly and late-stage tailoring are partially decoupled. *In vitro* reconstitution and structural characterization of the tailoring methyltransferases uncovered complementary enzymatic strategies, ranging from strict site selectivity to substrate–promiscuous activity, which facilitate diversification of the terpene core. Together with the observed structure–activity relationships, these findings highlight how subtle variations in enzyme architecture and pathway organization can generate chemically and functionally distinct antibiotics from closely related biosynthetic blueprints.

## Conclusions

In this study, we report the genome mining–guided discovery of the darumycins, a new family of guanidine-containing sesterterpene antibiotics from *M. rubida* with potent activity against high-priority Gram-positive pathogens and mycobacteria. Identification of the minimal darumycin biosynthetic gene cluster and systematic gene deletion analysis enabled isolation of a series of structurally defined biosynthetic intermediates and derivatives, thereby substantially expanding the chemical space of this rare terpene subclass. Combined LC–MS and NMR analyses provided a detailed view of scaffold assembly and late-stage tailoring, establishing a modular biosynthetic framework for guanidine-containing sesterterpenes.

Biochemical reconstitution of the two pathway-encoded methyltransferases, DarG and DarM, revealed complementary but distinct catalytic strategies for scaffold diversification. DarM exhibits strict site selectivity for carboxyl methylation, whereas DarG displays broader substrate tolerance and relaxed positional selectivity, enabling multiple methylation outcomes from a shared cyclic intermediate. These functional differences are directly rationalized by structural analysis: the crystal structure of DarM uncovered a narrowly confined, SAM-dependent class I methyltransferase architecture that enforces a single productive substrate binding mode, in contrast to the more open and adaptable binding pocket predicted for DarG.

Beyond their immediate relevance to darumycin biosynthesis, these findings provide broader insight into the evolutionary diversification of tailoring enzymes in terpene pathways. Subtle architectural variations within a conserved methyltransferase fold appear sufficient to generate distinct specificity profiles, facilitating late-stage chemical diversification without altering core scaffold assembly. Together, this work establishes the darumycins as a chemically and biosynthetically distinct class of sesterterpene antibiotics and highlights tailoring enzyme evolution as a powerful driver for expanding natural product chemical space, with clear implications for genome mining, pathway engineering, and the discovery of new bioactive terpenoids.

## Supplementary Material



## Notes

The authors have cited additional references within the Supporting Information. Darumycins Supporting Information (word file) (PDF)
