# Supplementary material for: Discovery and Biosynthetic Characterization of Darumycins Expand the Chemical Space of Sesterterpene Antibiotics
Source: JACS Au. 2026 Aug 19;6(9):4944–55. doi: 10.1021/jacsau.6c00499 (PMC13625510; doi:10.1021/jacsau.6c00499)
Supplement: Supplementary file 1 [file au6c00499_si_001.pdf]

Supplementary Information

Discovery and Biosynthetic Characterization of Darumycins Expand the Chemical Space of Sesterterpene Antibiotics

Dmytro Bratiichuk[1], Dominik Kolling[2,3]*, Irma Redzic[1]*, Marc Stierhof[1,3,4], Franziska Fries[3,5], Josef Zapp[1,3], Rolf Müller[3,5], Maksym Myronovskyi[1], Jesko Koehnke[2#] and Andriy Luzhetskyy[1,3#]*

[1]Department of Pharmaceutical Biotechnology, Pharma Science Hub (PSH), Saarland University, Bld. C2.3, 66123 Saarbrücken, Germany.

[2]Institute of Food Chemistry, Leibniz University, 30167 Hannover, Germany.

[3]Helmholtz Institute for Pharmaceutical Research Saarland (HIPS), Helmholtz Centre for Infection Research (HZI), Saarland University, 66123 Saarbrücken, Germany.

[4]Fraunhofer Institute for Translational Medicine and Pharmacology (ITMP) Immunology, Infection und Pandemic Research (IIP), 80799 München, Germany

[5]German Center for Infection Research (DZIF), Partner Site Hannover-Braunschweig, 38124 Braunschweig, Germany.

*Authors contributed equally.

# Corresponding authors: Jesko Koehnke (jesko.koehnke@lci.uni-hannover.de), Andriy Luzhetskyy (a.luzhetskyy@mx.uni-saarland.de)

Table of Contents

Materials and Methods 6
Bacterial Strains, Plasmids, and Culture Conditions....6
Metabolite Extraction and Analysis 6
Isolation and Purification of Darumycins 7
Cosmid Library Construction....8
Heterologous Expression of Darumycin Gene Cluster and Systematic Gene Deletion....8
Genome-Guided Identification of Darumycin Cluster....8
Cloning, Recombinant Expression and Purification of DarG and DarM....9
Cloning, Recombinant Expression and Purification of DarM mutants for mechanistic studies....10
In vitro Enzyme Assay of DarG, DarM and DarM mutants 10
HPLC-MS analysis of DarG and DarM *in vitro* reactions....11
Crystallization of DarM and Structure Determination....11
Antibiotic Activity (Minimum Inhibitory Concentrations)....12
Cytotoxic Activity (IC50) 13
Maximum Tolerated Concentration 13
Structure elucidation 15
Nuclear Magnetic Resonance Spectroscopy 15
Isolated Compounds and Their Analytical Data 15
Mosher Analysis of Darumycin C....16
List of Supplementary Figures....17
Figure S1: A) Chemical structure of guanidine-containing terpene natural products....17
Figure S2: HPLC-MS chromatograms depicting the heterologous expression of the cosmid P05-E12 with darumycin cluster in *S. albus* Del14. 18
Figure S3: A) HPLC-MS chromatograms of the strains *S. lividans* DelYA9, *S. lividans* DelYA9 cos15AAmInt-5E12.....19
Figure S4: Structure of the darumycin A (1)....20
Figure S5: ^{1}H NMR spectrum (500 MHz) of darumycin A (1) in CD_3OH. 20
Figure S6: ^{13}C NMR spectrum (125 MHz) of darumycin A (1) in CD_3OH. 21
Figure S7: ^{1}H-^{1}H COSY spectrum (500 MHz) of darumycin A (1) in CD_3OH. 21
Figure S8: NOESY spectrum (500 MHz) of darumycin A (1) in CD_3OH. 22
Figure S9: Edited HSQC spectrum (500 MHz) of darumycin A (1) in CD_3OH....22
Figure S10: ^{13}C HMBC spectrum (500 MHz) of darumycin A (1) in CD_3OH....23
Figure S11: ^{15}N-HMBC spectrum (500 MHz) of darumycin A (1) in CD_3OH. 23
Figure S12: Structure of darumycin B (2)....24
Figure S13: ^{1}H-NMR spectrum (500 MHz) of darumycin B (2) in CD_3OD 24

Figure S14: ^{13}C-NMR spectrum (125 MHz) of darumycin B (2) in CD_3OD. 25
Figure S15: H-H COSY spectrum (500 MHz) of darumycin B (2) in CD_3OD 25
Figure S16: Edited HSQC spectrum (500 MHz) of darumycin B (2) in CD_3OD. 26
Figure S17: ^{13}C-HMBC spectrum (500 MHz) of darumycin B (2) in CD_3OD....................26
Figure S18: ^{15}N HMBC spectrum (500 MHz) of darumycin B (2) in CD_3OD....................27
Figure S19: Diagram of the DNA segment containing the darumycin biosynthetic gene cluster, depicted in grey 28
Figure S20A: HPLC-MS chromatograms of the strains *S. lividans* DelYA9, *S. lividans* DelYA9 cos15AAmInt-5E12, *S. lividans* DelYA9 cos15AAmInt-5E12_darA del. 29
Figure S20B: HPLC-MS chromatograms of the strains *S. lividans* DelYA9, *S. lividans* DelYA9 cos15AAmInt-5E12, *S. lividans* DelYA9 cos15AAmInt-5E12_darI del. 29
Figure S21: Structure of pentaprenylguanidine (3). 30
Figure S22: ^{1}H-NMR spectrum (500 MHz) of pentaprenylguanidine (3) in CD_3OD. 31
Figure S23: ^{13}C-NMR spectrum (125 MHz) of pentaprenylguanidine (3) in CD_3OD 31
Figure S24: H-H COSY spectrum (500 MHz) of pentaprenylguanidine (3) in CD_3OD 32
Figure S25: Edited HSQC spectrum (500 MHz) of pentaprenylguanidine (3) in CD_3OD. ..32
Figure S26: HMBC spectrum (500 MHz) of pentaprenylguanidine (3) in CD_3OD. 33
Figure S27: HPLC-MS chromatograms of the strains *S. albus* Del14, *S. albus* Del14 cos15AAmInt-5E12, *S. albus* Del14 cos15AAmInt-5E12_darG del...................................34
Figure S28: Structure of darumycin C (4)...34
Figure S29: ^{1}H-NMR spectrum (500 MHz) of darumycin C (4) in CD_3OD 35
Figure S30: ^{13}C-NMR spectrum (125 MHz) of darumycin C (4) in CD_3OD. 35
Figure S31: H-H COSY spectrum (500 MHz) of darumycin C (4) in CD_3OD 36
Figure S32: Edited HSQC spectrum (500 MHz) of darumycin C (4) in CD_3OD. 36
Figure S33: HMBC spectrum (500 MHz) of darumycin C (4) in CD_3OD. 37
Figure S34: HPLC-MS chromatograms of the strains *S. albus* Del14, *S. albus* Del14 cos15AAmInt-5E12, *S. albus* Del14 cos15AAmInt-5E12_darM del. 38
Figure S35: Structure of darumycin D (5). 38
Figure S36: ^{1}H-NMR spectrum (500 MHz) of darumycin D (5) in CD_3OD 39
Figure S37: ^{13}C-NMR spectrum (125 MHz) of darumycin D (5) in CD_3OD. 39
Figure S38: H-H COSY spectrum (500 MHz) of darumycin D (5) in CD_3OD. 40
Figure S39: Edited HSQC spectrum (500 MHz) of darumycin D (5) in CD_3OD................40
Figure S40: HMBC spectrum (500 MHz) of darumycin D (5) in CD_3OD. 41
Figure S41: Structure of the darumycin C (4) Mosher conjugate. 42
Figure S42: HPLC chromatogram and mass spectra (*m/z*) of the darumycin C (4) Mosher conjugates 42
Figure S43: ^{1}H NMR spectrum (500MHz) of darumycin C (4) (*R*)-MTPA ester in $CDCl_3$.43

Figure S44: Edited HSQC spectrum (500 MHz) of darumycin C (4) (*R*)-MTPA ester in $CDCl_3$. 43
Figure S45: ^{1}H NMR spectrum (500MHz) of darumycin C (4) (*S*)-MTPA ester in $CDCl_3$. 44
Figure S46: Edited HSQC spectrum (500 MHz) of darumycin C (4) (S)-MTPA ester in $CDCl_3$ 44
Figure S47: HPLC-MS chromatograms of the strains *S. albus* Del14, *S. albus* Del14 cos15AAmInt-5E12, *S. albus* Del14 cos15AAmInt-5E12_darG/darM del. 45
Figure S48: Structure of darumycin E (6). 45
Figure S49: ^{1}H-NMR spectrum (500 MHz) of darumycin E (6) in CD_3OD........................46
Figure S50: ^{13}C-NMR spectrum (125 MHz) of darumycin E (6) in CD_3OD. 46
Figure S51: H-H COSY spectrum (500 MHz) of darumycin E (6) in CD_3OD. 47
Figure S52: Edited HSQC spectrum (500 MHz) of darumycin E (6) in CD_3OD. 47
Figure S53: HMBC spectrum (500 MHz) of darumycin E (6) in CD_3OD 48
Figure S54: HPLC-MS chromatograms of the strains *S. albus* Del14, *S. albus* Del14 cos15AAmInt-5E12, *S. albus* Del14 cos15AAmInt-5E12_darC del. 49
Figure S55: Structure of darumycin F (7). 49
Figure S56: ^{1}H-NMR spectrum (500 MHz) of darumycin F (7) in CD_3OD. 50
Figure S57: ^{13}C-NMR spectrum (125 MHz) of darumycin F (7) in CD_3OD. 50
Figure S58: H-H COSY spectrum (500 MHz) of darumycin F (7) in CD_3OD. 51
Figure S59: Edited HSQC spectrum (500 MHz) of darumycin F (7) in CD_3OD. 51
Figure S60: HMBC spectrum (500 MHz) of darumycin F (7) in CD_3OD. 52
Figure S61: HPLC-MS chromatograms of the strains *S. albus* Del14, *S. albus* Del14 cos15AAmInt-5E12, *S. albus* Del14 cos15AAmInt-5E12_darC/darG del. 53
Figure S62: Structure of darumycin G (8). 53
Figure S63: ^{1}H-NMR spectrum (500 MHz) of darumycin G (8) in CD_3OD. 54
Figure S64: ^{13}C-NMR spectrum (125 MHz) of darumycin G (8) in CD_3OD. 54
Figure S65: H-H COSY spectrum (500 MHz) of darumycin G (8) in CD_3OD. 55
Figure S66: Edited HSQC spectrum (500 MHz) of darumycin G (8) in CD_3OD................55
Figure S67: HMBC spectrum (500 MHz) of darumycin G (8) in CD_3OD. 56
Figure S68: HPLC-MS chromatograms of the strains *S. albus* Del14, *S. albus* Del14 cos15AAmInt-5E12, *S. albus* Del14 cos15AAmInt-5E12_darK del. 57
Figure S69: LC-MS analysis of DarG biochemical reactions using darumycin G (8) as substrates. 58
Figure S70: LC-MS analysis of DarG biochemical reactions using darumycin E (6) as substrates. 59
Figure S71: LC-MS analysis of DarM biochemical reactions using darumycin G (8) and darumycin E (6) as substrates...60
Figure S72: LC-MS analysis of DarG biochemical reactions using darumycin C(4) and darumycin D (5) as substrates. 61

Figure S73: LC-MS analysis of DarM biochemical reactions using darumycin C (4) and darumycin D (5) as substrates. 62
Figure S74: Crystal structure of DarM in complex with SAH. 63
Figure S75: Detailed view on DarM substrate pocket and propsed mechanism for SAM-dependent enzymatic methylation of darumycin E. 64
Figure S75: Model of DarM-SAH crystal structure with darumycin D and biochemical evaluation of DarM mutants. 65
Figure S77: Superposition of DarG AlphaFold3 model and AtM involved in rebeccamycin biosynthesis. 66
Figure S78: Natural products whose biosynthesis involves enzymatic methylation via SAM-dependent methyltransferases. 67
List of Supplementary Tables 68
Table S1: NMR data of Darumycin A (1) in CD_3OH. 68
Table S2: NMR data of Darumycin B (2) in CD_3OD. 69
Table S3: Proposed function of genes in darumycin and gromomycin BGCs. 70
Table S4: NMR data of Pentaprenylguanidine (3) in CD_3OD. 71
Table S5: NMR data of Darumycin C (4) in CD_3OD. 72
Table S6: NMR data of Darumycin D (5) in CH_3OD. 73
Table S7: ^{1}H NMR ($CDCl_3$) data of Darumycin C (*S*)-MTPA ester and Darumycin F (*R*)-MTPA ester) and their $\Delta\delta_{S-R}$ values. 74
Table S8: NMR data of Darumycin E (6) in CD_3OD. 75
Table S9: NMR data of Darumycin F (7) in CH_3OD. 76
Table S10: NMR data of Darumycin G (8) in CD_3OD. 77
Table S11: Summary of DALI search using the structure of DarM in complex with SAH as input. 78
Table S12: Summary of DALI search using an AlphaFold 3 model of DarG as input. 79
Table S13: Methyltransferases embedded in *gro* like BGCs. 80
Table S15: Bacterial strains used in this work. 82
Table S16: Plasmids used in this work. 83
Table S17: Primers used in this work. 84
Table S18: DarG and DarM nucleotide and amino acid sequence. 86
Table S19: Errors associated with exact mass data reported in this study. 87
Table S20: Data collection and refinement statistics 88
References 89

Materials and Methods

Bacterial Strains, Plasmids, and Culture Conditions

All strains, plasmids and primers used in this work are listed in Supplementary **Tables S15-S17**. The strain *Micromonospora rubida* JCM 32386 was obtained from the Japan Collection of Microorganisms, RIKEN BRC. ISP-1 (656) medium (5 g L^{-1} tryptone, 3 g L^{-1} yeast extract, 2 g L^{-1} $MgSO_4$*7H_2O, pH to 7.0-7.2) or Sucrose-Bennets agar (104) medium (1 g L^{-1} yeast extract, 1 g L^{-1} beef extract, 2 g L^{-1} N-Z amine (type A), 10 g L^{-1} sucrose, 15 g L^{-1} agar, pH 7.3) were used for the *Micromonospora* strain cultivation. *S. albus* Del14 and *S. lividans* DelYA9 were used as hosts for heterologous expression of the darumycin biosynthetic gene cluster. *E. coli* strains were grown in Luria-Bertani (LB) broth (Sigma-Aldrich, St. Louis, MO, USA). *Streptomycetes* strains were grown on MS agar medium (Soy flour 20 g, Mannitol 20 g, tap water 1 L, pH 7.2 prior autoclaving) and in liquid tryptic soy broth medium (TSB; Sigma-Aldrich, St. Louis, MO, USA). If necessary, the following antibiotics were added: apramycin (50 µg mL^{-1}), kanamycin (50 µg mL^{-1}) and nalidixic acid (50 µg mL^{-1}) (Sigma-Aldrich, St. Louis, MO, USA; Roth, Karlsruhe, Germany). For conjugation, the *Streptomycetes* strains were grown on MS agar for sporulation. For compounds production, S*treptomyces* strains were grown in liquid DNPM medium (40 g L^{-1} dextrin, 7.5 g L^{-1} soytone, 5 g L^{-1} baking yeast, and 21 g L^{-1} MOPS, pH 6.8), SG (20 g L^{-1} glucose, 10 g L^{-1} soy peptone, and 2 g L^{-1} $CaCO_3$, pH 7.2), GYM (4 g L^{-1} glucose, 4 g L^{-1} yeast extract, 10 g L^{-1} malt extract and 2 g L^{-1} $CaCO_3$, pH 7.2) and MM (minimal medium) (0.5 g L^{-1} L-Aspartic acid, 0.5 g L^{-1} K_2HPO_4, 0.2 g L^{-1} $MgSO_4$*7H_2O, 0.2 g L^{-1} $FeSO_4$*7H_2O, 10 g L^{-1} glucose).

Metabolite Extraction and Analysis

S. albus Del14 and *S. lividans* DelYA9 carrying the cluster were grown in 50 mL of TSB medium at 28 °C for 2 days to generate the pre-culture. For the main cultures, 100 mL of DNPM, SG, GYM or MM medium was inoculated with 1 mL of the pre-culture. In the fermentation experiments aimed at identifying the most productive medium for darumycin synthesis, *S. albus* Del14, carrying no cluster, and *S. albus* Del14 cos5E12, carrying the intact darumycin cluster, were used as controls. After six days of incubation at 28 °C, darumycin derivatives were extracted from the culture supernatant using ethyl acetate, followed by solvent evaporation. Chromatographic analysis was performed by injecting 1 µL of the extracted sample into a Dionex Ultimate 3000 UPLC system (Thermo Fisher Scientific, Waltham, MA, USA) with a 10-cm ACQUITY UPLC® BEH C18 column (1.7 µm, Waters, Milford, MA, USA). Separation followed a linear gradient from 5% to 95% acetonitrile with 0.1% formic

acid (FA) against 0.1% FA in water over 25 minutes, at a 0.6 mL min^{-1} flow rate and 45 °C column temperature. Mass spectrometric analysis was conducted on an amaZon speed mass spectrometer or a maXis high-resolution LC-QTOF system (Bruker, USA). Bruker Compass Data Analysis software (version 6.1, Bruker, Billerica, MA, USA) was used for data collection and processing.

Isolation and Purification of Darumycins

Darumycins were isolated after strain cultivation in 10 L of DNPM, SG or GYM medium (depending on the derivative) at 28 °C for six days under continuous agitation at 180 rpm. Following fermentation, the compounds were extracted from the culture supernatant using ethyl acetate. The resulting extracts were dried through solvent evaporation, redissolved in methanol, and subjected to purification via size-exclusion chromatography on a Sephadex LH-20 column (Sigma-Aldrich, Louis, MO, USA) with methanol as the mobile phase. Fraction collection was performed at 10-minute intervals at a flow rate of 0.8 mL min^{-1}. The fractions containing the desired darumycin derivative were pooled together, concentrated and dissolved in methanol, followed by Reversed-Phase High Performance Liquid Chromatography (RP-HPLC) (Waters AutoPurification™ System) separation on preparative C18 column Nucleodur HTec, 5 µm, 250 mm x 21 mm (Macherey-Nagel, Germany) using a water solution containing 0.1% (v/v) formic acid (solvent A), and an acetonitrile solution containing 0.1% (v/v) formic acid (solvent B) as a mobile phase. For compounds separation by RP-HPLC, the following gradient at a flow rate of 20 mL min^{-1} was used: 0 min-5% B, 0.5 min-5%, 12 min-65% B, 25 min-65%B, 27 min-95% B, 28 min-95% B, 29 min-5% B, 31 min-5% B. The fractions were collected using MS detector (Waters™ SQ Detector 2). The darumycin-containing fractions were pooled, evaporated, and subjected to the final purification step. The final purification step involved RP-HPLC using Thermo Scientific Dionex UltiMate 3000 system equipped with a semipreparative Synergy™ 4 µm Fusion-RP C18 column (80 Å, 250 × 10 mm; Phenomenex, Torrance, CA, USA). Separation was achieved with a mobile phase consisting of water with 0.1% formic acid (solvent A) and acetonitrile with 0.1% formic acid (solvent B). Separation was performed using the following gradient at a flow rate of 4 mL/min: 0 min-5% B, 0.5 min-5% B, 10 min-65% B, 12 min-65% B, 15 min-95% B, 17 min-95% B, and 18 min-5% B. Fractions containing the purified compound were collected, pooled, and evaporated.

Cosmid Library Construction

A cosmid library of *M. rubida* JCM 32386 was prepared using the EpiCentre CopyControl™ Fosmid Library Production Kit in pCos15A_AmInt vector by adapting the protocol from Lucigen. A library of 30−40 kb fragments was constructed according to the manufacturer's protocol using genomic DNA isolated with NucleoSpin Microbial DNA Mini kit for DNA from microorganisms (MACHEREY-NAGEL GmbH & Co. KG, Germany). The purified genomic DNA fragments were ligated into linearized pCos15A_AmInt vectors. The ligation reactions were packaged into λ phage for *E.coli* EPI300 infection. The packaged library was plated on LB agar plates containing 50 µg mL^{-1} apramycin, and grown overnight at 37 °C. Approximately 1700 single colonies were picked and inoculated into individual wells of 96-well plates. This arrayed cosmid library was stored with 20% glycerol and kept at −80 °C.

Heterologous Expression of Darumycin Gene Cluster and Systematic Gene Deletion

The constructs used for heterologous expression in this study were introduced into *S. albus* Del14 and *S. lividans* DelYA9 by conjugation using donor strain *E. coli* ET12657/pUB307 on MS plates. After incubating at 29 °C for 15 hours, exconjugants were selected with 50 µg mL^{-1} apramycin and and 50 µg mL^{-1} nalidixic acid. Single colonies from this plate were patched onto MS plates with 50 µg mL^{-1} apramycin.

Genome-Guided Identification of Darumycin Cluster

Three genes involved in the biosynthesis of gromomycins were used for genome-wide quantitative screening of novel derivatives: *groD*, *groF* and *groH*. The nucleotide sequences of these genes were used as probes in the NCBI protein BLAST database to identify new gromomycin-like clusters. The strains with all three of these genes identified in the genome were selected. In this way, many actinomycetes strains were identified, including *M. rubida* strain. The new gromomycin-like cluster, later renamed to darumycin cluster, was isolated from the genome of the *M. rubida* strain through the cosmid library construction. Cosmid P05-E12 containing whole darumycin cluster was identified by end sequencing along with PCR with two pairs of primers designed to amplify regions to the left or right side of the cluster. The P05-E12 cosmid was introduced into *S. albus* Del14 and *S. lividans* DelYA9 strains. The ability of heterologous strains to produce new derivatives was determined by HPLC.

Cloning, Recombinant Expression and Purification of DarG and DarM

The full-length synthetic codon-optimized genes for DarG (accession number: WP_161689877.1) and DarM (accession number: WP_161689883.1) from *M. rubida* were ordered from GenScript pre-cloned into the expression vector pET-29a(+) (Table S18). A tobacco etch virus (TEV) protease site (ENLYFQG) was inserted upstream of both genes to enable cleavage of the N-terminal His_6-tag during protein purification. *E. coli* Lemo21 (DE3) was used for recombinant protein expression and the two plasmids were transformed using a standard heat shock protocol.

A single colony of *E. coli* Lemo21 harboring the plasmid pET-29a(+)-TEV-DarG or pET-29a(+)-TEV-DarM was used to inoculate a preculture in LB medium supplemented with 25 µg mL^{-1} chloramphenicol and 50 µg mL^{-1} kanamycin and incubated overnight at 37 °C and 180 rpm. At the next day 1 L LB main culture in 2 L buffled flasks was inoculated with 15 mL overnight culture and incubated at 37 °C and 180 rpm until an OD_{600} of 0.8 was reached. Protein expression was induced with 1 mM IPTG and the culture was incubated at 16 °C and 180 rpm for 16 h. The cells were harvested by centrifugation at 12 800 g and 4 °C for 15 min, and the pellet was flash-frozen and stored at - 80 °C until further use. Both proteins DarG and DarM were purified using the same following conditions. The cell pellet (100 mL lysis buffer per every 25 g wet cell mass) was resuspended in buffer A (50 mM Tris pH 8.0, 500 mM NaCl, 10% glyercol, 20 mM imidazole and 3 mM BME) supplemented with DNAse (0.4 mg g^{-1} wet cell pellet, Sigma) and cOmplete EDTA-free protease inhibitor tablets (Roche). The cells were lysed by passing them twice through a cell disruptor (24.5 kPSI, 4 °C, Constant Systems) and the cell debris was removed by centrifugation at 40 000 g and 4 °C for 30 min. The clarified lysate was subsequently loaded using an ÄKTA go onto a HisTrap HP 5 mL column (Cytiva) pre-equilibrated with buffer A using a flow rate of 5 mL min^{-1} and at 4 °C. After a column wash with 20 CV buffer A, elution was performed using buffer B (50 mM Tris pH 8.0, 500 mM NaCl, 10% glyercol, 300 mM imidazole and 3 mM BME), and protein containing fractions were pooled together. For subsequent removal of the His-tag, the pooled fractions were desalted into buffer C (50 mM Tris pH 8.0, 500 mM NaCl, 10% glyercol, 20 mM imidazole and 3 mM BME) by passing over a HiPrep 26/10 desalting column (Cytiva) at a flow rate of 10 mL min^{-1} connected to a ÄKTA pure, TEV protease was added in a 1:10 mass ratio and incubated overnight at 4 °C. Undigested protein and His-tagged TEV protease were removed by another Ni affinity chromatography. To further increase protein purity, anion exchange chromatography was performed, whereby DarG- / DarM-containing fractions were buffer-exchanged into buffer D (25 mM Tris pH 8.0, 30 mM NaCl, 5% glycerol and 1 mM DTT) using a HiPrep26/10 column. The desalted sample was loaded onto a HiTrap Q 5 mL column (Cytiva) previously

equilibrated with buffer D and both proteins were eluted using a linear gradient with buffer E (25 mM Tris pH 8.0, 1 M NaCl, 5% glycerol and 1 mM DTT). DarG or DarM containing fractions were pooled and the proteins were finally purified by size-exclusion chromatography on a Superdex S75 16/600 column pre-equilibrated with storage buffer F (10 mM Tris pH 8.0, 100 mM NaCl) at a flow rate of 1 mL min^{-1}. The resulting peak was collected and fractions containing purified DarG or DarM were concentrated to 20 mg mL^{-1} and 30 mg mL^{-1} using a 10 kDa cut-off filter (Thermo Fisher Scientific) and flash-frozen in liquid nitrogen. The protein concentration was measured by specific absorption at A_{280} on a Nanodrop2000 (Thermo Fisher Scientific) and protein purity was assayed using sodium dodecyl-sulfate polyacrylamide gel electrophoresis.

Cloning, Recombinant Expression and Purification of DarM mutants for mechanistic studies

Site-directed mutagenesis within the darM gene cloned into the expression palsmid pET-29a(+)-TEV was performed via PCR using the Pusion™ High-Fidelity DNA polyermase from NEB and primers listed in table S17. The linearized plasmid was ligated using the KLD mix form NEB and transformed into chemically competent *E. coli* DH5α using a standard heat-shock protocol. Clones were selected, plasmid DNA was purified and successful incorporation of single point mutations was confirmed by Sanger sequencing. The constructs were transformed into *E. coli* Lemo21 (DE3) and DarM mutants as well as DarM wild-type were expressed under the same conditions as aforementioned. Cell pellets from 100 mL main culture were resuspendend in 20 mL lysis buffer (50 mM Tris pH 8.0, 500 mM NaCl, 10% glyercol, 20 mM imidazole and 3 mM BME) and cell disruption was performed by sonication (5 min, 15 sec pulse on, 30 sec off, 75% amplitude). The lysate was clarified by centrifugation and proteins were purified by gravity flow at 4 °C using HisPur™ Ni-NTA resin (Thermo Fisher Scientific). DarM mutant and wild-type containing fractions were pooled and desalted into storage buffer 10 mM Tris pH 8.0, 100 mM NaCl using PD-10 columns (Cytiva) as described in the manufacturers protocol. Protein concentrations were determined as mentioned above.

***In vitro* Enzyme Assay of DarG, DarM and DarM mutants**

Biochemical reactions were performed in 10 mM Tris pH 8.0 and 100 mM NaCl in a reaction volume of 50 µL. For DarG reactions a mixture of 100 µM compound (darumycin G (**8**), darumycin E (**6**), darumycin D (**5**) and darumycin C (**4**)), 10 µM purified enzyme, and1 mM freshly prepared SAM were incubated for 2 h at 37 °C. For DarM reactions a mixture of 100 µM compound (darumycin G (**8**), darumycin E (**6**), darumycin D (**5**) and darumycin C (**4**)), 10 µM purified enzyme, and 1 mM freshly prepared SAM were incubated for 2 h at 37 °C.

Biochemical reactions using DarM mutants were performed in triplicates using 100 µM darumycin D (**5**) as substrate, 10 µM purified DarM variant and 1 mM SAM. The reaction mixture was incubated for 2 h at 37 °C. Reactions were stopped by by adding 50% acetonitrile and the mixture was flash-frozen in liquid nitrogen. The samples were stored at -80 °C at least 2 h prior to LC-MS analysis.

HPLC-MS analysis of DarG and DarM *in vitro* reactions

Samples obtained from *in vitro* DarG and DarM reactions were analysed using UHPLC-MS and the retention time as well as the exact mass was determined. Separation was achived on a Vanquish™ Flex UHPLC system (Thermo Fisher Scientifc) equipped with a binary pump module, a diode array detector and a split autosampler module. 1 µL sample was loaded onto a Waters Acquity® BEH C18 column (100 x 2.1 mm, 1.7 µm) connected to a Waters VanGuard BEH C18 pre-column at a flow rate of 0.6 mL min^{-1} and a linear gradient with 5-95% ACN + 0.1% FA over 18 min was applied for separation. Exact masses were determined using a Bruker maXis TOF coupled to the UHPLC. The following MS settings were applied: ESI ionization mode, capillary voltage 4000 V, end plate off-set -500 V, nebulizer gas pressure 1.1 bar, dry gas flow rate 5.0 L min^{-1}, dry gas temperature 200 °C, mass scan range 200-2000 *m/z* and sodium formate cluster was used for calibration. LC-MS results were analyzed using Bruker Compass DataAnalysis Version 6.1. Errors associated with exact mass data are reported in **table S19**.

Crystallization of DarM and Structure Determination

Crystallization screening experiments for DarM in complex with SAH were performed at a protein concentration of 10 mg mL^{-1} (stored in gel filtration buffer 10 mM Tris pH 8.0 and 100 mM NaCl) and using commercially available screens form Nextal. The screens were pipetted into sitting-drop SwissSCI plates using a Gryphron crystallization robot (Art Robbins Instruments) and incubated at 293.15 K. Diffraction quality crystals of DarM appeared within the next few days in a condition with a well solution of 0.2 M sodium chloride, 0.1 sodium acetate pH 4.5 and 1.26 M ammonium sulfate. The crystals were cryoprotected using the well solution supplemented with 32% glycerol, mounted into cryo-loops (Hampton Research) and immediately flash-frozen in liquid nitrogen. A dataset for DarM in complex with SAH was collected at ESRF using the beamlines ID-30B at 100 K. Data were processed using POINTLESS and AIMLESS as well as ctruncate implemented in ccp4 (CCP4Interface 8.0.019).[1-3] The structure was solved in Phenix (phenix.phase) by molecular replacement using

a model generated with AlphaFold3, following by further processing using Phenix.autobild.[4-6] The resulting structure was manually rebuild in COOT (Version 0.9.8.93 under Xquartz 11 version 2.8.5) and refined several times using Phenix.refine.[7-8] Final PDB coordinates were analyzed using MolProbity[9] implemented in Phenix before deposition into the protein database. Data collection and refinement statistics are summarized in **table S20**. The coordinates were deposited in the Protein Data Bank (PDB) under entry code 28jb and were further used to search for structural homologs via the DALI server. The experimental DarM-SAH complex structure served as target for docking of substrate darumycin D using AutoDock Vina 1.2.0.[10] All figures shown in this study were rendered in PyMOL (The PyMOL Molecular Graphics System Version 3.1.6.1, Schrödinger, LLC).

Antibiotic Activity (Minimum Inhibitory Concentrations)

Darumycin stock solutions were prepared in dimethyl sulfoxide (DMSO). All microorganisms used in this study were obtained from the German Collection of Microorganisms and Cell Cultures (DSMZ), the American Type Culture Collection (ATCC), the Coli Genetic Stock Center, or were part of our internal strain collection. *Staphylococcus aureus* strains Newman, N315, Mu50 and Cowan 1 were obtained from M. Bischoff, Saarland University Hospital, Homburg. *S. aureus* wild type and Dap^R HG001 were provided by T. Schneider, University of Bonn. *E. coli* WO153 was provided by K. Lewis, Northeastern University, Boston, USA. Minimum inhibitory concentrations (MICs) were determined using the broth microdilution method according to EUCAST guidelines (ISO 20776-1:2019). In short, serial two-fold dilutions of darumycins (0.03125 to 64 µg mL^{-1}) were prepared in 75 µL of cation-adjusted Mueller-Hinton broth (MHB2) in sterile 96-well plates. Equal volume of the bacterial suspension was added, and the plates were incubated at 37 °C for 18 h. For *Streptococcus pneumoniae*, MHF broth (MHB2 supplemented with 5% lysed horse blood and 20 mg L^{-1} β-NAD) was used and plates were incubated at 37 °C with 5% CO_2.

The MIC was defined as the lowest concentration of the antibiotic causing complete inhibition of visible growth of the microorganism. The same method was used for testing *Mycobacterium smegmatis*, but with the use of Middlebrook 7H9 complete medium supplemented with oleic acid, albumin, dextrose and catalase (OADC, 10%). *M. smegmatis* plates were incubated for 48 h at 37 °C. For assessing activity against *Mycobacterium tuberculosis,* an adapted resazurin microtitre assay (REMA) was performed. In short, *M. tuberculosis* single cells were prepared and added to compound dilutions in M7H9. Plates were incubated for 6 d at 37 °C, followed by addition of 50 µL of resazurin and incubation for another day at 37 °C. The MIC was determined

visually and additionally confirmed by measuring fluorescence (excitation at 530 nm, emission at 590 nm).

Cytotoxic Activity (IC50)

HepG2 cells (human hepatoblastoma cell line; ACC 180) and CHO-K1 (chinese hamster ovary cells; ACC 110) were obtained from the German Collection of Microorganisms and Cell Cultures (DSMZ) and cultured under the conditions recommended by the depositor. Cells were propagated in Roswell Park Memorial Institute (RPMI) 1640 medium and Ham's F12 medium, respectively, supplemented with 10% fetal bovine serum (FBS), and seeded at 6×10^3 cells per well of 96-well plates in 120 µL of complete medium. After 2 h of equilibration (37 °C, 5% CO_2), the cells were treated with a serial dilution of darumycins. Darumycins, doxorubicin as reference, as well as the solvent control (DMSO) were tested as duplicates in two independent experiments. After 5 d of incubation (37 °C, 5% CO_2), a total of 20 µL of 5 mg mL^{-1} MTT (thiazolyl blue tetrazolium bromide) in phosphate-buffered saline (PBS) were added to each well and the cells were further incubated for 2 h at 37 °C before the supernatant was discarded. Subsequently, the cells were washed with 100 µL of PBS and treated with 100 µl of 2-propanol/10 N HCl (250:1) to dissolve formazan granules. Cell viability was measured as a percentage relative to the respective solvent control by measuring the absorbance at 570 nm using a microplate reader (Tecan Infinite M200Pro). GraphPad Prism (version 10.0.3, GraphPad, Boston, MA, USA) was used for sigmoidal curve fitting to determine the IC_{50} values.

Maximum Tolerated Concentration

Husbandry of adult zebrafish was performed according to internal guidelines set out in the German Animal Welfare Act (§11 Abs. 1 TierSchG). Experiments were carried out with wild type AB (obtained from the European Zebrafish Resource Center at Karlsruhe Institute of Technology) embryos within the first 120 hours post fertilization (hpf) as these early life stages are not considered as animal experiments according to the EU Directive 2010/63/EU. Embryos were maintained in fresh 0.3x Danieau's (17.4 mM NaCl, 0.21 mM KCl, 0.12 mM $MgSO_4$, 0.18 mM $Ca(NO_3)_2$, 1.5 mM HEPES, 1.2 µM methylene blue, pH 7.1-7.3) at 28 °C. At a maximum of 120 hpf, embryos were euthanized by submersion in ice water for at least 12 h. For evaluation of the maximum tolerated concentration (MTC), embryos were dechorionated at 30 hpf using 1 mg mL^{-1} pronase and placed in a flat-bottom 96-well plate with one embryo per well. Excess medium was removed and 150 µL of darumycin dilutions (in 0.3x Danieau's, maximum of 1% DMSO) and of the solvent control (1% DMSO in 0.3x Danieau's) were added.

Ten embryos were used per condition. Exposed embryos were maintained at 28 °C until 120 hpf and they were monitored daily under a stereo microscope (Stemi 508, Zeiss) in order to record survival as well as anomalies, pigmentation, heartbeat and locomotor responses. An embryo was considered dead when no heartbeat could be observed. The maximum tolerated concentration (MTC) was defined as the highest concentration of the antibiotic with more than 90% survival of zebrafish embryos.

Structure elucidation

Nuclear Magnetic Resonance Spectroscopy

The chemical structures of all the compounds were determined *via* multidimensional NMR analysis. ^{1}H-NMR, ^{13}C-NMR, and 2D spectra were recorded at 500 MHz (^{1}H)/ 125 MHz (^{13}C), conducted in the Bruker Avance Neo 500 MHz, equipped with a Prodigy Cryo-probe. Samples were dissolved in methanol-d_4 or methanol-d_3. Chemical shifts are reported in ppm relative to tetramethylsilane; the solvent was used as the internal standard. Coupling constants are reported in Hertz (Hz). Multiplicity is reported with the usual abbreviations (s: singlet, br s: broad singlet, d: doublet, dd: doublet of doublets, ddd: doublet of doublet of doublets, t: triplet, dt: doublet of triplets, q: quartet, p: pentet, dp: doublet of pentets, m: multiplet).

Isolated Compounds and Their Analytical Data

Darumycin A (**1**) (*3S, 4S, 6S, 7S, 10R, 11S*): white powder, $[\alpha]_D^{20}$ +26° (c 0.37, MeOH). For NMR see Table S1 (500 MHz, CD_3OH), HRESIMS *m/z* 470.34 $[M+H]^+$, (calcd. for $C_{28}H_{44}N_3O_3$, 470.3383).

Darumycin B (**2**) (*3S, 4S, 6S, 7S, 10R, 11S*): white powder, $[\alpha]_D^{20}$ +57° (c 0.23, MeOH). For NMR see Table S2 (500 MHz, CD_3OD), HRESIMS *m/z* 468.32 $[M+H]^+$, (calcd. for $C_{28}H_{42}N_3O_3$, 468.32262).

Pentaprenylguanidine (**3**): white powder, for NMR see Table S4 (500 MHz, CD_3OD), HRESIMS *m/z* 400.37 $[M+H]^+$, (calcd. for $C_{26}H_{46}N_3$, 400.3692).

Darumycin C (**4**) (*3S, 4S, 6S, 7S, 10R, 11S*): white powder, $[\alpha]_D^{20}$ -23° (c 0.20, MeOH). For NMR see Table S5 (500 MHz, CD_3OD), HRESIMS *m/z* 456.32 $[M+H]^+$, (calcd. for $C_{27}H_{42}N_3O_3$, 456.3226).

Darumycin D (**5**) (*3S, 4S, 6S, 7S, 10R 11S*): white powder, $[\alpha]_D^{20}$ -19° (c 0.50, MeOD). For NMR see Table S6 (500 MHz, CD_3OD), HRESIMS *m/z* 456.32 $[M+H]^+$, (calcd. for $C_{27}H_{42}N_3O_3$, 456.3226).

Darumycin E (**6**) (*3S, 4S, 6S, 7S, 10R, 11S*): white powder, $[\alpha]_D^{20}$ -18° (c 0.24, MeOH). For NMR see Table S8 (500 MHz, CD_3OD), HRESIMS *m/z* 442.31 $[M+H]^+$, (calcd. for $C_{26}H_{40}N_3O_3$, 442.3070).

Darumycin F (**7**) (*3S, 4S, 6S, 7S, 10R, 11S*): white powder, $[\alpha]_D^{20}$ -60° (c 0.70, MeOH). For NMR see Table S9 (500 MHz, CD_3OD), HRESIMS *m/z* 426.35 $[M+H]^+$, (calcd. for $C_{27}H_{44}N_3O$, 426.3484).

Darumycin G (**8**) (*3S, 4S, 6S, 7S, 10R, 11S*): white powder, $[\alpha]_D^{20}$ -50° (c 0.22, MeOH). For NMR see Table S10 (500 MHz, CD_3OD), HRESIMS *m/z* 412.33 $[M+H]^+$, (calcd. for $C_{26}H_{42}N_3O$, 412.3328).

Mosher Analysis of Darumycin C

The absolute configuration was determined according to Mosher et al.[11] In brief, two glass vials with 1 mg dried darumycin C in 150 µL dry $CDCl_3$, 7 µL dry pyridine and 7 µL R-MTPA-Cl or S-MTPA-Cl, respectively, were prepared. The vials were flushed with nitrogen, closed airtight and were incubated with occasional shaking at room temperature while the reaction progress was monitored by LC-MS analysis by a Bruker Amazon Ion trap. Even after one day, 10% to 25% of unused darumycin C were still detectable. However, the desired Mosher products with *m/z* =672.41 was detected in higher abundance in both approaches. Subsequently, the reactions were stopped, and the samples were dried and purified by HPLC (Thermo Scientific Dionex UltiMate 3000 system equipped with a Phenomenex Synergi 4 µm C18 column 250*10 mm and a 20 min gradient of 50-65% acetonitrile/water containing 0.1% formic acid). This resulted in 0.6 mg darumycin C (*R*)-MTPA ester and 0.7 mg darumycin C (*S*)-MTPA ester (**Figure S41**). The purified samples were dissolved in $CDCl_3$ and NMR spectroscopy was performed.

According to the rules of Mosher analysis, protons H-1 and H-21 (group 1) and the remaining protons (group 2) should be divided into two groups based on the sign of the ΔδS-R values, whereby the type of sign should allow conclusions about the stereochemistry at C-4. (**Figure S42**) Unfortunately, the analysis of the ΔδS-R values did not lead to the desired result. While negative values were found for group 2, the values for group 1 were also negative, which is actually incompatible with the rules of Mosher analysis. MTPA derivatives not always fully meet the specified criteria, which can lead to a loss of confidence in the assignement.[12] Nevertheless, we attribute the strikingly high negative values for H-5a and H-5b, which are spatially very close to the MTPA residue, to an *S*-configuration at C-4, as was found in the closely related gromomycins.[13]

List of Supplementary Figures

A)

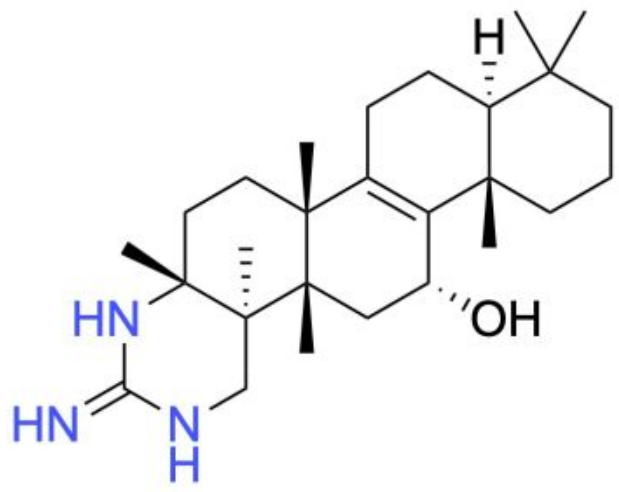

Cybastacine A

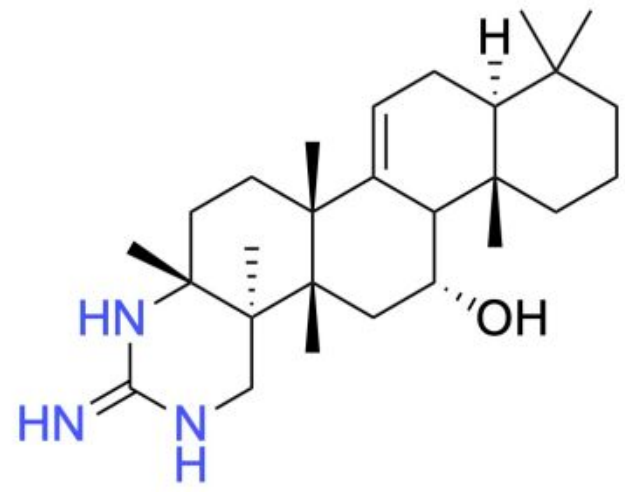

Cybastacine B

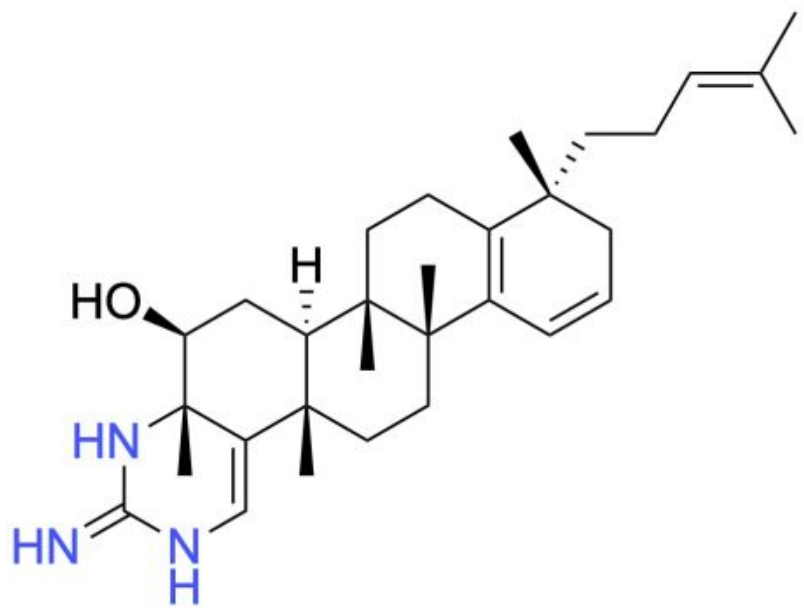

Gromomycin A

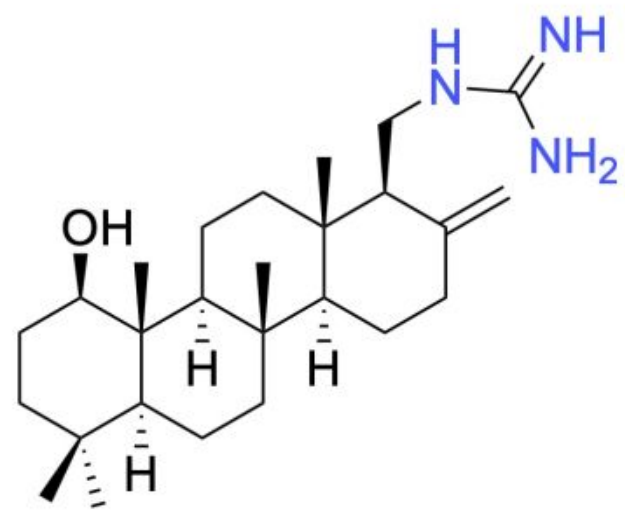

Scytoscalarol

B)

Cybastacine A

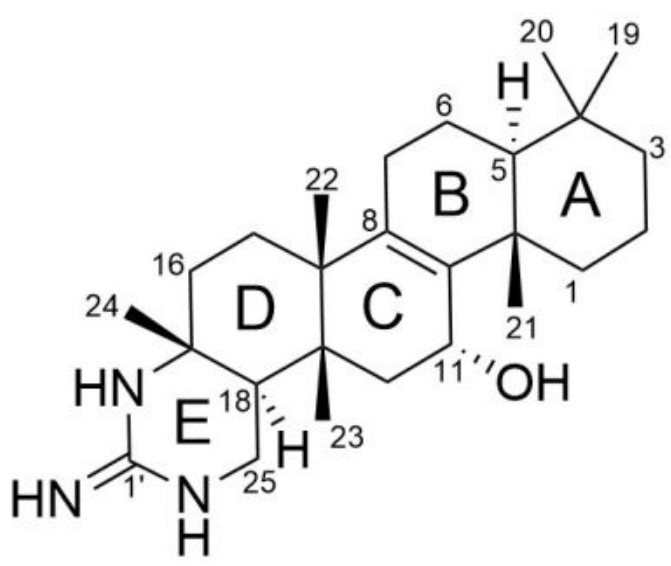

Darumycin A

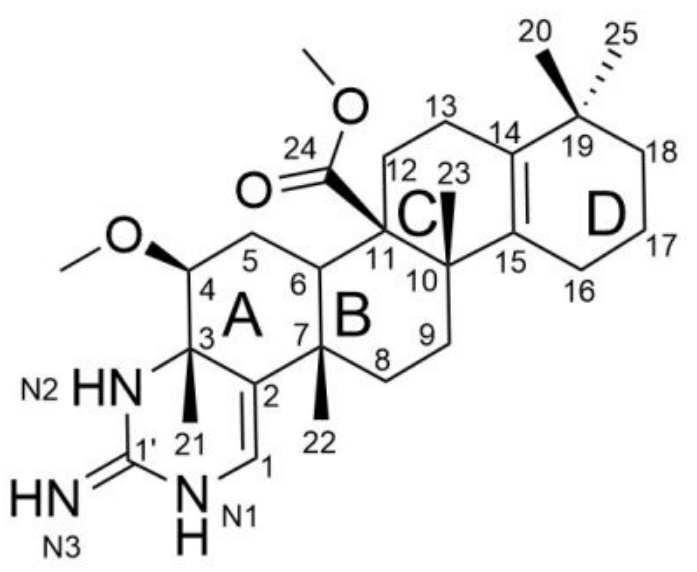

Gromomycin A

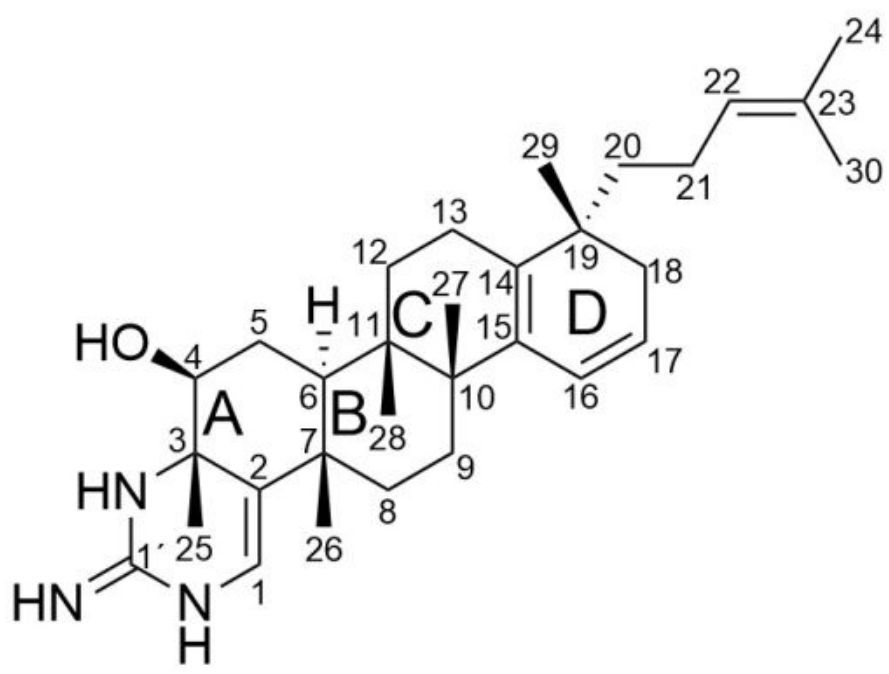

Figure S1: A) Chemical structure of guanidine-containing terpene natural products. The two sesterterpenes cybastacine A and B were isolated from the cyanobacterium *Nostoc* sp. Gromomycin A was purified after heterologous expression in *S. albus* Del14 and its biosynthesis was recently proposed based on stable isotope feeding experiments and targeted gene deletion experiments. Scytoscalarol was purified from the cyanobacterium *Scytonema sp.* All compounds show strong antimicrobial activity against clinically relevant strains. **B) Structures of guanidine-bearing terpenoids: Cybastacine A, Darumycin A and Gromomycin A in comparison.**

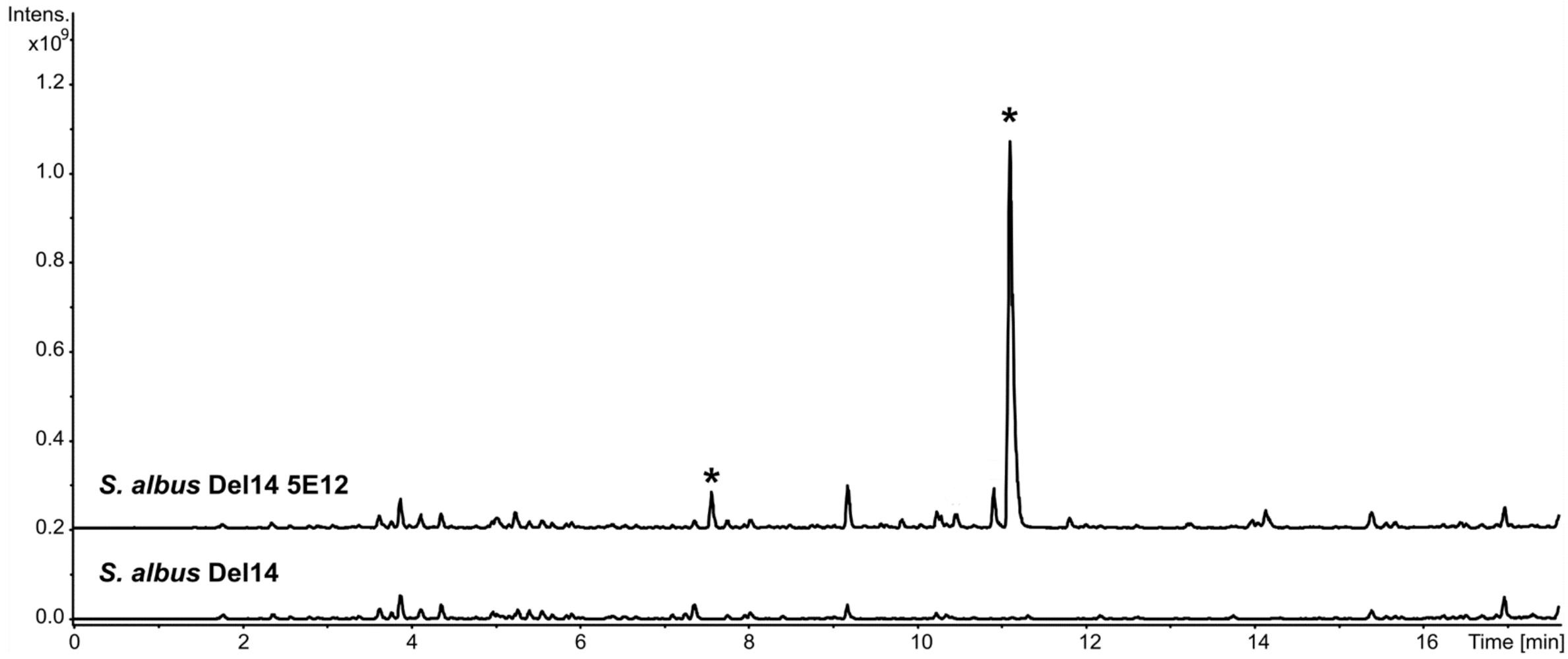

Figure S2: HPLC-MS chromatograms depicting the heterologous expression of the cosmid P05-E12 with darumycin cluster in *S. albus* Del14. The novel peaks are marked by (*) asterisks, representing metabolite *m/z* 486.33 $[M+H]^+$ (left, later degrades to darumycin B (**2**) during purification) and darumycin A (**1**) (right).

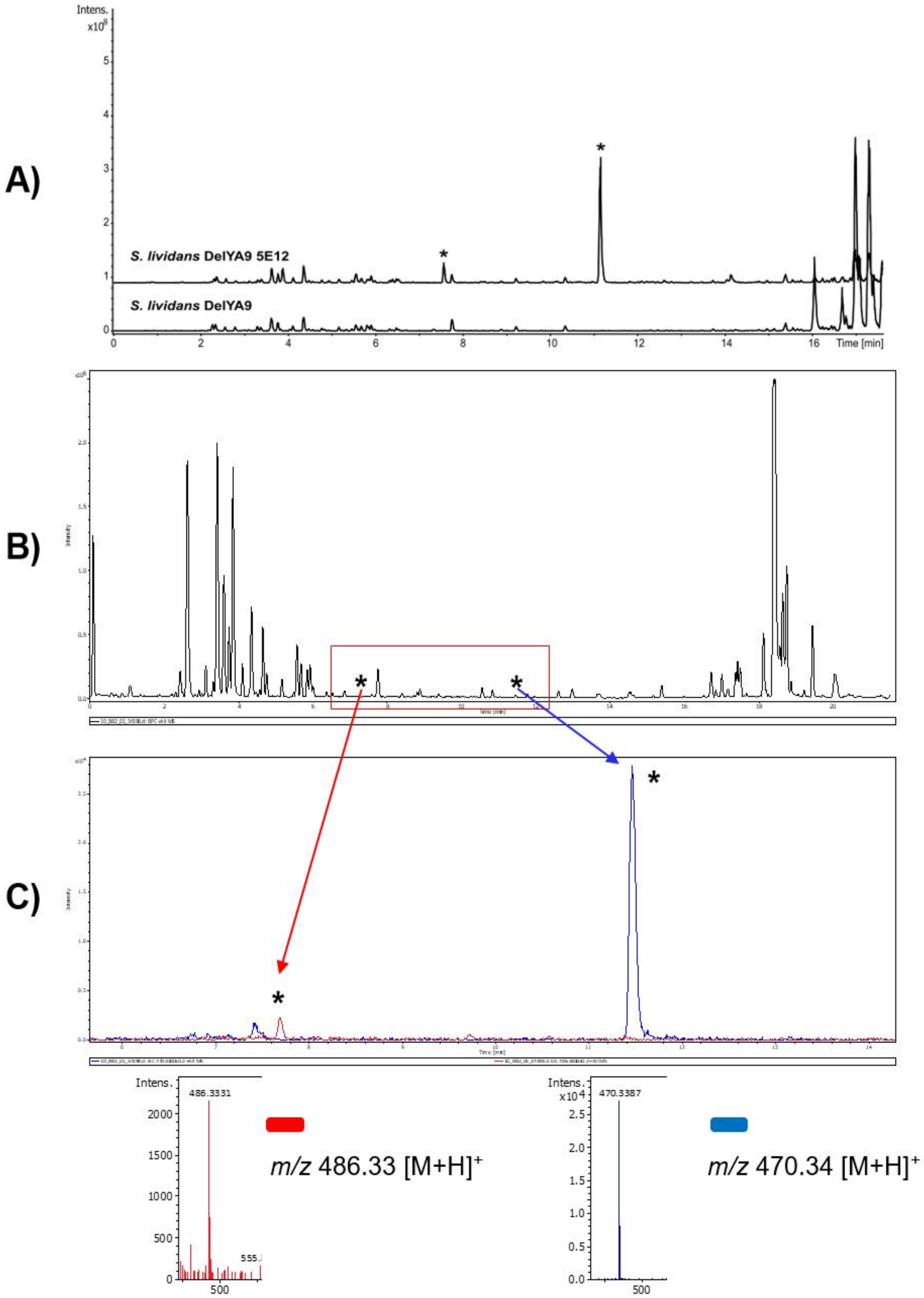

Figure S3: A) HPLC-MS chromatograms of the strains *S. lividans* DelYA9, *S. lividans* DelYA9 cos15AAmInt-5E12. The peak representing metabolite *m/z* 486.33 $[M+H]^+$ (left**,** later degrades to darumycin B (**2**) during purification**)** and darumycin A (**1**) (right) are marked by (*) asterisks. The production of **1** is lower compared to the heterologous host *S. albus* Del14. **B)** HPLC-MS chromatogram of the *M. rubida* strain. The peak representing the metabolite *m/z* 486.33 $[M+H]^+$ (later degraded to darumycin B (**2**) during purification (left, **in red**)) and darumycin A (**1**) (right, **in blue**) are marked by (*) asterisks. **C)** Extracted Ion Chromatogram (EIC) of the *M. rubida* strain.

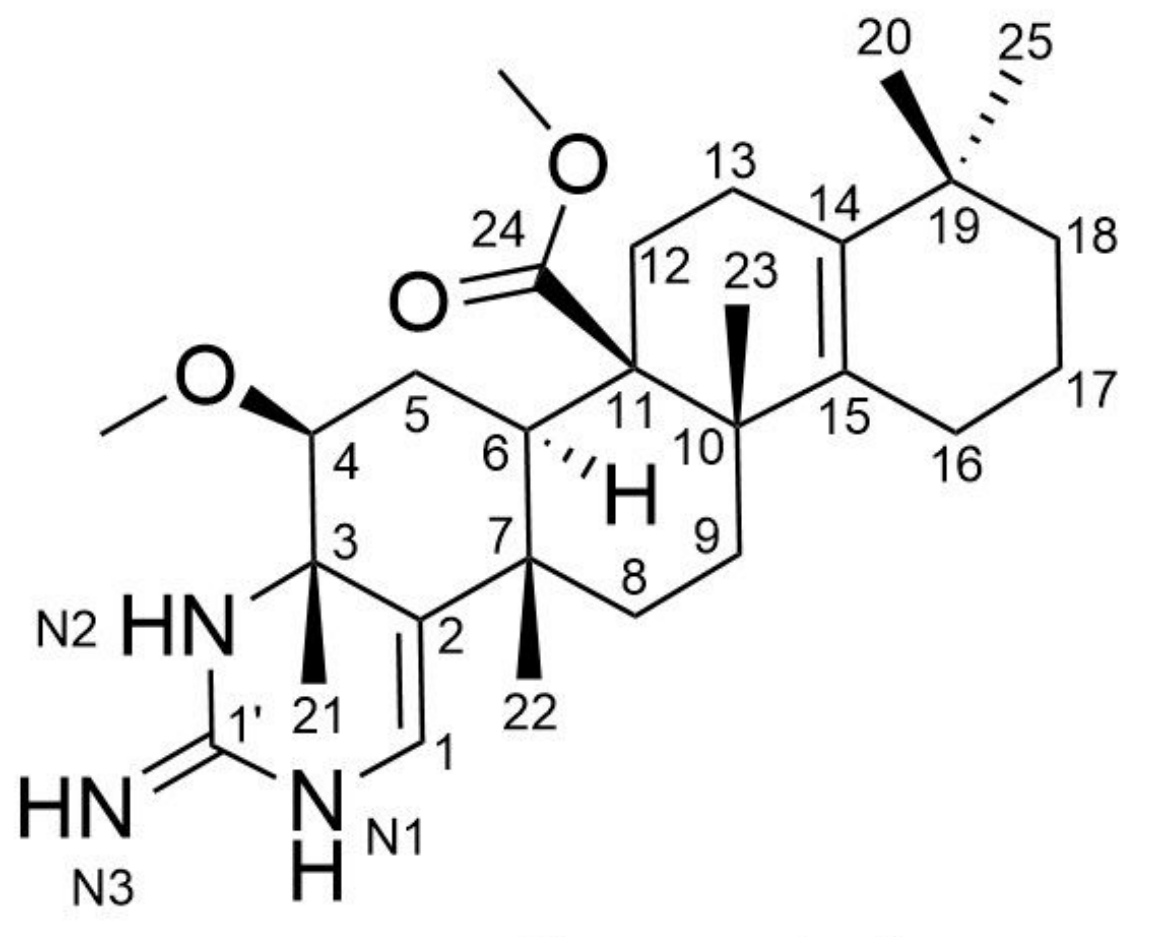

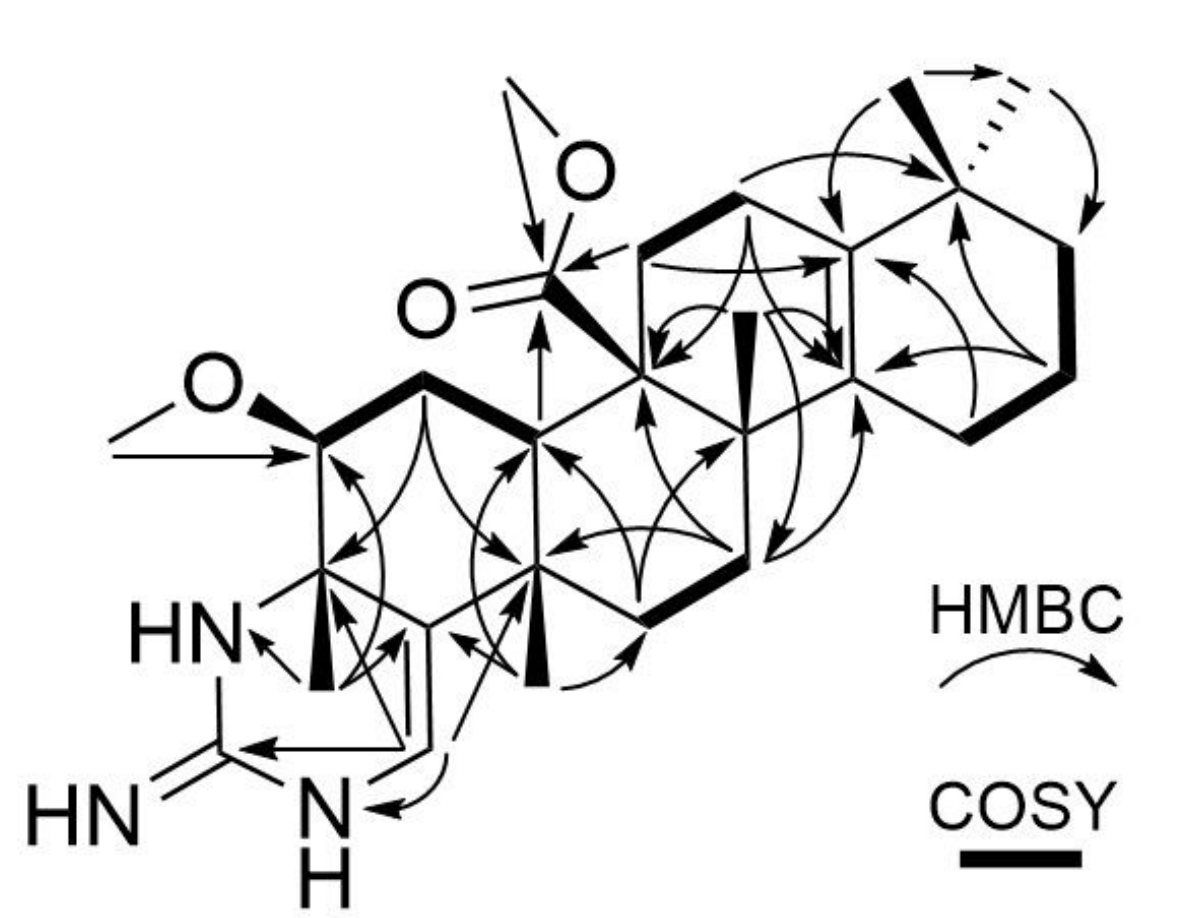

Figure S4: Structure of the darumycin A (**1**).

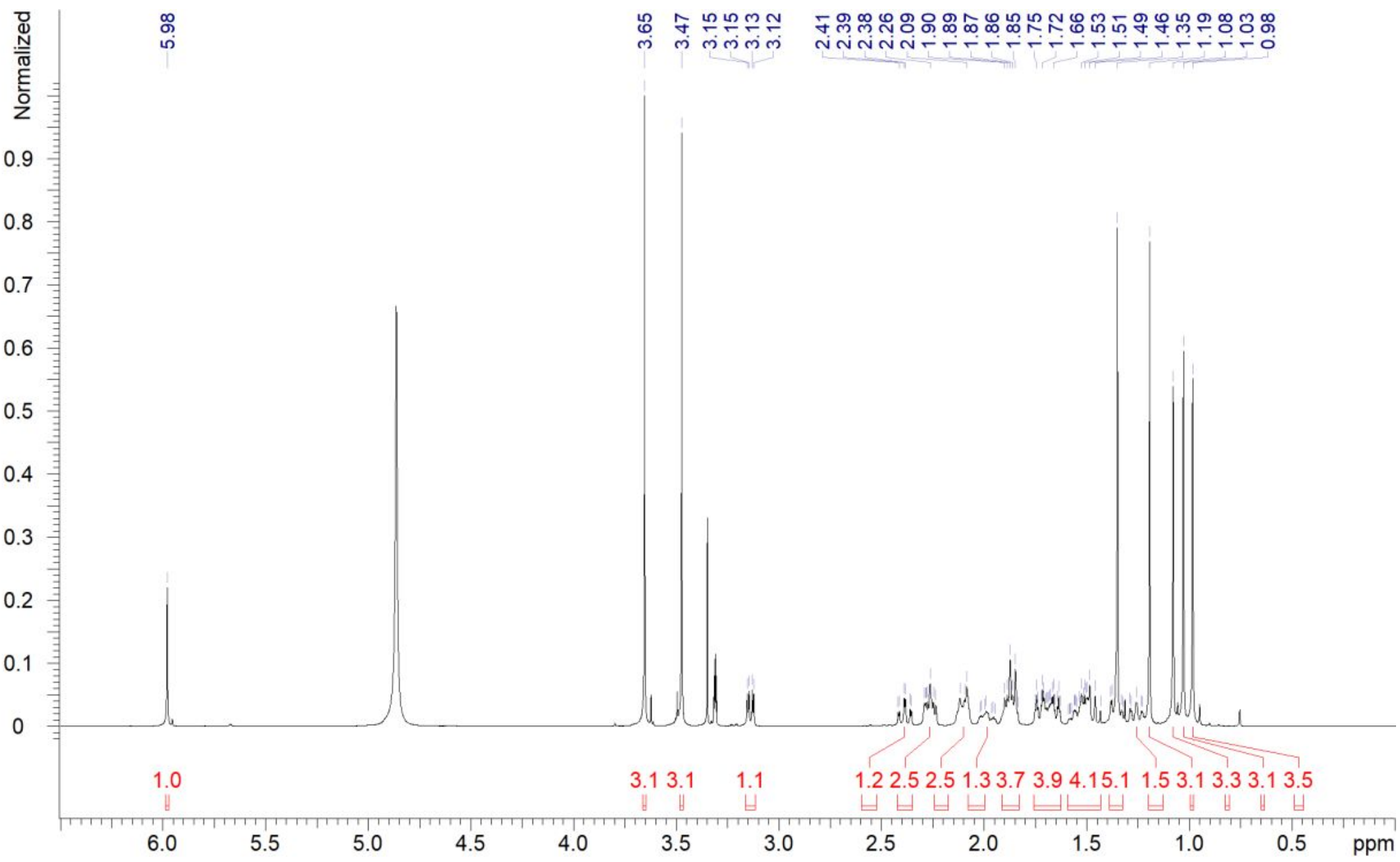

Figure S5: ^{1}H NMR spectrum (500 MHz) of darumycin A (**1**) in CD_3OH.

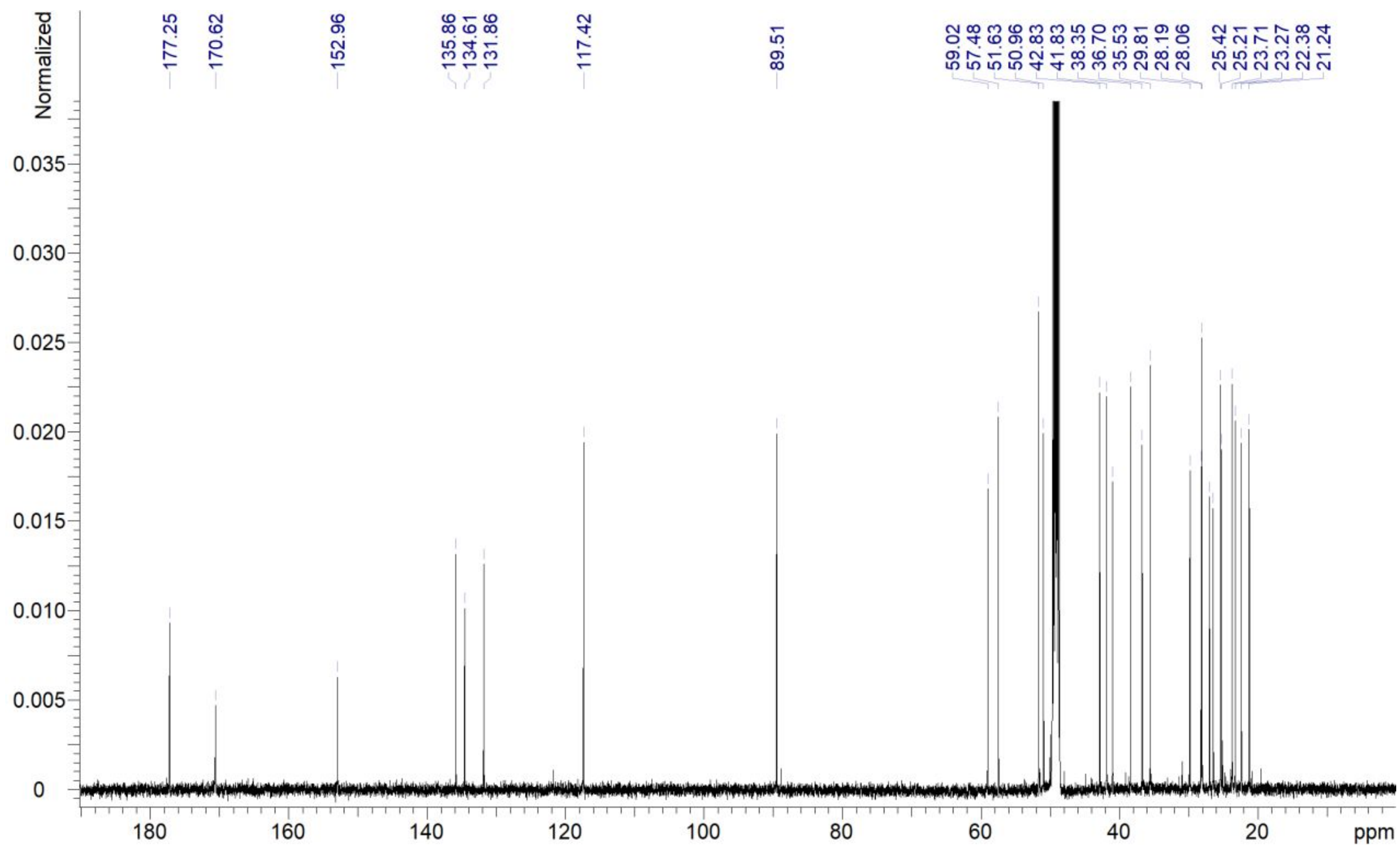

Figure S6: ^{13}C NMR spectrum (125 MHz) of darumycin A (**1**) in CD_3OH.

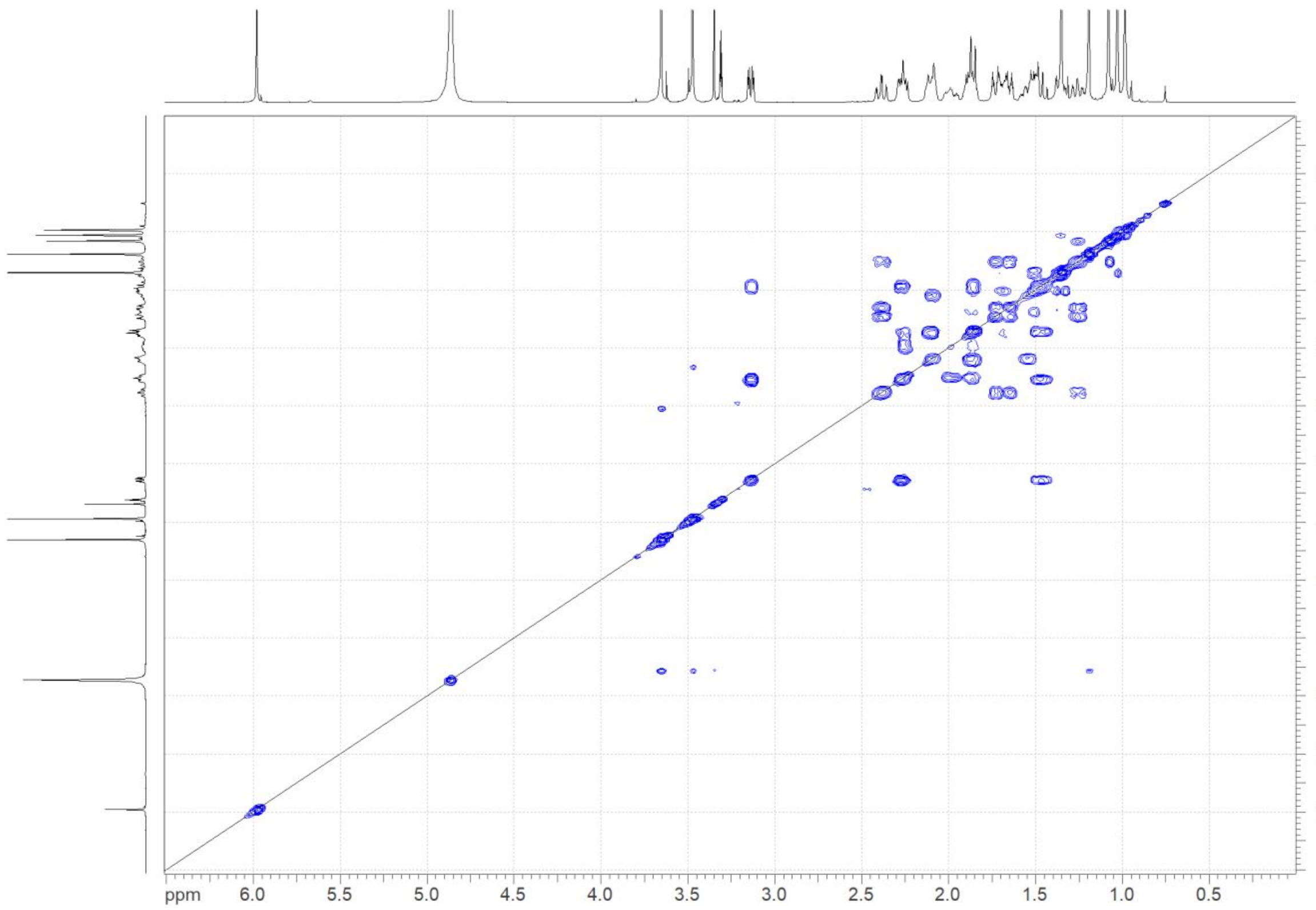

Figure S7: ^{1}H-^{1}H COSY spectrum (500 MHz) of darumycin A (**1**) in CD_3OH.

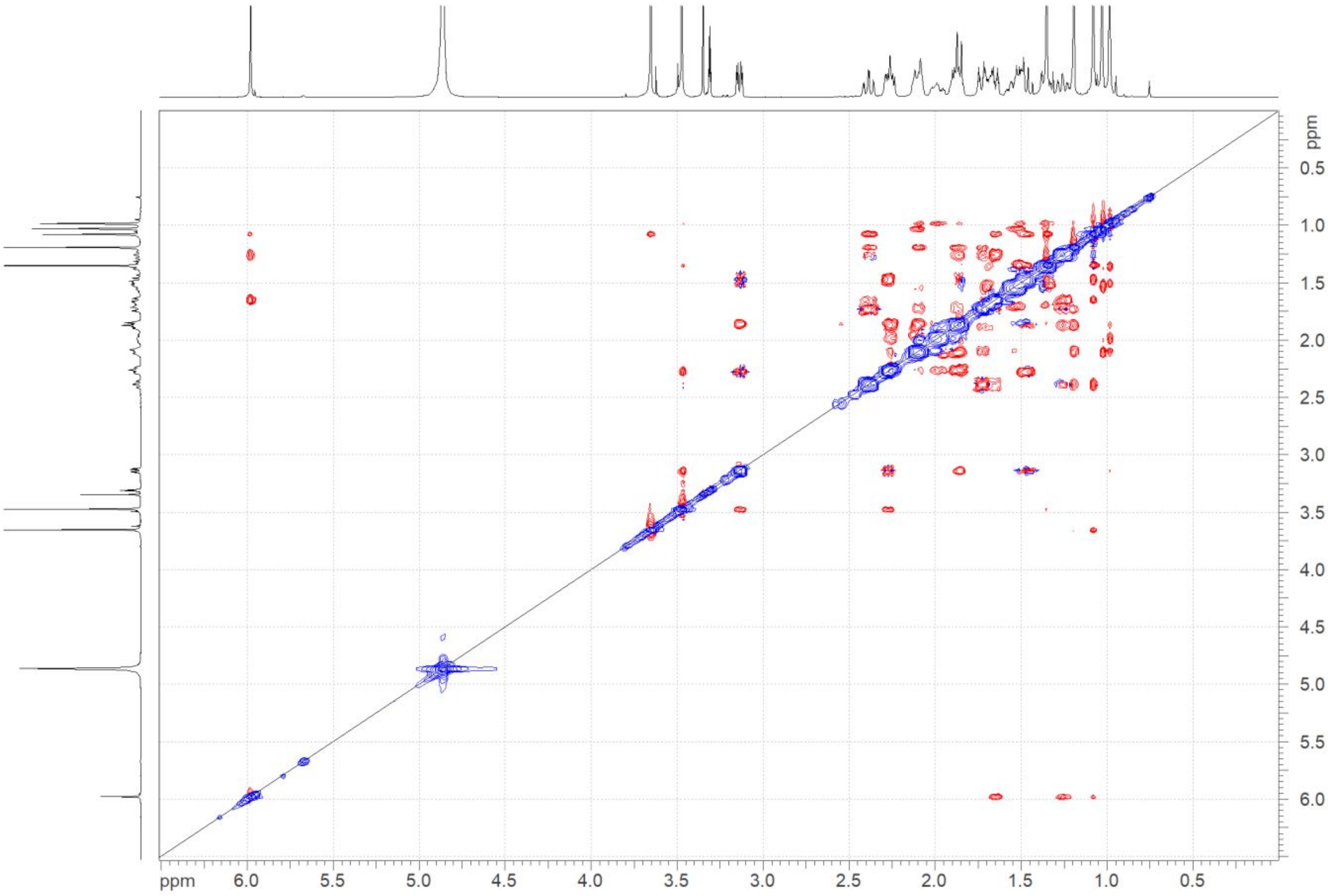

Figure S8: NOESY spectrum (500 MHz) of darumycin A (**1**) in CD_3OH.

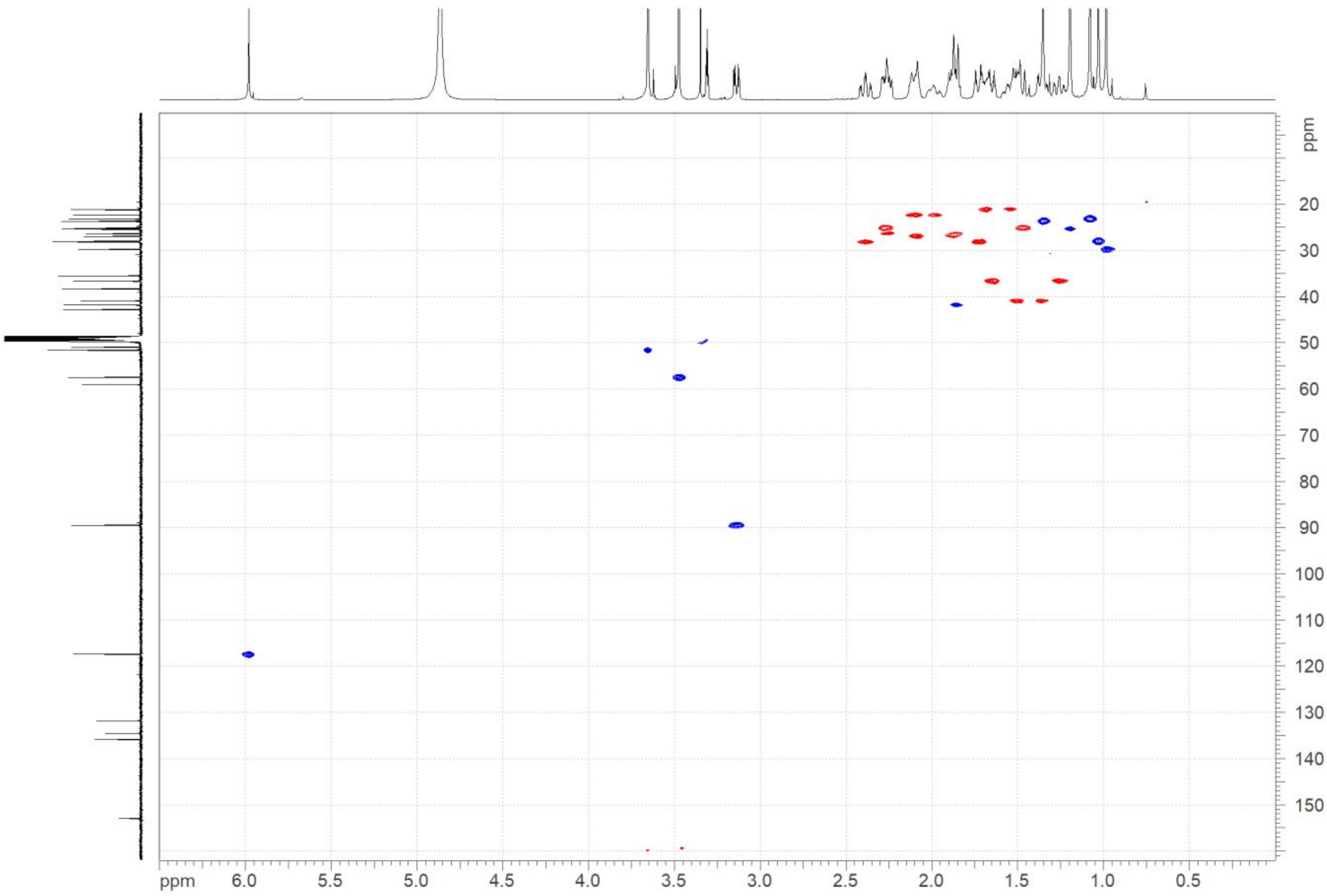

Figure S9: Edited HSQC spectrum (500 MHz) of darumycin A (**1**) in CD_3OH.

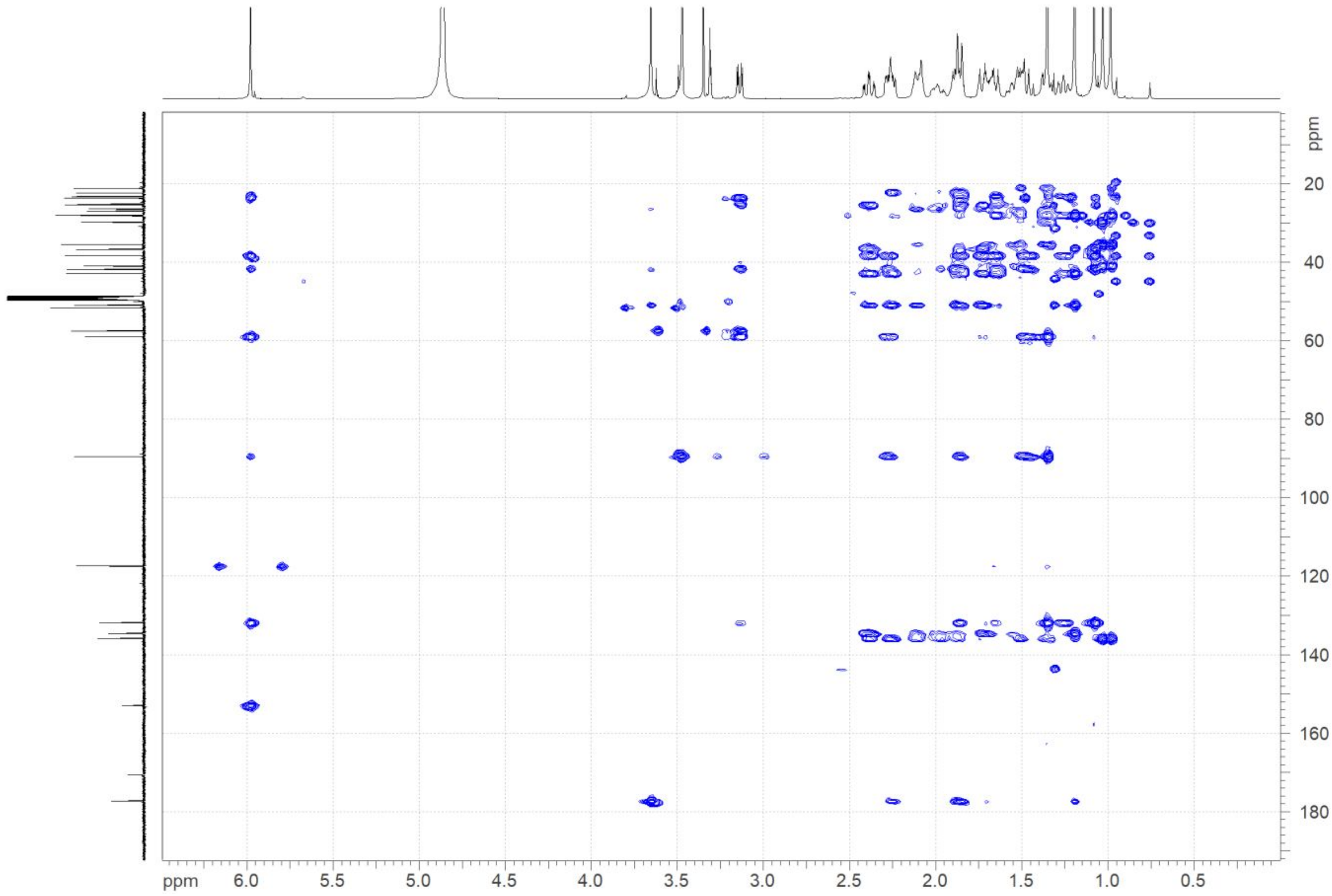

Figure S10: ^{13}C HMBC spectrum (500 MHz) of darumycin A (**1**) in CD_3OH.

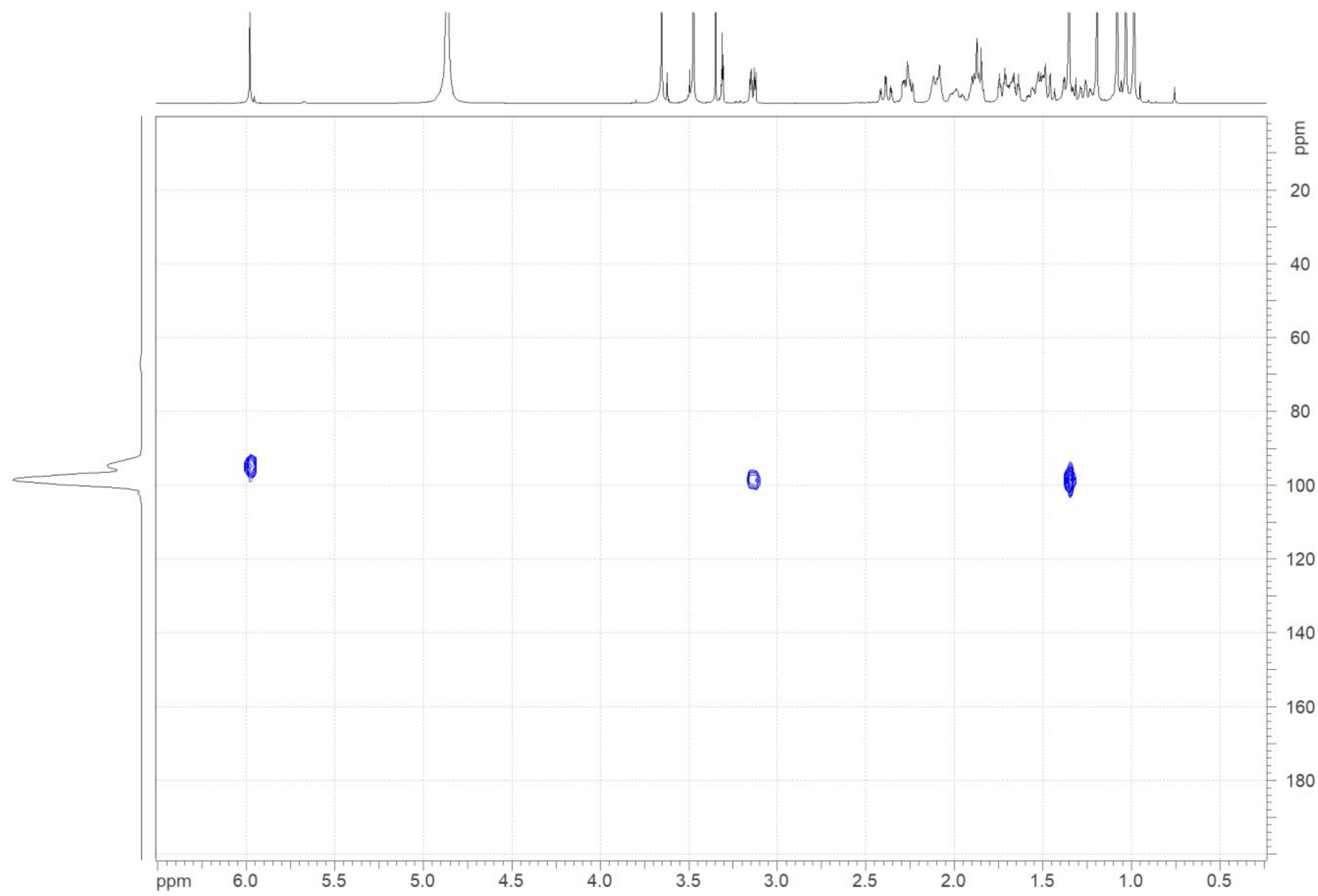

Figure S11: ^{15}N-HMBC spectrum (500 MHz) of darumycin A (**1**) in CD_3OH.

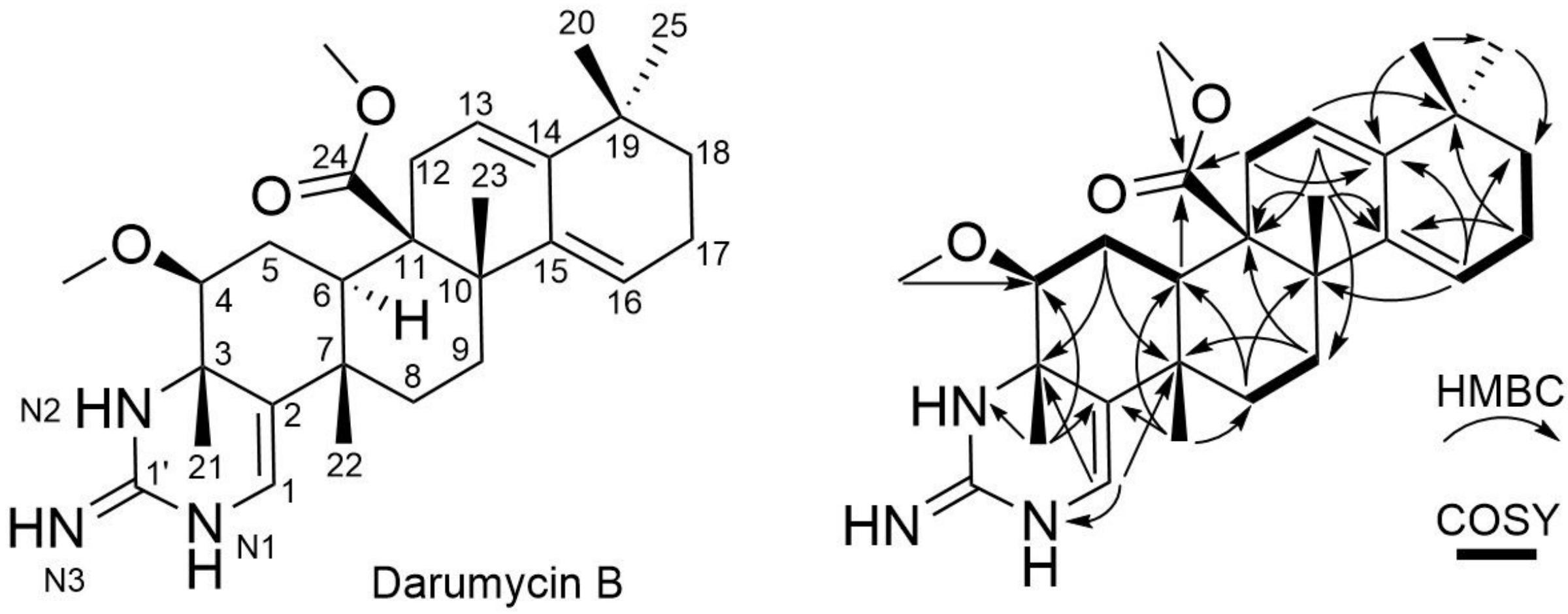

Figure S12: Structure of darumycin B (**2**).

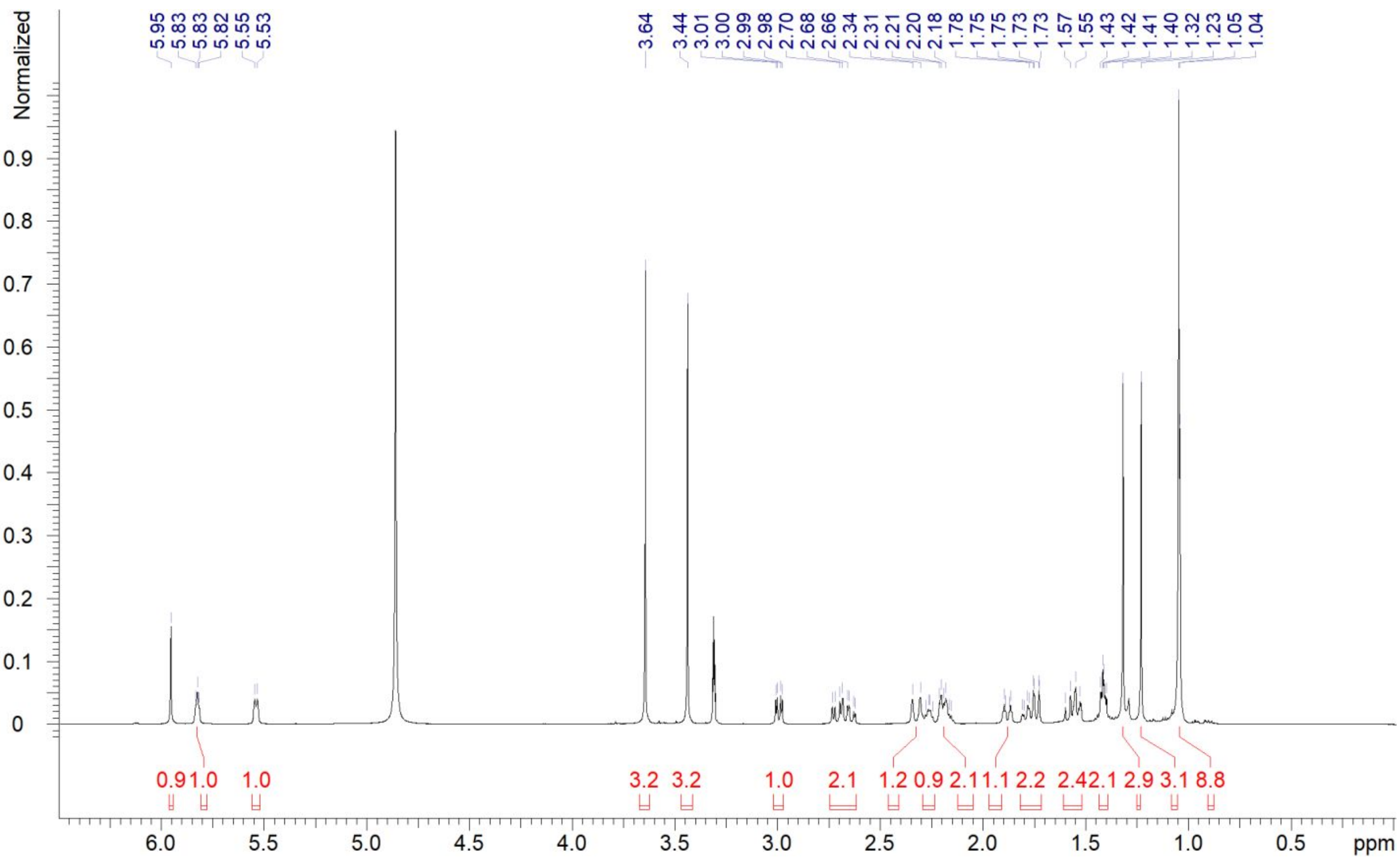

Figure S13: ^{1}H-NMR spectrum (500 MHz) of darumycin B (**2**) in CD_3OD.

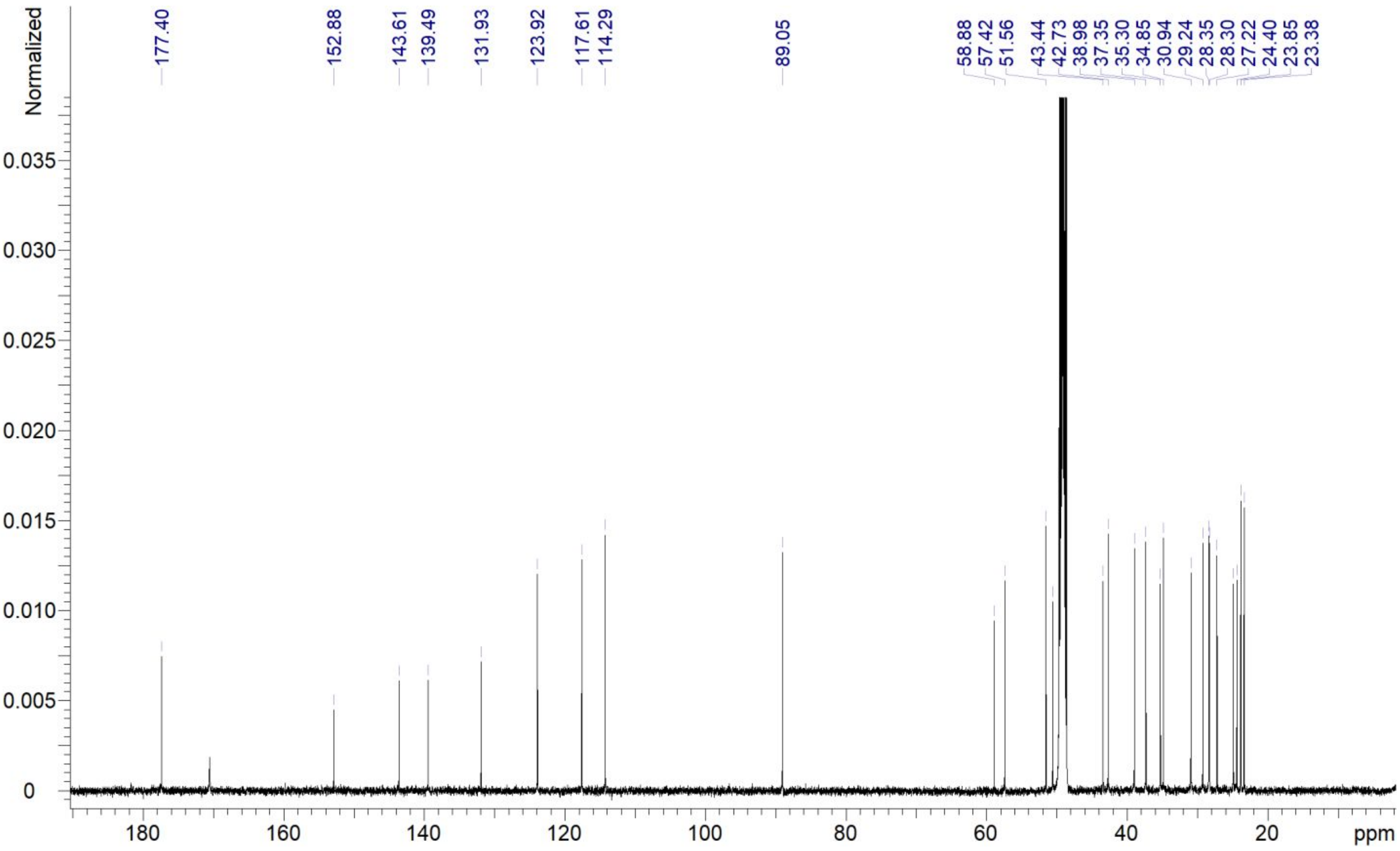

Figure S14: ^{13}C-NMR spectrum (125 MHz) of darumycin B (**2**) in CD_3OD.

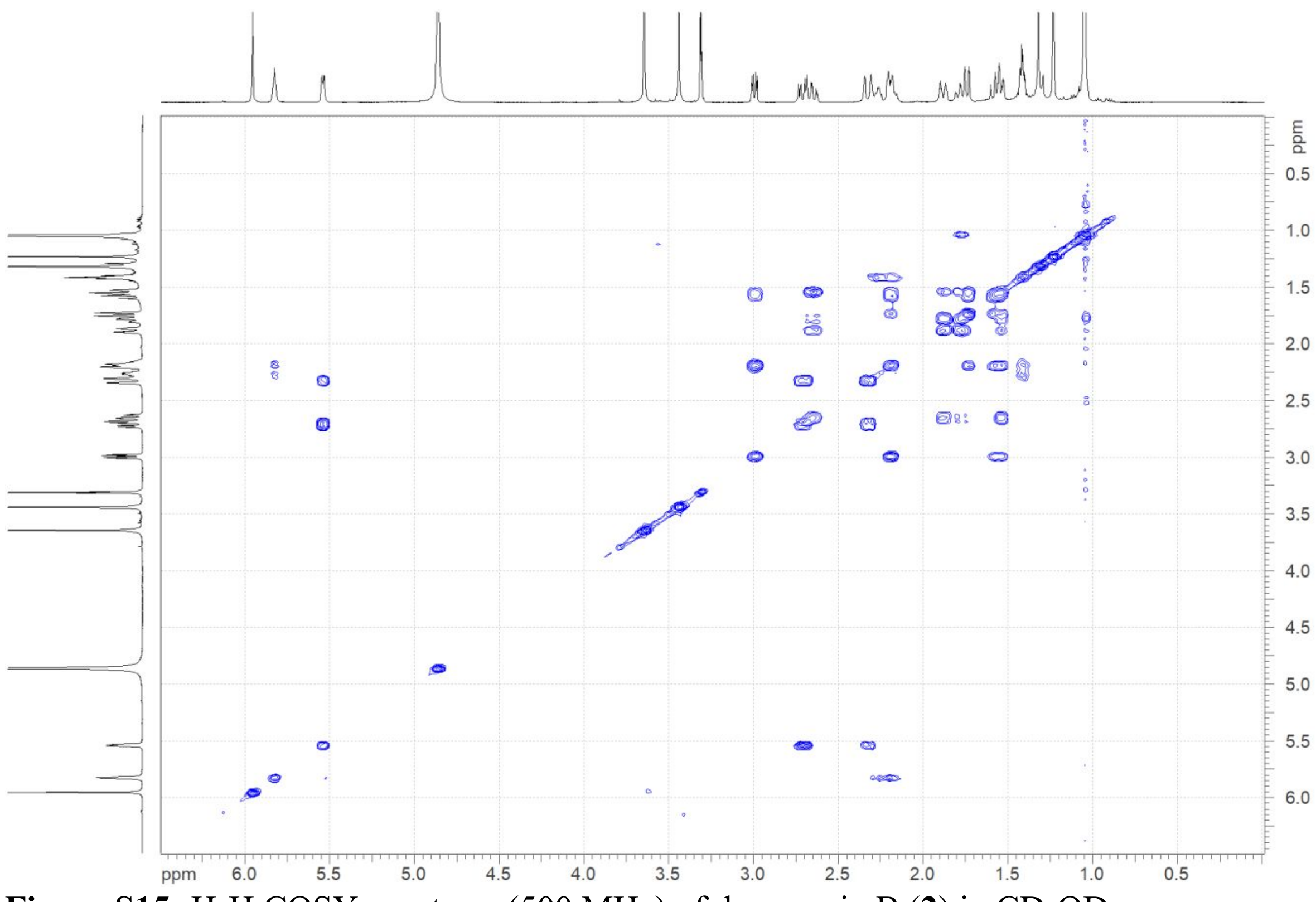

Figure S15: H-H COSY spectrum (500 MHz) of darumycin B (**2**) in CD_3OD.

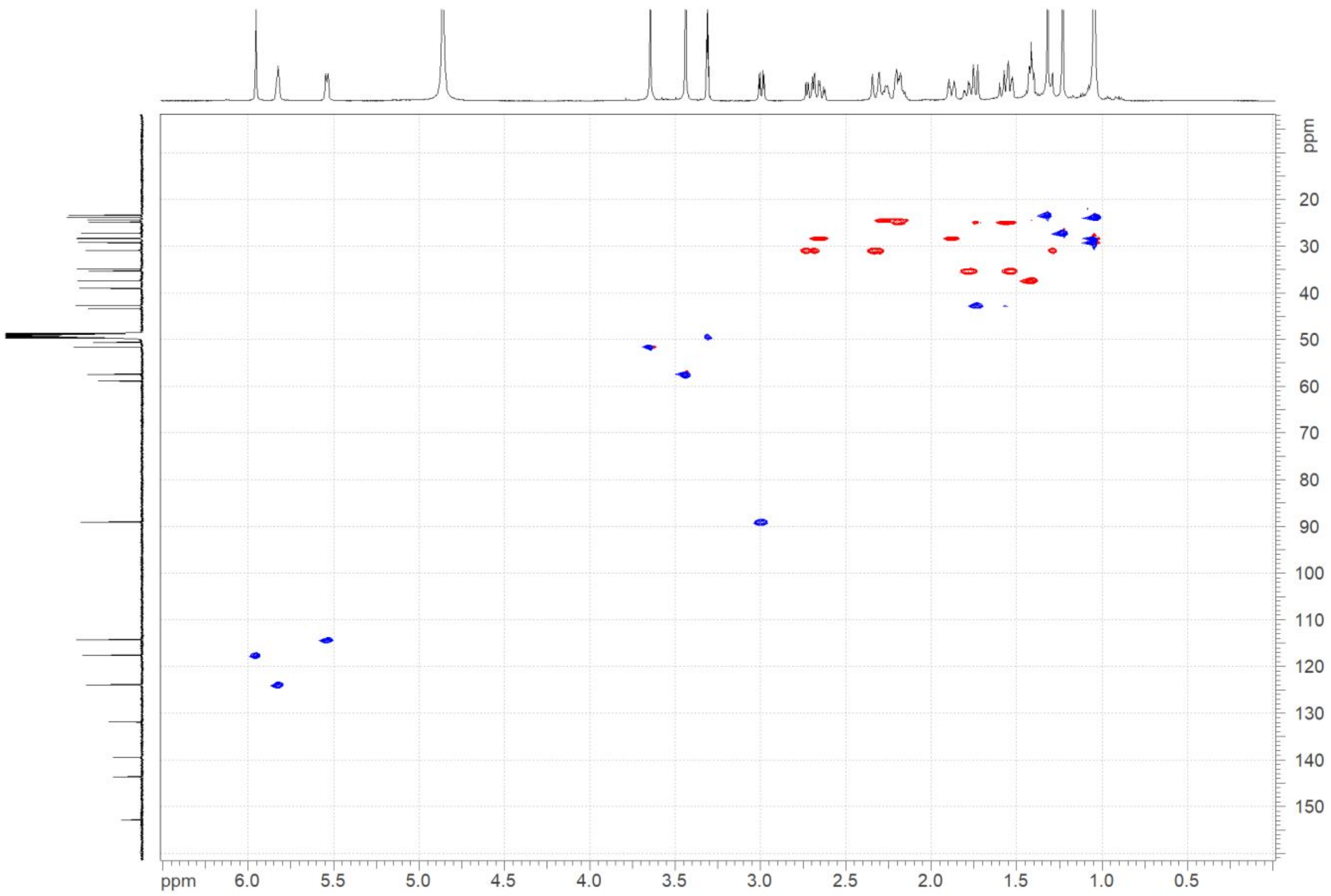

Figure S16: Edited HSQC spectrum (500 MHz) of darumycin B (**2**) in CD_3OD.

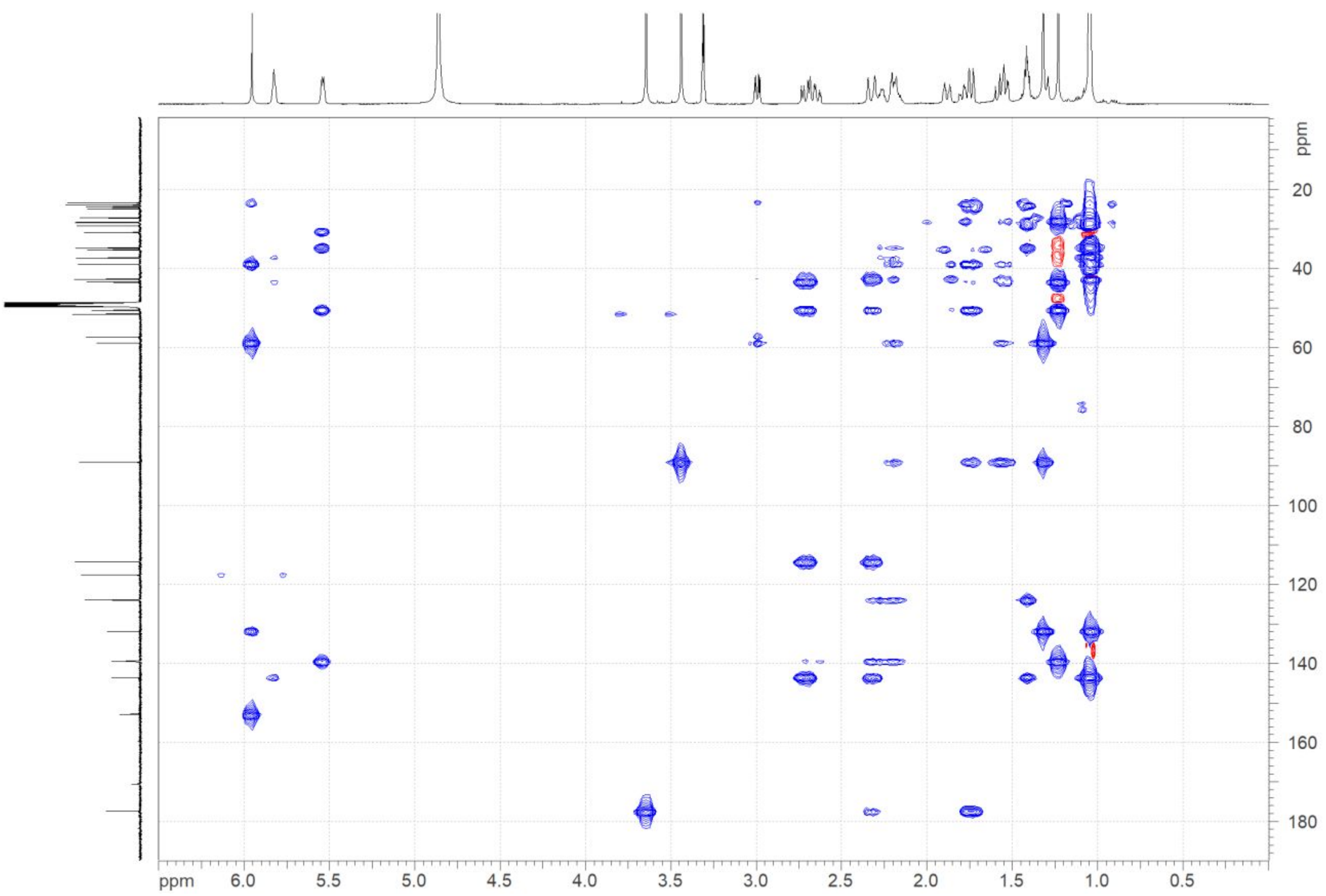

Figure S17: ^{13}C-HMBC spectrum (500 MHz) of darumycin B (2) in CD_3OD.

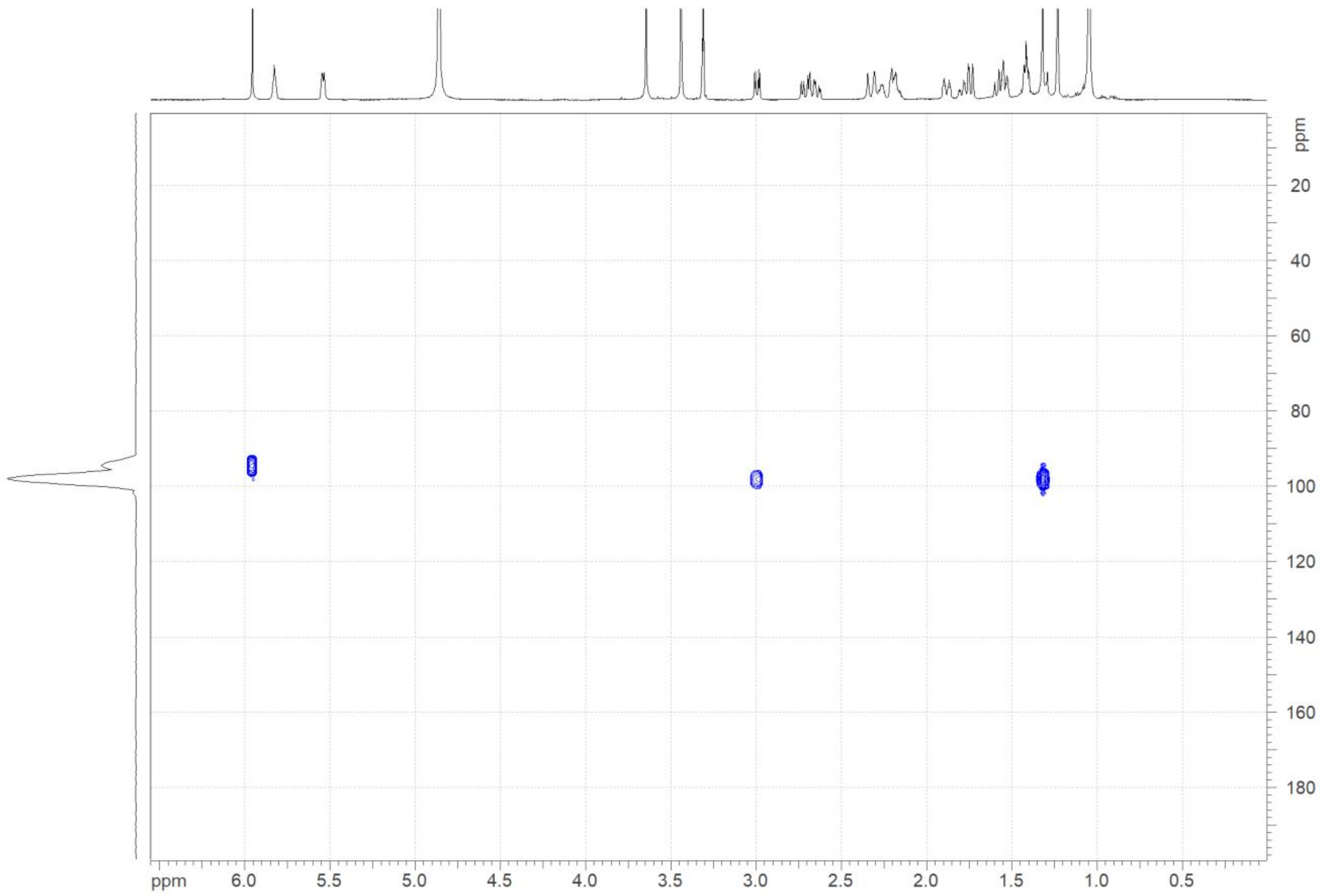

Figure S18: ^{15}N HMBC spectrum (500 MHz) of darumycin B (**2**) in CD_3OD.

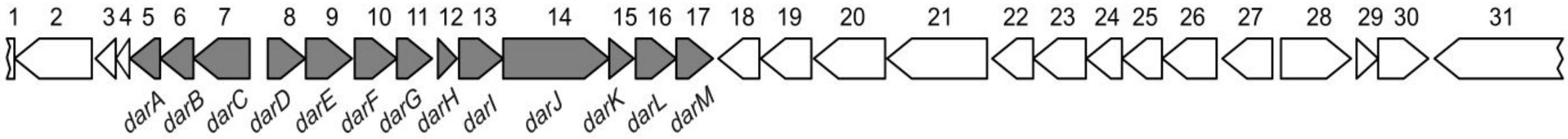

Figure S19: Diagram of the DNA segment containing the darumycin biosynthetic gene cluster, depicted in grey.

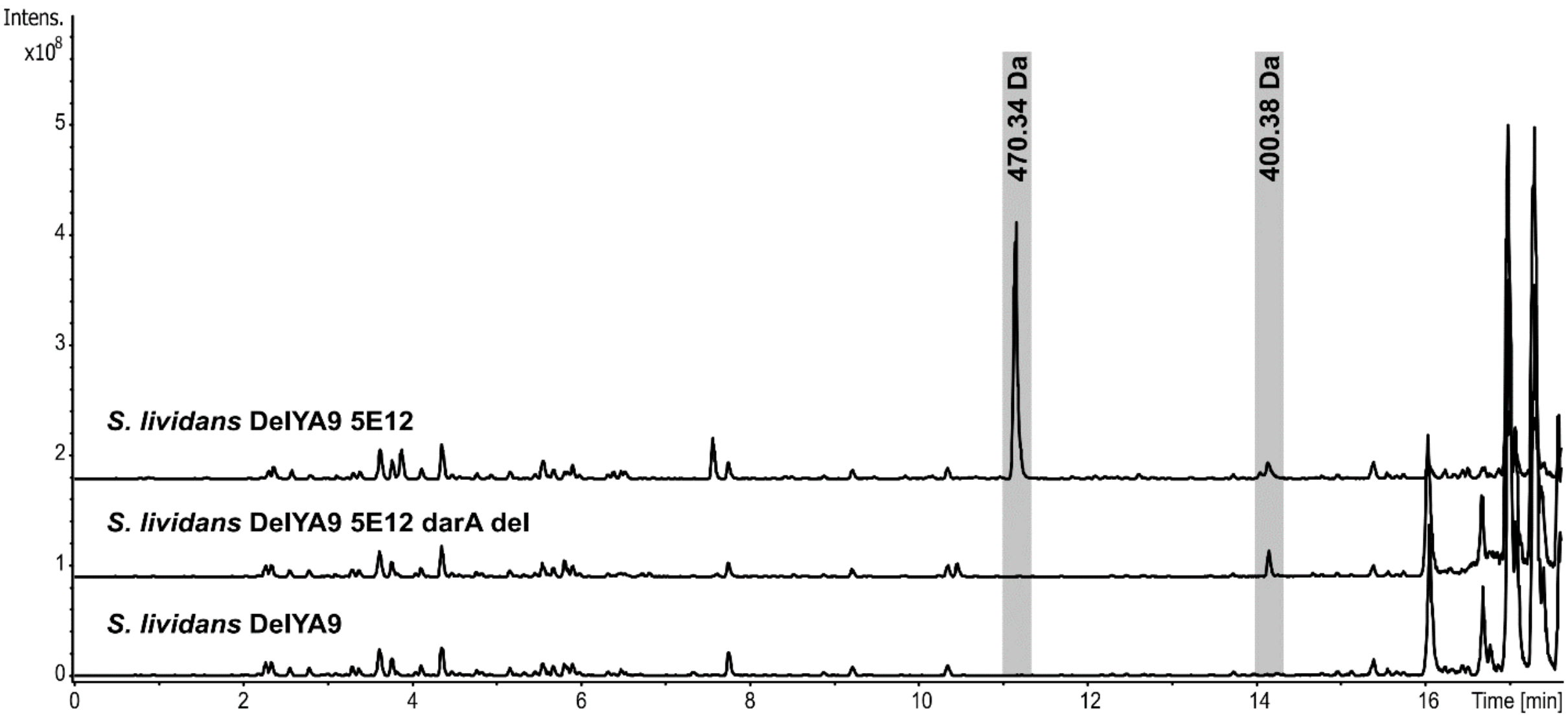

Figure S20A: HPLC-MS chromatograms of the strains *S. lividans* DelYA9, *S. lividans* DelYA9 cos15AAmInt-5E12, *S. lividans* DelYA9 cos15AAmInt-5E12_darA del. The peaks representing darumycin A (**1**) (left) and pentaprenylguanidine (**3**) (right) are marked by grey rectangles. B)

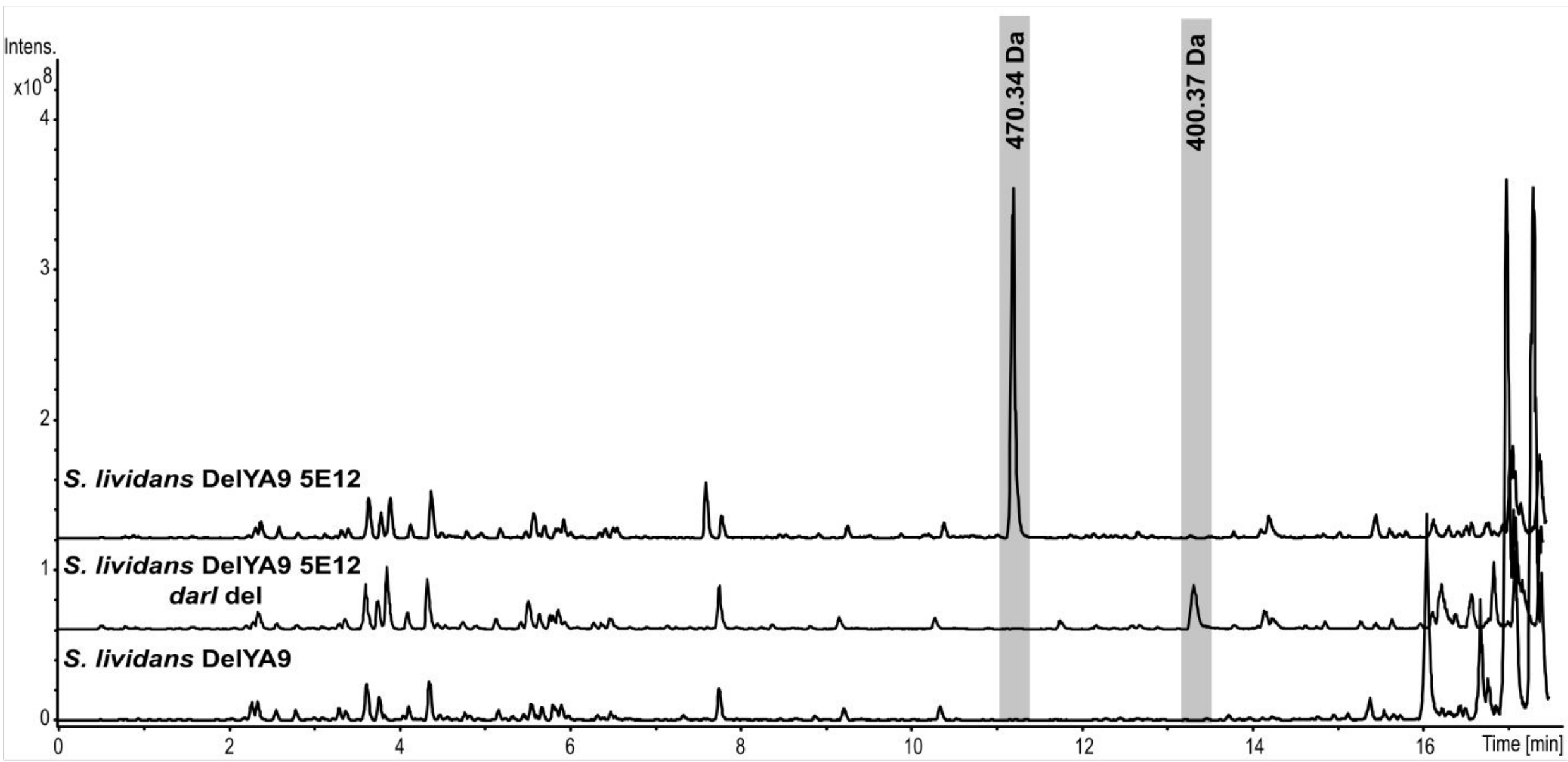

Figure S20B: HPLC-MS chromatograms of the strains *S. lividans* DelYA9, *S. lividans* DelYA9 cos15AAmInt-5E12, *S. lividans* DelYA9 cos15AAmInt-5E12_darI del. The peaks representing darumycin A (**1**) (left) and pentaprenylguanidine (**3**) (right) are marked by grey rectangles.

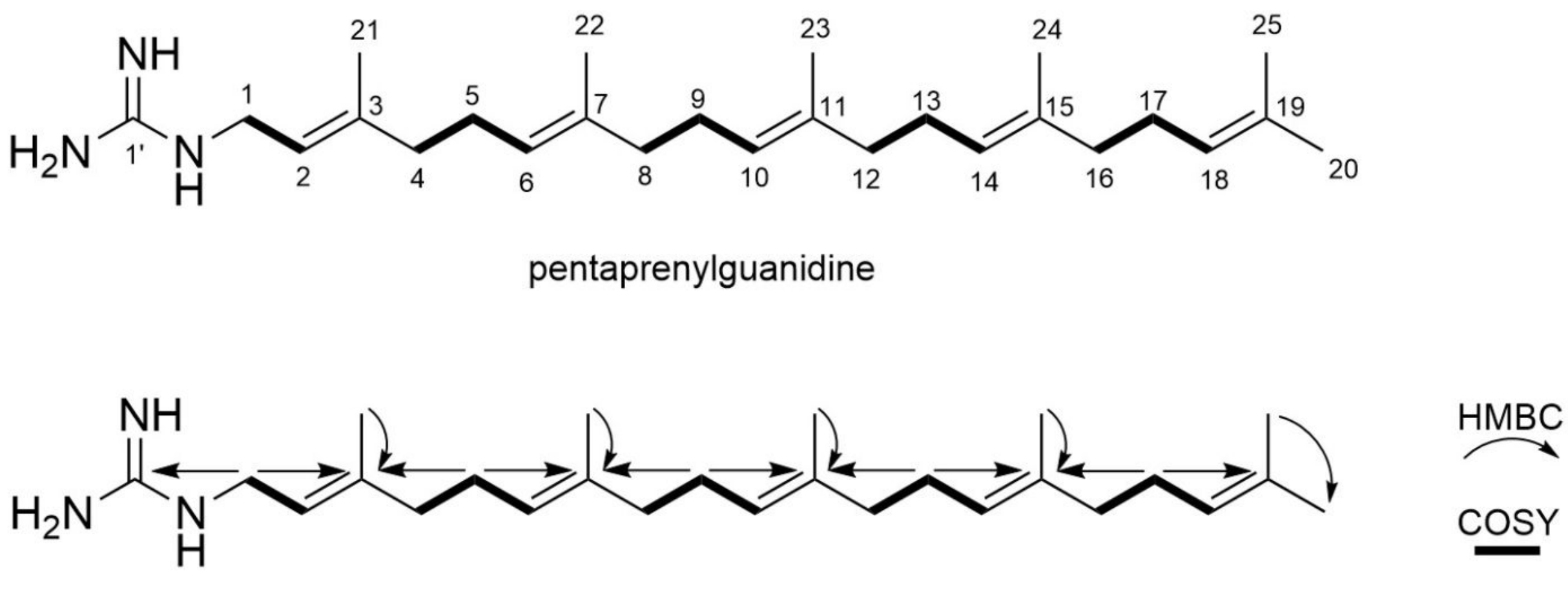

Figure S21: Structure of pentaprenylguanidine (**3**).

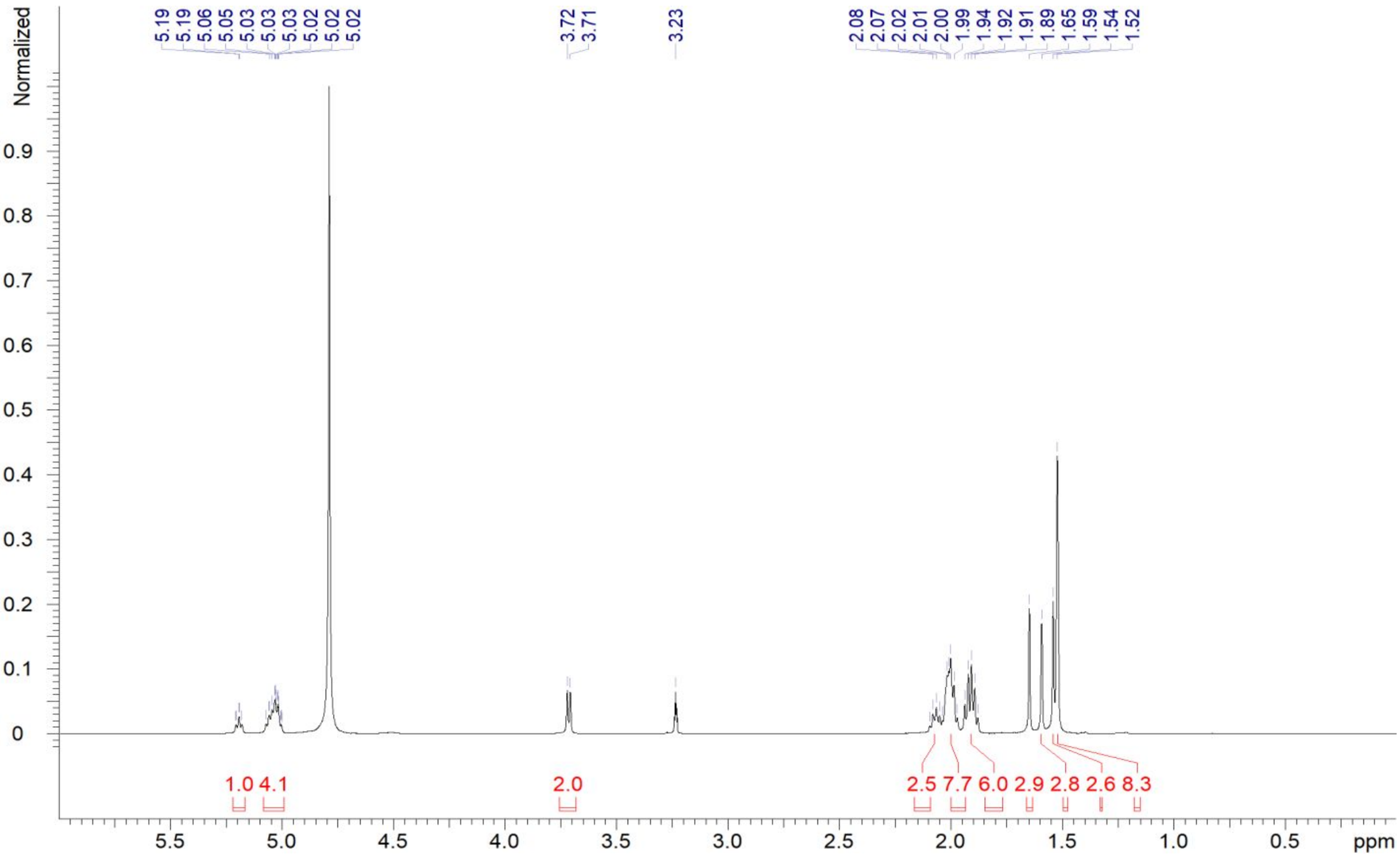

Figure S22: ^{1}H-NMR spectrum (500 MHz) of pentaprenylguanidine (**3**) in CD_3OD.

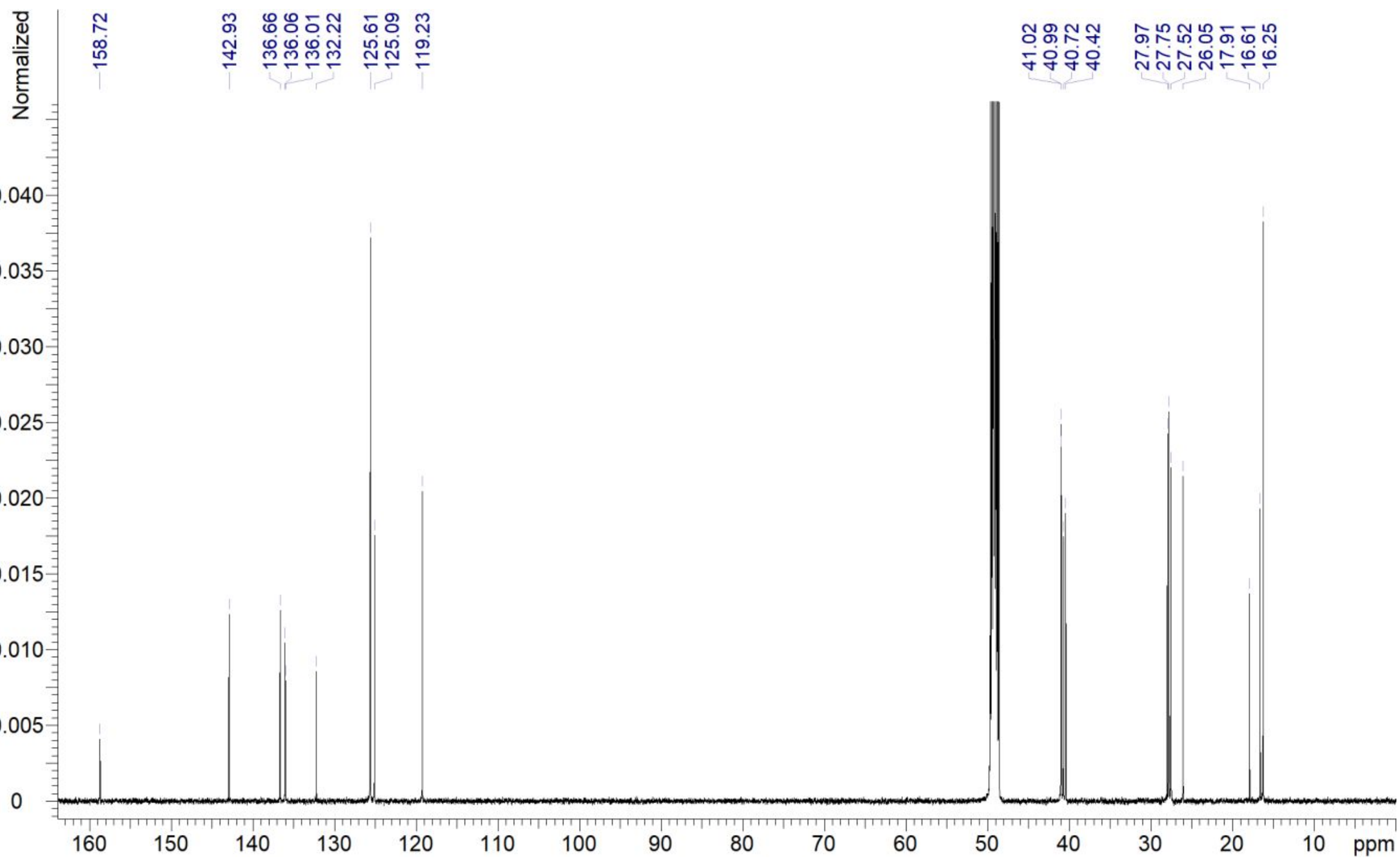

Figure S23: ^{13}C-NMR spectrum (125 MHz) of pentaprenylguanidine (**3**) in CD_3OD.

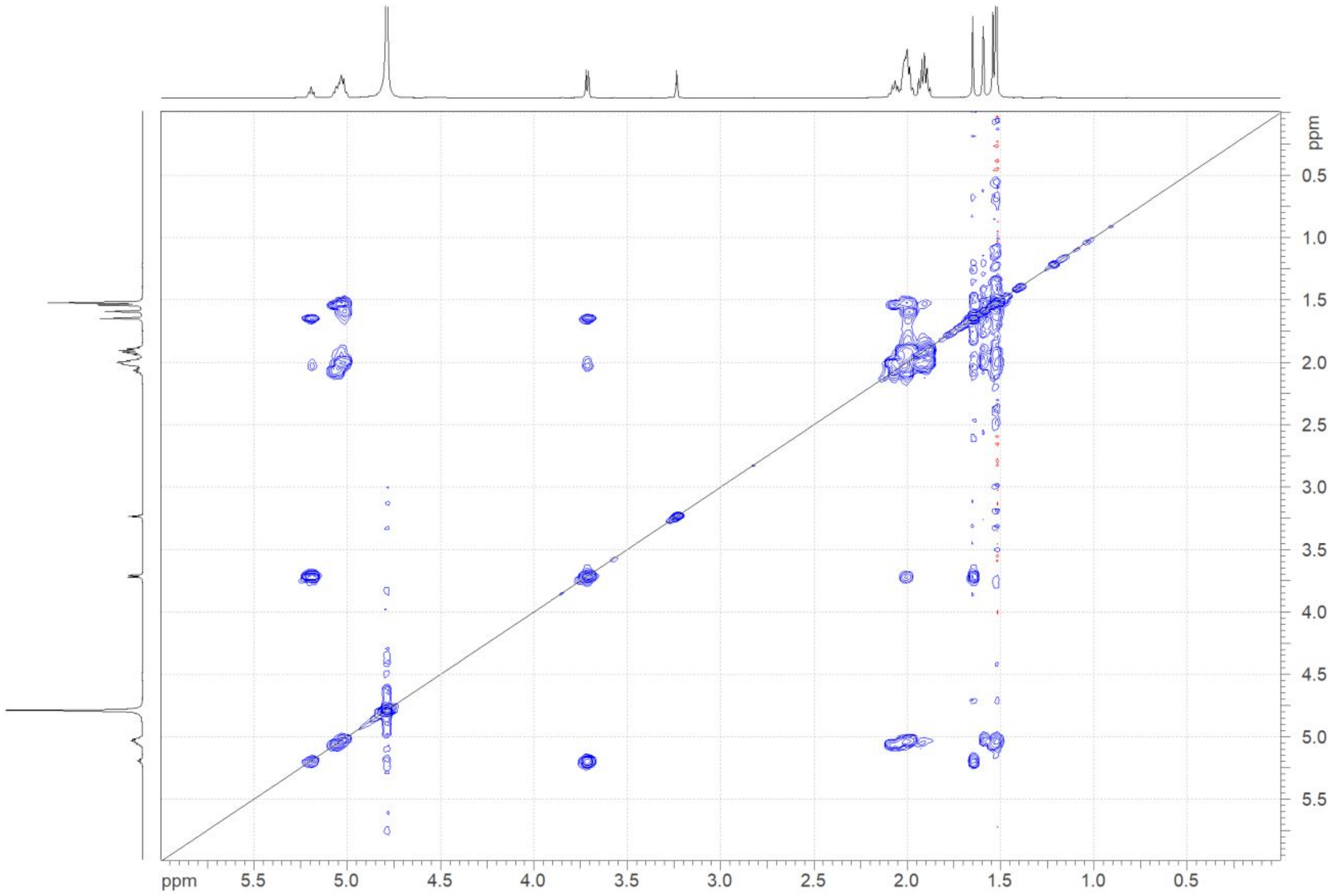

Figure S24: H-H COSY spectrum (500 MHz) of pentaprenylguanidine (**3**) in CD_3OD.

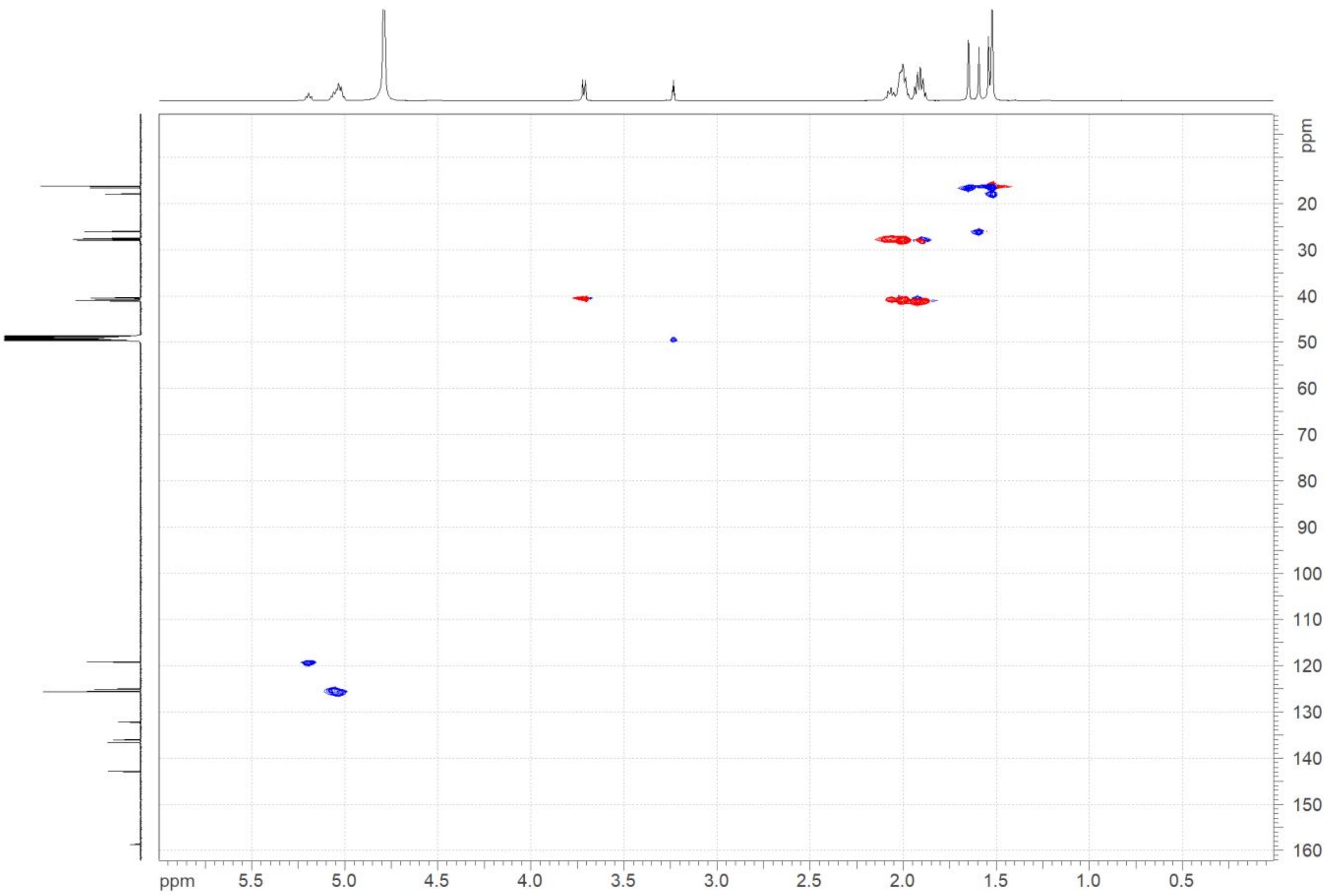

Figure S25: Edited HSQC spectrum (500 MHz) of pentaprenylguanidine (**3**) in CD_3OD.

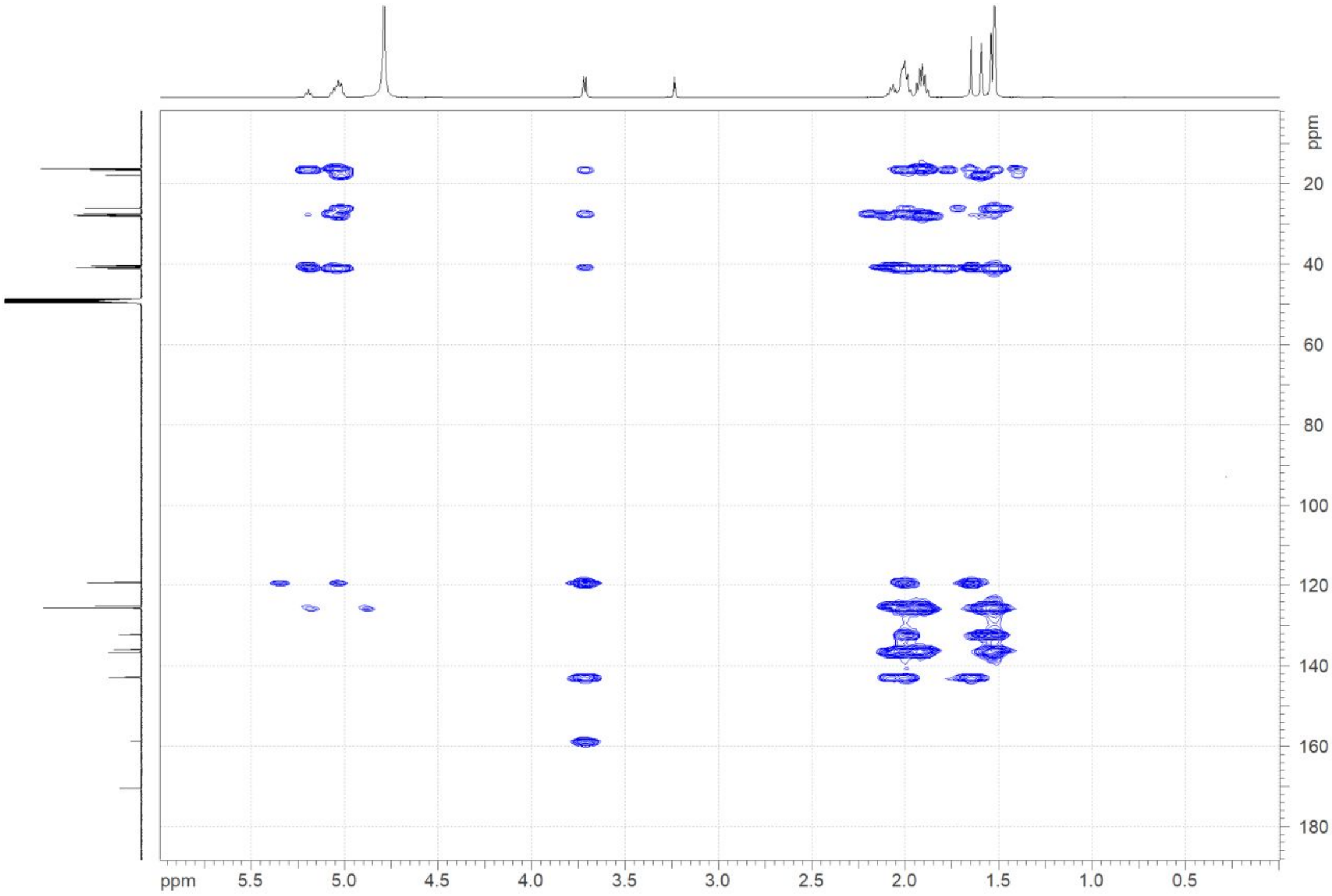

Figure S26: HMBC spectrum (500 MHz) of pentaprenylguanidine (**3**) in CD_3OD.

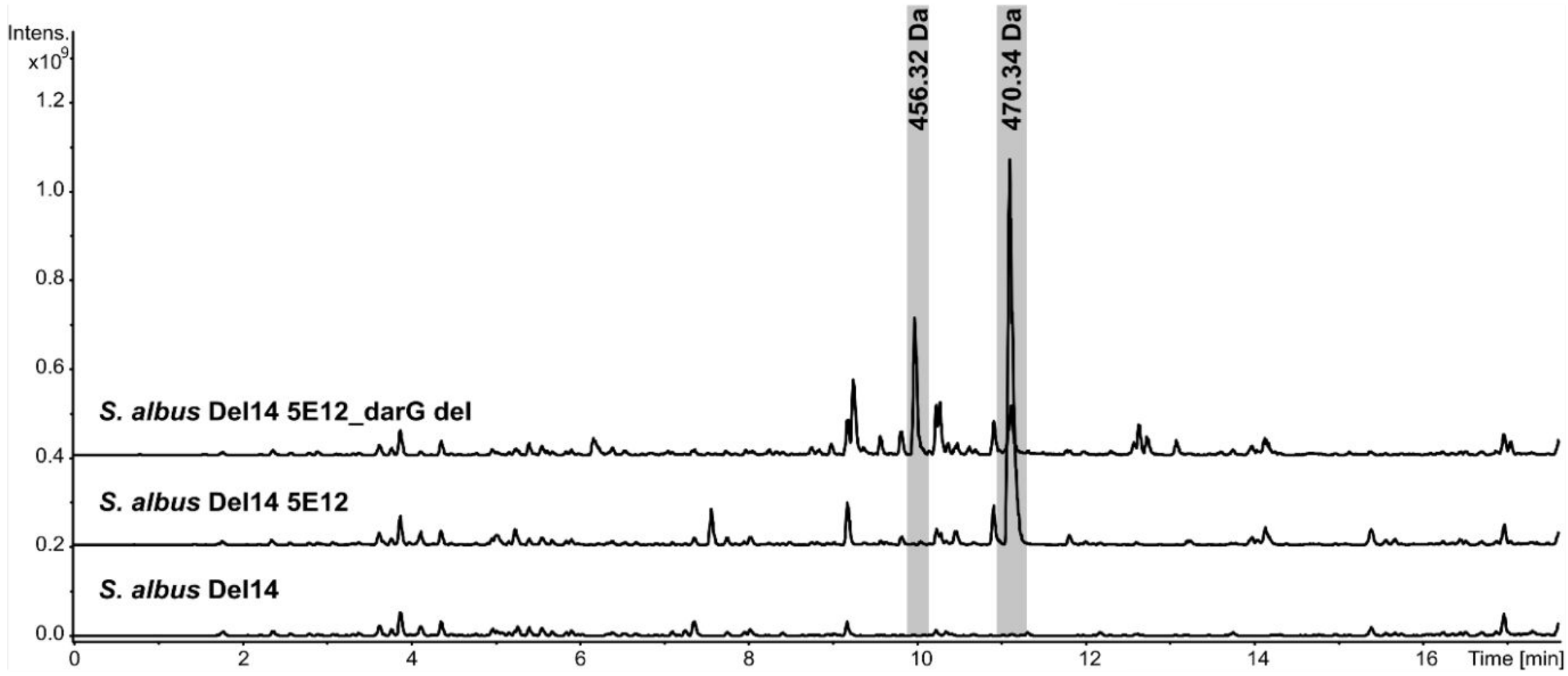

Figure S27: HPLC-MS chromatograms of the strains *S. albus* Del14, *S. albus* Del14 cos15AAmInt-5E12, *S. albus* Del14 cos15AAmInt-5E12_darG del. The peaks representing darumycin A (**1**) and darumycin C (**4**) derivative are marked by grey rectangles.

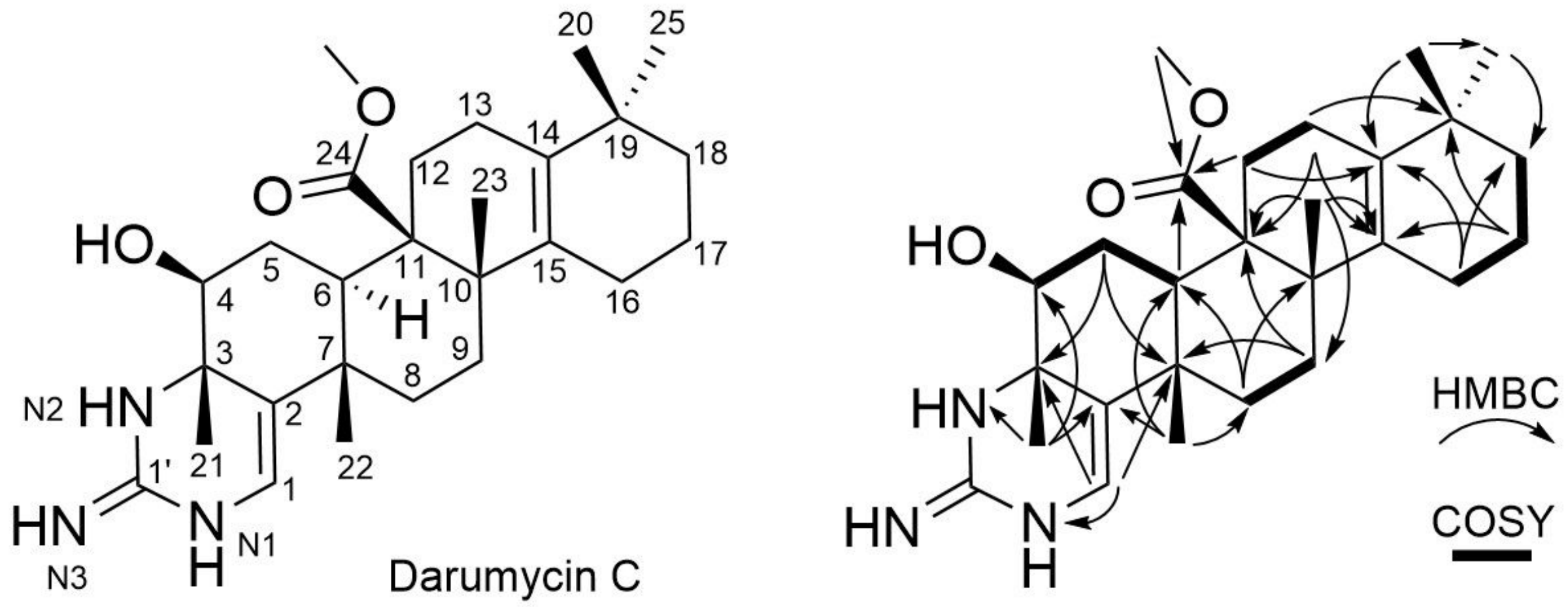

Figure S28: Structure of darumycin C (**4**).

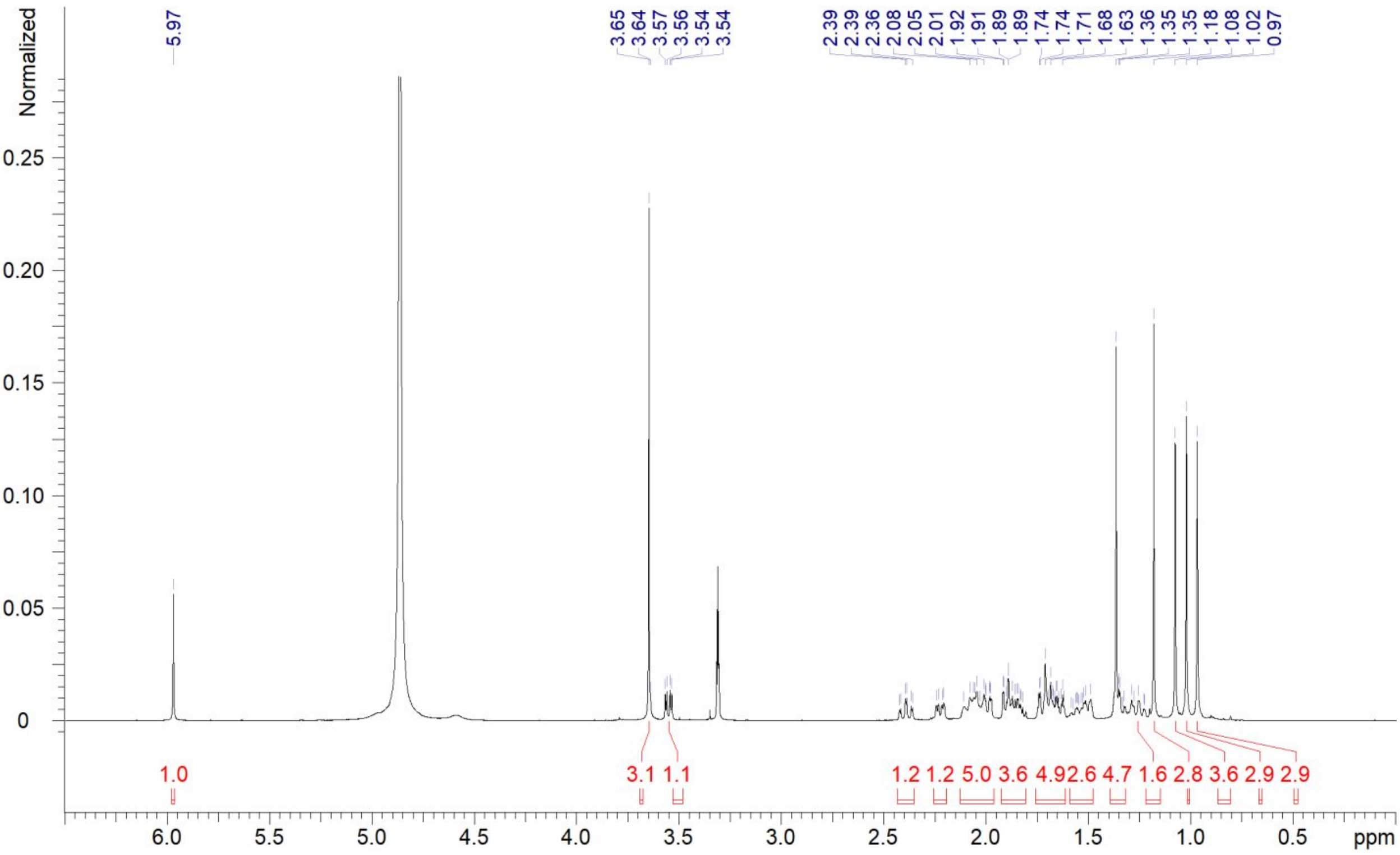

Figure S29: ^{1}H-NMR spectrum (500 MHz) of darumycin C (**4**) in CD_3OD.

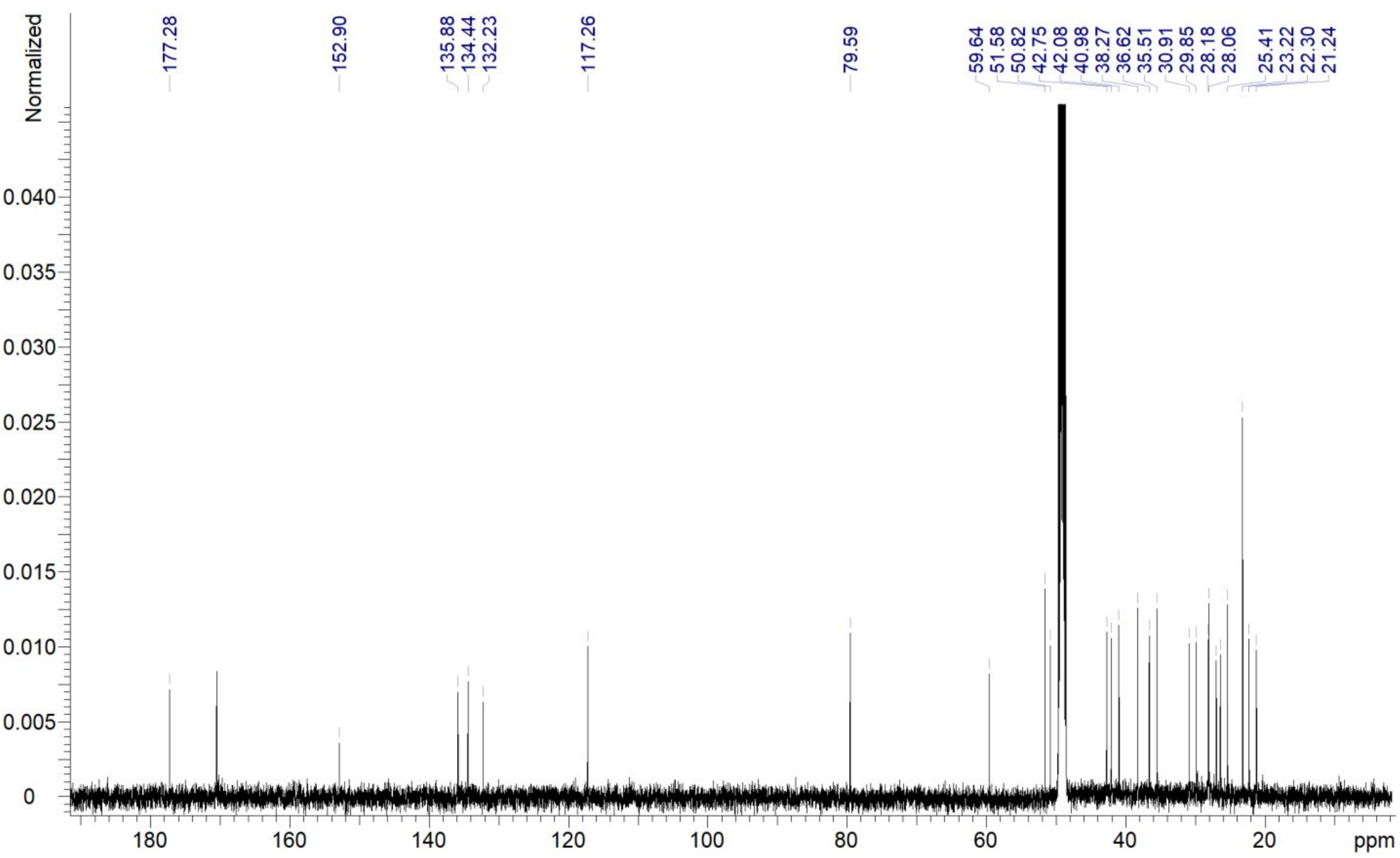

Figure S30: ^{13}C-NMR spectrum (125 MHz) of darumycin C (**4**) in CD_3OD.

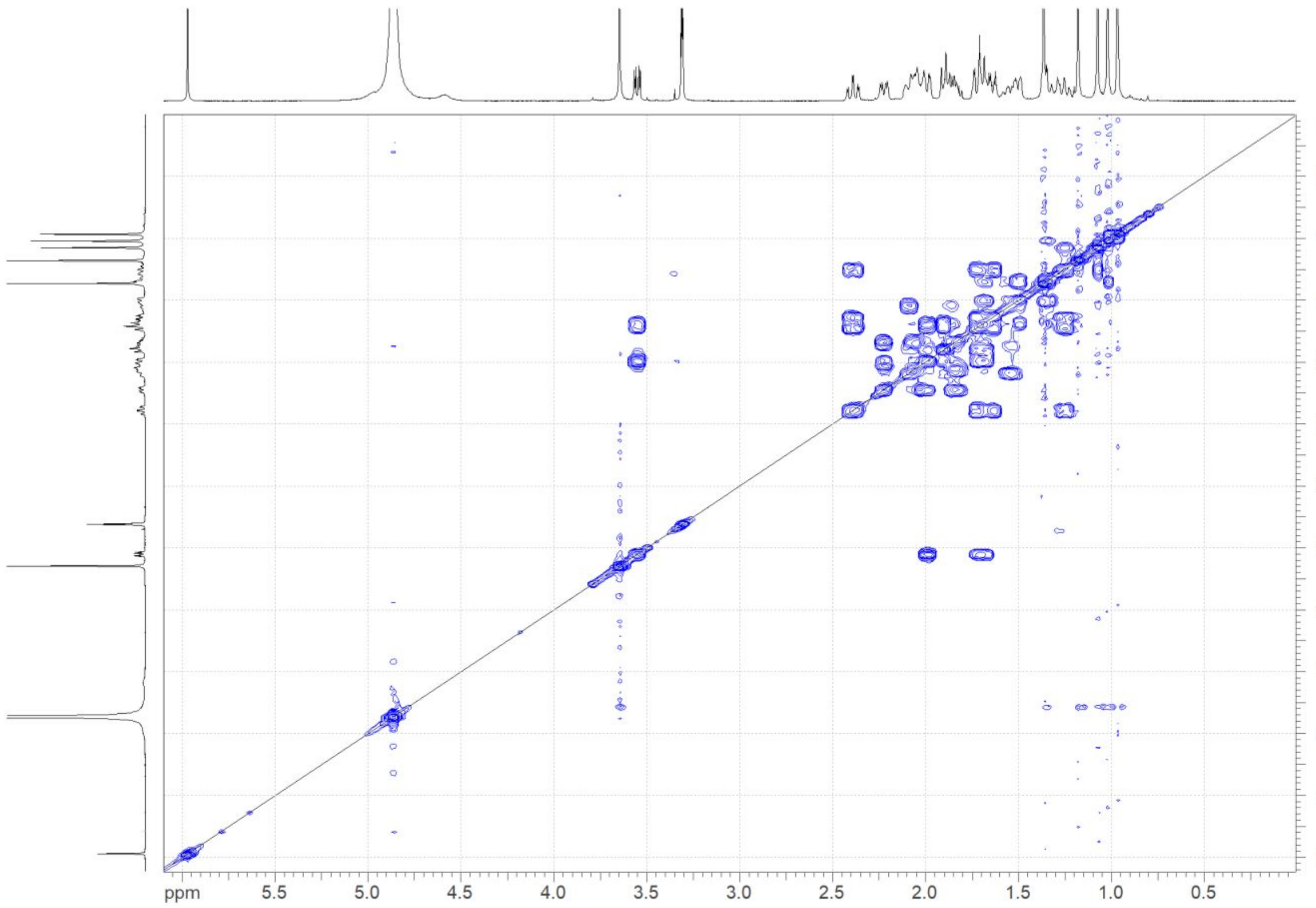

Figure S31: H-H COSY spectrum (500 MHz) of darumycin C (**4**) in CD_3OD.

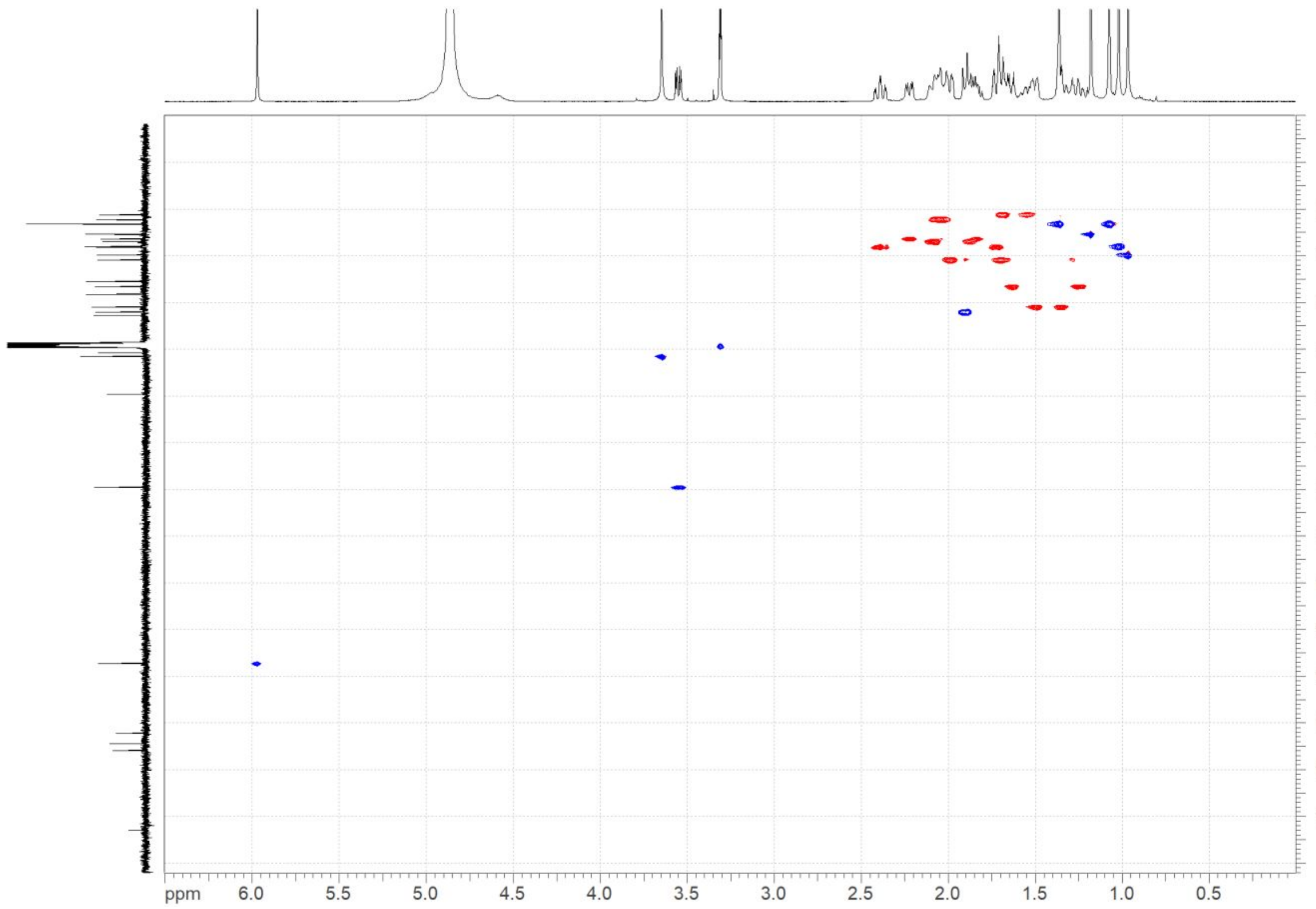

Figure S32: Edited HSQC spectrum (500 MHz) of darumycin C (**4**) in CD_3OD.

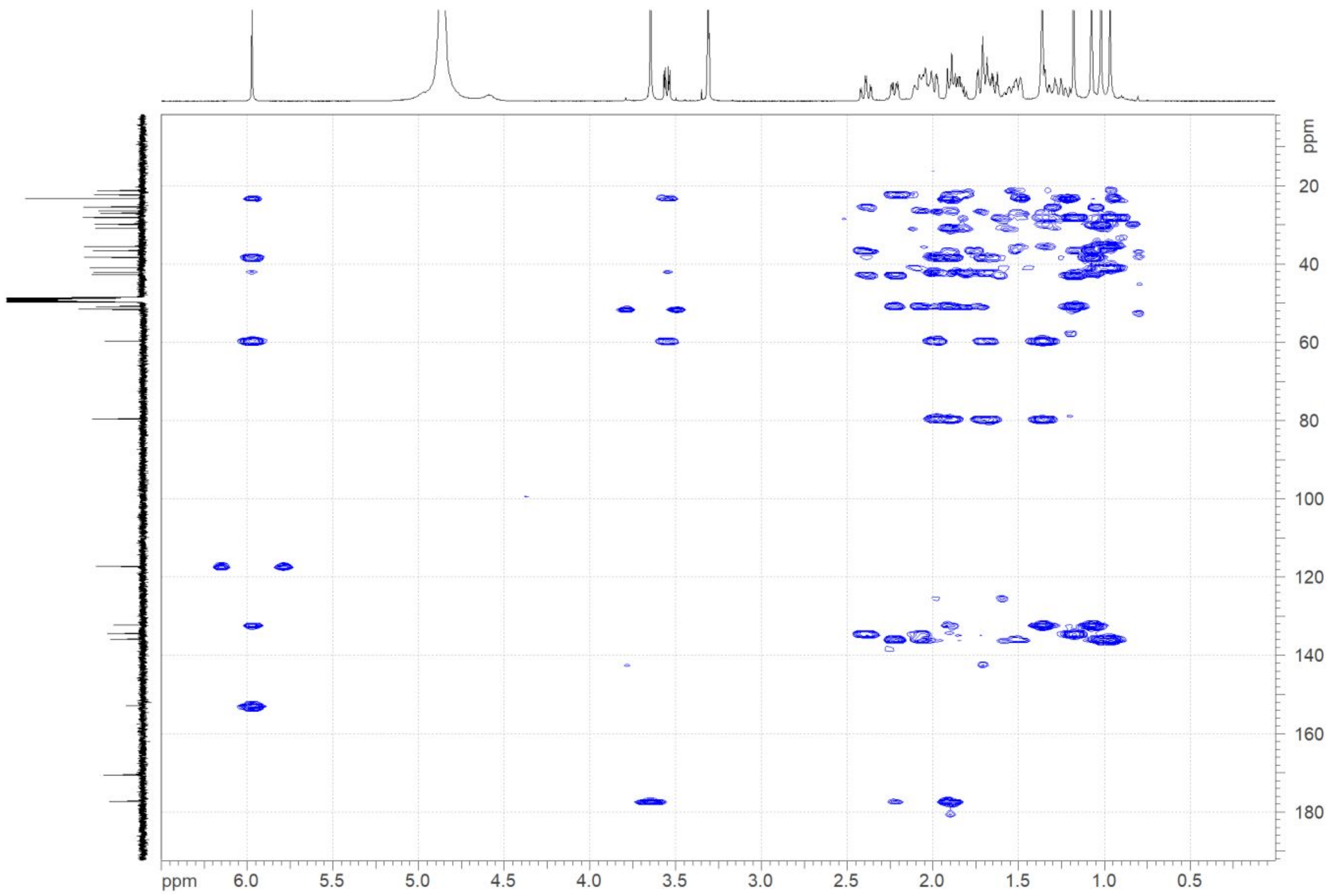

Figure S33: HMBC spectrum (500 MHz) of darumycin C (**4**) in CD_3OD.

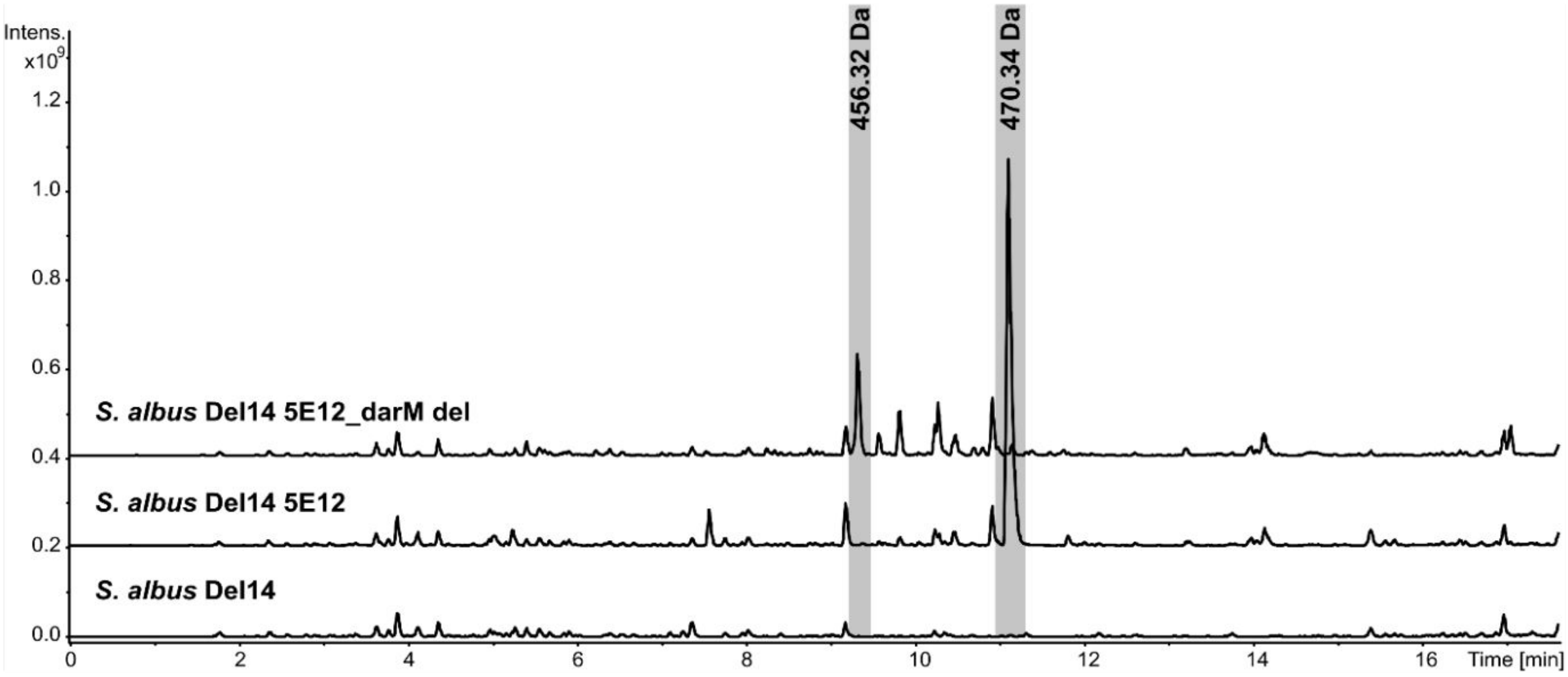

Figure S34: HPLC-MS chromatograms of the strains *S. albus* Del14, *S. albus* Del14 cos15AAmInt-5E12, *S. albus* Del14 cos15AAmInt-5E12_darM del. The peaks representing darumycin A (**1**) and darumycin D (**5**) derivative are marked by grey rectangles.

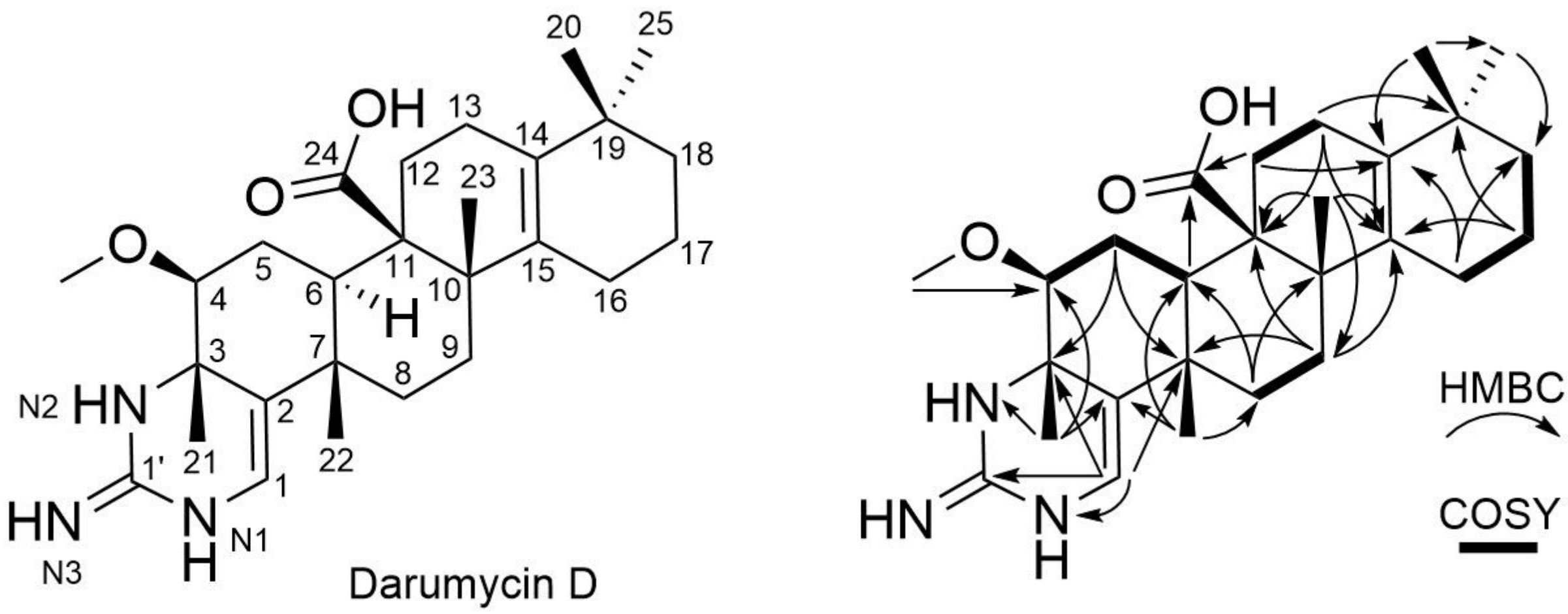

Figure S35: Structure of darumycin D (**5**).

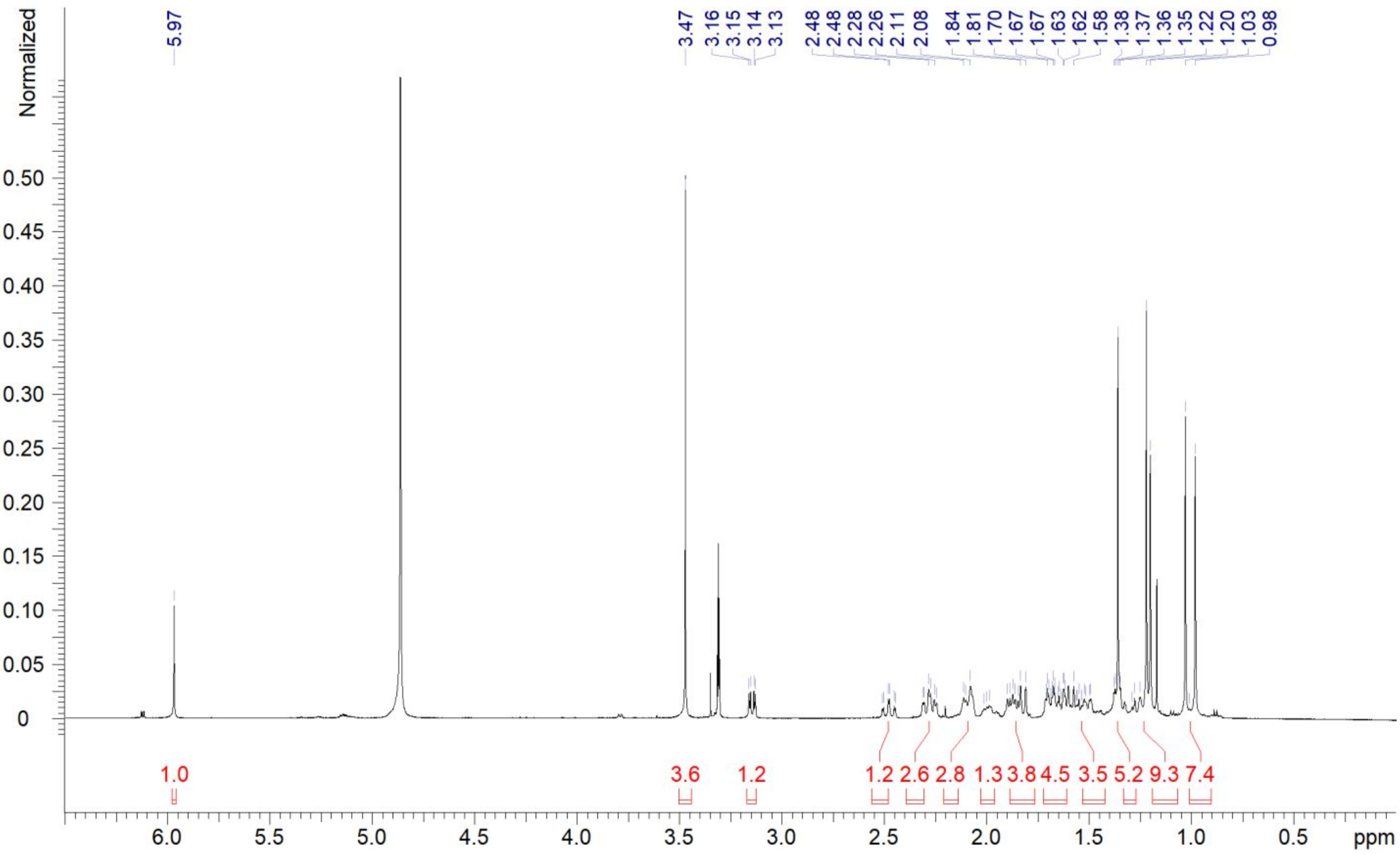

Figure S36: ^{1}H-NMR spectrum (500 MHz) of darumycin D (**5**) in CD_3OD**.**

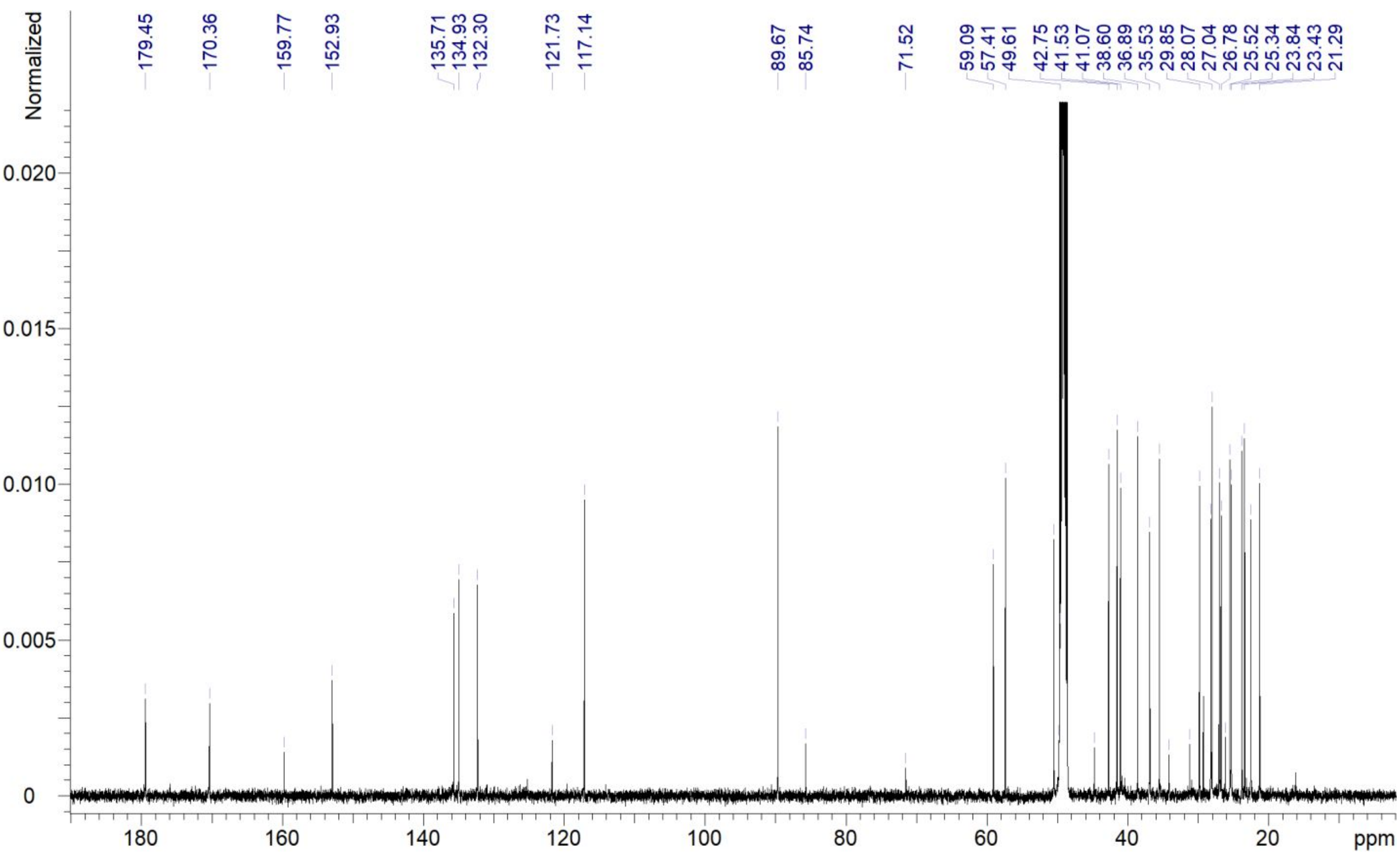

Figure S37: ^{13}C-NMR spectrum (125 MHz) of darumycin D (**5**) in CD_3OD.

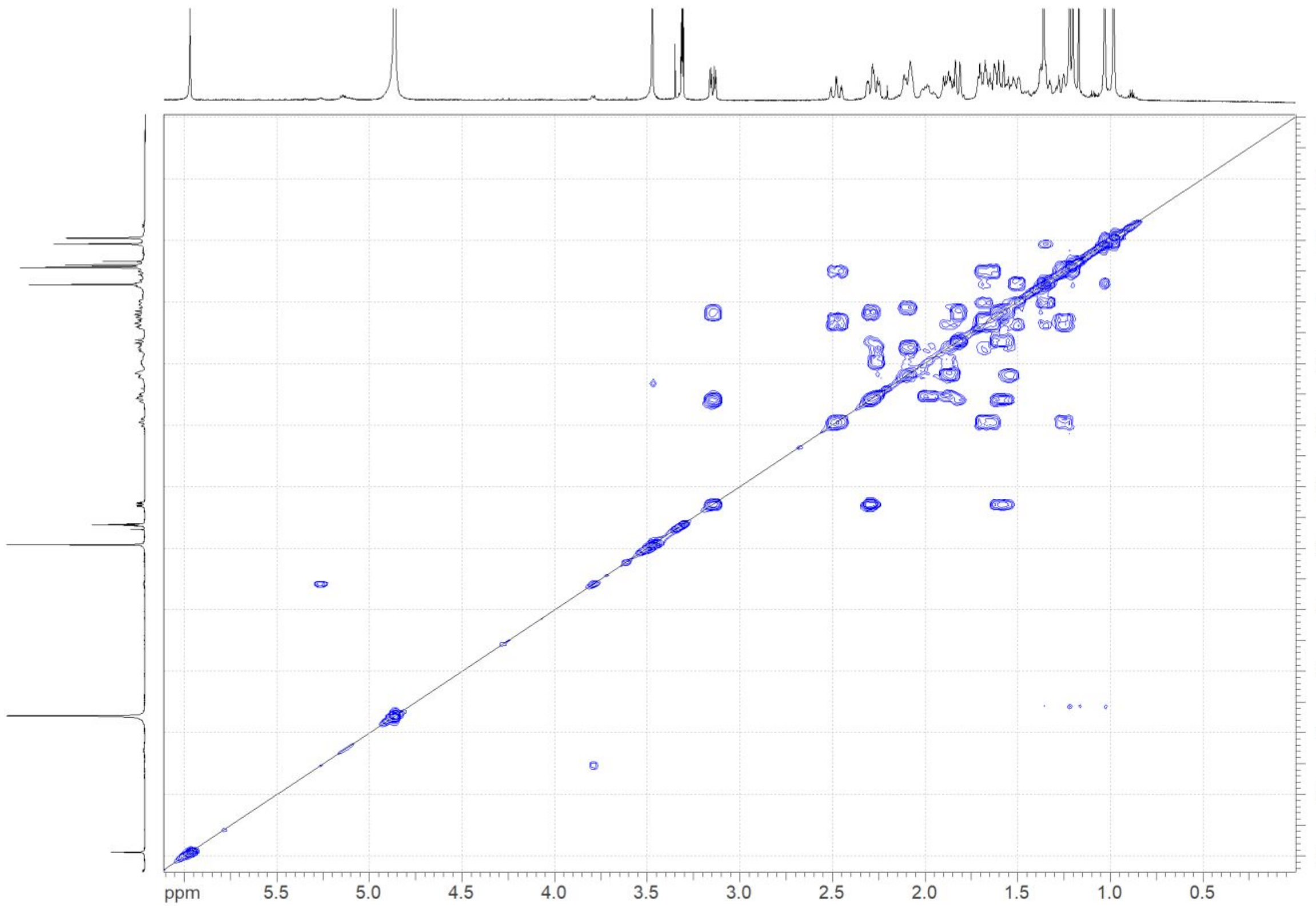

Figure S38: H-H COSY spectrum (500 MHz) of darumycin D (**5**) in CD_3OD.

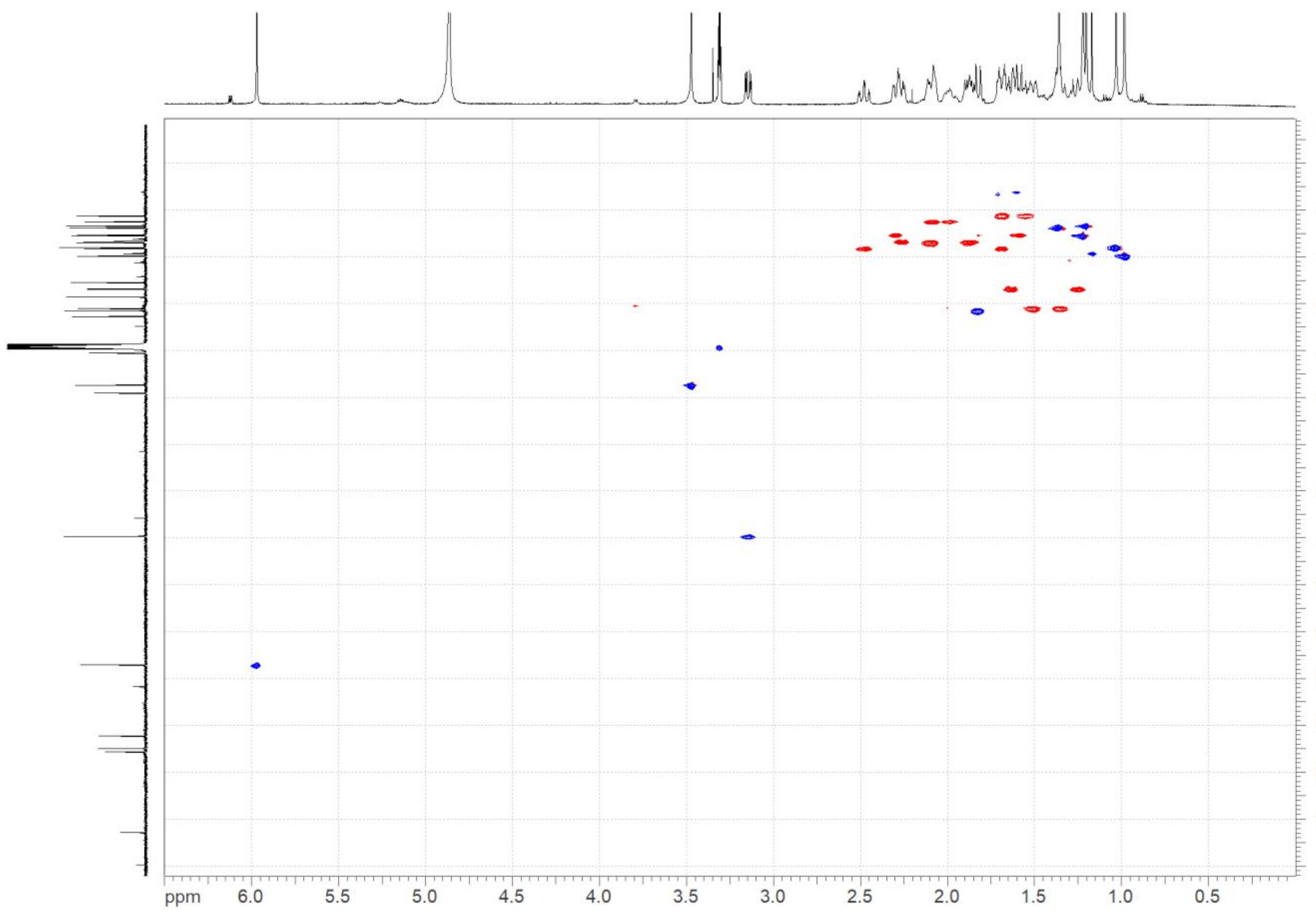

Figure S39: Edited HSQC spectrum (500 MHz) of darumycin D (**5**) in CD_3OD.

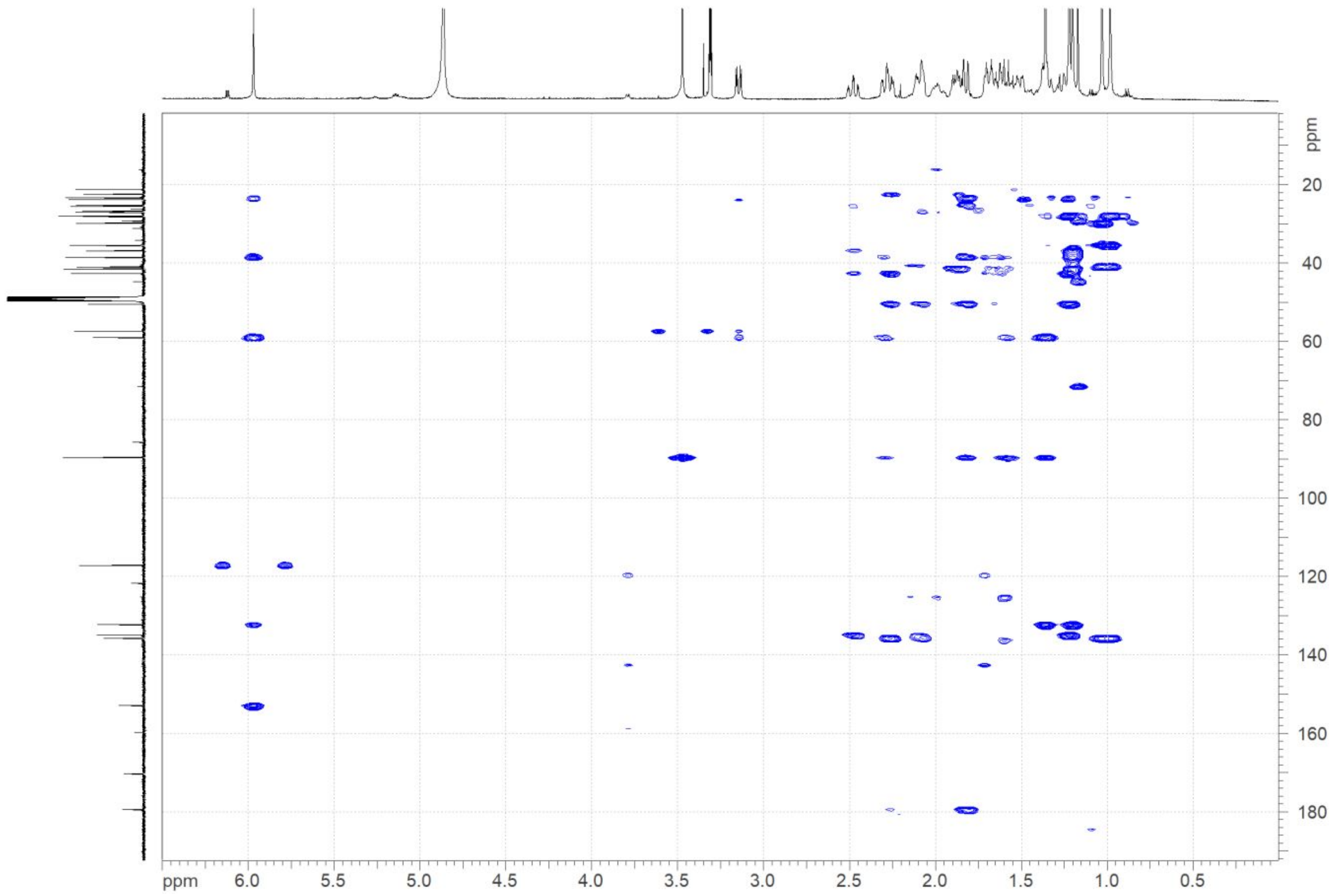

Figure S40: HMBC spectrum (500 MHz) of darumycin D (**5**) in CD_3OD.

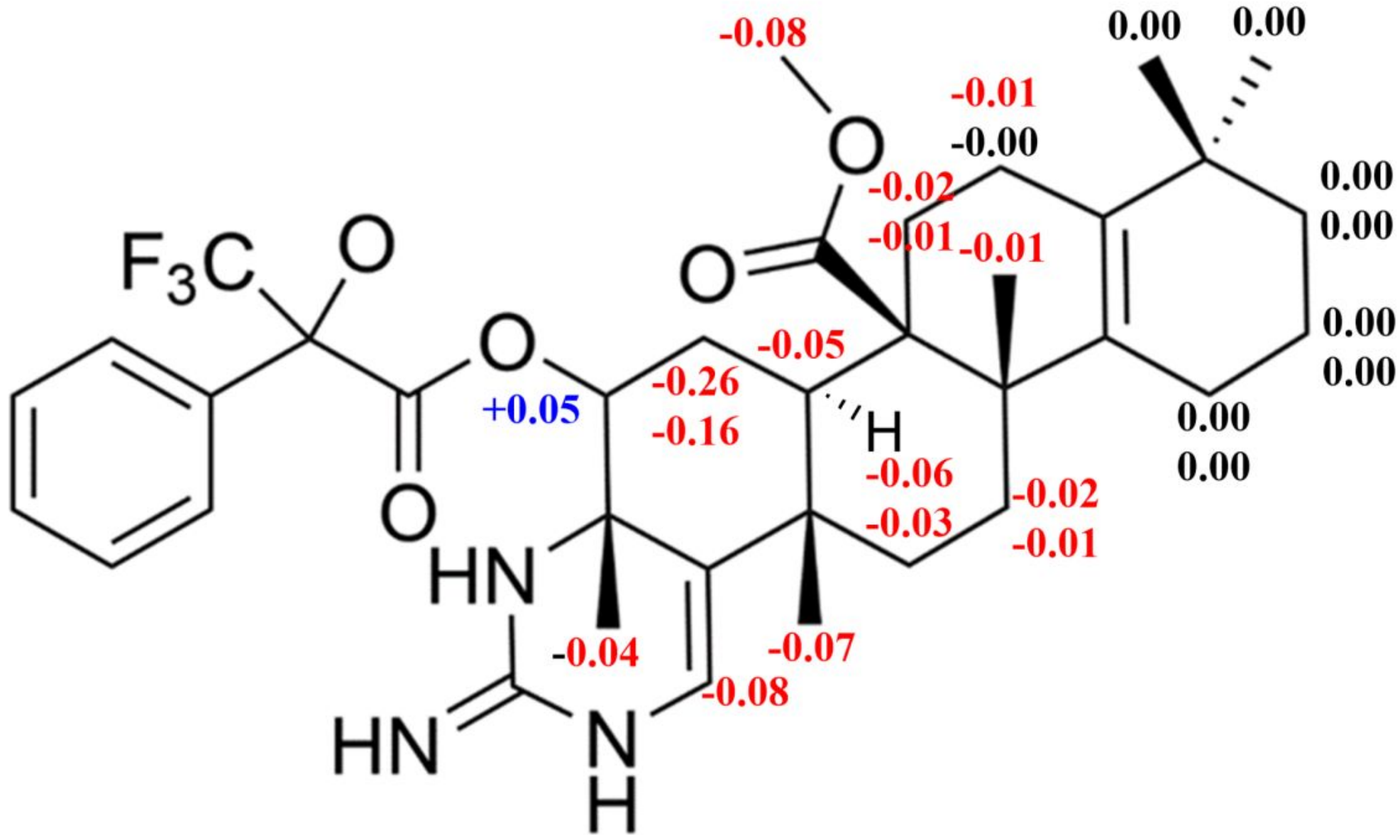

Figure S41: Structure of the darumycin C (**4**) Mosher conjugate.

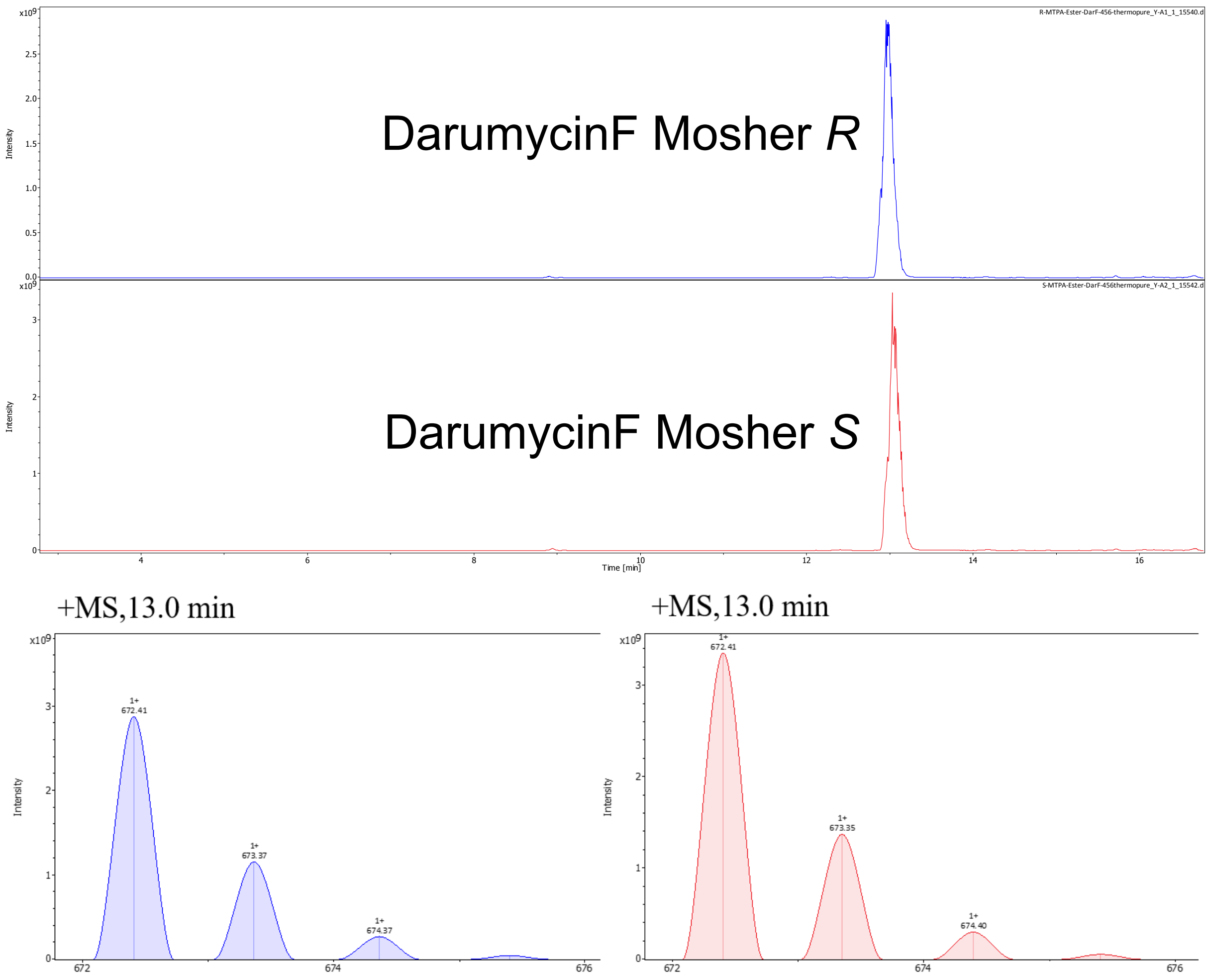

Figure S42: HPLC chromatogram and mass spectra (*m/z*) of the darumycin C (**4**) Mosher conjugates.

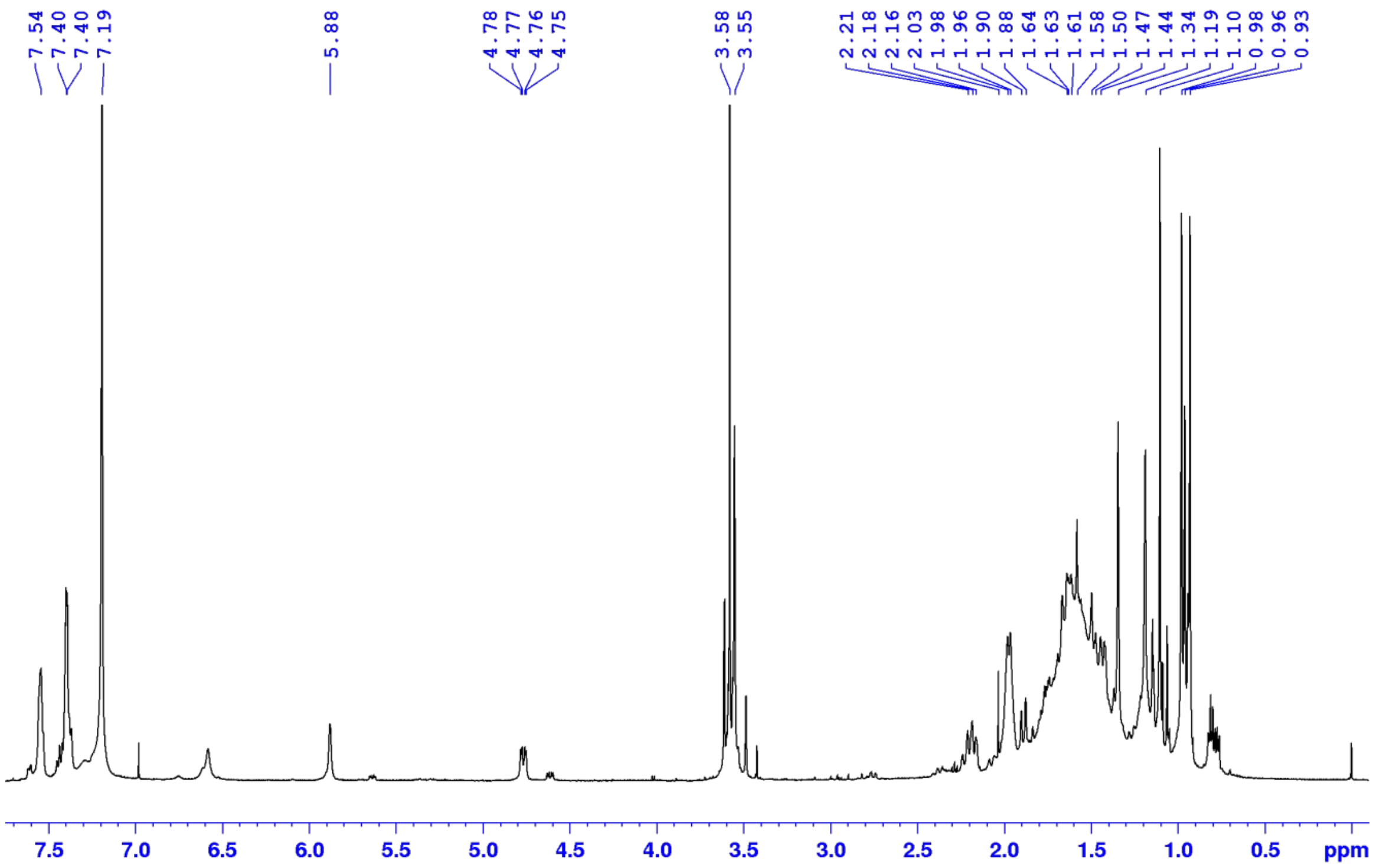

Figure S43: ^1H NMR spectrum (500MHz) of darumycin C (**4**) (*R*)-MTPA ester in $CDCl_3$.

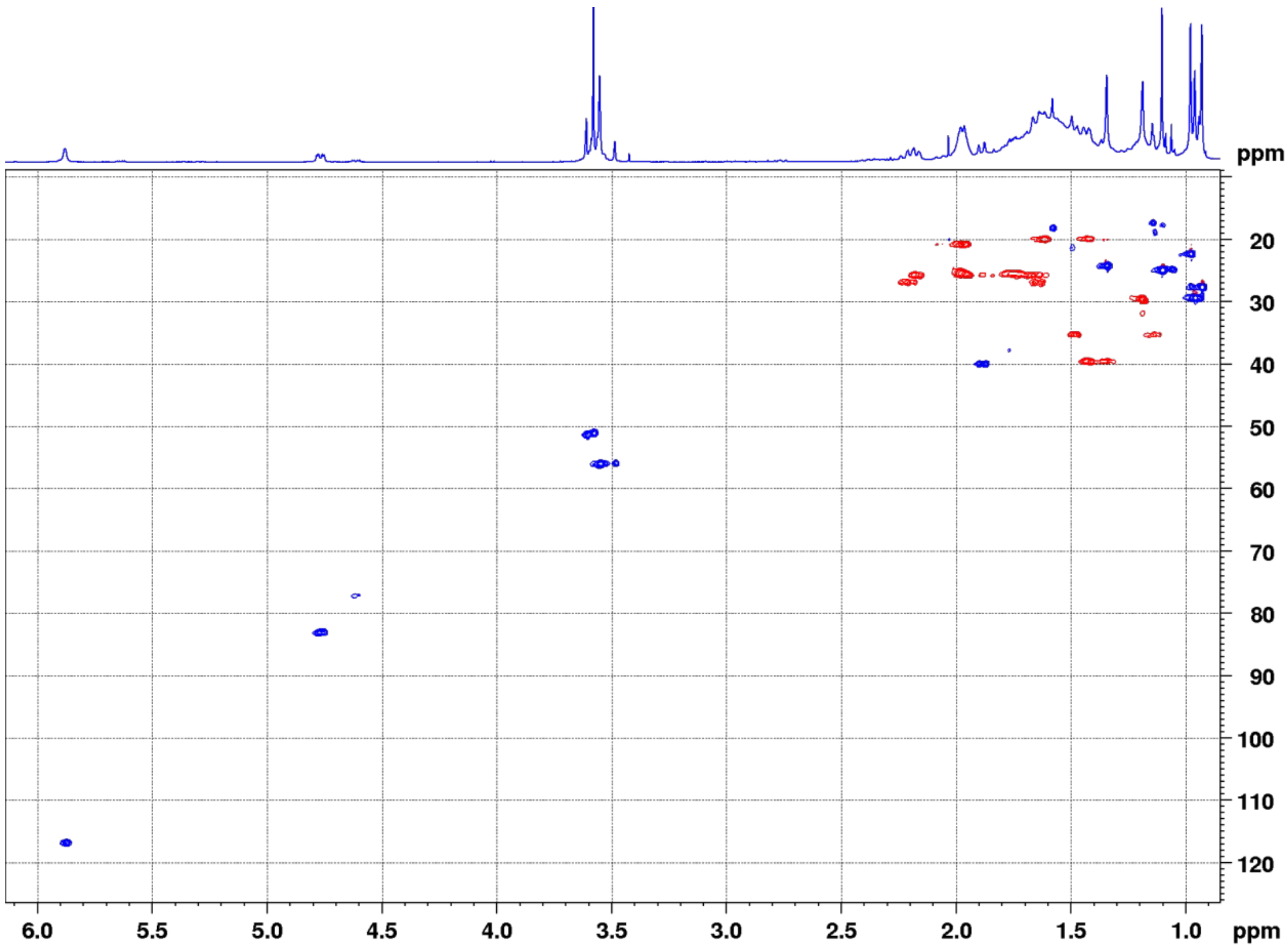

Figure S44: Edited HSQC spectrum (500 MHz) of darumycin C (**4**) (*R*)-MTPA ester in $CDCl_3$.

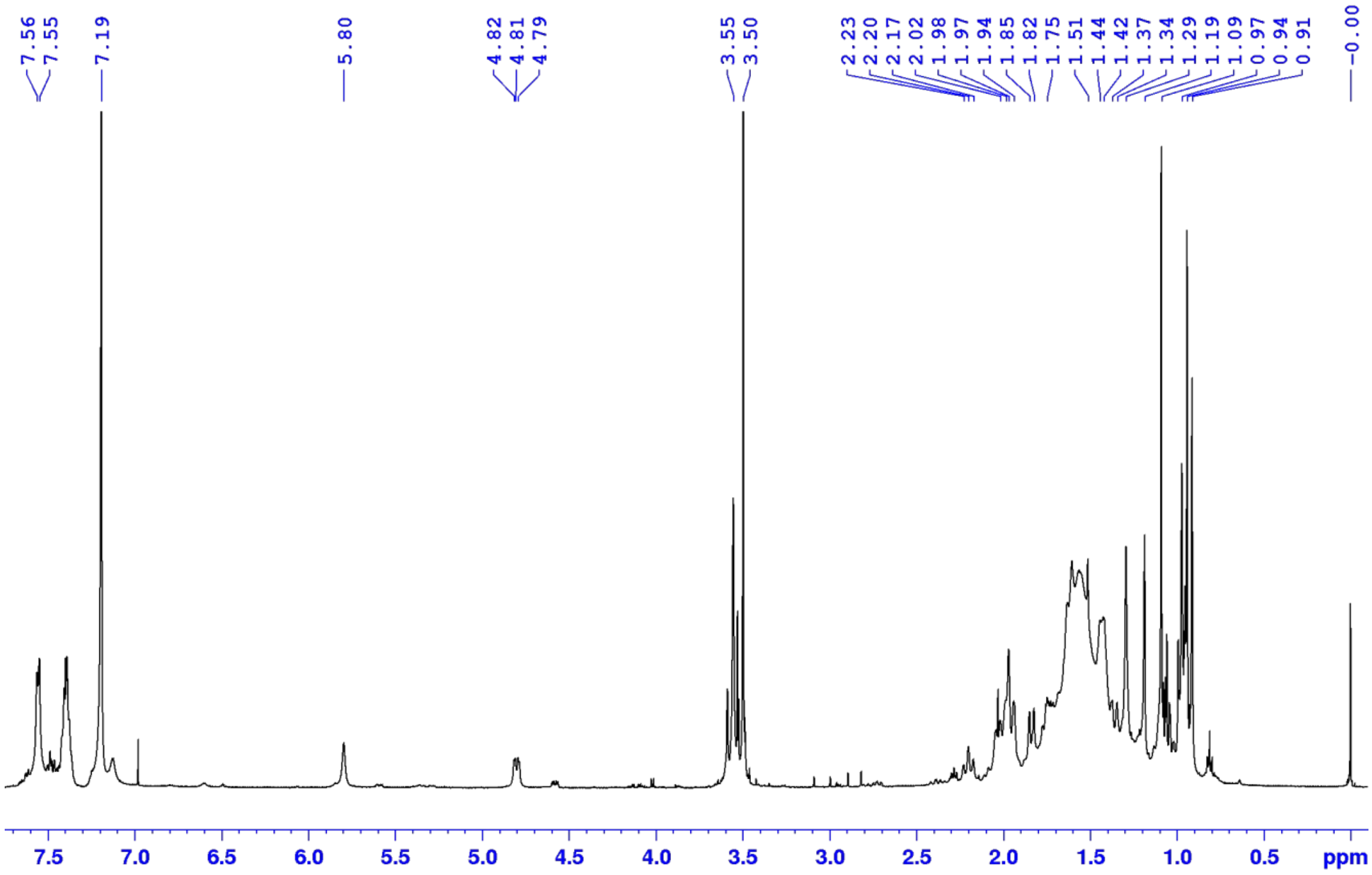

Figure S45: ^{1}H NMR spectrum (500MHz) of darumycin C (**4**) (*S*)-MTPA ester in $CDCl_3$.

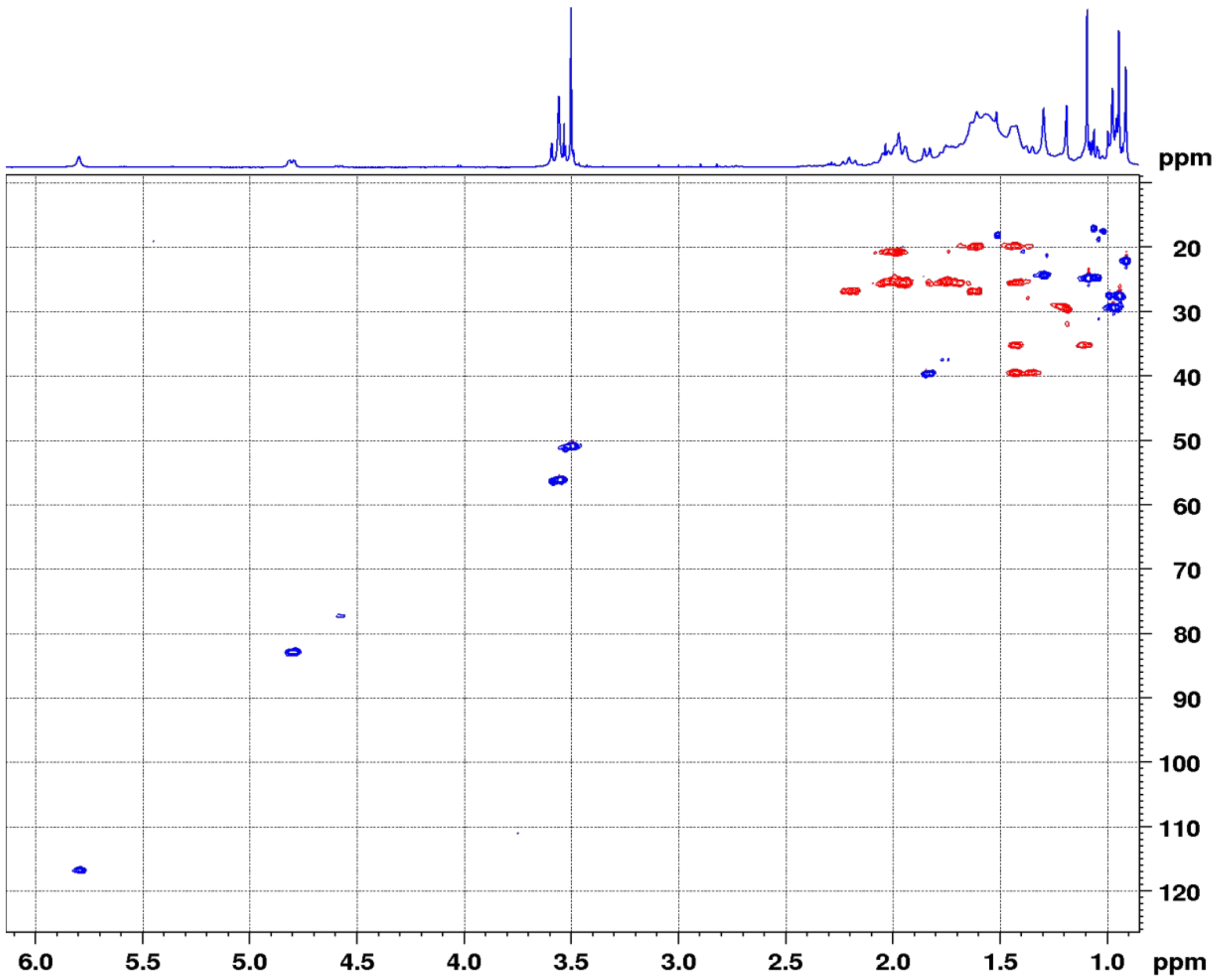

Figure S46: Edited HSQC spectrum (500 MHz) of darumycin C (**4**) (S)-MTPA ester in $CDCl_3$.

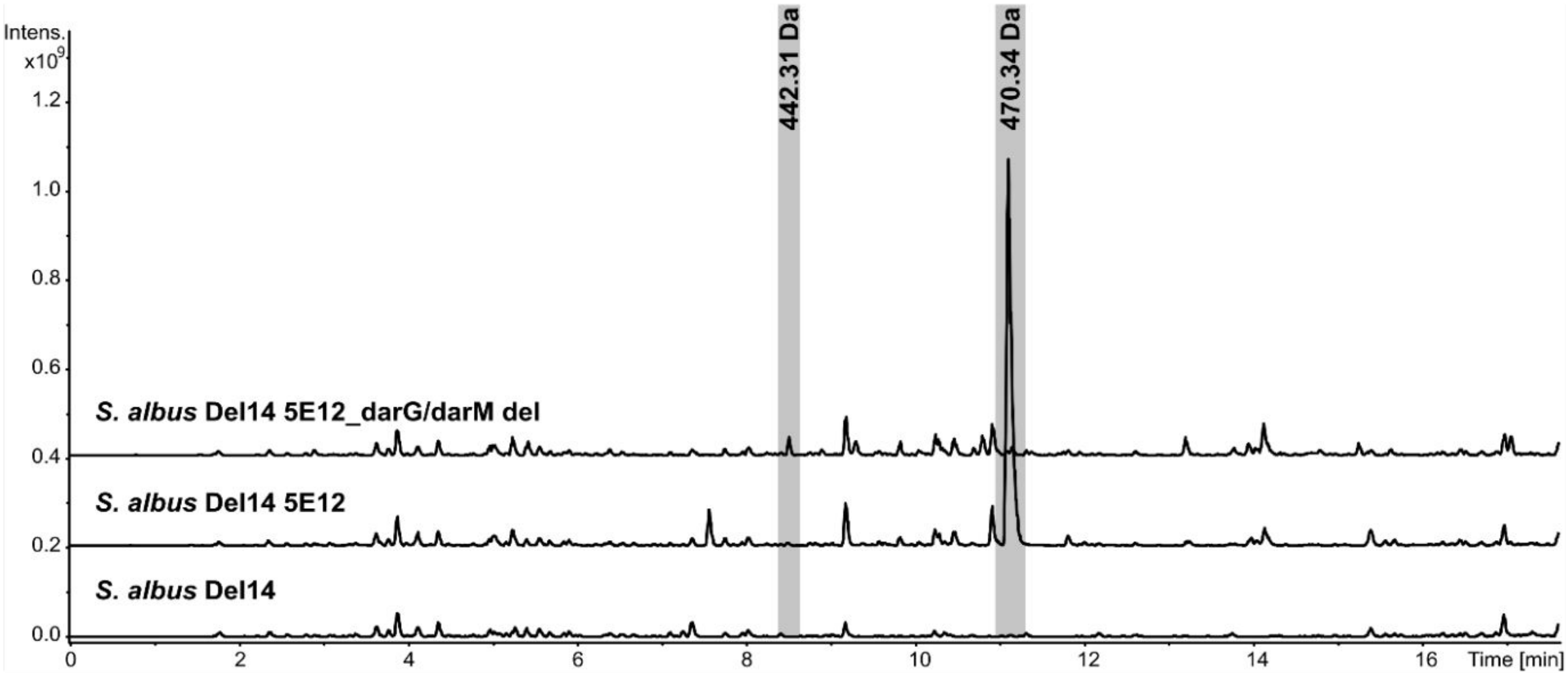

Figure S47: HPLC-MS chromatograms of the strains *S. albus* Del14, *S. albus* Del14 cos15AAmInt-5E12, *S. albus* Del14 cos15AAmInt-5E12_darG/darM del. The peaks representing darumycin A (**1**) and darumycin E (**6**) derivatives are marked by grey rectangles.

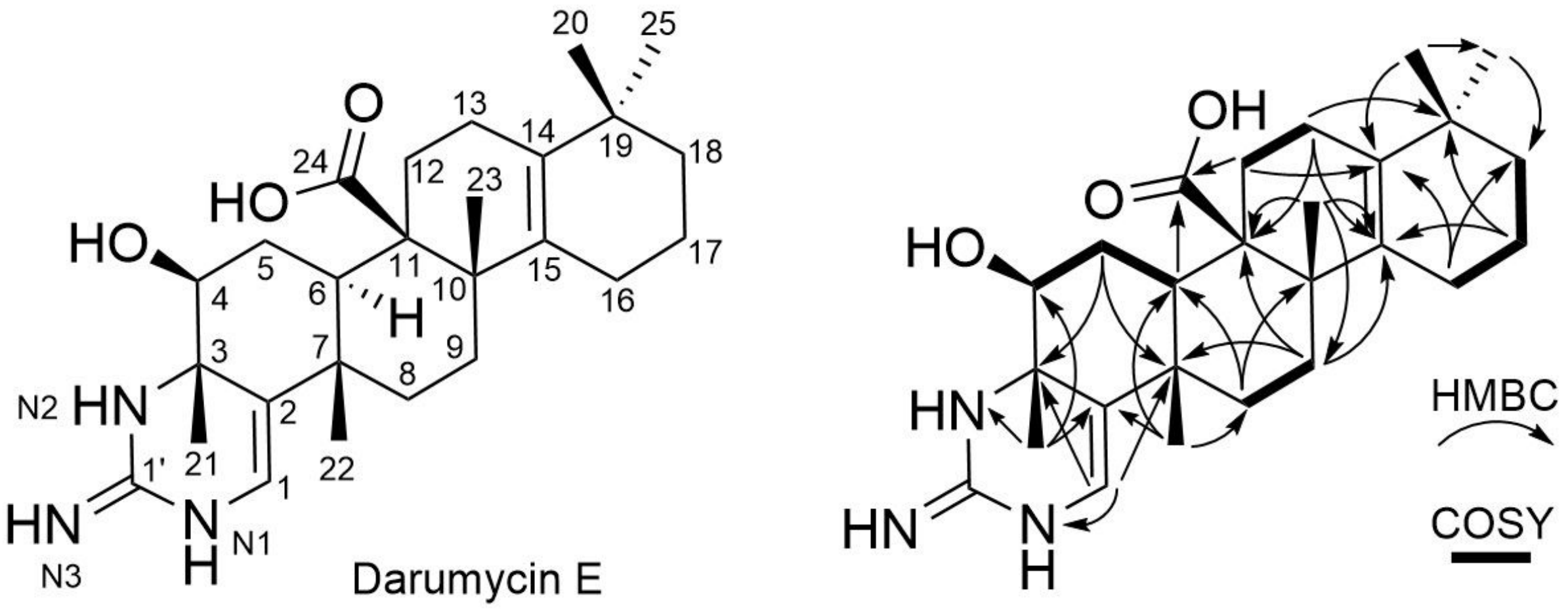

Figure S48: Structure of darumycin E (**6**).

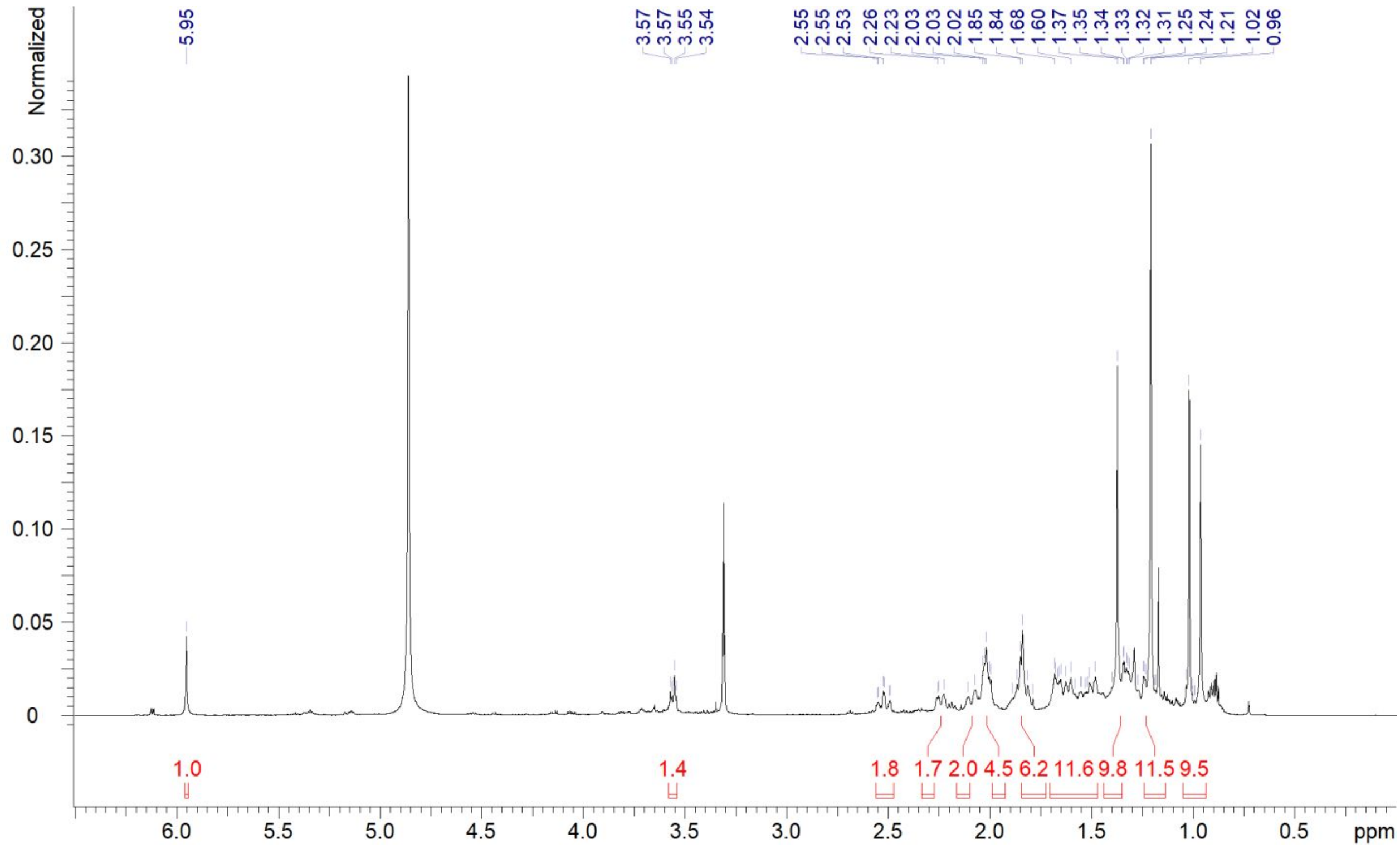

Figure S49: ^{1}H-NMR spectrum (500 MHz) of darumycin E (**6**) in CD_3OD**.**

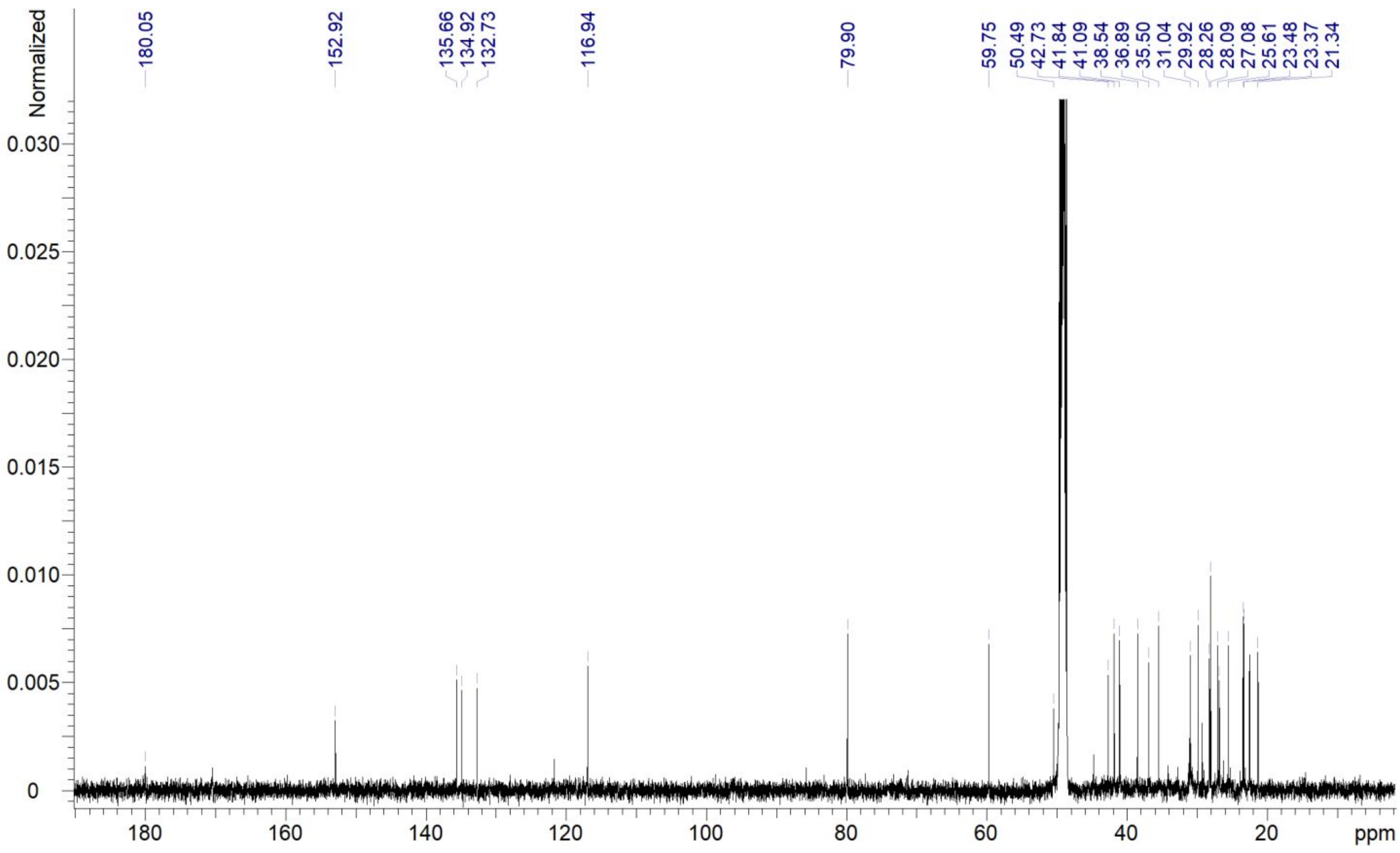

Figure S50: ^{13}C-NMR spectrum (125 MHz) of darumycin E (**6**) in CD_3OD.

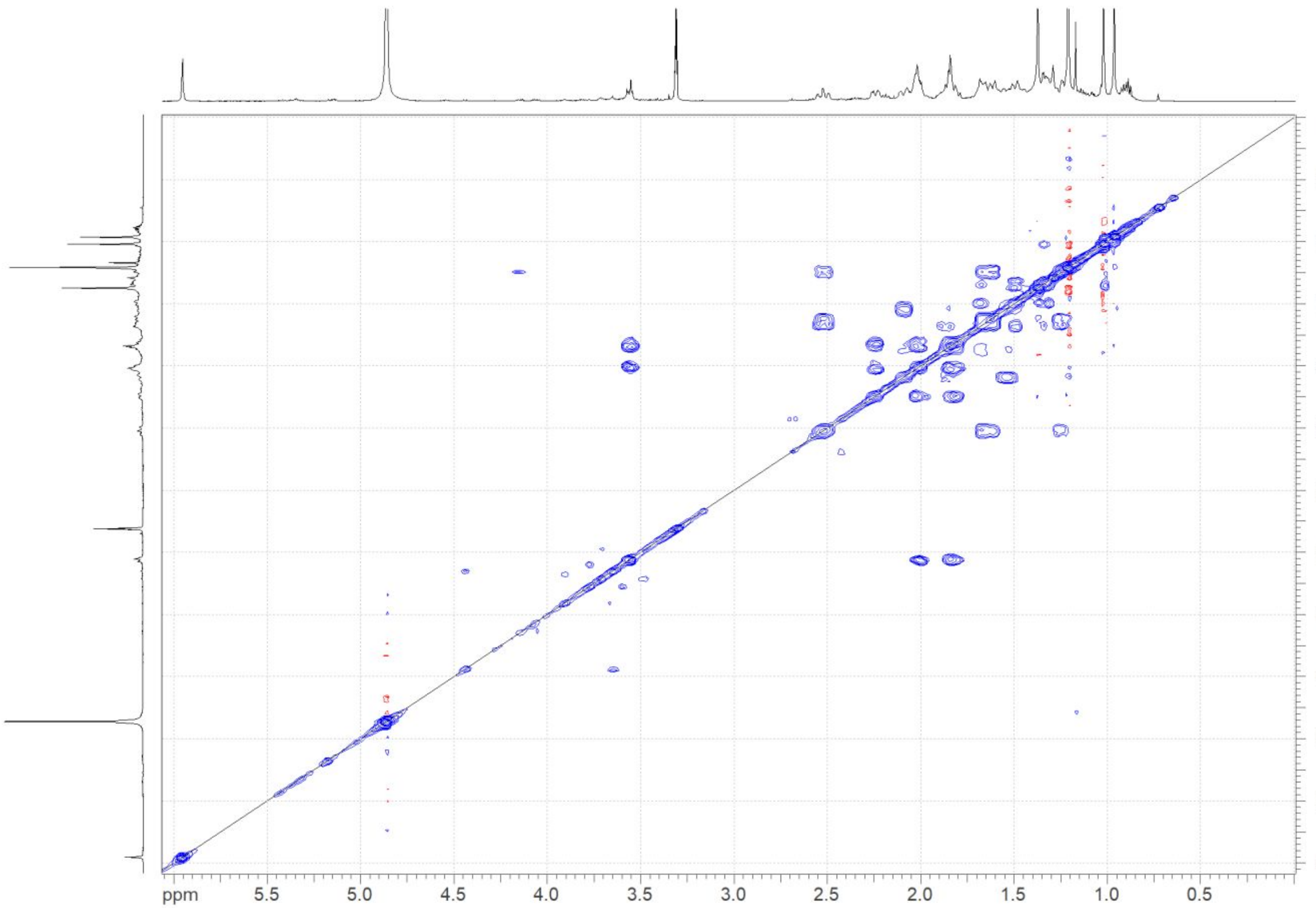

Figure S51: H-H COSY spectrum (500 MHz) of darumycin E (**6**) in CD_3OD.

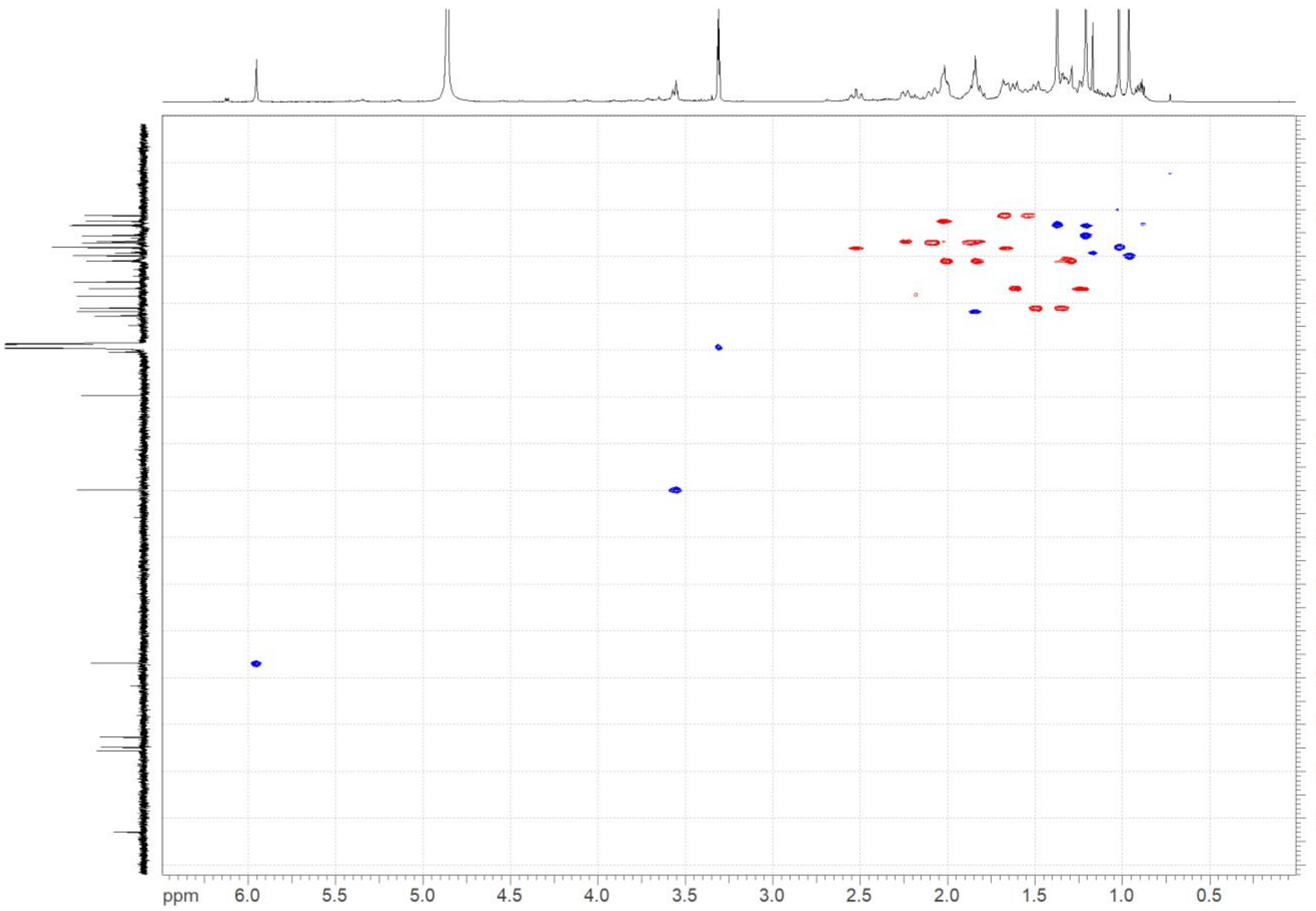

Figure S52: Edited HSQC spectrum (500 MHz) of darumycin E (**6**) in CD_3OD.

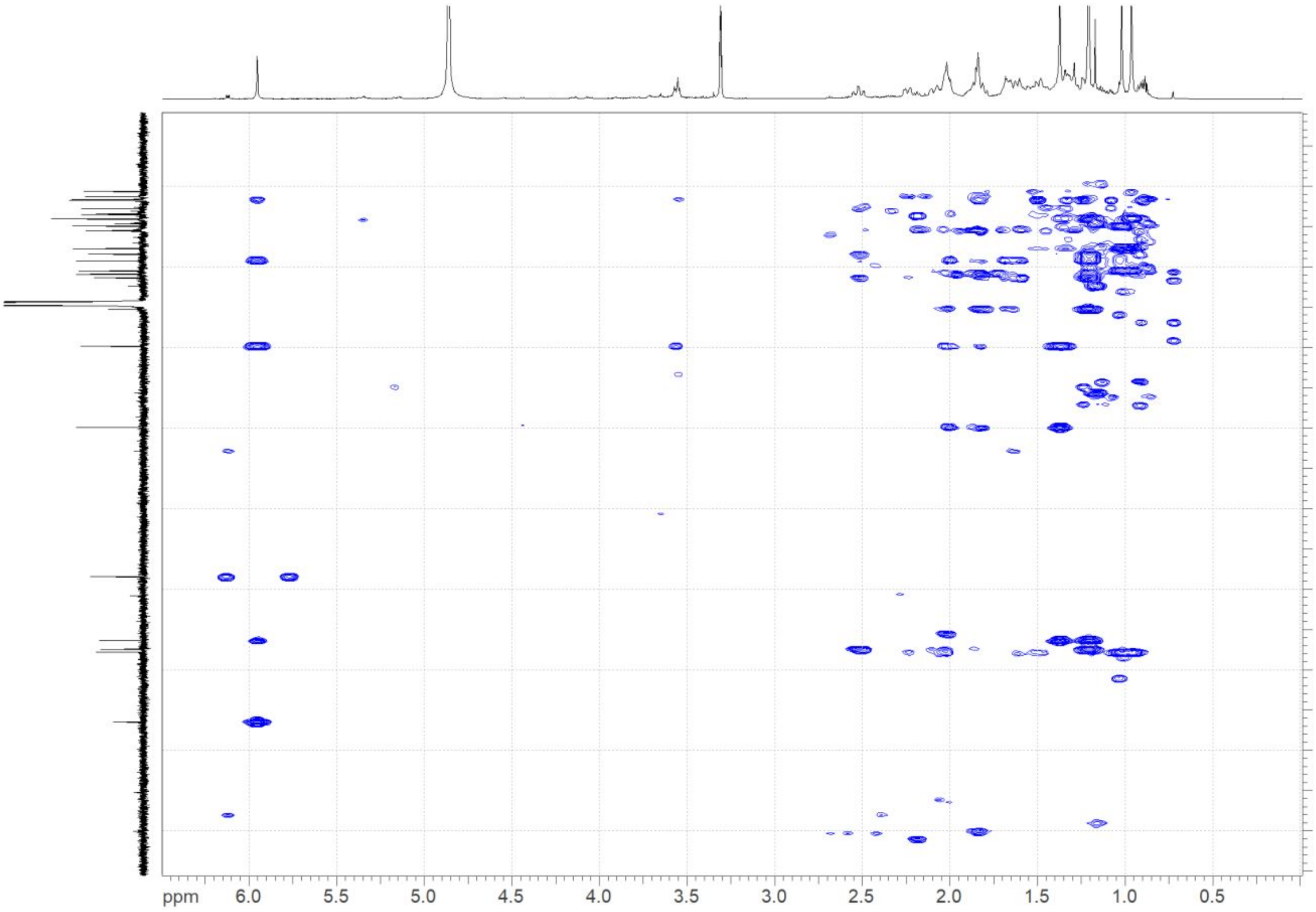

Figure S53: HMBC spectrum (500 MHz) of darumycin E (**6**) in CD_3OD.

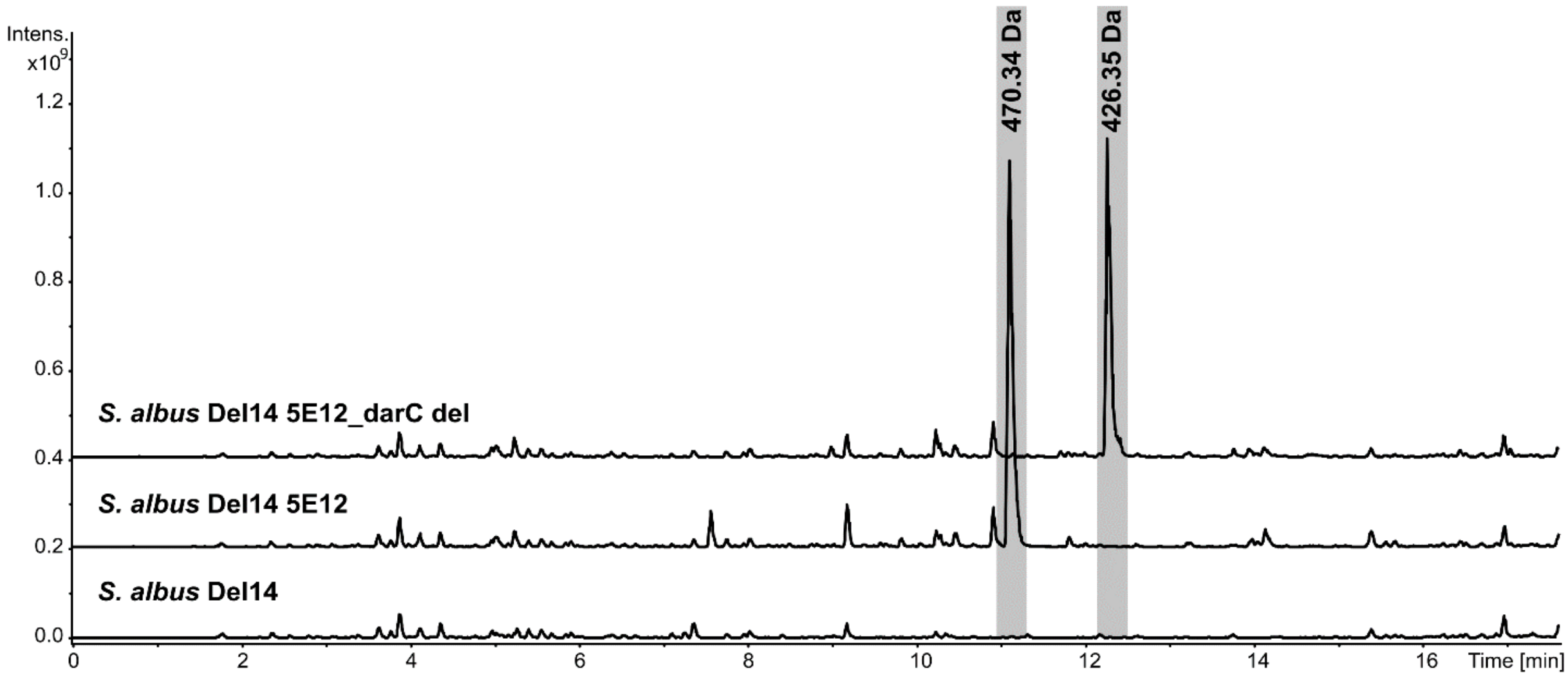

Figure S54: HPLC-MS chromatograms of the strains *S. albus* Del14, *S. albus* Del14 cos15AAmInt-5E12, *S. albus* Del14 cos15AAmInt-5E12_darC del. The peaks representing darumycin A (**1**) and darumycin F (**7**) derivative are marked by grey rectangles.

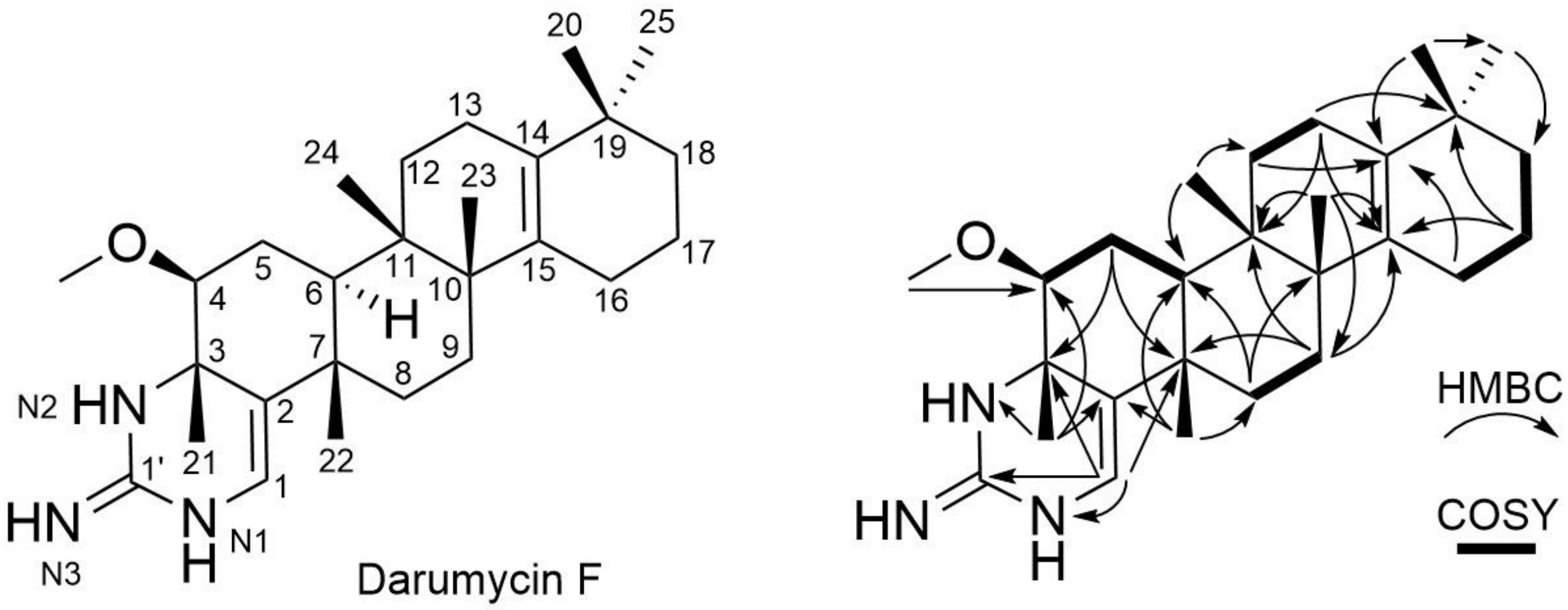

Figure S55: Structure of darumycin F (**7**).

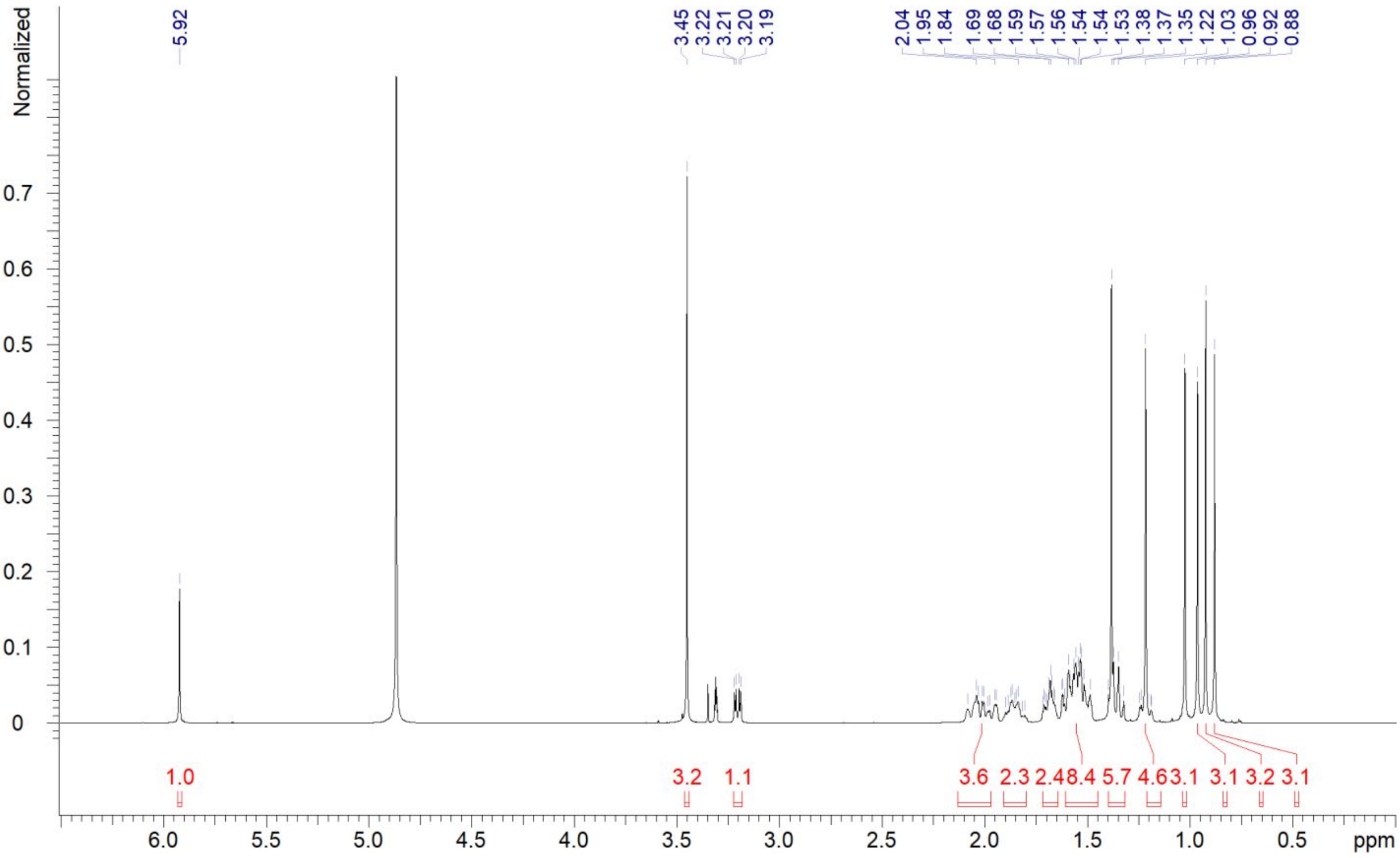

Figure S56: ^{1}H-NMR spectrum (500 MHz) of darumycin F (**7**) in CD_3OD.

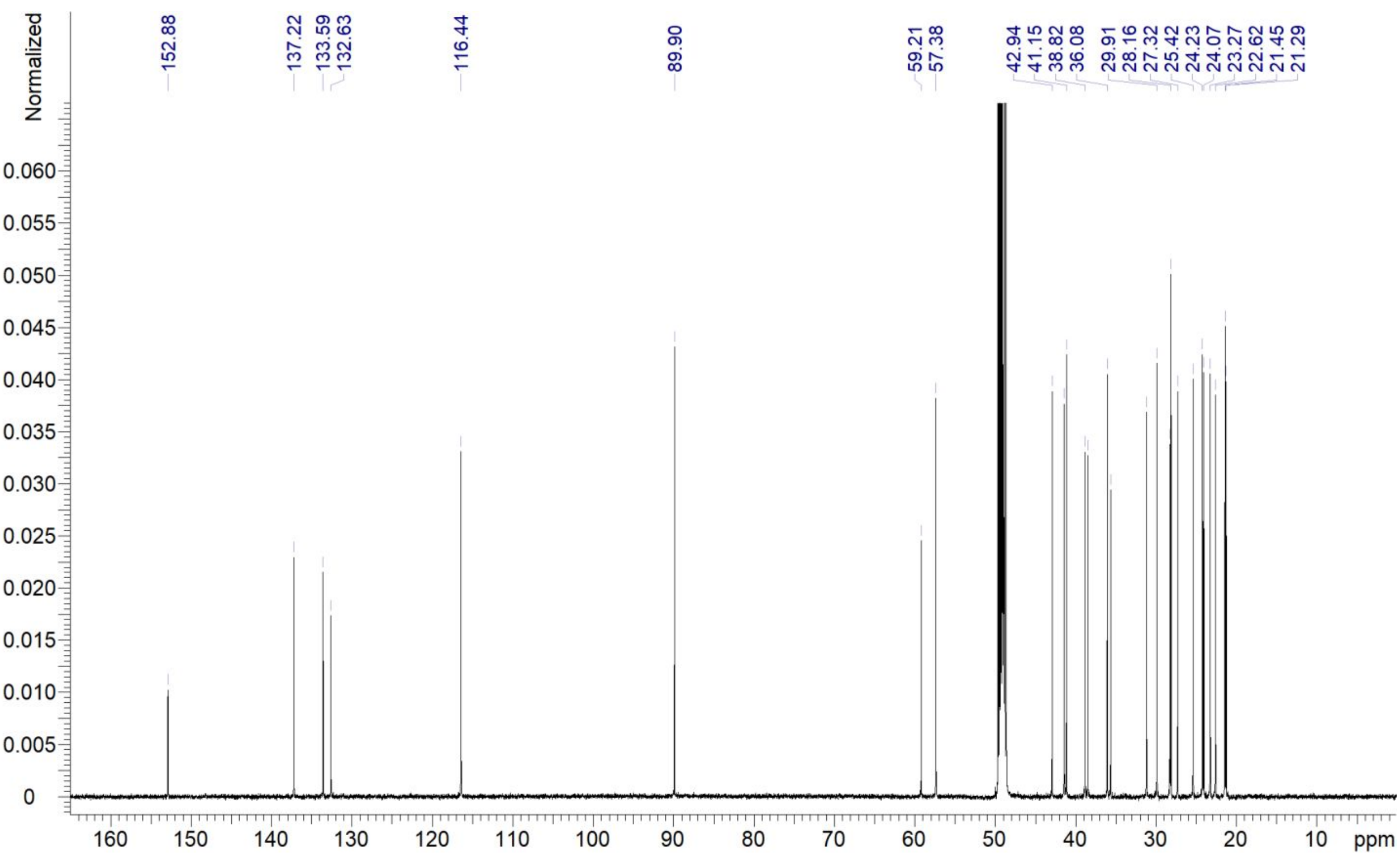

Figure S57: ^{13}C-NMR spectrum (125 MHz) of darumycin F (**7**) in CD_3OD.

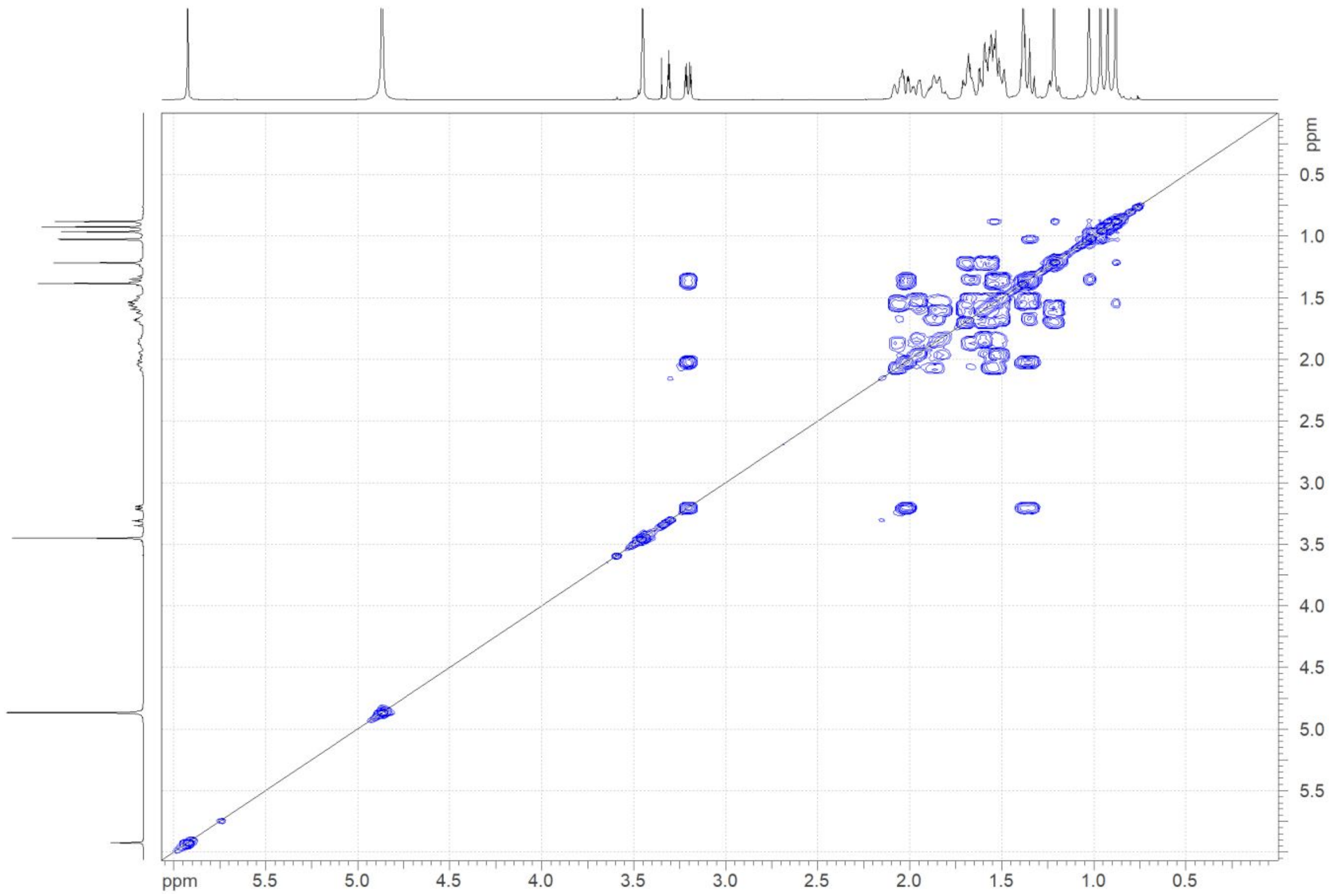

Figure S58: H-H COSY spectrum (500 MHz) of darumycin F (**7**) in CD_3OD.

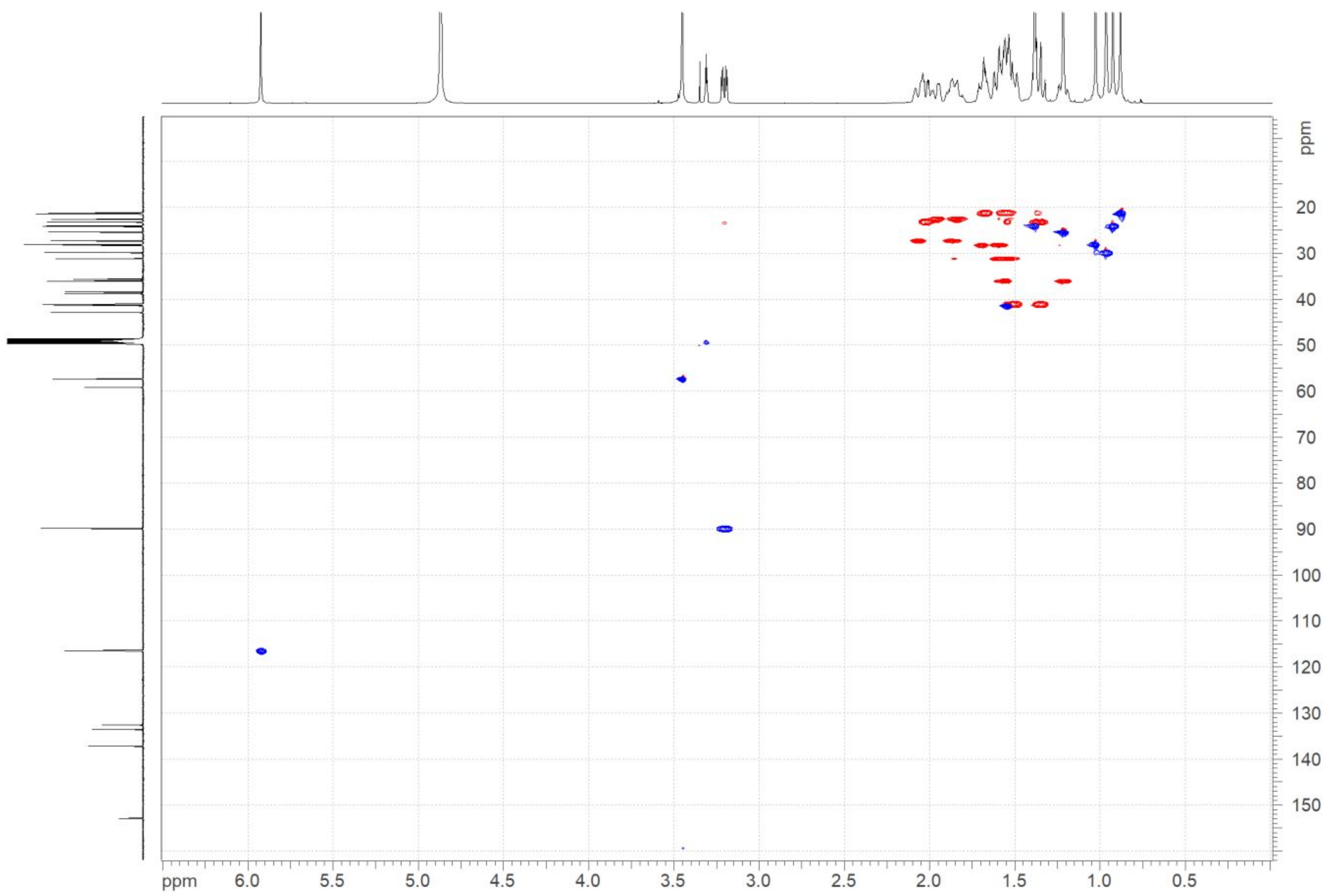

Figure S59: Edited HSQC spectrum (500 MHz) of darumycin F (**7**) in CD_3OD.

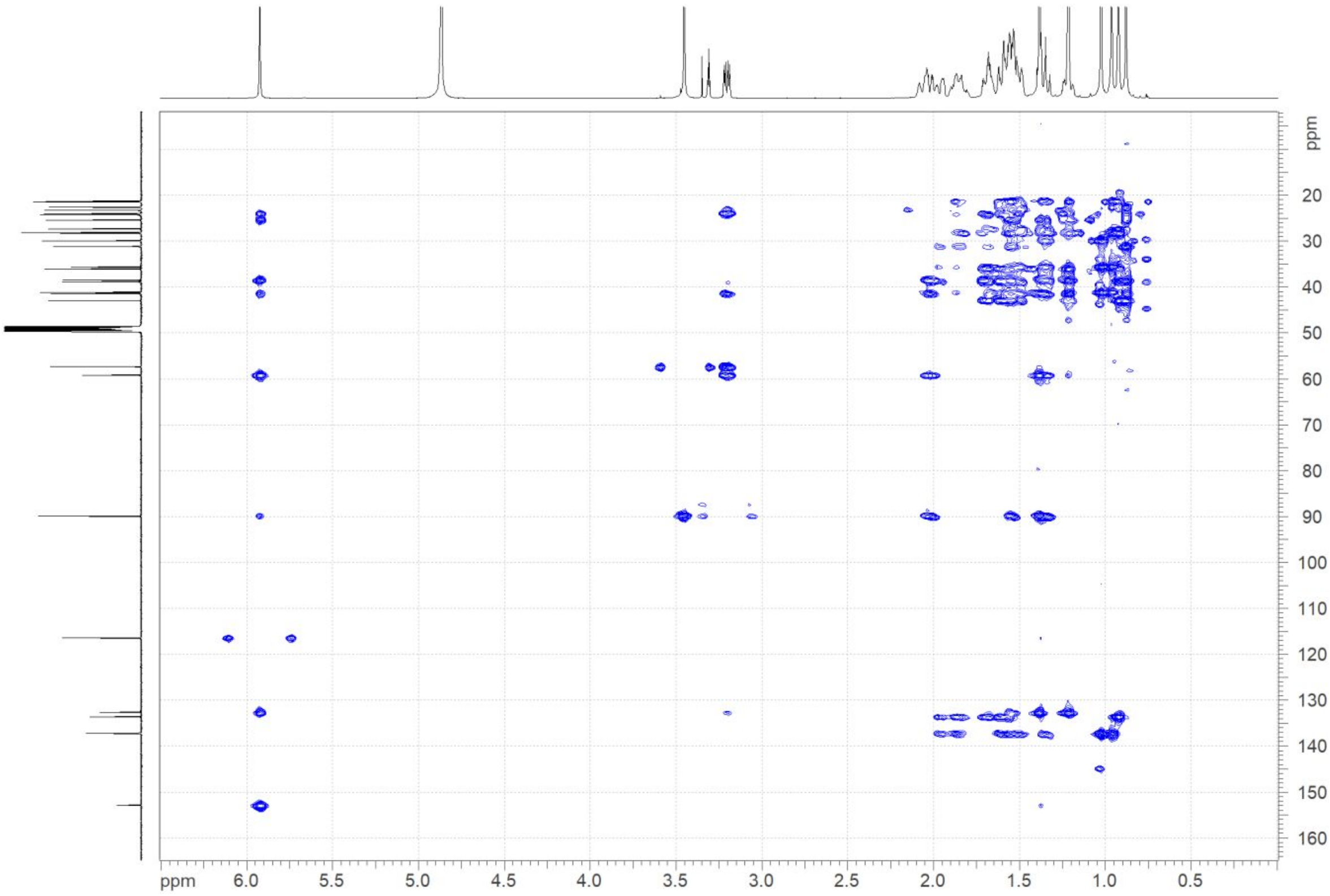

Figure S60: HMBC spectrum (500 MHz) of darumycin F (**7**) in CD_3OD.

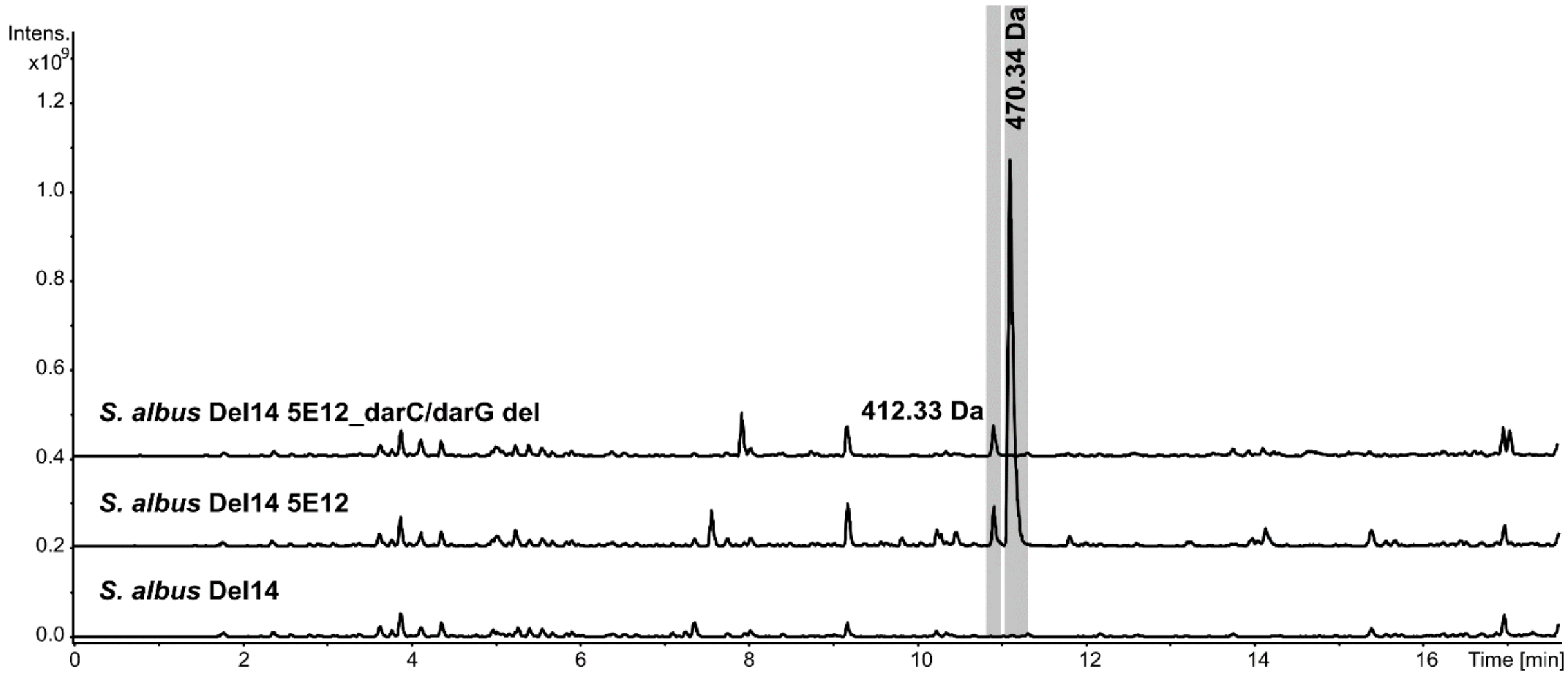

Figure S61: HPLC-MS chromatograms of the strains *S. albus* Del14, *S. albus* Del14 cos15AAmInt-5E12, *S. albus* Del14 cos15AAmInt-5E12_darC/darG del. The peaks representing darumycin A (**1**) and darumycin G (**8**) derivative are marked by grey rectangles.

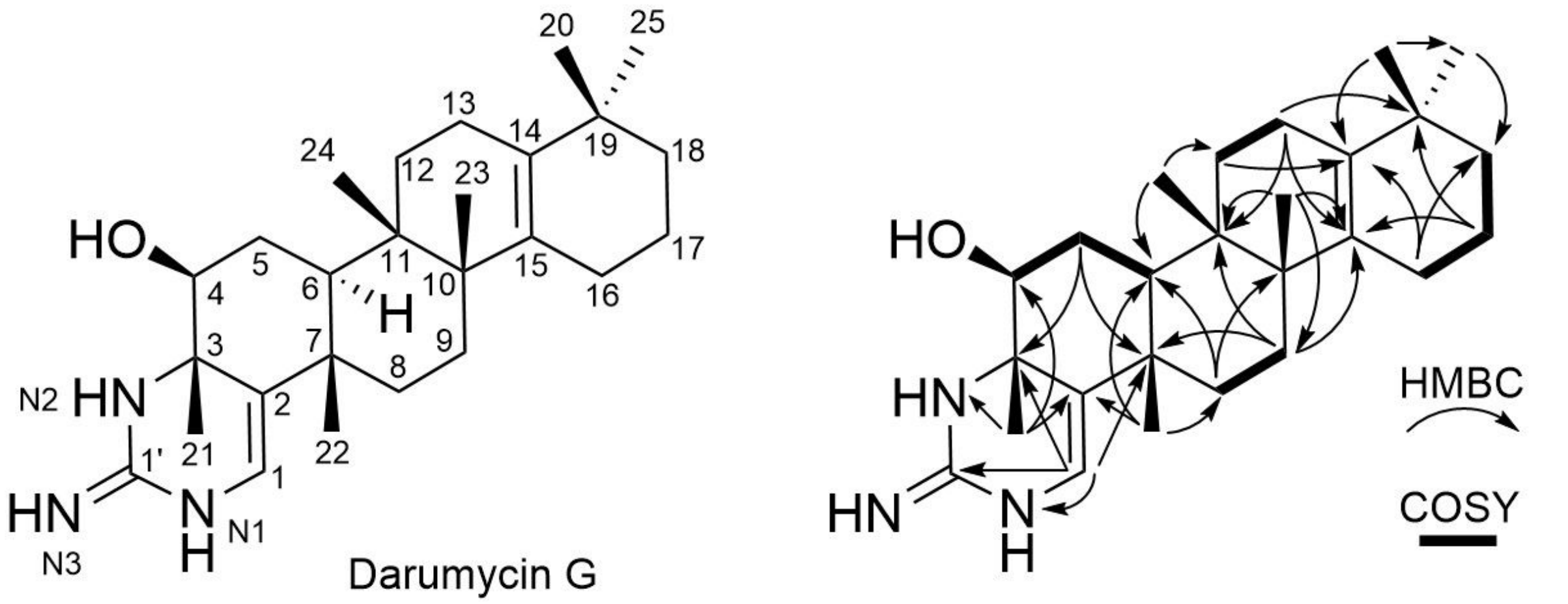

Figure S62: Structure of darumycin G (**8**).

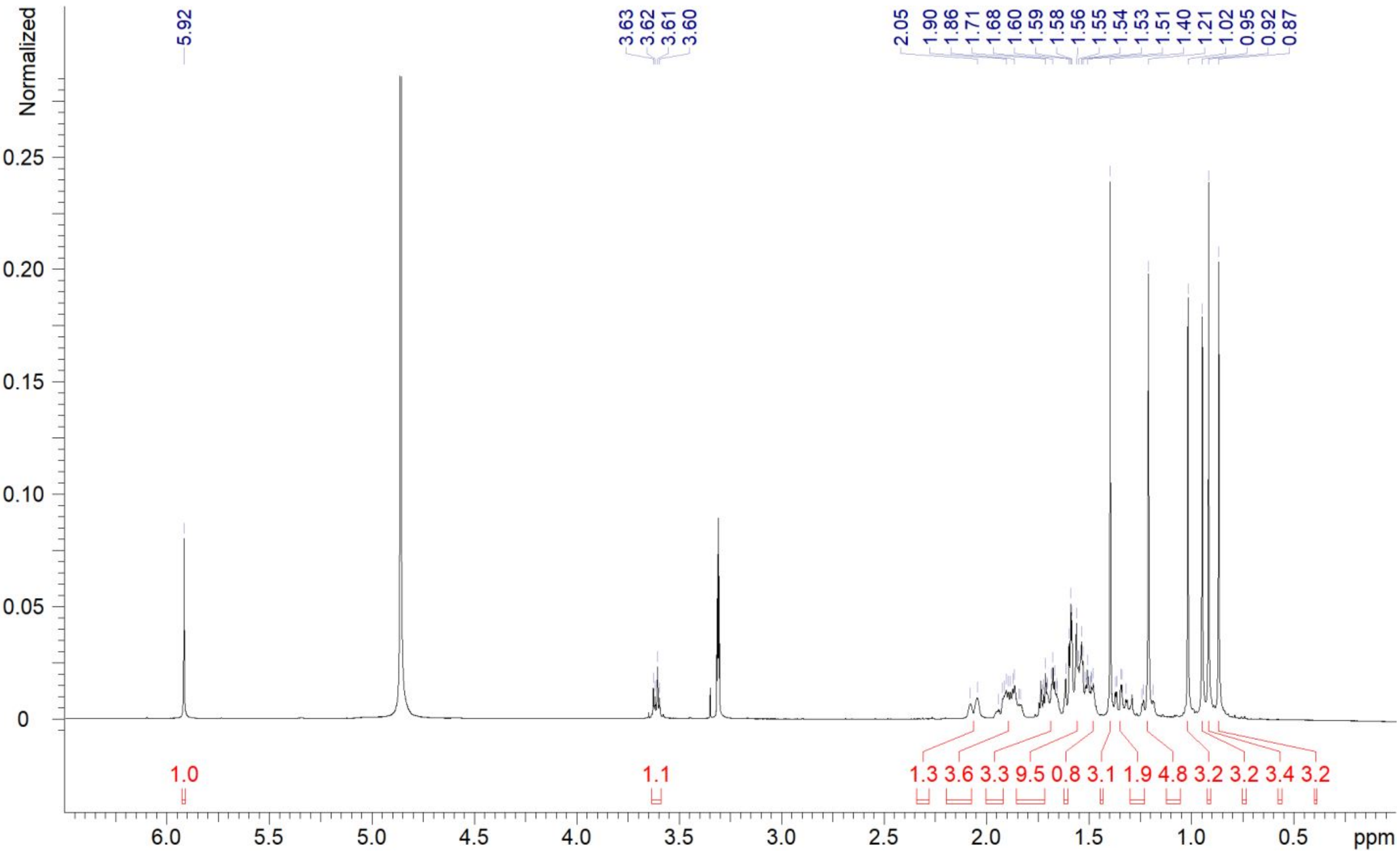

Figure S63: ^{1}H-NMR spectrum (500 MHz) of darumycin G (**8**) in CD_3OD.

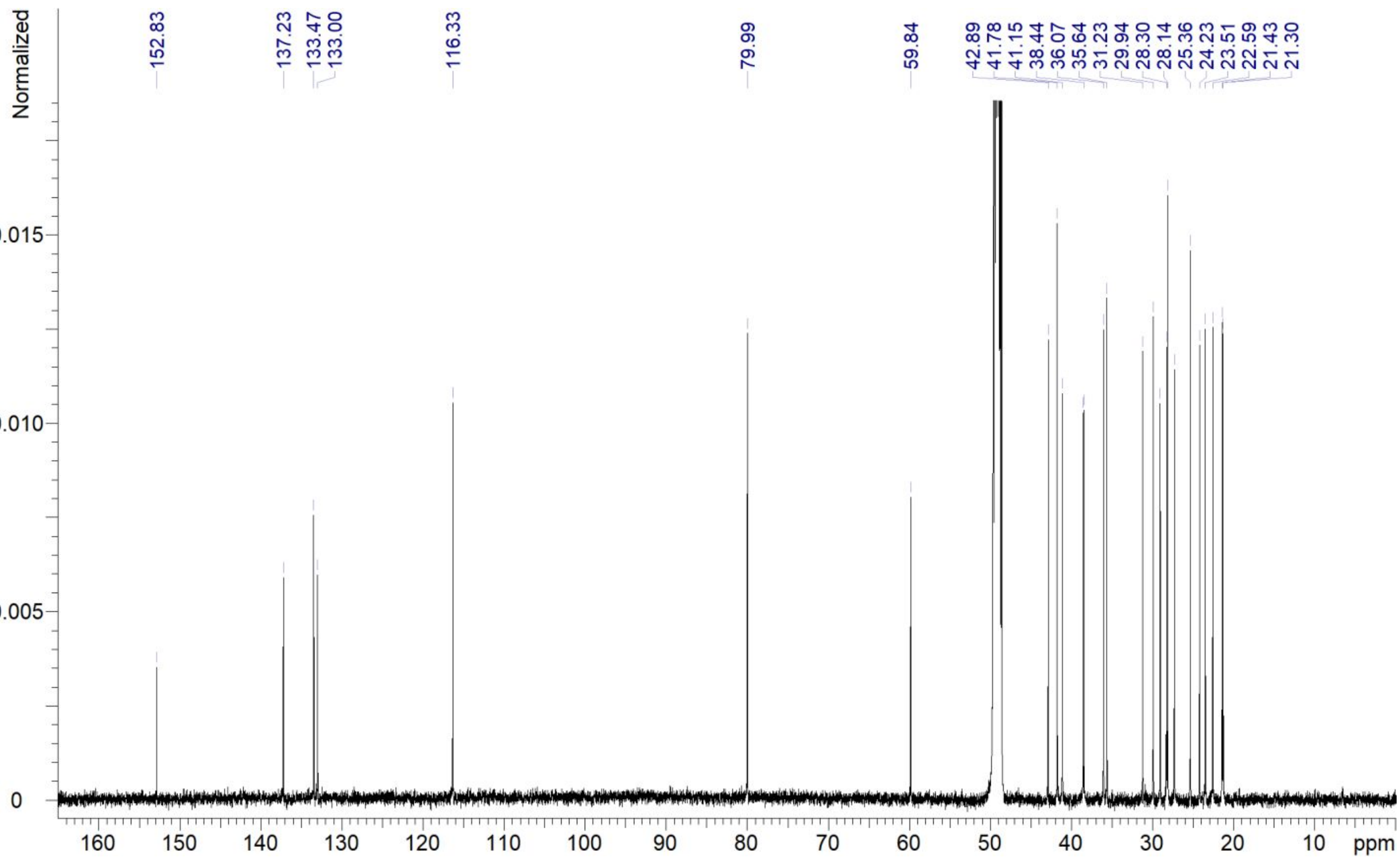

Figure S64*:* ^{13}C-NMR spectrum (125 MHz) of darumycin G (**8**) in CD_3OD.

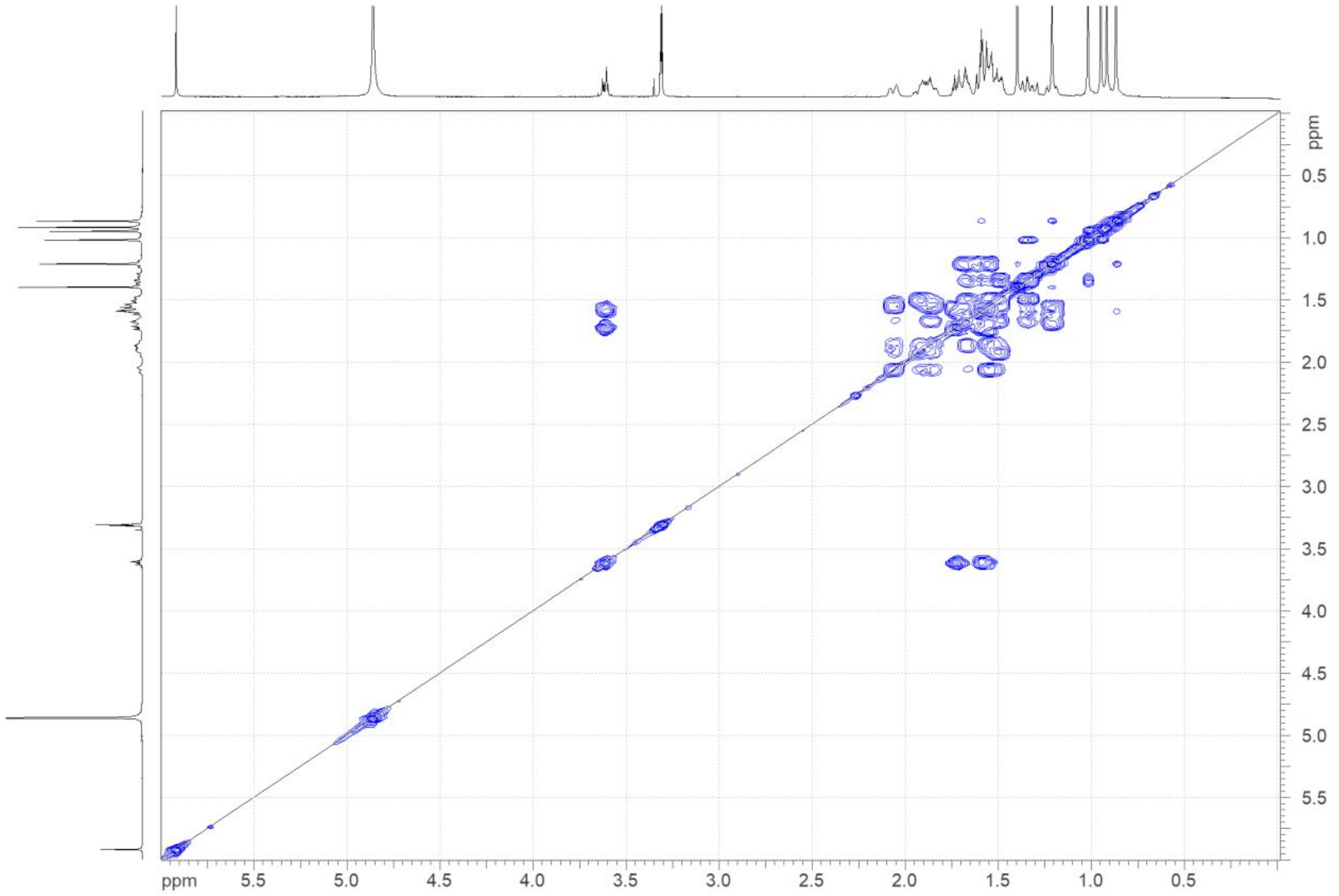

Figure S65: H-H COSY spectrum (500 MHz) of darumycin G (**8**) in CD_3OD.

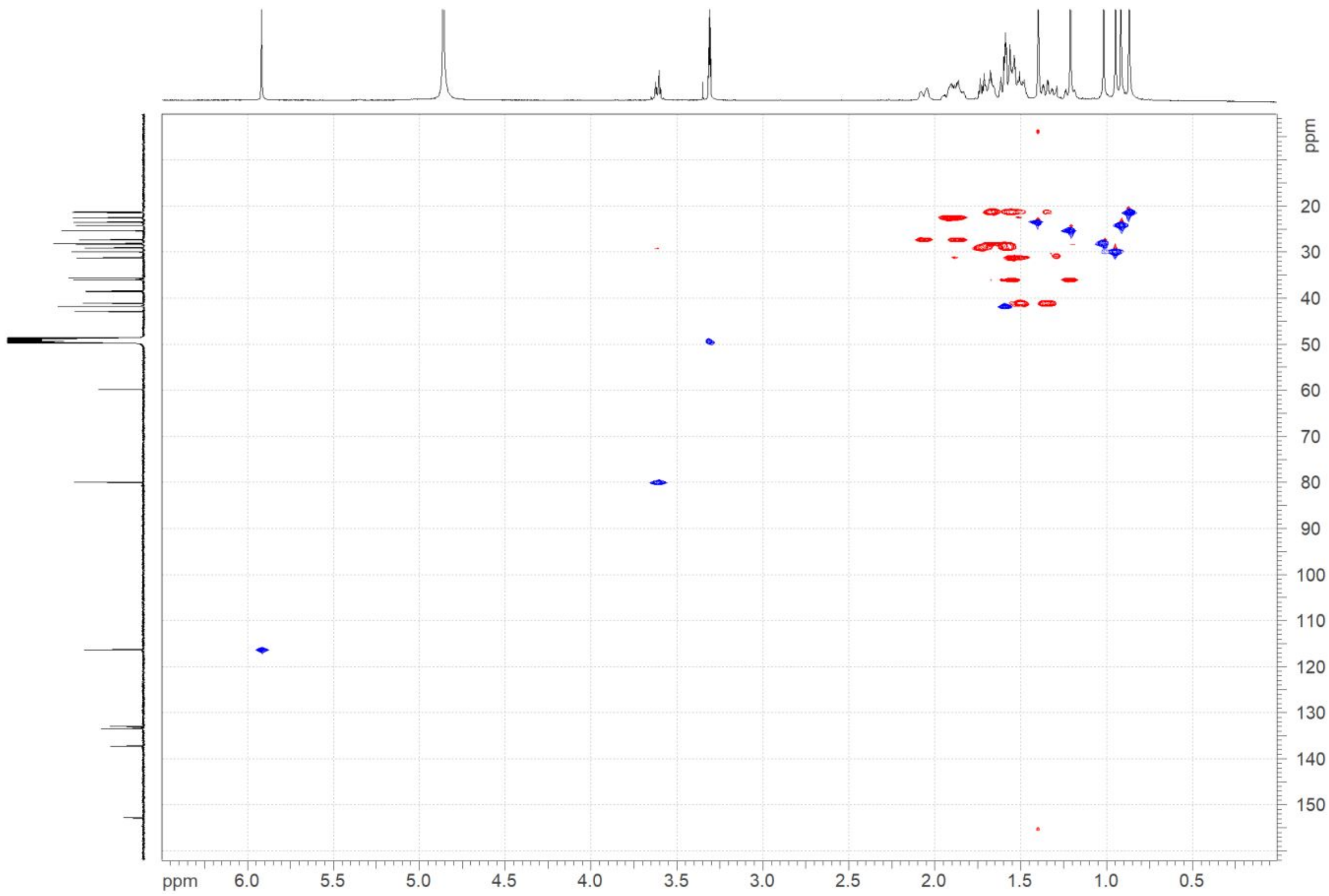

Figure S66: Edited HSQC spectrum (500 MHz) of darumycin G (**8**) in CD_3OD.

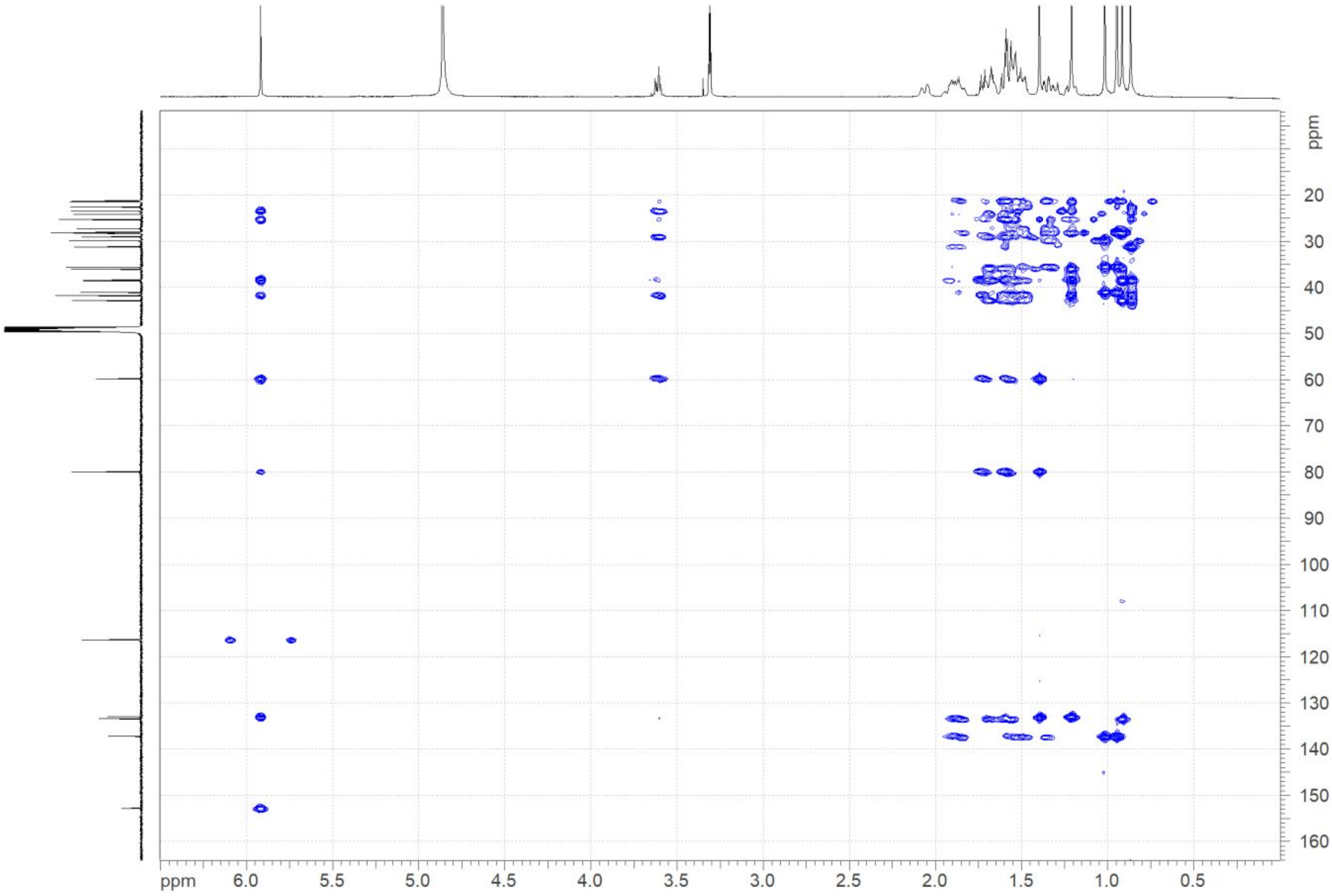

Figure S67: HMBC spectrum (500 MHz) of darumycin G (**8**) in CD_3OD.

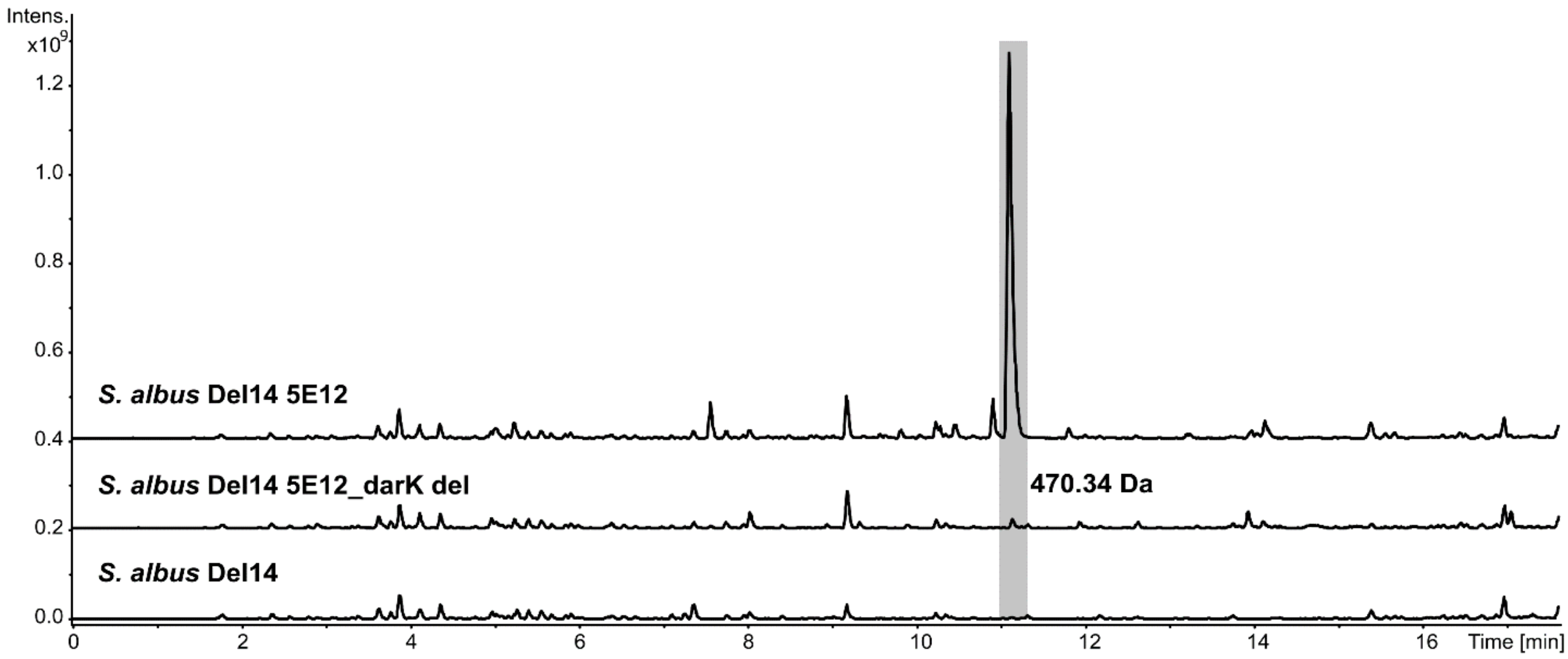

Figure S68: HPLC-MS chromatograms of the strains *S. albus* Del14, *S. albus* Del14 cos15AAmInt-5E12, *S. albus* Del14 cos15AAmInt-5E12_darK del. The peak representing darumycin A (**1**) is marked by a grey rectangle. The production of darumycin A (**1**) is significantly lowered in the strain harboring the *darK* gene deletion.

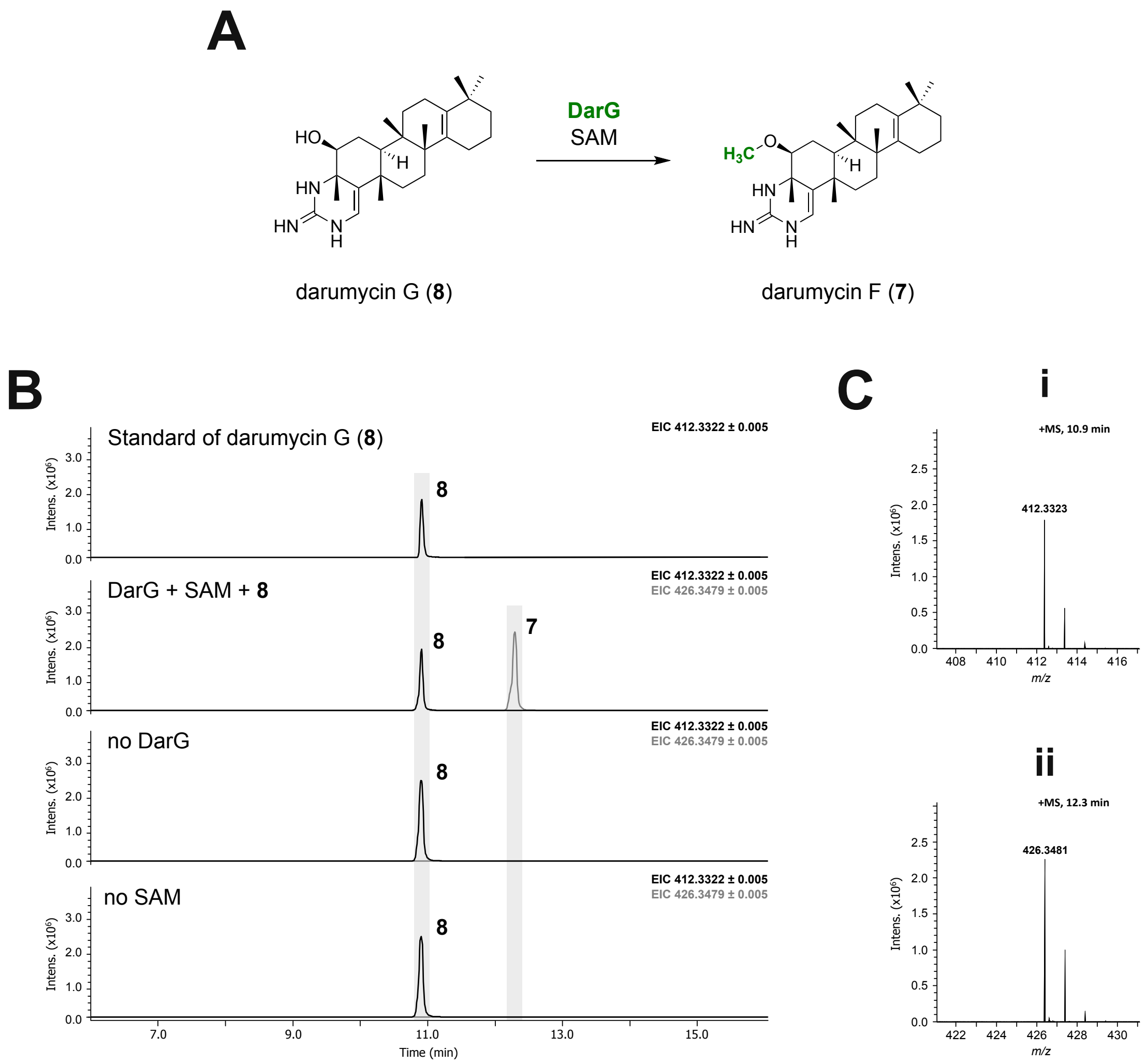

Figure S69: LC-MS analysis of DarG biochemical reactions using darumycin G (**8**) as substrates.

(A) Biochemical reaction of DarG in presence of SAM and darumycin G (8) as substrate. (B) LC-MS analysis of SAM-dependent DarG reaction using darumycin G (8) as substrate revealed efficient enzymtic formation of darumycin F (7). (C) ESI-MS spectrum of i darumycin G (8), ii darumycin F (7).

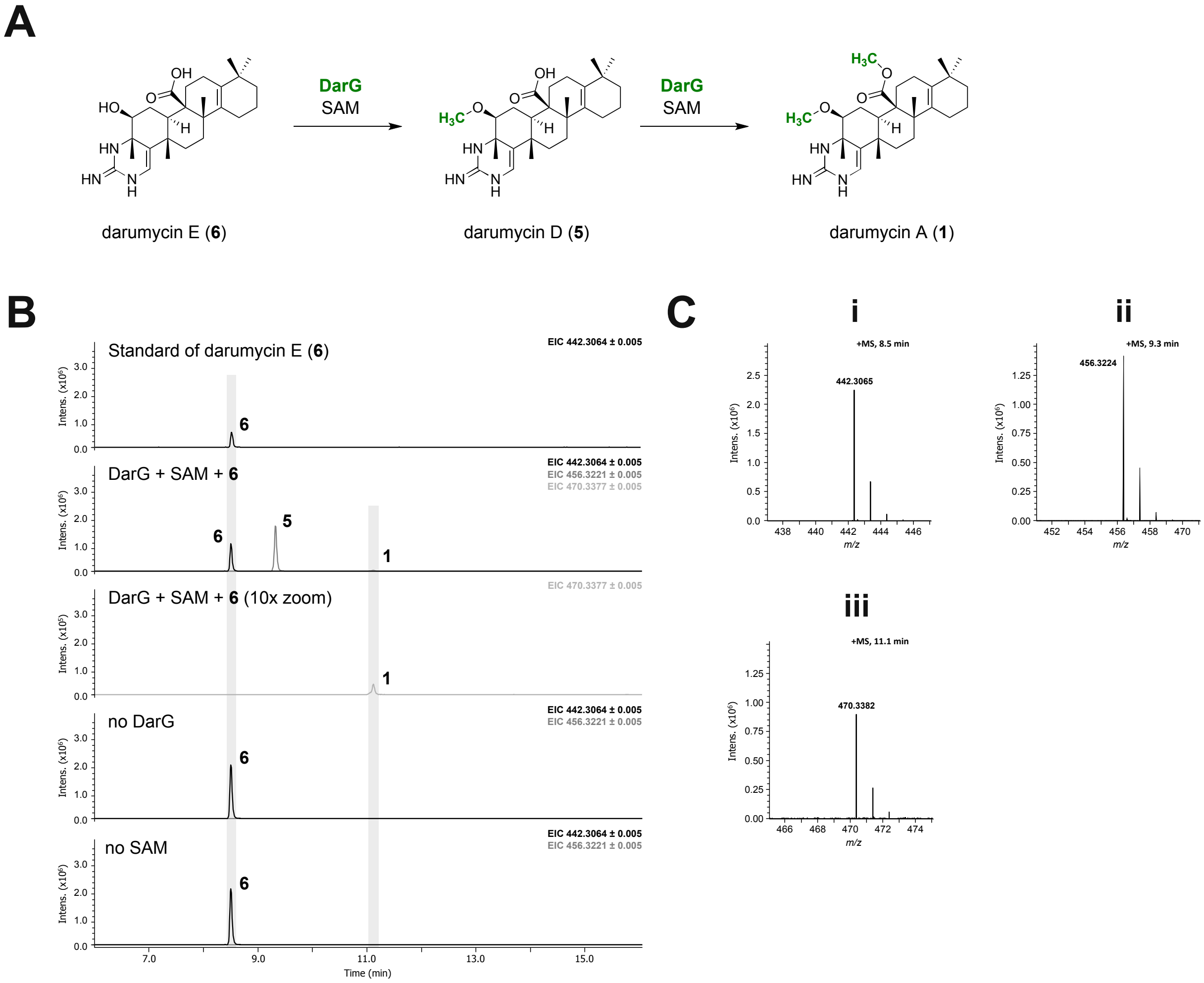

Figure S70: LC-MS analysis of DarG biochemical reactions using darumycin E (**6**) as substrates.

(A) Biochemical reaction of DarG in presence of SAM and darumycin E (6) as substrate. (B) LC-MS analysis of DarG reaction indicated the formation of darumycin D (5), as well as the conversion into darumycin A (1) in trace amounts. (C) ESI-MS spectrum of i darumycin E (6), ii darumycin D (5) and iii darumycin A (1).

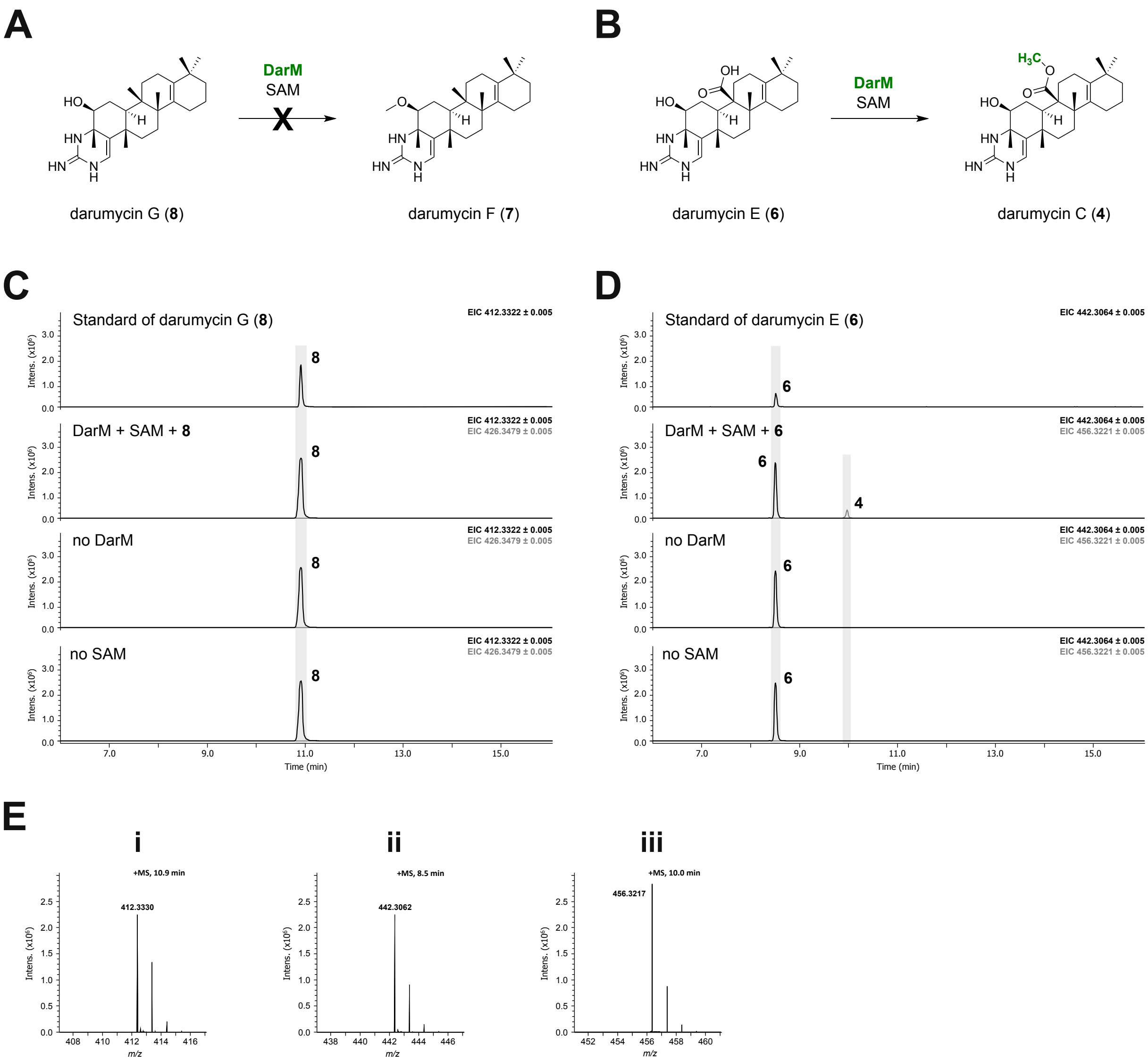

Figure S71: LC-MS analysis of DarM biochemical reactions using darumycin G (**8**) and darumycin E (**6**) as substrates.

(A) Biochemical reaction of DarM in presence of SAM and darumycin G (8) as substrate. (B) Biochemical reaction of DarM in presence of SAM and darumycin E (6) as substrate. (C) LC-MS analysis of DarM reaction with darumycin G (8) and SAM. No conversion of compound 8 into methylated derivative was observed after incubation with DarM. (D) LC-MS analysis of DarM reaction with darumycin E (6) and SAM. Marginal conversion in darumycin C (4) was detected. (E) ESI-MS spectrum of i darumycin G (8), ii darumycin E (6) and iii darumycin C (4).

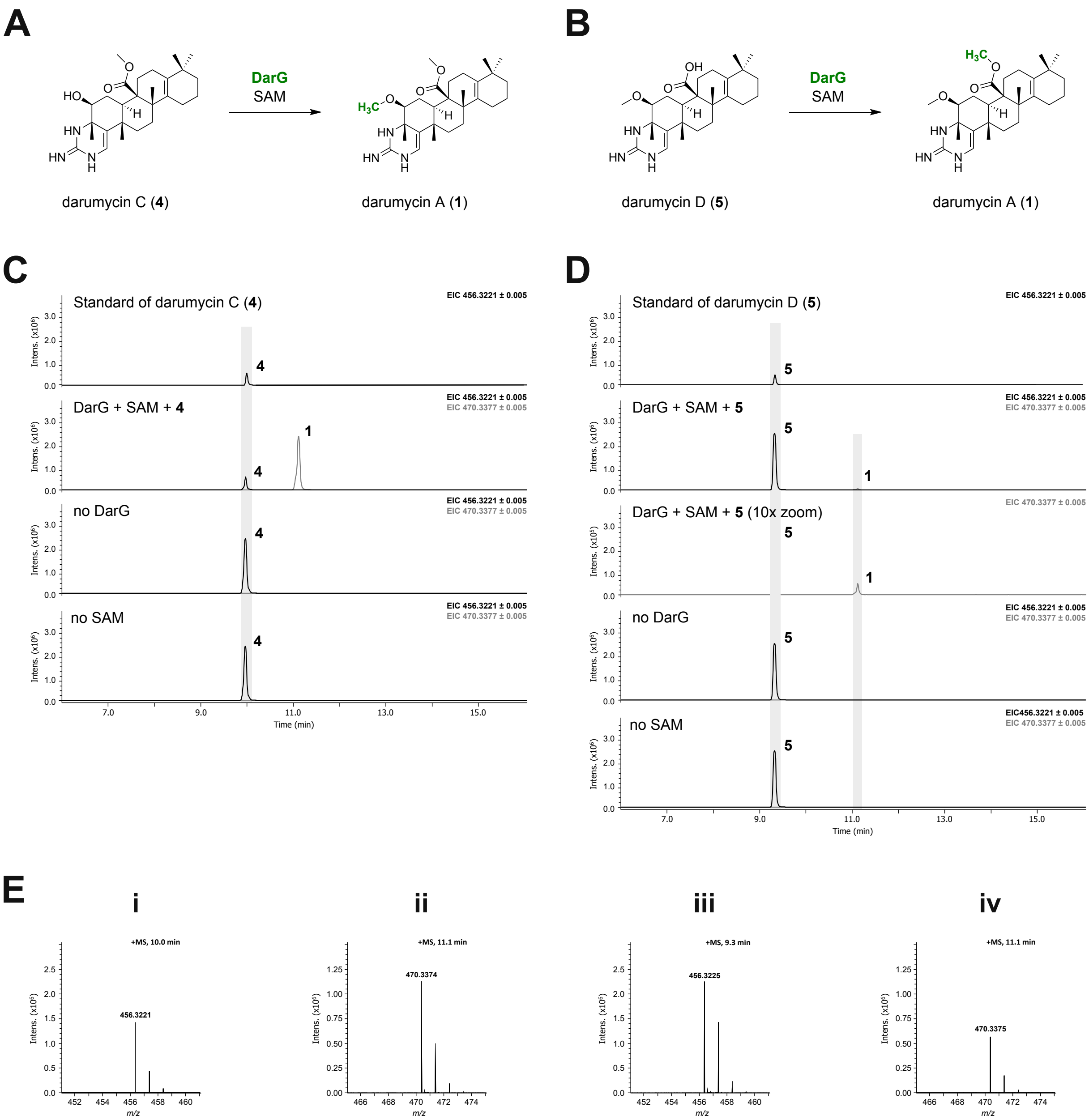

Figure S72: LC-MS analysis of DarG biochemical reactions using darumycin C(4) and darumycin D (5) as substrates.

(A) Biochemical reaction of DarG in presence of SAM and darumycin C (4) as substrate. (B) Biochemical reaction of DarG in presence of SAM and darumycin D (5) as substrate. (C) LC-MS analysis of the DarG catalyzed formation of darumycin A (1) in presence of substrate darumycin C (4) and cofactor SAM. (D) LC-MS analysis of the DarG catalyzed formation of darumycin A (1) in presence of substrate darumycin D (5) and cofactor SAM. The final natural product was detected in trace amounts (E) ESI-MS spectrum of i darumycin C (4), ii darumycin A (1), iii darumycin D (5) and iv darumycin A (1).

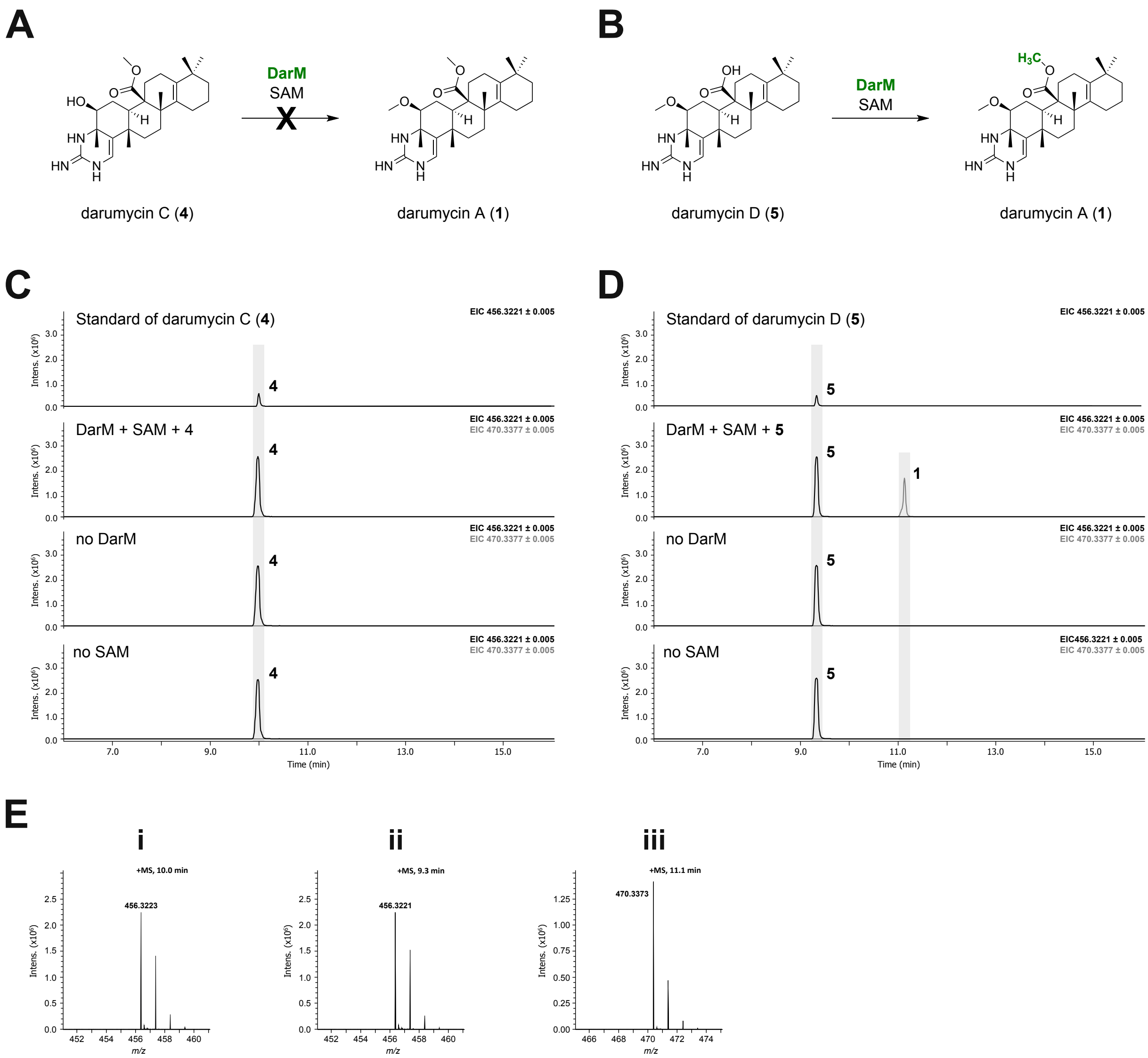

Figure S73: LC-MS analysis of DarM biochemical reactions using darumycin C (**4**) and darumycin D (**5**) as substrates.

(A) Biochemical reaction of DarM in presence of SAM and darumycin C (4) as substrate. (B) Biochemical reaction of DarM in presence of SAM and darumycin D (5) as substrate. (C) LC-MS analysis of DarM reaction with darumycin C (4) and SAM. No enzymatic conversion of darumycin C (4) in darumycin A (1) was detected. (D) LC-MS analysis of DarM reaction with darumycin D (5) and SAM. Formation of darumycin A (1) catalyzed by DarM was observed after incubation of substrate darumycin D (5) and SAM. (E) ESI-MS spectrum of i darumycin C (4), ii darumycin D (5) and iii darumycin A (1).

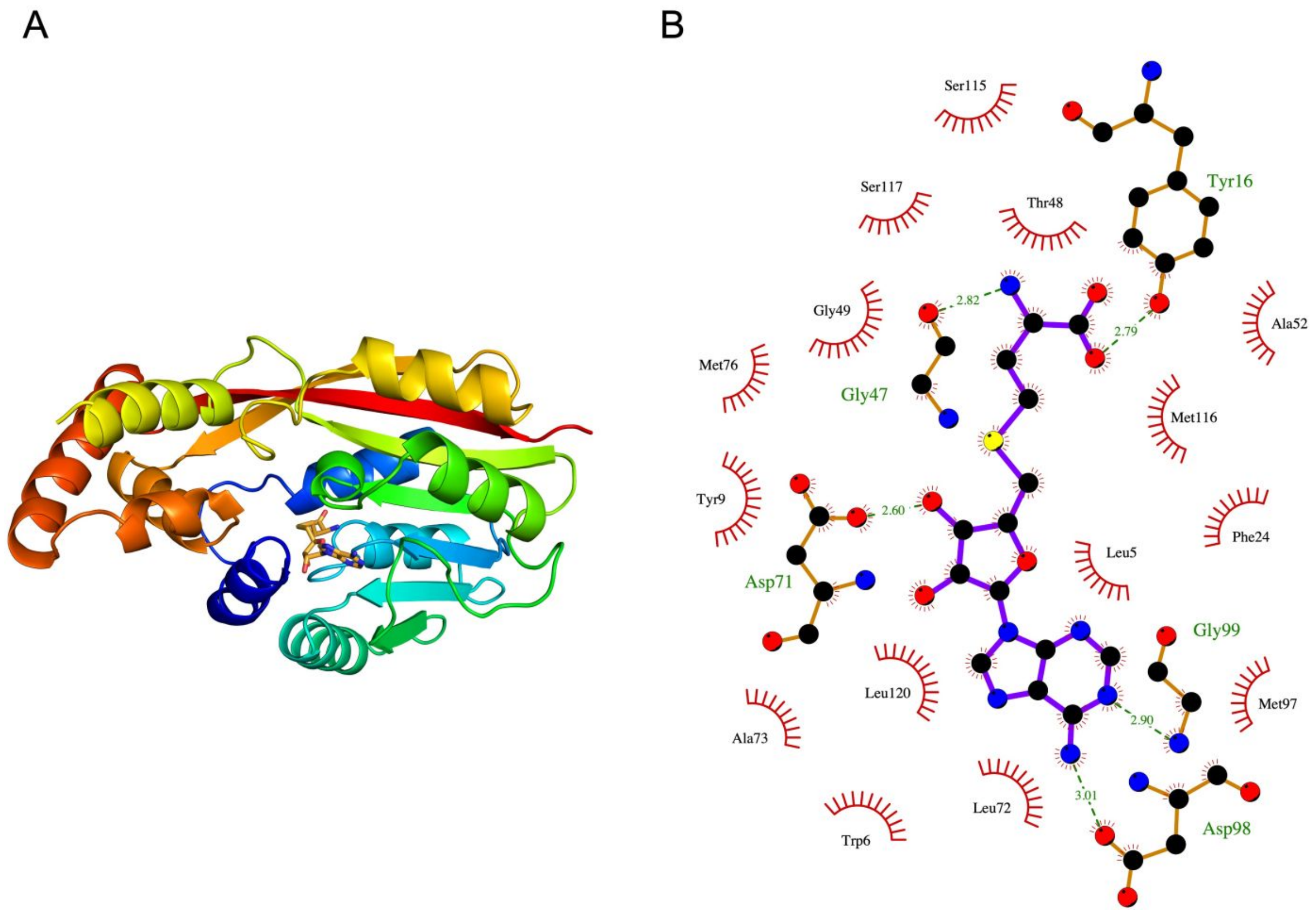

Figure S74: Crystal structure of DarM in complex with SAH. (A) Cartoon representation of DarM crystal structure in complex with SAH cofactor depicted in rainbow color. (B) LigPlot+ diagram for interactions between DarM and its bound cofactor SAH. Ligand bonds are shown in purple whereas protein bonds are colored in dark brown. The distance for hydrogen bonds is shown and the interactions are represented as green, dashed lines. Hydrophobic interactions are shown as red spoked arcs.

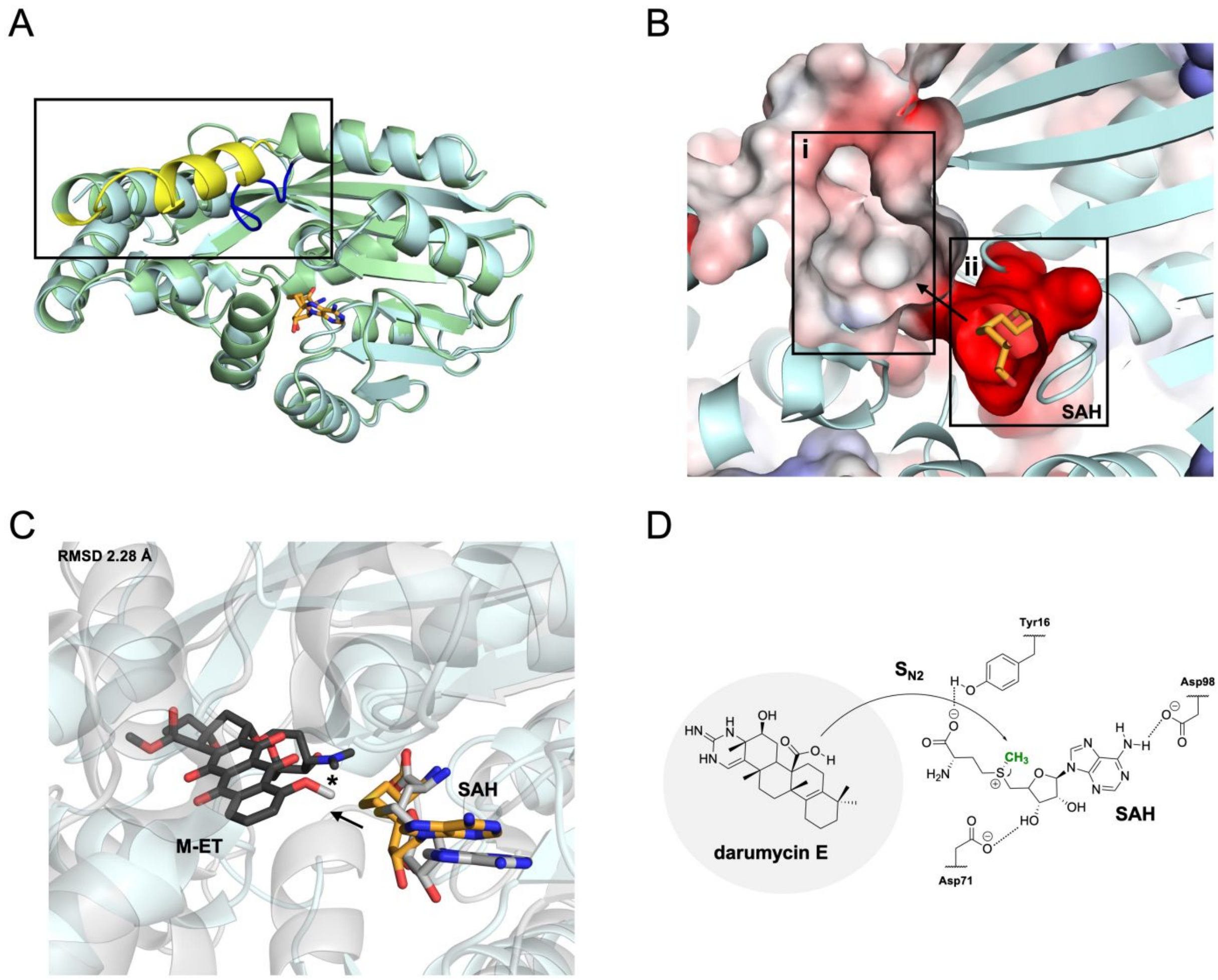

Figure S75: Detailed view on DarM substrate pocket and propsed mechanism for SAM-dependent enzymatic methylation of darumycin E. (**A**) Superposition of DarM (light blue) in complex with SAH (orange sticks) and a AlphaFold3 model (green) of DarM. The unresolved loop (Asp168-Asp180) is shown as yellow colored helix in the AlphaFold3 model. The blue colored coil region in the experimental structure covers the potential substrate binding pocket. (**B**) Electrostatic surface representation of DarM. The surface potential of substrate binding pocket (i) and SAM/SAH binding cavity (ii) was visualized using the APBS Tool2.1 plugin implemented in PyMOL. SAH is surounded by negatively charged amino acid side chains, whereas the hydrophobic substrate pocket exhibits a neutral electrostatic surface. Both pockets are connected via a narrow tunnel (black arrow) enabling the methyl transfer. (**C**) Superposition of DarM (light blue) in complex with SAH (orange) and DnrK (PDB ID 1tw2) (grey) in complex with 4-methoxy-e-rhodomycin T (M-ET, black) and SAH (grey). The cofactor and the substrate are positioned in close proximity for methyl transfer. The methylated hydroxyl group in M-ET is indicated by asterisk and the direction of methyltransfer is highlighted by an arrow. (**D**) Proposed mechanism of the SAM-dependent methyltransferase DarM. The nucleophilic oxygen atom of the darumycin's carboxyl group attacks the polarized methyl group of SAM and the methyl transfer follows a S_{N2} mechanism. The darumycin substrate binding pocket is simplified as grey sphere. Amino acid side chains engaged in SAM binding are shown and hydrogen bond interactions are highlighted as dashed lines.

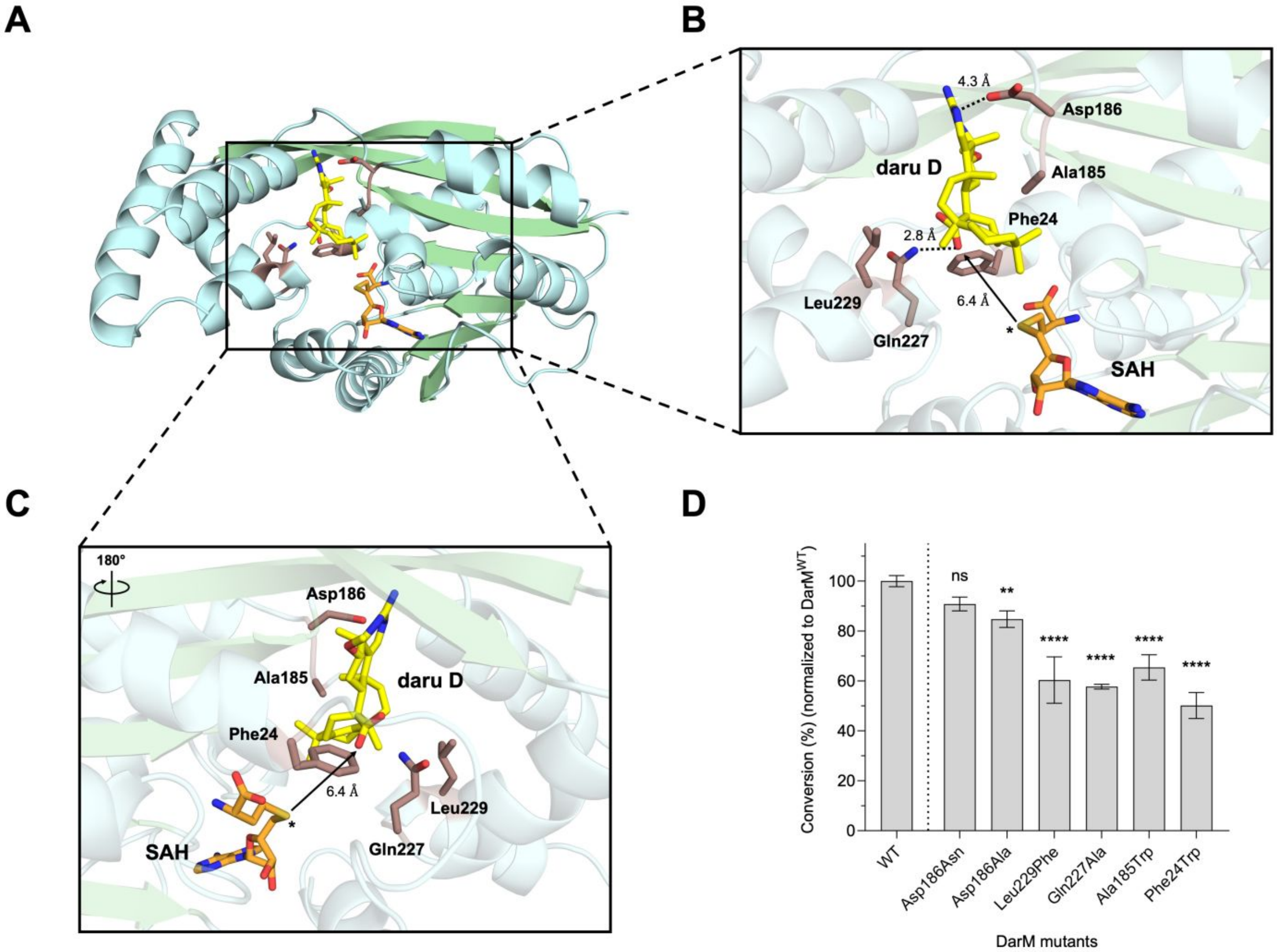

Figure S76: Model of DarM-SAH crystal structure with darumycin D and biochemical evaluation of DarM mutants. (**A**) Modeled complex of DarM crystal structure with SAH and docked substrate darumycin D using AutoDock Vina 1.2.0.[10] DarM is show as green and light-blue cartoons, SAH is dipicted as orange sticks, docked darumycin D is shown as yellow sticks and amino acid residues presumably involved in substrate binding are shown as dark-red sticks. (**B**) Detailed view on predicted darumycin D substrate binding site. Amino acid side changes predicted to participate in substrate binding are labeled. Hydrogen bond and salt bridge interactions are indicated by dashed lines and the distance is shown in Å. The direction of methyl transfer is indicated by an arrow and the distance form the SAH sulfur atom to the oxygen atom of the substrate carboxyl moiety is shown in Å. (**C**) Detailed view on predicted darumycin D substrate binding site rotated by 180°. (**D**) *In vitro* biochemical characterization of selected single amino acid mutants of DarM using darumycin D as substrate. Data represent mean ± SD from independent biochemical reactions (n = 3) and product formation of all mutants was normalized to DarM wild-type. Statistical analysis was performed by one-way ANOVA with Dunnett's multiple comparison test. $p < 0.05$: *, $p < 0.005$: **, $p < 0.0005$: ***, $p < 0.0001$: ****

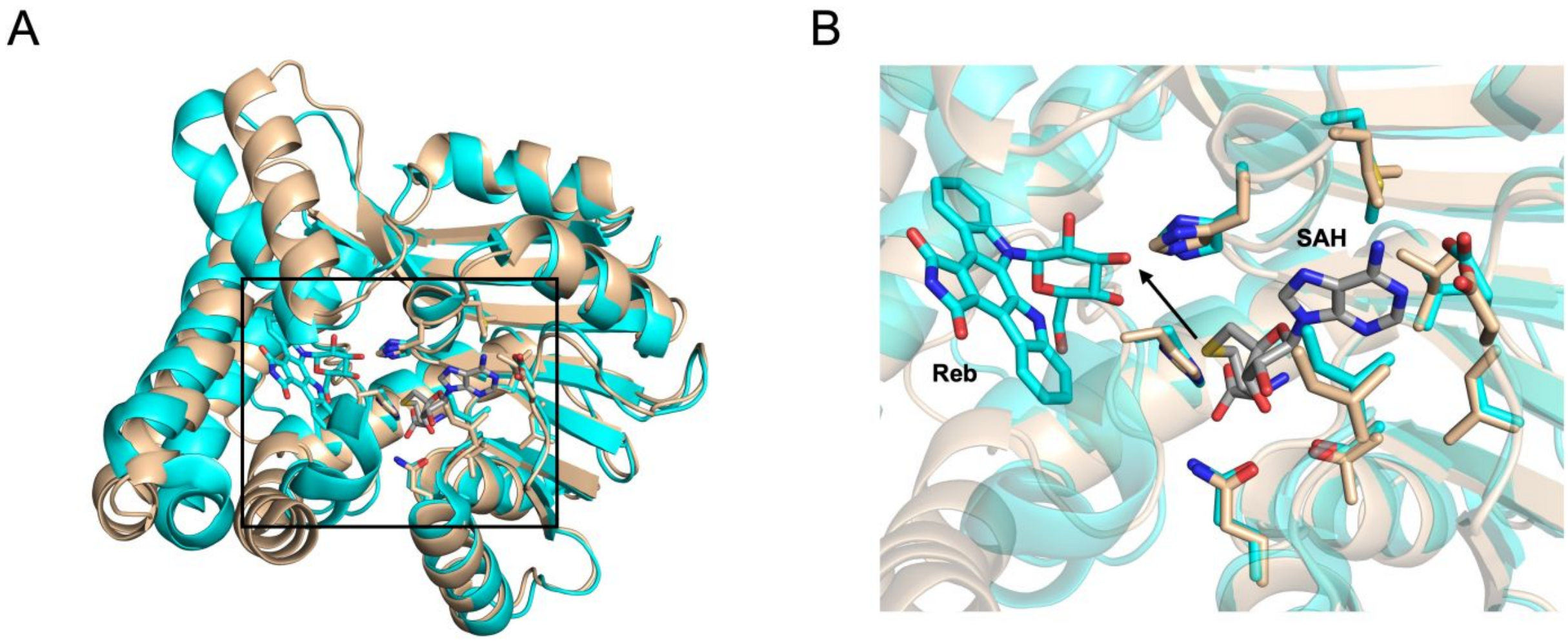

Figure S77: Superposition of DarG AlphaFold3 model and AtM involved in rebeccamycin biosynthesis. (**A**) Cartoon representation and superposition of DarG AlphaFold3 model (wheat) and AtM (cyan, PDB ID 6uv6) involved in rebeccamycin biosynthesis. (**B**) Close-up view on the active site of AtM in complex with rebeccamycin analog (cyan) and SAH (grey). Conserved amino acid side chains engaged in SAH cofactor binding are represented as sticks.

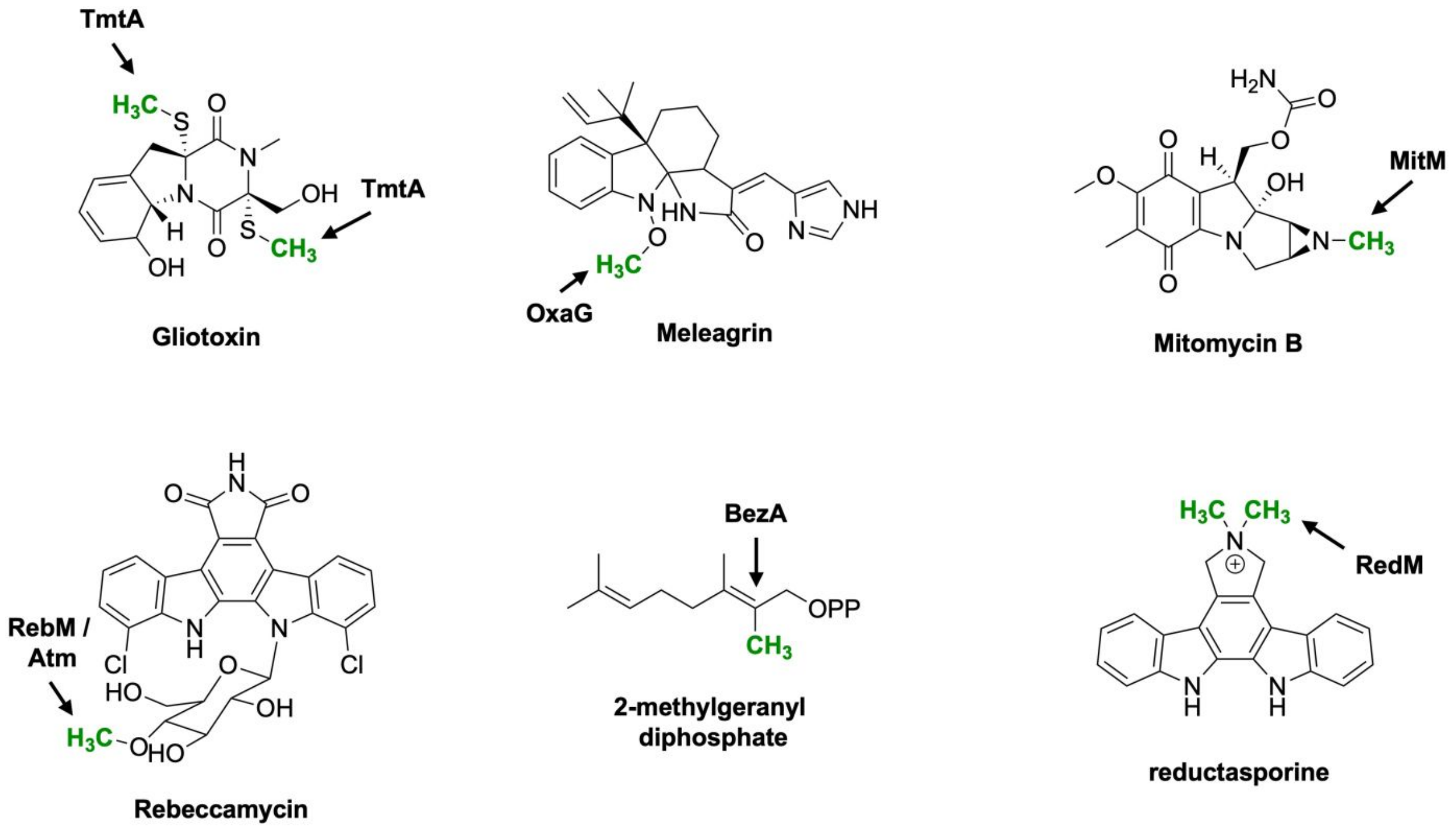

Figure S78: Natural products whose biosynthesis involves enzymatic methylation via SAM-dependent methyltransferases. Added methyl groups are highlighted in bold green and the site of enzymatic methylation is indicated by an arrow. Natural products names and the enzyme responsible for methyltransfer is shown.

List of Supplementary Tables

Table S1: NMR data of Darumycin A (**1**) in CD_3OH.

No	δ_C [ppm]	δ_H [ppm], mult (J)	HHCOSY	HMBC (H→C/N)	key-NOESY
1	117.4, CH	5.97, bs		1', 2, 3, 7, N_1	
2	131.9, C				
3	59.0, C				
4	89.5, CH	3.13, dd (11.8,3.9)	5	3, 6, 21, N_2	
5	25.2, CH_2	a: 2.27, m<br>b: 1.47, m	4, 6	3, 4, 6, 7, 11	 22
6	41.8, CH	1.85, m	5	2, 4, 5, 7, 8, 12, 24	
7	38.3, C				
8	36.7, CH_2	a: 1.64, m<br>b: 1.25, m	9	6, 7, 10, 22	22
9	28.2, CH_2	a: 2.38, td (14.6, 2.9)<br>b: 1.72, dt (15.3, 3.5)	8	7, 8, 10, 11, 15, 23	22
10	42.8, C				
11	51.0, C				
12	26.4, CH_2	a: 2.25, m<br>b: 1.86, m	13	6, 10, 11, 13, 14, 24	
13	22.4, CH_2	a: 2.10, m<br>b: 1.98, m	12	11, 12, 14, 15, 19	20, 23<br>25
14	135.9, C				
15	134.6, C				
16	27.0, CH_2	a: 2.09, m<br>b: 1.87, m	17	14, 15, 17	
17	21.2, CH_2	a: 1.68, m<br>b: 1.53, m	16, 18	15, 19	
18	41.0, CH_2	a: 1.50, m<br>b: 1.35, m	17	14, 16, 17, 19, 20, 25	
19	35.5, C				
20	28.1, CH_3	1.02, s		14, 18, 19, 25	H-13a
21	23.7, CH_3	1.34, s		2, 3, 4, N_2	H-5b, 22, 4-OMe,
22	23.3, CH_3	1.07, s		2, 6, 7, 8	21, H-9a, H-8a, H-5b
23	25.4, CH_3	1.18, s		9, 10, 11, 15	24-OMe, H-13a, H-9a
24	177.2, C				
25	29.8, CH_3	0.98, s		14, 18, 19, 20	H-13b
4-OMe	57.5, CH_3	3.47, s		4	H-5a, 21
24-OMe	51.6, CH_3	3.64, s		24	
1'	153.0, C				
N_1	94.7, NH				
**N_2**	98.6, NH				

Table S2: NMR data of Darumycin B (**2**) in CD_3OD.

No	δ_C [ppm]	δ_H [ppm], mult (J)	HHCOSY	HMBC (H→C/N)
1	117.6, CH	5.96, s		N_1, 2, 3, 7
2	131.9, C			
3	58.9, C			
4	89.1, CH	2.99, dd (11.8, 4.0)	5	N_2, 3, 4-OMe
5	24.9, CH_2	a: 2.19, m	4, 6	3, 4, 6, 7
		b: 1.56, m		
6	42.7, CH	1.74, dd (12.6, 1.8)	5	2, 4, 11, 24
7	39.0, C			
8	35.3, CH_2	a: 1.79, m	9	6, 10
		b: 1.54, m		
9	28.3, CH_2	a: 2.66, m	8	7, 8, 11
		b: 1.88, m		
10	43.4, C			
11	50.6, C			
12	30.9, CH_2	a: 2.71, dd (18.7, 6.7)	13	6, 10, 11, 13, 14, 24
		b: 2.32, bd (19.2)		
13	114.3, CH	5.54, d (6.4)	12	11, 12, 15, 19
14	143.6, C			
15	139.5, C			
16	123.9, CH	5.83, t (4.2)	17	10, 14, 17, 18
17	24.4, CH_2	a: 2.28, m	16, 18	15, 16, 18, 19
		b: 2.18, m		
18	37.3, CH_2	1.42, m	17	14, 16, 17, 19, 20
19	34.9, C			
20	29.2, CH_3	1.05, s		14, 18, 19, 25
21	23.4, CH_3	1.32, s		N_2, 2, 3, 4
22	23.8, CH_3	1.04, s		2, 6, 7, 8
23	27.2, CH_3	1.23, s		9, 10, 11, 15
24	177.4, C			
25	28.4, CH_3	1.05, s		4, 18, 19, 20
4-OMe	57.4, CH_3	3.44, s		4
24-OMe	51.6, CH_3	3.65, s		24
1'	152.9, C			
N_1	94.5, NH			
N_2	98.0, NH			

Table S3: Proposed function of genes in darumycin and gromomycin BGCs.

dar BGC	Function	*gro* BGC	Function	ID (%)
darA	Hypothetical protein (cyclase)	*groF*	Hypothetical protein (cyclase)	54
darB	Hypothetical protein (prenyltransferase)	*groH*	Hypothetical protein (prenyltransferase)	50
darC	Cytochrome P450 oxygenase	*groI*	Cytochrome P450 107B1 (Monooxygenase/Oxidoreductase)	31
darD	Polyprenyl synthetase family protein	*groD*	Polyprenyl synthetase family protein	48
darE	Rieske 2Fe-2S domain-containing protein	*groC*	Rieske 2Fe-2S domain-containing protein	56
darF	Rieske 2Fe-2S domain-containing protein	*groB*	Rieske 2Fe-2S domain-containing protein	40
darG	Methyltransferase domain-containing protein	-	-	-
darH	Protein phosphatase	*gro3*	Protein phosphatase	53
darI	Rieske 2Fe-2S domain-containing protein	*groA*	Rieske 2Fe-2S domain-containing protein	50
darJ	Phosphoenolpyruvate synthase	*gro5*	Phosphoenolpyruvate synthase	40
darK	Hypothetical protein	-	-	-
darL	Lysylphosphatidylglycerol synthase	*gro4*	Lysylphosphatidylglycerol synthase	35
darM	Methyltransferase domain-containing protein	-	-	-
dar3	phosphatase PAP2 family protein	*groG*	PAP2 superfamily protein	21
dar4	QacE family quaternary ammonium compound efflux SMR transporter	-	-	-

Table S4: NMR data of Pentaprenylguanidine (**3**) in CD_3OD.

No	δ_C [ppm]	δ_H [ppm], mult (J)	HHCOSY	HMBC (H→C)
1'	158.7, C			
1	40.4, CH_2	3.71, d (6.8)	2	1', 2, 3
2	119.2, CH	5.20, tq (9.8, 1.1)	1	1, 4, 21
3	142.9, C			
4	40.7, CH_2	2.00, m		2, 5
5	27.5, CH_2	2.07, m		3, 4, 6, 7
6	125.1, CH	5.06, m		5
7	136.7, C			
8	41.0, CH_2	1.91, m	9	6, 10, 22
9	28.0, CH_2	2.01, m	8, 10	7, 11
10	125.6, CH	5.03, m	9	8, 12, 23
11	136.1, C			
12	41.0, CH_2	1.91, m	13	10, 14, 23
13	27.7, CH_2	2.01, m	12, 14	11, 15
14	125.6, CH	5.03, m	13	12, 16, 24
15	136.0, C			
16	41.0, CH_2	1.91, m	17	14, 18, 24
17	27.8, CH_2	2.01, m	16, 18	15, 19
18	125.6, CH	5.03, m	17	16, 20, 25
19	132.2, C			
20	17.9, CH_3	1.52, bs		18, 19, 25
21	16.6, CH_3	1.65, bs		2, 3, 4
22	16.3, CH_3	1.54, bs		6, 7, 8
23, 24	16.3, CH_3	1.52, bs		10, 11, 12, 14, 15, 16
25	26.1, CH_3	1.59, bs		18, 19, 20

Table S5: NMR data of Darumycin C (**4**) in CD_3OD.

No	δ_C [ppm]	δ_H [ppm], mult (J)	HHCOSY	HMBC (H→C)
1	117.3, CH	5.97, s		2, 3, 7
2	132.2, C			
3	59.6, C			
4	79.6, CH	3.55, dd (11.8, 4.2)	5	3, 5, 6, 21
5	30.9, CH_2	a: 1.99, m<br>b: 1.70, m	4, 6	3, 4, 6, 7, 11
6	42.1, CH	1.90, m	5	2, 4, 5, 7, 8, 10, 12, 24
7	38.3, C			
8	36.6, CH_2	a: 1.64, m<br>b: 1.25, m	9	6, 7, 10, 22
9	28.2, CH_2	a: 2.39, td (14.6, 3.1)<br>b: 1.72, m	8	7, 8, 10, 11, 15, 23
10	42.8, C			
11	50.8, C			
12	26.4, CH_2	a: 2.23, ddd (14.0, 5.3, 1.2)<br>b: 1.84, m	13	10, 11, 14, 24
13	27.0, CH_2	a: 2.09, m<br>b: 1.87, m	12	11, 12, 14, 15
14	135.9, C			
15	134.4, C			
16	22.3, CH_2	2.05, m	17	14, 15, 18
17	21.2, CH_2	a: 1.69, m<br>b: 1.55, m	16, 18	15, 19
18	41.0, CH_2	a: 1.51, m<br>b: 1.35, m	17	14, 16, 20, 25
19	35.5, C			
20	29.8, CH_3	0.97, s		14, 18, 19, 25
21	23.2, CH_3	1.36, s		2, 3, 4
22	23.2, CH_3	1.08, s		2, 6, 7, 8
23	25.4, CH_3	1.18, s		15, 9, 10, 11
24	177.3, C			
25	28.1, CH_3	1.02, s		14, 18, 19, 20
24-OMe	51.6, CH_3	3.65, s		24
1’	152.9, C			

Table S6: NMR data of Darumycin D (**5**) in CH_3OD.

No	δ_C [ppm]	δ_H [ppm], mult (J)	HHCOSY	HMBC (H→C)
1	117.1, CH	5.97, s		1', 2, 3, 7
2	132.3, C			
3	59.1, C			
4	89.7, CH	3.15, dd (11.8, 4.0)	5	3, 6, 21, 4-OMe
5	25.3, CH_2	a: 2.30, m	4, 6	3, 4, 6, 7
		b: 1.59, m		
6	41.5, CH	1.83, m	5	2, 4, 5, 7, 8, 11, 22, 24
7	38.6, C			
8	36.9, CH_2	a: 1.64, m	9	2, 6, 7, 9, 10
		b: 1.25, m		
9	28.2, CH_2	a: 2.48, td (14.5, 2.8)	8	7, 8, 10, 11, 15, 23
		b: 1.69, dt (14.8, 3.0)		
10	42.7, C			
11	50.5, C			
12	26.8, CH_2	a: 2.27, m	13	6, 10, 11, 13, 14, 24
		b: 1.86, m		
13	22.5, CH_2	a: 1.99, m	12	11, 12, 14, 15, 19
		b: 2.09, m		
14	135.7, C			
15	134.9, C			
16	27.0, CH_2	a: 1.88, m	17	10, 14, 17, 18
		b: 2.10, m		
17	21.3, CH_2	a: 1.56, m	16, 18	15, 19
		b: 1.69, m		
18	41.1, CH_2	a: 1.35, m	17	14, 16, 19, 20, 25
		b: 1.51, m		
19	35.5, C			
20	29.9, CH_3	0.98, s		14, 18, 19, 25
21	23.8, CH_3	1.36, s		2, 3, 4
22	23.4, CH_3	1.20, s		2, 6, 7, 8
23	25.5, CH_3	1.22, s		9, 10, 11, 15
24	179.4, C			
25	28.1, CH_3	1.03, s		14, 18, 19, 20
4-OMe	57.4, CH_3	3.47, s		4
1'	152.9, C			

Table S7: ^{1}H NMR ($CDCl_3$) data of Darumycin C (*S*)-MTPA ester and Darumycin F (*R*)-MTPA ester) and their $\Delta\delta_{S\text{-}R}$ values.

	Darumycin F (*S*)-MTPA ester δ_H (*J* in Hz)	Darumycin F (*R*)-MTPA ester δ_H (*J* in Hz)	$\Delta\delta_{S\text{-}R}$ (δS -δR)
1	5.80 s	5.88 s	- 0.08
4	4.81 dd (11.5, 3.5)	4.76 dd (11.5, 3.5)	+0.05
5	2.02 m	2.18 m	- 0.16
	1.42 m	1.68 m	- 0.26
6	1.84 d (13)	1.89 d (13)	- 0.05
8	1.43 m	1.49 m	- 0.06
	1.11 m	1.14 m	- 0.03
9	2.20 m	2.21 m	- 0.01
	1.62	1.64 m	- 0.02
12	1.95 m	1.97 m	- 0.02
	1.71 m	1.72 m	- 0.01
13	1.96 m	1.97 m	- 0.01
	1.74 m	1.74 m	0.00
16	1.98 m (2H)	1.98 m (2H)	0.00
17	1.61 m	1.61 m	0.00
	1.44 m	1.44 m	0.00
18	1.43 m	1.43 m	0.00
	1.35 m	1.35 m	0.00
20	0.97 s (3H)	0.97 s (3H)	0.00
21	1.29 s (3H)	1.33 s (3H)	- 0.04
22	0.91 s (3H)	0.98 s (3H)	- 0.07
23	1.09 s (3H)	1.10 s (3H)	- 0.01
25	0.94 s (3H)	0.94 s (3H)	0.00
24-OCH_3	3.50 s (3H)	3.58 s (3H)	- 0.08

Table S8: NMR data of Darumycin E (**6**) in CD_3OD.

No	δ_C [ppm]	δ_H [ppm], mult (J)	HHCOSY	HMBC (H→C)
1	116.9, CH	5.95, s		2, 6, 7, 8
2	132.7, C			
3	59.7, C			
4	79.9, CH	3.56, dd (10.8, 3.8)	5	3, 4, 6
5	31.0, CH_2	a: 2.01, m<br>b: 1.85, m	4, 6	3, 4, 6, 7, 11
6	41.8, CH	1.84, m	5	4, 5, 10, 22, 24
7	38.5, C			
8	36.9, CH_2	a: 1.24, m<br>b: 1.62, m	9	2, 6, 7, 9, 10
9	28.3, CH_2	a: 2.52, td (14.7, 2.3)<br>b: 1.66, m	8	7, 8, 10, 11, 15, 23
10	42.7, C			
11	50.5, C			
12	22.5, CH_2	2.03, m	13	6, 13, 14, 24
13	26.9, CH_2	a: 2.24, m<br>b: 1.82, m	12	11, 12, 14
14	135.7, C			
15	134.9, C			
16	27.1, CH_2	a: 1.87, m<br>b: 2.09, m	17	14, 15, 18
17	21.3, CH_2	a: 1.68, m<br>b: 1.55, m	16, 18	15, 19
18	41.1, CH_2	a: 1.49, m<br>b: 1.34, m	17	14, 16, 17, 19, 20, 25
19	35.5, C			
20	29.9, CH_3	0.96, s		14, 18, 19, 25
21	23.4, CH_3	1.37, s		2, 3, 4
22	23.5, CH_3	1.21, s		2, 6, 7, 8
23	25.6, CH_3	1.21, s		9, 10, 11, 15
24	180.0, C			
25	28.1, CH_3	1.02, s		14, 18, 19, 20
1'	152.9, C			

Table S9: NMR data of Darumycin F (**7**) in CH_3OD.

No	δ_C [ppm]	δ_H [ppm], mult (J)	HHCOSY	HMBC (H→C)
1	116.4, CH	5.92, s		2, 3, 7, 1',
2	132.6, C			
3	59.2, C			
4	89.9, CH	3.21, dd (11.6, 4.2)	5	2, 3, 4, 5, 6, 21, 4-OMe
5	23.3, CH_2	a: 1.36, m<br>b: 2.02, m	4, 6	3, 4, 6, 7, 11
6	41.4, CH	1.54, m	5	2, 4, 5, 8, 10, 11, 22, 24
7	38.5, C			
8	36.1, CH_2	a: 1.23, m<br>b: 1.57, m	9	2, 9, 10, 22
9	28.3, CH_2	a: 1.59, m<br>b: 1.69, m	8	7, 8, 10, 11, 15, 23
10	42.9, C			
11	38.8, C			
12	31.2, CH_2	a: 1.60, m<br>b: 1.53, m	13	10, 14, 24
13	22.6, CH_2	a: 1.84, m<br>b: 1.96, m	12	11, 12, 14, 15, 19
14	137.2, C			
15	133.6, C			
16	27.3, CH_2	a: 2.07, m<br>b: 1.87, m	17	14, 15
17	21.3, CH_2	a: 1.68, m<br>b: 1.56, m	16, 18	10, 14, 15, 17, 18
18	41.1, CH_2	a: 1.50, m<br>b: 1.35, m	17	14, 16, 17, 19, 20, 25
19	35.7, C			
20	29.9, CH_3	0.96, s		14, 18, 19, 25
21	24.1, CH_3	1.38, s		2, 3, 4
22	25.4, CH_3	1.22, s		2, 6, 7, 8
23	24.2, CH_3	0.93, s		9, 10, 11, 15
24	21.4, CH_3	0.88, s		6, 10, 11, 12
25	28.2, CH_3	1.03, s		14, 18, 19, 20
4-OMe	57.4, CH_3	3.45, s		4
1'	152.9, C			

Table S10: NMR data of Darumycin G (**8**) in CD_3OD.

No	δ_C [ppm]	δ_H [ppm], mult (J)	HHCOSY	HMBC (H→C)
1	116.3, CH	5.92, s		1', 2, 3, 7
2	133.0, C			
3	59.8, C			
4	80.0, CH	3.60, dd (10.7, 4.1)	5	2, 3, 5, 6, 21
5	29.1, CH_2	a: 1.58, m<br>b: 1.72, m	4, 6	3, 4, 6, 7
6	41.8, CH	1.59, m	5	2, 4, 8, 22, 24
7	38.4, C			
8	36.1, CH_2	a: 1.22, m<br>b: 1.55, m	9	2, 6, 9, 10, 22
9	28.3, CH_2	a: 1.90, m<br>b: 1.70, m	8	7, 11, 15, 23
10	42.9, C			
11	38.6, C			
12	22.6, CH_2	a: 1.88, m<br>b: 1.92, m	13	11, 13, 14, 24
13	31.2, CH_2	a: 1.56, m<br>b: 1.52, m	12	11, 12, 14, 15, 19
14	137.2, C			
15	133.5, C			
16	27.3, CH_2	a: 2.07, m<br>b: 1.86, m	17	14, 15, 17, 18
17	21.3, CH_2	a: 1.67, m<br>b: 1.56, m	16, 18	15, 16, 19
18	41.1, CH_2	a: 1.50, m<br>b: 1.35, m	17	14, 16, 17, 19, 20, 25
19	35.6, C			
20	29.9, CH_3	0.95, s		14, 18 ,19 ,25
21	23.5, CH_3	1.40, s		2, 3, 4
22	25.4, CH_3	1.21, s		2, 6, 7, 8
23	24.2, CH_3	0.92, s		9, 10, 11, 15
24	21.4, CH_3	0.87, s		6, 10, 11, 12
25	28.1, CH_3	1.02, s		14, 18, 19, 20
1'	152.8, C			

Table S11: Summary of DALI search using the structure of DarM in complex with SAH as input.

PDB ID	Description	Classification	Organism	Z score	RMSD	% ID
5egp	TmtA, gliotoxin inactivation	Class I S-methyltransferase	fungi	24.3	3.0	18%
5w7k	OxaG, oxaline biosynthes	Class I O-methyltransferase	fungi	23.5	3.0	16%
5w7m	RoqN, glandicoline biosynthes	Class I O-methyltransferase	fungi	22.5	3.1	17%
3ccf	n.D.	n.D.	bacteria	21.3	2.6	22%
6f5z	Trm112	Class I methyltransferase	archaea	21.2	2.9	20%
2p35	*trans*-Aconitate 2-methyltransferase	n.D.	bacteria	20.8	2.6	19%
3dh0	SAM-dependent methyltransferase	n.D.	bacteria	20.6	2.1	18%
7veo	JHAMT3, juvenile hormone biosynthesis	Class I methyltransferase	eukarya	20.4	2.8	14%
6uv6	AtmM, rebeccamycin biosynthesis	Class I O-methyltransferase	bacteria	19.9	2.8	21%
8zsa	4VPMT2, 4VA biosynthesis	Class I O-methyltransferase	eukarya	19.8	3.1	17%
6lyh	CkTcS, 1,3,7-trimethyluric acid modification	Class I N-methyltransferase	plant	19.8	3.1	14%
2glu	YcgJ	n.D.	bacteria	19.7	3.3	17%
4obx	Coq5, coenzyme Q biosynthesis	Class I C-methyltransferase	eukarya	19.6	3.0	16%
1m6e	SAMT	Class I O-methyltransferase	plant	19.4	3.2	15%
5dpm	UbiG	n.D.	bacteria	19.3	2.6	19%
7ec0	JHAMT, juvenile hormone biosynthesis	Class I O-methyltransferase	eukarya	19.3	3.1	14%
4kri	HcPMT2	Class I N-methyltransferase	eukarya	19.1	2.8	21%
8x8i	AbBioc, malonyl-ACP methyltransferase	Class I O-methyltransferase	bacteria	19.1	3.4	14%
6c8r	CrLAMT, loganic acid biosynthesis	Class I O-methyltransferase	plant	19.0	3.2	12%
8zc8	MitM, mitomycin biosynthesis	n.D.	bacteria	18.9	3.0	23%

Table S12: Summary of DALI search using an AlphaFold 3 model of DarG as input.

PDB ID	Description	Classification	Organism	Z score	RMSD	% ID
6uv6	AtmM, rebeccamycin biosynthesis	Class I O-methyltransferase	bacteria	31.7	2.5	37%
8zc8	MitM, mitomycin biosynthesis	n.D.	bacteria	30.6	2.3	36%
3bus	RebM, rebeccamycin biosynthesis	Class I O-methyltransferase	bacteria	30.4	2.1	38%
2o57	Putative sarcosine dimethylglycine methyltransferase	n.D.	algae	29.7	2.9	20%
1l1e	PcaA, mycolic acid biosynthesis	Class I C-methyltransferase	bacteria	29.0	2.1	19%
3ha7	MMAA4	n.D.	bacteria	28.4	2.2	19%
4pne	SpnF, spinosyn biosynthesis	cyclase	bacteria	28.3	2.7	30%
1kpg	CmaA1, mycolic acid biosynthesis	Class I C-methyltransferase	bacteria	28.2	2.2	19%
7lxi	UmaA, mycolic acid biosynthesis	Class I C-methyltransferase	bacteria	28.1	2.6	20%
4f84	Gernanyl diphosphate methyltransferase	Class I C-methyltransferase	bacteria	27.5	1.9	23%
4krh	HcPMT2	Class I N-methyltransferase	eukarya	27.5	3.1	28%
7fbh	BezA, geranyl diphosphate methyltransferase	Class I C-methyltransferase	bacteria	27.4	2.5	23%
5gm1	TleD, teleocidin biosynthesis	Class I C-methyltransferase	bacteria	26.0	2.4	32%
6p3m	G*f*TNMT, tetrahydroprotoberberine	Class I N-methyltransferase	plant	25.6	3.6	19%
6gkv	CNMT, coclaurine biosynthesis	Class I N-methyltransferase	plant	24.9	3.9	20%
5koc	PavNMT, pavine biosynthesis	Class I N-methyltransferase	plant	24.5	2.6	17%
6ecu	StiD, stigmatellin biosynthesis	Class I O-methyltransferase	bacteria	22.3	2.8	19%
6f5z	Trm112	Class I methyltransferase	archaea	19.8	3.1	23%
7wdw	DsyB, dimethylsulfoniopropionate biosynthesis	Class I S-methyltransferase	bacteria	18.3	2.5	17%
8tjj	RedM, reductasporine biosynthesis	Class I N-methyltransferase	bacteria	17.2	3.2	18%

Table S13: Methyltransferases embedded in *gro* like BGCs.

Organism	Methyltransferase	Accession no. (GenBank)	DarG homolog	Accession no. (GenBank)	DarM homolog	Accession no. (GenBank)
Kitasatospora sp. NPDC096077	**Met1**	MER7844818.1	-	-	-	-
Lentzea rhizosphaerae	-	-	**Met2G**	MFC3892460.1	**Met2M**	MFC3892454.1
Micromonospora rubida	-	-	**DarG**	WP_161689877.1	**DarM**	WP_161689883.1
Micromonospora sp. CA-111912	-	-	**Met3G**	XVT37669.1	**Met3M**	XVT37675.1
Micromonospora sp. NBC_01405	-	-	**Met4G**	WTY81274.1	**Met4M**	WTY81268.1
Micromonospora sp. NPDC001898	-	-	**Met5G**	MFF3868181.1	**Met5M**	MFF3868175.1
Micromonospora sp. NPDC002296	-	-	**Met6G**	MER5704886.1	**Met6M**	MER5704892.1
Micromonospora sp. NPDC020750	-	-	**Met7G**	MFI1195271.1	**Met7M**	MFI1195265.1
Micromonospora sp. NPDC020751	-	-	**Met8G**	MFI1276459.1	**Met7M**	MFI1276465.1
Streptomyces cyaneofuscatus	**Met9**	MGW5186865.1	-	-	-	-
Streptomyces microflavus	**Met10**	MFD5901906.1	-	-	-	-
Streptomyces noursei	**Met11**	MGW5114136.1	-	-	-	-
Streptomyces parvulus	**Met12**	RDD89939.1	-	-	-	-
Streptomyces pilosus	**Met13**	MFE0550060.1	-	-	-	-
Streptomyces sioyaensis	**Met14**	WP_432000008.1	-	-	-	-
Streptomyces sp. MH60	**Met15**	PPS88248.1	-	-	-	-
Streptomyces sp. NBC_01351	**Met16**	WSF31771.1	-	-	-	-
Streptomyces sp. NPDC005549	**Met17**	MET7319022.1	-	-	-	-
Streptomyces sp. NPDC020571	**Met18**	MFI1758179.1	-	-	-	-
Streptomyces sp. NPDC050388	**Met19**	MEV0689121.1	-	-	-	-
Streptomyces sp. RK23	**Met20**	MBQ1008771.1	-	-	-	-

Streptomyces sp. RK74B	**Met21**	MBQ0966293.1	-	-	-	-
Streptomyces sp. RS2	**Met22**	MCW1094477.1	-	-	-	-
Streptomyces sp. ScaeMP	**Met23**	WP_158689994.1	-	-	-	-
Streptomyces sp. SID5594	**Met24**	MZF58105.1	-	-	-	-
Streptomyces sp. SID5914	**Met25**	MZG16120.1	-	-	-	-
Streptomyces sp. VTCC 41912	**Met26**	MFL4492904.1	-	-	-	-
Streptomyces sp. x-19	**Met27**	WP_411123112.1	-	-	-	-
Streptomyces tendae	**Met28**	MGW5764291.1				

Table S15: Bacterial strains used in this work.

Strains	Description	Reference or Source
Micromonospora rubida JCM 32386	Type strain	Japan Collection of Microorganisms, RIKEN BRC
S. albus Del14	Cluster-free heterologous host strain	[14]
S. lividans DelYA9	Cluster-free heterologous host strain	[15]
S. albus Del14 cos15AAmInt-5E12	S. *albus* strain Del14 containing the cos15AAmInt-5E12 vector	This work
S. lividans DelYA9 cos15AAmInt-5E12	*S. lividans* ΔYA9 containing the cos15AAmInt-5E12 vector	This work
S. albus Del14 cos15AAmInt-5E12del-darG	S. *albus* strain Del14 containing the cos15AAmInt-5E12del-darG vector, with the deletion of the methyltransferase *darG* gene	This work
S. albus Del14 cos15AAmInt-5E12del-darM	S. *albus* strain Del14 containing the cos15AAmInt-5E12del-darM vector, with the deletion of the methyltransferase *darM* gene	This work
S. albus Del14 cos15AAmInt-5E12del-darA	S. *albus* strain Del14 containing the cos15AAmInt-5E12del-darA vector, with the deletion of the cyclase *darA* gene	This work
S. albus Del14 cos15AAmInt-5E12del-darC	S. *albus* strain Del14 containing the cos15AAmInt-5E12del-darC vector, with the deletion of the cytochrome P450 oxygenase *darC* gene	This work
S. albus Del14 cos15AAmInt-5E12del-darG/darM	S. *albus* strain Del14 containing the cos15AAmInt-5E12del-darG/darM vector, with the double deletion of the *darG* and *darM* genes	This work
S. albus Del14 cos15AAmInt-5E12del-darK	S. *albus* strain Del14 containing the cos15AAmInt-5E12del-darK vector, with the deletion of the hypothetical protein *darK* gene	This work
S. albus Del14 cos15AAmInt-5E12del-darC/darG	S. *albus* strain Del14 containing the cos15AAmInt-5E12del-darC/darG vector, with the double deletion of the *darC* and *darG* genes	This work
S. lividans ΔYA9 cos15AAmInt-5E12del-darA	*S. lividans* ΔYA9 containing the cos15AAmInt-5E12del-darA vector, with the deletion of the cyclase *darA* gene	This work
S. lividans ΔYA9 cos15AAmInt-5E12del-darI	*S. lividans* ΔYA9 containing the cos15AAmInt-5E12del-darI vector, with the deletion of the Rieske *darI* gene	This work
E. coli ET12567 pUB307	Donor strain for intergeneric conjugation	[16]
E. coli GB05-red	Strain used for Red/ET	[17]
E.coli EPI300-T1R	Strain used for Construction of inducible-copy-number genomic libraries using the CopyControl™ Cloning System, with clones that are resistant to contaminating phage T1 and T5	Lucigen
E. coli DH5α	Strain used for site-directed mutagenesis plasmid amplification.	NEB
E. coli Lemo21 (DE3)	*fhuA2 [lon] ompT gal (λ DE3) [dcm] ΔhsdS/* pLemo(Cam^R) *λ DE3 = λ sBamHIo ΔEcoRI-B int::(lacI::PlacUV5::T7 gene1) i21*	NEB

	Δ*nin5* pLemo = pACYC184-*PrhaBAD-lysY*, Host for gene expression and protein purification	

Table S16: Plasmids used in this work.

Plasmids	Description	Reference or Source
pCos15A_AmInt	pCos15A_gus_AmInt, with *aac(3)IV*, *oriT*, and integrase, where *gus* gene was deleted	[13]
pUC19	Plasmid containing *bla* gene, which encodes a β-lactamase enzyme (ampicillin resistance gene)	[18]
pCos15AAmInt-5E12	Cosmid 5E12 containing darumycin gene cluster	This work
cos15AAmInt-5E12-darG-del	Cosmid 5E12 containing darumycin gene cluster with *darG* (methyltransferase) gene deletion	This work
cos15AAmInt-5E12-darM-del	Cosmid 5E12 containing darumycin gene cluster with *darM* (methyltransferase) gene deletion	This work
cos15AAmInt-5E12-darG/darM-del	Cosmid 5E12 containing darumycin gene cluster with *darG/darM* (both methyltransferases) genes deletion	This work
cos15AAmInt-5E12-darA-del	Cosmid 5E12 containing darumycin gene cluster with *darA* (cyclase) gene deletion	This work
cos15AAmInt-5E12-darC-del	Cosmid 5E12 containing darumycin gene cluster with *darC* (cytochrome P450 oxygenase) gene deletion	This work
cos15AAmInt-5E12-darC/darG-del	Cosmid 5E12 containing darumycin gene cluster with *darC/darG* (cytochrome P450 oxygenase and methyltransferase) genes deletion	This work
cos15AAmInt-5E12-darA-del	Cosmid 5E12 containing darumycin gene cluster with *darA* (cyclase) gene deletion	This work
cos15AAmInt-5E12-darK-del	Cosmid 5E12 containing darumycin gene cluster with *darK* (hypothetical protein) gene deletion	This work
cos15AAmInt-5E12-darI-del	Cosmid 5E12 containing darumycin gene cluster with *darI* (Rieske protein) gene deletion	This work
pET-29a(+)-TEV-DarG	pET-29a(+) derived, *darG* gene cloned with N-terminal His_6-tag and TEV site	GenScript
pET-29a(+)-TEV-DarM	pET-29a(+) derived, *darM* gene cloned with N-terminal His_6-tag and TEV site	GenScript

Table S17: Primers used in this work.

Primer name	Sequence (5'-3')	Description	Source
cos_5E12_L_F<br>cos_5E12_L_R	GCTGCTGTCATTGGTGTGC<br>GCAGGACCTCATCGACGA	To sequence the insert containing the darumycin cluster from the left side of the cluster	This work
cos_5E12_R_F<br>cos_5E12_R_R	TTCAGACCGAAGTTGCCCAA<br>GCCTCATCTGGAAGTGGGAC	To sequence the insert containing the darumycin cluster from the right side of the cluster	This work
rubida_groF_F<br>rubida_groF_R	GCAGTTGGACGGCGATGA<br>TCGAATTCAACGACCCGGAC	To test for *groF* homolog gene presence	This work
rubida_groH_F<br>rubida_groH_R	GCGGTAGATGATTCCCACGT<br>TCTTCCGGGAGTACGTGACC	To test for *groH* homolog gene presence	This work
del-darG-F	CGAGGACATGCTCGGCGACAGTCATCACTTCGGGTACTTCCCGGACGGCGGTTTAAACAGCTGTTCCGGGGATCCGTC	For construction of cos15AAmInt-5E12-darG-del	This work
del-darG-R	ATGAGCCCCCGCACCTCCTGCGCGTAGTCGCCCAGCGCCGAGATCCGCTCGTTTAAACTGTAGGCTGGAGCTGCTTCG		
del-darM-F-Bst	TCAAACCGGTCGGCAGCAGGCTCGTCGGCCACGCGGCGGTAGCGCCTGGTGTATACCGTCAGGTGGCACTTTTCG	For construction of cos15AAmInt-5E12-darM-del	This work
del-darM-R-Bst	AGCAGACCGGGTTCGTGAGCCGGATACAACCGTCGGCGTCCCGCCGGGTGGTATACTTACCAATGCTTAATCAGTG		
del-cycl-F	TAGAGATAGTTGACCAGGGGCGTCAGCGGATAGTCCTGGTGCGTCACGCTGTTTAAACCGTCAGGTGGCACTTTTCG	For construction of cos15AAmInt-5E12-darA-del	This work
del-cycl-R	CTACCGCGGCTTCAAGGAACACACCTACGGGATGCCGATCGCGGCGCTGGGTTTAAACTTACCAATGCTTAATCAGTG		
del-P_450-F	GTGGCCTCGACGTAGCTGAACTGGTCGCCGATGCACTTGCGGGCGCCGCCGTTTAAACCGTCAGGTGGCACTTTTCG	For construction of cos15AAmInt-5E12-darC-del	This work
del-P_450-R	CCTCACGCTCGGGCCGGTGCGGATCGTGGTCGTCTGCGACCCGGAACTCAGTTTAAACTTACCAATGCTTAATCAGTG		
del-ubiq-F	TGCCCGGCGACGACCCCTCCGTCGTCCCGTGGTACGTCCGGCTCCTGGAGGTTTAAACCGTCAGGTGGCACTTTTCG	For construction of cos15AAmInt-5E12-darK-del	This work
del-ubiq-R	TGGCGTAGAAGCGGAGCAGGTCAGGGGTGTGGATGCCCAACTTCGCCCGAGTTTAAACTTACCAATGCTTAATCAGTG		
del-Rieske-3-F	AAAGCGATCACGCTGTTCGGCCGCCGCCTGGTGGCCTGGCGGGACGGCAGGTTTAAACCGTCAGGTGGCACTTTTCG	For construction of cos15AAmInt-5E12-darI-del	This work
del-Rieske-3-R	GTCGCCGGGTTCGAGGGTGTTGAAGATCGGCAGATCCTGGTTCGACGTGAGTTTAAACTTACCAATGCTTAATCAGTG		

chk-MT1-del-F<br>chk-MT1-del-R	GAGGTGGTGGTGAAGGAGTT<br>ACCAGGTAGACCCCGAACT	To confirm *darG* gene deletion	This work
chk-MT2-del-F<br>chk-MT2-del-R	AACCTGGGCACGTTCGAG<br>CAATCGTCCGGCTGCCAC	To confirm *darM* gene deletion	This work
chk-cyclase-del-F<br>chk-cyclase-del-R	CGGACCAGATCGCATAGGC<br>GAGCACTACACACAGGACGT	To confirm *darA* gene deletion	This work
chk-P450-del-F<br>chk-P450-del-R	GCCTCGAACTTGCCCTTGTA<br>TGAGCTGGGAAAACGGTGAG	To confirm *darC* gene deletion	This work
chk-ubi-del-F<br>chk-ubi-del-R	GACGTGTTGGTCGCCAGCTA<br>GGACGCCCTCATGCTTGA	To confirm *darK* gene deletion	This work
chk-Rieske3-del-F<br>chk-Rieske3-del-R	ACTTCGTCGGCATCCTGC<br>CCAGCTCGGTGTGGTATTGG	To confirm *darI* gene deletion	This work
DarM-Asp186Asn-fwr	GGATGCAGCCAACGAAGTGGATGC	DarM site-directed mutagensis	This work
DarM-Asp186Asn-rev	AGGGCATCGGCGGTG	DarM site-directed mutagensis	This work
DarM-Asp186Ala-fwr	GGATGCAGCCGCGGAAGTGGATGC	DarM site-directed mutagensis	This work
DarM-Asp186Ala-rev	AGGGCATCGGCGGTG	DarM site-directed mutagensis	This work
DarM-Leu229Phe-fwr	TGCACAGGGCTTTCGCCTGTTTT	DarM site-directed mutagensis	This work
DarM-Leu229Phe-rev	TAGGTCCAGCGCACAAATTC	DarM site-directed mutagensis	This work
DarM-Gln227Ala-fwr	GACCTATGCAGCGGGCCTGCGCC	DarM site-directed mutagensis	This work
DarM- Gln227Ala -rev	CAGCGCACAAATTCTTCAC	DarM site-directed mutagensis	This work
DarM-Ala185Trp-fwr	CCTGGATGCATGGGATGAAGTGGATG<br>CAATTACCC	DarM site-directed mutagensis	This work
DarM- Ala185Trp-rev	GCATCGGCGGTGCCT	DarM site-directed mutagensis	This work
DarM-Phe24Trp-fwr	CCCGGCCTTTTGGAAACCGGTGG	DarM site-directed mutagensis	This work
DarM- Phe24Trp-rev	CCAATTTCATCATACACATCTGCC	DarM site-directed mutagensis	This work

Table S18: DarG and DarM nucleotide and amino acid sequence.

Name	Ordered nucleotide sequence (accesssion no. WP_161689877.1)	Amino acid sequence
**DarG**	GTTAATGATAGTTATCGTCCGGATCGTGAAAGTGTTGGCGAAATGTAT GATAAAATTACCGTGCTGAGTGAAGATATGCTGGGCGATAGCCATCAT TTTGGTTATTTTCCGGATGGCGGTTATGAAGATGTGGGCATGCGCGAA GCCGCAGATAATCTGACCGATCTGCTGATTGAACGCCTGGCCCTGCGC CCGGGTCAGAGAGTTCTGGATCTGGGCTGTGGTACCGGTCGCCCGGCC GTTCGTCTGGCTGAAACCGTTCCGGTTGAAGTGGTGGGTATTACCATTA GCCAGGCCCAGGTTGATATTGCAACCGAACGCGCCGCCGGCGCCGGTG TGGCAGACCGTGTGAGATTTGAACTGGTGGATGCAACCAGTATGCCGT ATGATAAAGATAGTTTTGATGCAGTGCTGGCCATTGAAAGCCTGGAAC ATCTGCCGGATCAGGCCGGTGTTCTGAATGCAATTGCAGGTATGCTGC GTCCGGGTGGCCGTCTGGTGGTTGCCCAGCCTGTGCTGCAGCGCGTTG ATGATCATGAACGCGATGATTTTGTTCGTCAGCTGTATCAGAGTTATCA TCTGGCCCAGCCGCCGTATCTGGATGAATATCCGCAGCTGTTTGCCCGT GCCGGCCTGCGTACCCGTTATGTGGAAGATATCTATGAACAGGTTATT CCGCCGACCCTGGCAGTTGTTGATGCCTGGGTTCGTGCCAATAGCGAA CGCATTAGTGCCCTGGGTGACTATGCCCAGGAAGTTCGCGGTCTGATT GATGGCATGCGCACCTTTGCAGAACTGCCGGGCGTTGGTTTTGCAGTTC TGGTTGCAGGTAAAGGTTAA	MGSSHHHHHHSSGENLYFQGGVNDSYRPDRES VGEMYDKITVLSEDMLGDSHHFGYFPDGGYED VGMREAADNLTDLLIERLALRPGQRVLDLGCG TGRPAVRLAETVPVEVVGITISQAQVDIATERA AGAGVADRVRFELVDATSMPYDKDSFDAVLAI ESLEHLPDQAGVLNAIAGMLRPGGRLVVAQPV LQRVDDHERDDFVRQLYQSYHLAQPPYLDEYP QLFARAGLRTRYVEDIYEQVIPPTLAVVDAWV RANSERISALGDYAQEVRGLIDGMRTFAELPGV GFAVLVAGKG*
	**Ordered nucleotide sequence (accesssion no. WP_161689883.1**	**Amino acid sequence**
**DarM**	ATGACCGATCAGCTGTGGGCCGTTTATGATACCGTGGCAGATGTGTAT GATGAAATTGGCCCGGCCTTTTTCAAACCGGTGGGTAGTCGCCTGGTG GGTCATGCAGCCGTGGCCCCTGGTGAATGCATTCTGGATGTGGGCACC GGTCGTGGCGCCGTGCTGTTTCCGGCAGCAGCCGCAACCGGTCCGGAT GGTGAAGTTACCGGTATTGATCTGGCCCCGCGTATGATTGAATTTCTGC GTGATGATATTGCCCGCCGCGGCACCGTGAATGTTCGTGCCGTGGTTAT GGATGGCCGCGCACCGACCTTTGCCCCGGGTACATTTGATCTGATTACC GCCAGTATGAGTCTGCCGCTGGTGCCGGATCATCCGGCAACCCTGGCA AATTATGCCCGTCTGCTGCGTCCGGGCGGCCGTATTGCACTGGCCGTTC CGGGTGCCGGCACCGAAGGTGACGGTAGCCAGATTGCACTGGAATTTT TCCAGCGTGCAATGCGTGATTTTGCCGCAACCGATGCAGCAGCAGGCA CCGCCGATGCCCTGGATGCAGCCGATGAAGTGGATGCAATTACCCGTC ATCTGCGTGGCGCAGGCTTTGCAGAACCGCGTATTGTTGTTGAAGATG TTCCGCTGGTTGCCGCCAGTGGTGAAGAATTTGTGCGCTGGACCTATGC ACAGGGCCTGCGCCTGTTTTGGGATATGATTCCGCAGGAACGCCGCCC GGATCTGGAACGTCAGCTGGCAGCCGAACTGGATACCCGTCGCGATGC AGATGGTTGCATTCGCCTGACCAATCCGGTGTGCTTTATTGTTGCAACC AGCCCGGATCCGAGTCAGGAACAGGTGCCGCGTTGCAGCAGTGGTACC CGTTAA	MGSSHHHHHHSSGENLYFQGGMTDQLWAVYD TVADVYDEIGPAFFKPVGSRLVGHAAVAPGECI LDVGTGRGAVLFPAAAATGPDGEVTGIDLAPR MIEFLRDDIARRGTVNVRAVVMDGRAPTFAPG TFDLITASMSLPLVPDHPATLANYARLLRPGGRI ALAVPGAGTEGDGSQIALEFFQRAMRDFAATD AAAGTADALDAADEVDAITRHLRGAGFAEPRI VVEDVPLVAASGEEFVRWTYAQGLRLFWDMIP QERRPDLERQLAAELDTRRDADGCIRLTNPVCF IVATSPDPSQEQVPRCSSGTR*

Table S19: Errors associated with exact mass data reported in this study.

Compound name	Compound number	Formula	Calculated mass (Da) $[M+H]^+$	Observed mass (Da) $[M+H]^+$	Deviation (Δppm)
Darumycin A	**1**	$C_{28}H_{43}N_3O_3$	470.3383	470.3386	+ 0.64
Darumycin B	**2**	$C_{28}H_{41}N_3O_3$	468.3226	468.3229	+ 0.64
Pentaprenylguanidine	**3**	$C_{26}H_{45}N_3$	400.3692	400.3687	- 1.25
Darumycin C	**4**	$C_{27}H_{41}N_3O_3$	456.3226	456.3224	- 0.44
Darumycin D	**5**	$C_{27}H_{41}N_3O_3$	456.3226	456.3225	- 0.22
Darumycin E	**6**	$C_{26}H_{39}N_3O_3$	442.3070	442.3068	- 0.45
Darumycin F	**7**	$C_{27}H_{43}N_3O$	426.3484	426.3483	- 0.23
Darumycin G	**8**	$C_{26}H_{41}N_3O$	412.3328	412.3323	- 1.21

Table S20: Data collection and refinement statistics

	DarM in complex with SAH
**PDB code**	28jb
**Data collection**	
Space group	$P2_12_12_1$
Cell dimensions	
a, *b*, *c* (Å)	38.7, 67.5, 91.9
α, β, γ (°)	90.0, 90.0, 90.0
Wavelength (Å)	0.8731
Resolution (Å)	91.86 – 1.65 (1.68 – 1.65)
$CC_{1/2}$	1.000 (0.895)
I / σI	21.7 (2.3)
Completeness (%)	100.0 (100.0)
Redundancy	13.0 (10.4)
**Refinement**	
Resolution (Å)	91.86 – 1.65
No. reflections	29730 (1548)
R_{work} / R_{free}	0.177 / 0.192
No. atoms	2158
Protein	2022
Ligand/ion	6
Water	130
B-factors	33.13
Protein	32.63
Ligand/ion	41.77
Water	40.43
R.m.s. deviations	
Bond lengths (Å)	0.007
Bond angles (°)	0.95
MolProbity clash score	6.72
Ramachandran (%)	
Favoured	98.07
Allowed	1.54
Outliers	0.39

Statistics for the highest resolution shell are shown in parentheses.

References

1. Evans, P., Scaling and Assessment of Data Quality. *Acta Crystallogr. D Struct. Biol.* **2006,** *62* (1), 72-82.
2. Evans, P. R.; Murshudov, G. N., How Good are My Data and What is the Resolution? *Acta Crystallogr. D Struct. Biol.* **2013,** *69* (7), 1204-1214.
3. The CCP4 Suite: Programs for Protein Crystallography. *Acta crystallographica. Section D, Biological crystallography* **1994,** *50* (Pt 5), 760-3.
4. McCoy, A. J.; Grosse-Kunstleve, R. W.; Adams, P. D.; Winn, M. D.; Storoni, L. C.; Read, R. J., Phaser Crystallographic Software. *J. Appl. Crystallogr.* **2007,** *40* (4), 658-674.
5. Abramson, J.; Adler, J.; Dunger, J.; Evans, R.; Green, T.; Pritzel, A.; Ronneberger, O.; Willmore, L.; Ballard, A. J.; Bambrick, J.; Bodenstein, S. W.; Evans, D. A.; Hung, C.-C.; O'Neill, M.; Reiman, D.; Tunyasuvunakool, K.; Wu, Z.; Žemgulytė, A.; Arvaniti, E.; Beattie, C.; Bertolli, O.; Bridgland, A.; Cherepanov, A.; Congreve, M.; Cowen-Rivers, A. I.; Cowie, A.; Figurnov, M.; Fuchs, F. B.; Gladman, H.; Jain, R.; Khan, Y. A.; Low, C. M. R.; Perlin, K.; Potapenko, A.; Savy, P.; Singh, S.; Stecula, A.; Thillaisundaram, A.; Tong, C.; Yakneen, S.; Zhong, E. D.; Zielinski, M.; Žídek, A.; Bapst, V.; Kohli, P.; Jaderberg, M.; Hassabis, D.; Jumper, J. M., Accurate Structure Prediction of Biomolecular Interactions with AlphaFold 3. *Nature* **2024,** *630* (8016), 493-500.
6. Terwilliger, T. C.; Grosse-Kunstleve, R. W.; Afonine, P. V.; Moriarty, N. W.; Zwart, P. H.; Hung, L.-W.; Read, R. J.; Adams, P. D., Iterative Model Building, Structure Refinement and Density Modification with the PHENIX AutoBuild Wizard. *Acta Crystallogr. D Struct. Biol.* **2008,** *64* (1), 61-69.
7. Emsley, P.; Lohkamp, B.; Scott, W. G.; Cowtan, K., Features and Development of Coot. *Acta Crystallogr. D Struct. Biol.* **2010,** *66* (4), 486-501.
8. Adams, P. D.; Afonine, P. V.; Bunkoczi, G.; Chen, V. B.; Davis, I. W.; Echols, N.; Headd, J. J.; Hung, L.-W.; Kapral, G. J.; Grosse-Kunstleve, R. W.; McCoy, A. J.; Moriarty, N. W.; Oeffner, R.; Read, R. J.; Richardson, D. C.; Richardson, J. S.; Terwilliger, T. C.; Zwart, P. H., PHENIX: A Comprehensive Python-Based System for Macromolecular Structure Solution. *Acta Crystallogr. D Struct. Biol.* **2010,** *66* (2), 213-221.
9. Afonine, P. V.; Sobolev, O. V.; Moriarty, N. W.; Terwilliger, T. C.; Adams, P. D., Overall Protein Structure Quality Assessment Using Hydrogen-Bonding Parameters. *Acta Crystallogr. D Struct. Biol.* **2023,** *79* (8), 684-693.
10. Eberhardt, J.; Santos-Martins, D.; Tillack, A. F.; Forli, S., AutoDock Vina 1.2.0: New Docking Methods, Expanded Force Field, and Python Bindings. *J. Chem. Inf. Model.* **2021,** *61* (8), 3891-3898.
11. Hoye, T. R.; Jeffrey, C. S.; Shao, F., Mosher Ester Analysis for the Determination of Absolute Configuration of Stereogenic (Chiral) Carbinol Carbons. *Nat. Protoc.* **2007,** *2* (10), 2451-8.
12. Seco, J. M.; Quiñoá, E.; Riguera, R., The Assignment of Absolute Configuration by NMR. *Chem. Rev.* **2004,** *104* (1), 17-118.
13. Tistechok, S.; Bratiichuk, D.; Sucipto, H.; Gummerlich, N.; Stierhof, M.; Gromyko, O.; Fries, F.; Fedorenko, V.; Müller, R.; Zapp, J.; Myronovskyi, M.; Luzhetskyy, A., Gromomycins: An Unprecedented Class of Triterpene Antibiotics Produced by a Novel Biosynthetic Pathway. *Angew. Chem. Int. Ed.* **2025,** *64* (22), e202422270.
14. Myronovskyi, M.; Rosenkränzer, B.; Nadmid, S.; Pujic, P.; Normand, P.; Luzhetskyy, A., Generation of a Cluster-Free *Streptomyces albus* Chassis Strains for Improved Heterologous Expression of Secondary Metabolite Clusters. *Metab. Eng.* **2018,** *49*, 316-324.
15. Ahmed, Y.; Rebets, Y.; Estévez, M. R.; Zapp, J.; Myronovskyi, M.; Luzhetskyy, A., Engineering of *Streptomyces lividans* for Heterologous Expression of Secondary Metabolite Gene Clusters. *Microb. Cell Fact.* **2020,** *19* (5), 1-16.

16. Flett, F.; Mersinias, V.; Smith, C. P., High Efficiency Intergeneric Conjugal Transfer of Plasmid DNA from *Escherichia coli* to Methyl DNA-Restricting *Streptomycetes*. *FEMS Microbiol. Lett.* **1997,** *155* (2), 223-229.
17. Fu, J.; Teucher, M.; Anastassiadis, K.; Skarnes, W.; Stewart, A. F., A Recombineering Pipeline to Make Conditional Targeting Constructs. In *Methods in enzymology*, Elsevier: 2010; Vol. 477, pp 125-144.
18. Yanisch-Perron, C.; Vieira, J.; Messing, J., Improved M13 Phage Cloning Vectors and Host Strains: Nucleotide Sequences of the M13mp18 and pUC19 Vectors. *Gene* **1985,** *33* (1), 103-19.
